# An encompassing Mendelian randomization study of the causes and consequences of major depressive disorder

**DOI:** 10.1038/s44220-025-00471-x

**Published:** 2025-08-22

**Authors:** Joëlle A. Pasman, Jacob Bergstedt, Arvid Harder, Tong Gong, Ying Xiong, Sara Hägg, Fang Fang, Jorien L. Treur, Karmel W. Choi, Patrick F. Sullivan, Yi Lu

**Affiliations:** 1 Department of Medical Epidemiology and Biostatistics, Karolinska Institute, Stockholm, Sweden; 2 Genetic Epidemiology, Department of Psychiatry, Amsterdam UMC location University of Amsterdam, Amsterdam, the Netherlands; 3 Institute of Environmental Medicine, Karolinska Institute, Stockholm, Sweden; 4 Department of Psychiatry, Harvard Medical School, Boston, MA USA; 5 Center for Precision Psychiatry, Department of Psychiatry, Massachusetts General Hospital, Boston, MA USA; 6 Departments of Genetics and Psychiatry, UNC Chapel Hill, Chapel Hill, NC USA

**Keywords:** Risk factors, Behavioural genetics

## Abstract

Major depressive disorder (MDD) is a prevalent and debilitating disorder whose causes and consequences remain insufficiently understood. Genetic variants can be used to study causal relationships with other traits. Here we reviewed 201 MDD-associated traits and performed genetic correlation analyses for 115 traits, two-sample Mendelian randomization for 89 of them, and one-sample Mendelian randomization for an additional 43 outcomes, applying sensitivity tests and power analyses. We show that MDD liability increases risk for poorer circadian, cognitive, diet, medical disease, endocrine, functional, inflammatory, metabolic, mortality, physical activity, reproduction, risk behavior, social, socioeconomic and suicide outcomes. Most associations were bidirectional, although with weaker evidence for diet, disease and endocrine traits causing MDD risk. These findings provide a systematic overview of traits putatively causally linked to MDD—confirming known links and identifying new ones—and underscore MDD as a cross-cutting risk factor across medical, functional and psychosocial domains.

## Abstract (web summary)

The authors analyze genetic correlations and perform Mendelian randomization to reveal bidirectional links between major depressive disorder and various traits, highlighting its role as an important risk factor across medical, functional and psychosocial domains and identifying potential causal relationships.

## Main

Major depressive disorder (MDD) is a highly prevalent and debilitating mental disorder. It is crucial to increase our understanding not only of what causes MDD but also of how it impacts other aspects of health and well-being. Although treatment provision has increased, prevalence is not decreasing^1^: one in every three women and one in five men experience MDD at some point in their life^2^. MDD substantially impacts quality of life^3^, accounting for 4.3% of years lived with disability^4^. It is related to high treatment use, lower socioeconomic outcomes and adverse clinical outcomes^5^. MDD is also highly comorbid with a range of medical conditions, most notably cardiovascular disease^6^ and overall disease-related mortality^7^. Moreover, MDD is in the top three predictors of suicide ideation (after previous suicide ideation and hopelessness), which in turn is a strong predictor of suicide death^8^. Still, for many outcomes, it is unclear whether MDD is causal. For instance, the association with suicide is at least in part driven by psychiatric comorbidity^9^. For other outcomes, there may be reverse causation—for instance, cardiovascular disease has been identified as both a risk factor for and a consequence of MDD^10^.

Many risk factors have been identified for MDD. For instance, low income, low educational attainment, not having a romantic partner, and loneliness are important social risk factors^11,12^. Psychiatric and medical conditions may also predispose individuals to MDD^5,13^. Diet, substance use and metabolic traits such as body mass index (BMI) are also associated with MDD^14^. Childhood trauma and stressful events may be the most robust risk factors for MDD^15,16^. Many risk factors may be subject to bidirectional associations, and there may be other, noncausal pathways driving the association. For instance, the well-established effect of stressful life events might be largely noncausal^17^. It is notoriously difficult to infer causality from observational data, requiring longitudinal or within-family designs.

By contrast, genetic risk factors fixed at conception cannot be affected by reverse causation. The heritability of MDD has been estimated at 30–50% in family-based studies^5,18^. Genome-wide association studies (GWASs) have been able to identify numerous common variants that contribute to the disorder^19,20^, explaining up to 7% of the variance^21^. Using genetic variants associated with a trait as instruments to capture it alleviates some of the difficulty in testing causal, directional associations. Such Mendelian randomization (MR) analysis can support causal interpretations if instrument variants have an effect on the outcome through their association with the risk factor, without having a relationship with the outcome or unmeasured confounders. Previous broad-ranging MR studies in MDD have largely focused on modifiable risk factors for MDD, or the causal link between MDD and disease outcomes. Support has been found for causal effects in both directions between MDD and health behaviors, cognitive measures, social behaviors, cardiovascular disease and metabolic traits^6,22–25^. In this study, we include traits that have been associated with MDD in previous observational studies, for which large-scale GWAS data were available. In addition, we assess causal effects of MDD on outcomes for which there are currently no GWAS data available (including outcomes related to daily functioning), relying on one-sample MR (OSMR) techniques. This approach allows us to give a systematic overview of putatively causal associations and helps identify which traits should be targeted in the prevention and intervention of MDD and its consequences.

## Results

### Trait selection

**Fig. 1:**
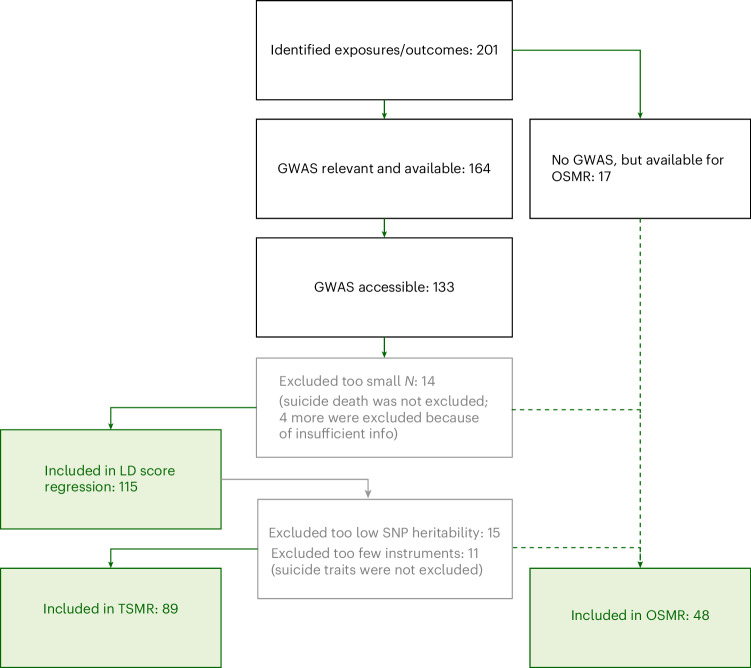
Trait selection procedure. Flow chart with the final number of traits included in each analysis.

We first reviewed the literature to identify potential causes and consequences of MDD. We identified 201 relevant traits (Fig. 1 and Supplementary Table 1). In many cases, the associations were framed as bidirectional, such that MDD could plausibly be the exposure as well as the outcome for the trait of interest. Of the identified traits, 133 were captured in a publicly accessible GWAS (Supplementary Table 2). Genetic correlation with MDD was assessed for 115 traits. Of these, 25 were excluded for TSMR because they had insufficient single-nucleotide polymorphism (SNP) heritability or had too few genome-wide significant SNPs (Fig. 1 and Supplementary Table 2). We made an exception for traits related to suicide, which were kept because they are pertinent outcomes of MDD (although results should be interpreted cautiously, as they were probably underpowered and may suffer from weak instrument bias). In the end, 89 traits were included for TSMR. Of the traits that did not meet the criteria for TSMR, 43 were captured (in a total of 48 variables) in the UK Biobank data and could be included in the OSMR analysis (Supplementary Table 3).

### Genetic correlations

**Fig. 2:**
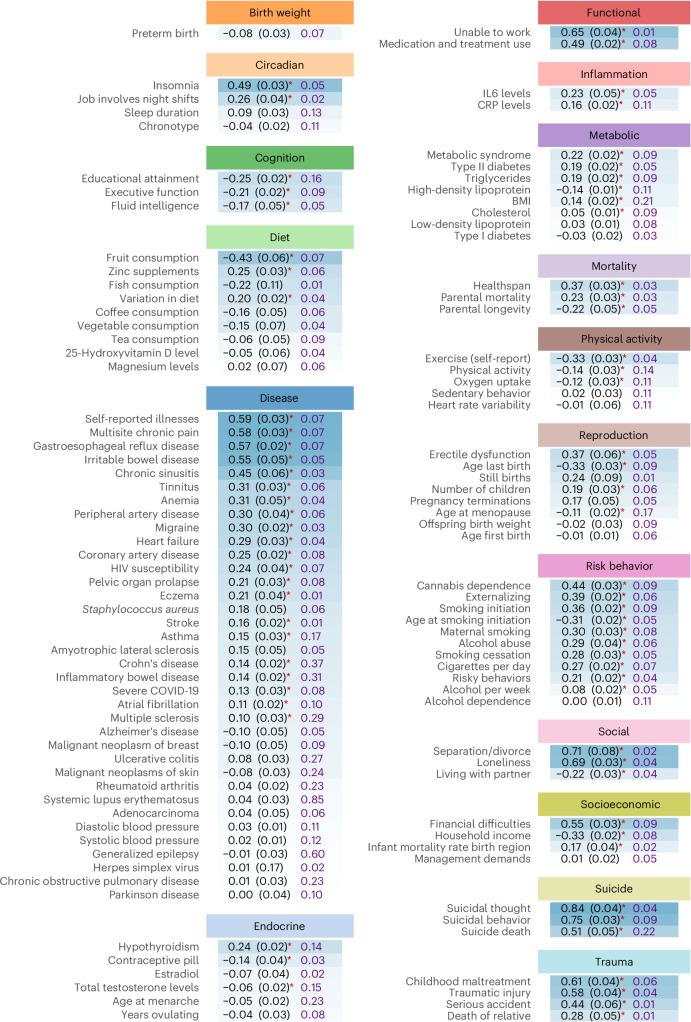
Genetic correlation between MDD and other traits. Estimates of genetic overlap derived with LD score regression are shown in black, accompanied by their standard error. Asterisks indicate a significant correlation at a Bonferroni-corrected two-sided *P* < 4.4 × 10^−4^ (for 115 traits). In blue, the SNP-based heritability is reported as computed using LD score regression (on the liability scale for binary traits, using sample prevalence as a proxy for population prevalence). The shading indicates the strength of the correlation, with the strongest correlations reported on top and in the darkest shade. IL6, interleukin-6; CRP, C-reactive protein.

As a first step, we assessed the genetic correlation between MDD and each trait. For polygenic traits, a causal association is likely to result in a genetic correlation. Results are shown in Fig. 2. The strongest associations (>0.50, in positive or negative direction) were found for subjective health and pain-related disease, trauma and stress, divorce, loneliness, suicide and functional outcomes. Most other traits showed moderate (~0.20–0.50) levels of genetic overlap. Detailed results are presented in Supplementary Table 4.

### TSMR

**Fig. 3:**
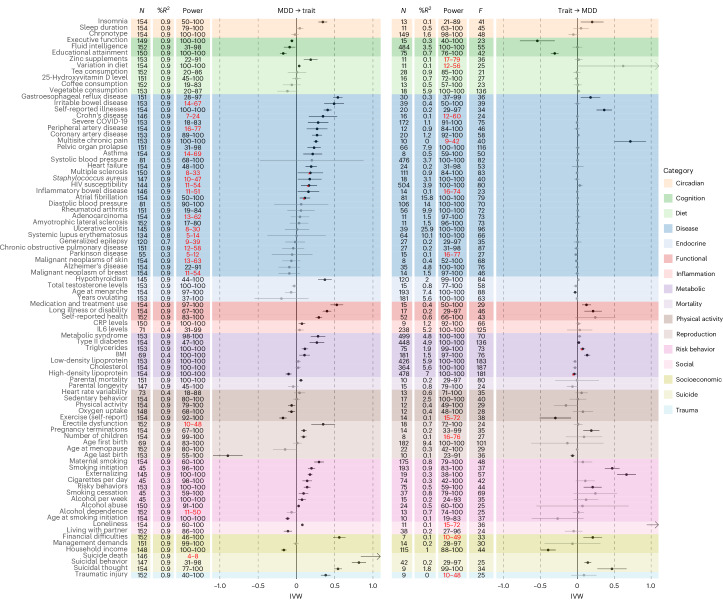
TSMR results. Putatively causal effects of MDD on 89 other disorders and traits (left) and vice versa with MDD as outcome (right). The forest plots show the IVW estimate, with error bars displaying the 95% CI. Estimates printed in black survive correction for multiple testing (two-sided *P*_FDR_ < 0.05). Estimates highlighted with an asterisk are significant estimates that showed evidence of horizontal pleiotropy (at a conservative MR Egger intercept two-sided *P* < 0.05). The columns show the number of instrument SNPs for each analysis (*N*), the percentage variance explained by the instruments in the exposure (%*R*^2^), the achieved level of power under an effect size of *β* = 0.1–0.3 or OR 1.1–1.3 (power), estimates where the upper limit fell short of 80% (printed in red), and the instrument strength of the exposure (*F*). In the left panel, the exposure is always MDD, but *R*^2^ still varies somewhat due to the variation in the number of SNPs present in the outcome summary statistics (*F* varied little (*F* = 40.1–42.6) and is presented in Supplementary Table 4). Note that the estimate of the effect of loneliness on MDD was out of the range of the plot (IVW 2.17, s.e.m. = 0.63). Also note that the summary statistics for anemia, atrial fibrillation, chronic sinusitis, severe COVID-19, cholesterol, alcohol dependence and cannabis dependence were based on analyses in multiancestry populations (with the largest subgroup being European). IL6, interleukin-6; CRP, C-reactive protein.

**Extended Data Fig. 1:**
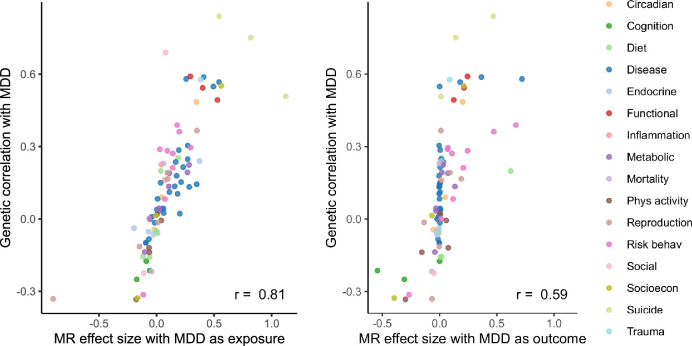
Relationship between the size of the genetic correlation and the TSMR effect size. The left panel shows the MR effect sizes for the analyses with MDD as exposure, the right panel those with MDD as outcome. The overall correlation (r) is labelled at the bottom right.

**Extended Data Fig. 2:**
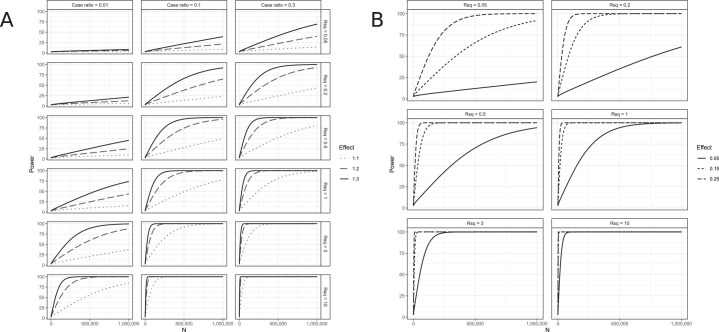
Power graphs visualizing achieved statistical power under different assumptions. Panels **a**) Power for binary traits achieved under 3 different case ratio scenarios (columns; from 1% cases to 30% cases), 6 different R2 scenarios (rows; the amount of variance explained by the instruments in the exposures, in %), and 3 different effect sizes (OR; line type). Panels **b**) Power for continuous traits under 6 different R2 scenarios (panels; the amount of variance explained by the instruments in the exposures, in %), and 3 different effect sizes (β; line type).

**Extended Data Fig. 3:**
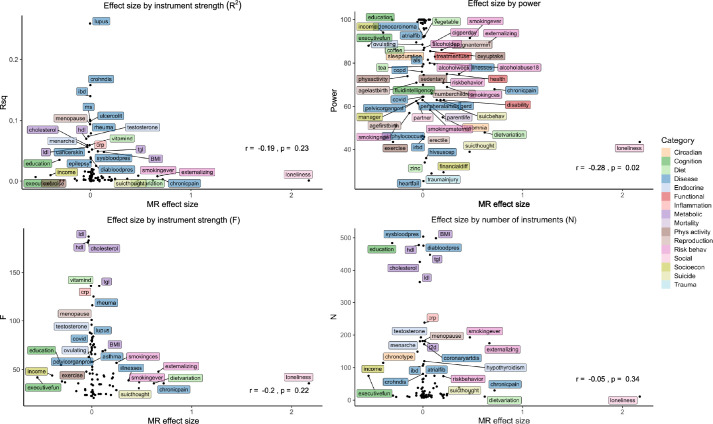
Relationship between different indicators of statistical power and the TSMR effect size with MDD as outcome. The **top left** panel shows the relationship between the TSMR effect size and the variance explained by the instruments in the exposure (R2). The **top right** panel shows the relationship between TSMR effect size and statistical power under an assumed effect size of β=0.2/ OR=1.2. In the **bottom left** panel, the relationship between TSMR effect size and instrument strength F is shown. **Bottom right** panel shows the relationship between TSMR effect size and the number of SNPs included in the instrument.

Next, we conducted two-sample MR (TSMR) to test the putatively causal effect of MDD risk on outcomes and vice versa. The main results (inverse variance-weighted (IVW) estimates) from the TSMR are summarized in Fig. 3, along with the estimated explained variance by the instrument in the exposure *R*^2^ and the statistical power. The full TSMR results including all sensitivity analyses are presented in Supplementary Table 5. Among the 89 disorders and traits studied, we found more support for MDD liability having a causal effect on a trait (57 traits; average absolute effect size *M* = 0.18, average standard error of mean (s.e.m.) = 0.05) than for traits having causal effects on MDD (24 traits, *M* = 0.13, s.e.m. = 0.06). Of the 24 traits with support for an effect on MDD, there was only one that did not also show an effect in the other direction (age at menarche). There was a sizable association between the genetic correlation and the effect size from TSMR, for the analyses with MDD as exposure *r* = 0.81 (95% confidence interval (CI) 0.73–0.87) and with MDD as outcome *r* = 0.59 (95% CI 0.43–0.71; Extended Data Fig. 1). Average power was 57–87% for the analyses with MDD as exposure and 64–94% with MDD as outcome under hypothesized ‘true’ effect sizes *β* = 0.1–0.3 (or odds ratio (OR) 1.1–1.3). A generalized power curve showing which sample sizes and effect sizes would be needed to achieve sufficient power is displayed in Extended Data Fig. 2. The effect sizes should be interpreted in light of these power estimates, although there was no clear positive relationship between power and the MR effect size (Extended Data Fig. 3).

For the circadian traits, we found support for a positive bidirectional effect of MDD on insomnia, such that genetic liability to MDD increased insomnia risk, and vice versa, while there was limited evidence for a similar effect on sleep duration or chronotype from either direction. Likewise, in the cognition category, there was support for a bidirectional causal negative effect for executive function and educational attainment, whereas there was a negative effect of MDD liability on fluid intelligence that was not observed in the other direction. For diet, the statistical power was mostly limited, but we still detected small positive effects of MDD liability on taking zinc supplements and dietary variation. There was evidence for a causal effect of MDD on a number of diseases, including gastrointestinal diseases, coronavirus disease 2019 (COVID-19), chronic pain, a number of cardiovascular diseases and others. There was little evidence for an effect on cancer or neurological disease risk, although power was limited for many of these analyses. In the other direction, there was support only for a causal effect of chronic pain, the self-reported total number of somatic illnesses, and gastroesophageal reflux disease. For endocrine traits, MDD liability increased risk for hypothyroidism, and a younger age of menarche marginally increased MDD risk. There were bidirectional causal associations with most functional outcomes. For the inflammatory traits, there was evidence only for a causal effect of MDD liability on elevated CRP levels. In the metabolic category, there was evidence that MDD increased the risk for all outcomes except low-density lipoprotein and total cholesterol. In the other direction, there was evidence for an effect of BMI and a small effect of type II diabetes. MDD liability had a positive effect on mortality liability (proxied with parental age at death). In the physical activity category, there was a negative effect of MDD on physical activity, exercise and oxygen uptake (a measure of fitness) and a negative effect of exercise on MDD risk. In the reproduction category, there was evidence for a positive effect of MDD liability on erectile dysfunction and the number of children, and bidirectional effects with pregnancy terminations and younger age at last childbirth. There was support for a causal effect of MDD liability on all risk behavior outcomes except alcohol dependence (this analysis was underpowered). In the other direction, there was evidence for a causal effect of externalizing behavior, smoking initiation and risky behaviors on MDD. For the two social traits, MDD lowered chances of living together with a partner and increased loneliness, while loneliness had an out-of-bounds effect on MDD (IVW 2.17, s.e.m. = 0.63, false discovery rate corrected *P* value (*P*_FDR_) = 0.003). In the socioeconomic category, there was evidence for a bidirectional association with a lower income and a higher chance of experiencing financial difficulties. There were effects of MDD liability on all suicide outcomes with an out-of-bounds effect on suicide death (IVW 1.12, s.e.m. = 0.14, *P*_FDR_ = 0.2 × 10^−15^, although power was 4–8%) and smaller but significant effects of suicidal behavior and thought on MDD. Finally, MDD increased the chances of self-reported traumatic injury, whereas traumatic injury had no effect on MDD.

**Extended Data Fig. 4:**
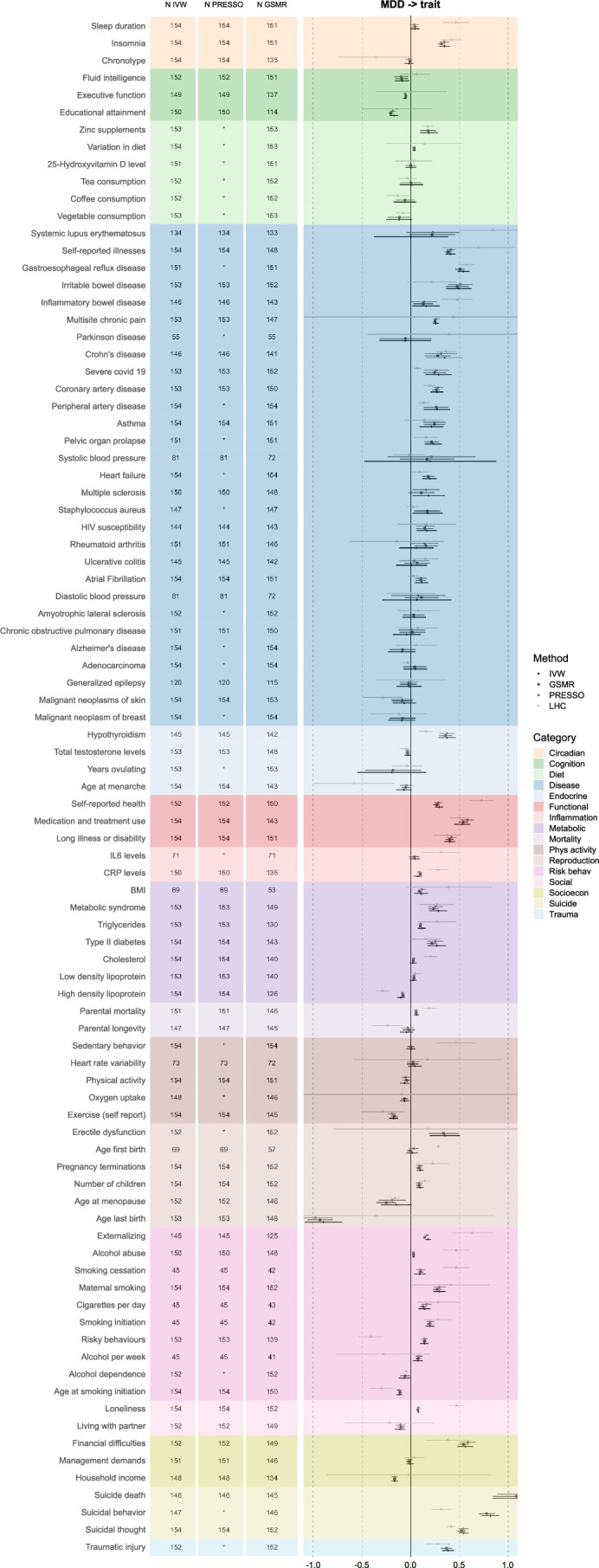
TSMR results with MDD as exposure for the different MR methods. Line color and symbol correspond to the different MR methods. The forest plots show the IVW estimate with 95% CI. The columns show the number of SNPs included in the method (not for LHC, where this number was not directly estimated). *MR PRESSO model could not be estimated for this trait.

**Extended Data Fig. 5:**
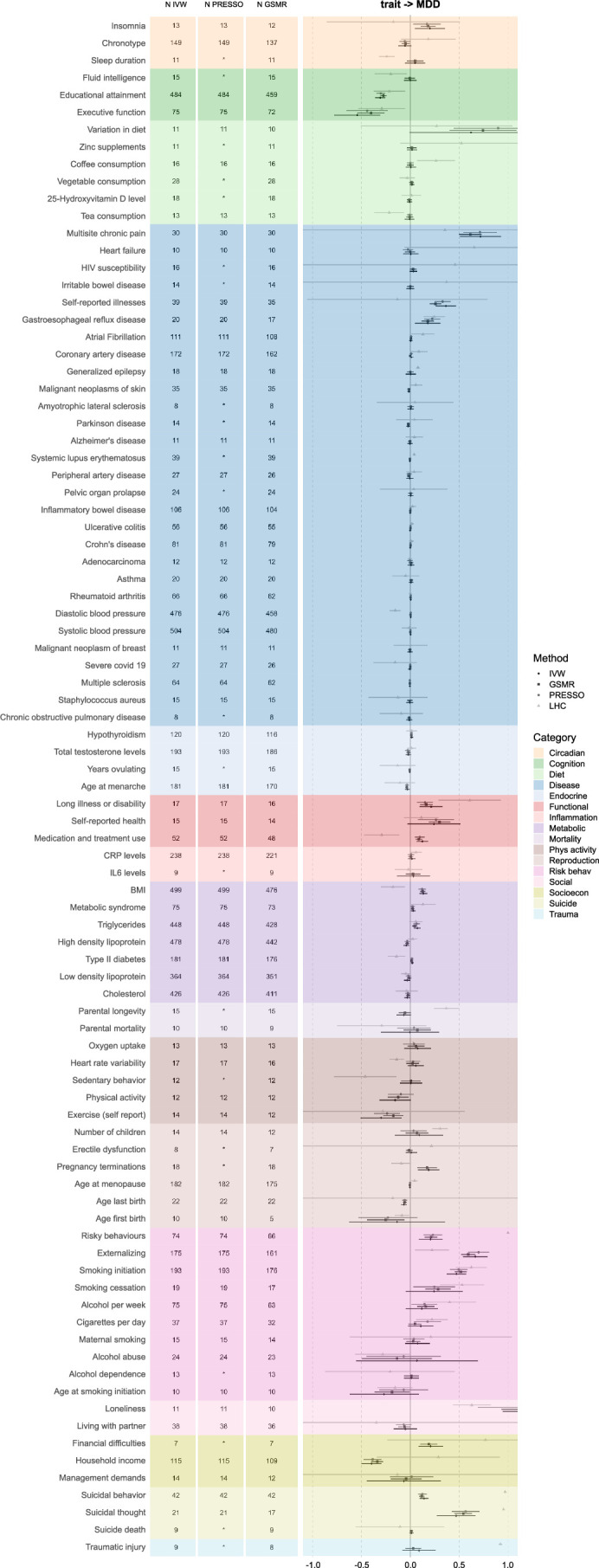
TSMR results with MDD as outcome for the different MR methods. Line color and symbol correspond to the different MR methods. The forest plots show the IVW estimate with 95% CI. The columns show the number of SNPs included in the method (not for LHC, where this number was not directly estimated). *MR PRESSO model could not be estimated for this trait.

Steiger-filtered IVW, MR Egger, simple mode, weighted median and mode estimates were overall consistent with the IVW, although sometimes significance was reached in one and not the other. Results were more pronounced in the generalized summary-data-based MR (GSMR) and MR-pleiotropy residual sum and outlier (MR-PRESSO) analyses (Extended Data Figs. 4 and 5), but CIs were overlapping with the IVW CIs in all analyses. The latent heritable confounder (LHC) analysis, which aims to correct for heritable confounders and sample overlap, mostly showed effects in the same direction, although with less precision. Finally, few trait combinations showed evidence of pleiotropy, as indicated by the MR Egger intercept (Fig. 3, asterisks). Exceptions were the effects of MDD liability on multiple sclerosis, human immunodeficiency virus (HIV) susceptibility and atrial fibrillation. For multiple sclerosis, there was no consistent robust effect across pleiotropy-robust sensitivity analyses, indicating that the effect of MDD was explained through pleiotropic pathways and unlikely to be causal. For HIV susceptibility and atrial fibrillation, there were significant effects for most pleiotropy-robust methods, indicating some evidence for causal effects of MDD. Likewise, the sensitivity analyses indicated some evidence for causal effects of triglycerides and high-density lipoprotein on MDD in spite of showing pleiotropy in the main analysis. Overall, the sensitivity analyses were in line with a causal interpretation of the IVW.

### OSMR

We conducted OSMR to test the effect of MDD on 48 outcomes for which no GWAS was available. Compared with TSMR, the statistical power of OSMR was much more limited (average 7–27% assuming effect sizes *β* = 0.1–0.3 or OR 1.1–1.3) because the instrument SNPs explained only minute amounts of variance in the MDD exposure (*R*^2^ = 0.08%, *F* = 0.58). Nevertheless, the OSMR diagnostics indicated that the MDD instrument did not suffer from weak instrument bias (for the significant findings at *P*_FDR_ < 0.05, average weak instrument test *F* = 39.9, average *P* = 3.5 × 10^−6^).

**Fig. 4:**
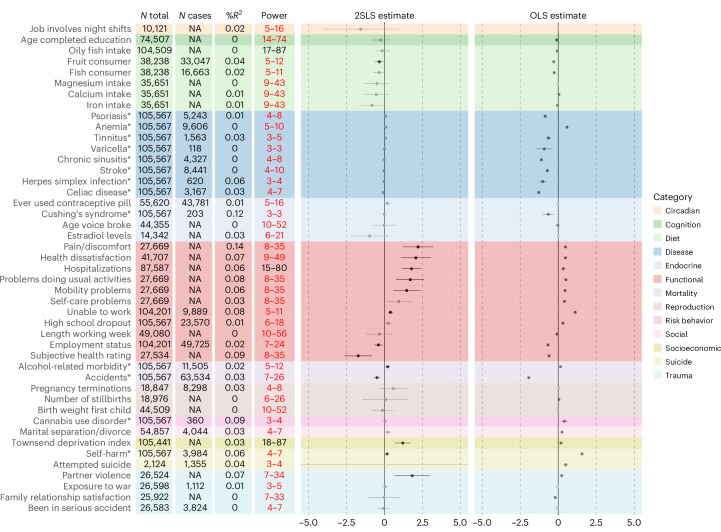
OSMR results. Results from the follow-up OSMR analysis for traits for which there was no GWAS available suited for TSMR. The point estimate represents the 2SLS regression weight, with the error bar representing its 95% CI. Printed in black are estimates that were significant at two-sided *P*_FDR_ < 0.05, with nonsignificant estimates in gray. The instrument was the PRS for MDD, and the outcomes were electronic health-recorded ICD-codes (marked with an asterisk) or survey-based phenotypes from the UK Biobank. Sample size (*N*), the number of cases for binary phenotypes (*N* cases; ‘NA’ for continuous phenotypes), explained variance by the instrument in the outcome (*R*^2^), the achieved level of power under an effect size of *β* = 0.1–0.3 or OR 1.1–1.3 (power), and 2SLS and OLS estimates (in red estimates where the upper limit was below 80%) are given in the columns. Note that *R*^2^ is used here to represent variance explained in the outcome rather than the exposure (as in the TSMR analyses) because the exposure was the same across analyses in the OSMR. The interpretation is accordingly different, such that it represents instrument strength in the TSMR and effect size in the OSMR.

Despite the overall limited power in the OSMR, we found evidence that MDD liability has an effect on a range of functional outcomes, increasing pain, problems with daily life, and hospitalizations, while reducing health satisfaction (Supplementary Table 6). Results are shown in Fig. 4 alongside the ordinary least squares (OLS) regression results relying on observational data only (MDD diagnosis predicting each outcome). The strongest OLS associations were found in the disease and functional outcomes category, with protective effects of MDD liability on disease outcomes. These protective effects are not in line with previous literature and findings from TSMR. They could be the effect of censoring in the hospital record data or, speculatively, be driven by a lower likelihood of an MDD being diagnosed in the presence of a medical disease that can be an alternative explanation for the observed symptoms. Regardless, the OSMR findings suggest that the protective effects of MDD on disease are not causal, while the negative impact on functional outcomes is. In addition, according to the OSMR results, genetic liability to MDD decreased the likelihood of being a fruit consumer and of having an accident and increased the chance of alcohol-related mortality, living in a socially deprived region, engaging in self-harm and experiencing partner violence.

**Extended Data Fig. 6:**
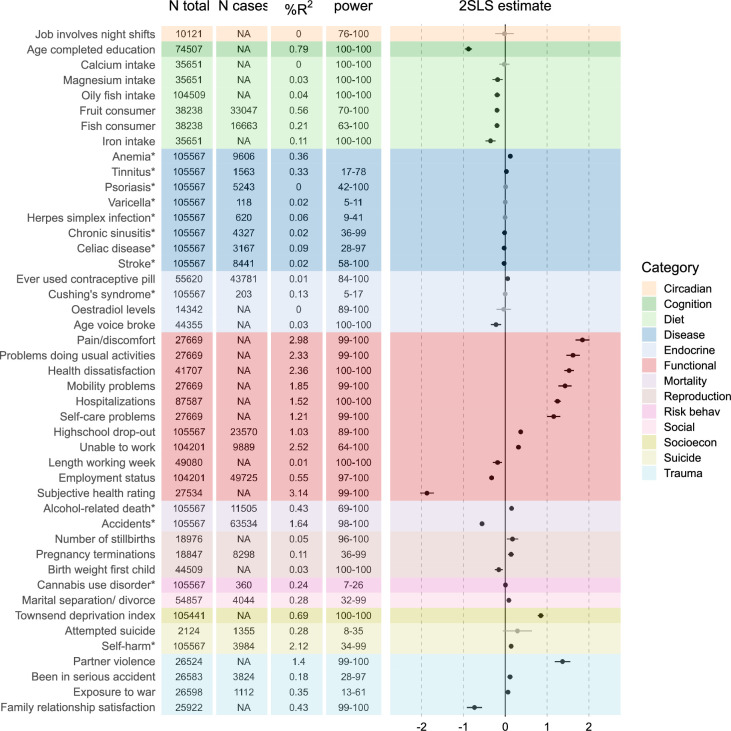
OSMR results using the LDpred2 PRS as instrument for MDD. The outcomes were electronic health recorded ICD-codes (marked with *) or survey-based phenotypes from UK-Biobank. Sample size (N), the number of cases for binary phenotypes (N cases; ‘NA’ for continuous phenotypes), percentage explained variance by the instrument in the outcome (%R2), and the achieved level of power under an effect size of β=0.1–0.3/ OR=1.1–1.3; ‘power’) are given in the columns.

Diagnostic tests and sensitivity analyses indicated that, for most analyses, the OSMR results were robust (Supplementary Table 6). The Wu–Hausman diagnostic indicated that the data were more in line with the two-stage least squares (2SLS) OSMR estimate (with a causal interpretation) than the OLS (noncausal; for the significant relationships, average Wu–Hausman 20.0, *P* = 0.004; full OLS results are in Supplementary Table 7). The sensitivity analyses based on per-instrument association tests were generally in line with 2SLS, without strong evidence for pleiotropy for the outcomes that showed a significant effect in the main OSMR analysis, and generally consistent effects in the IVW and weighted median analysis. The 2SLS analyses using the LDpred2 instrument polygenic risk score (PRS) showed similar results, but the causal estimates had smaller CIs, so that almost all effects reached significance (Extended Data Fig. 6 and Supplementary Table 6). LDpred2 results are discussed in more detail in Supplementary Section 5.

## Discussion

Using a literature-based selection of 115 traits captured in GWAS and 48 additional traits for which no GWAS was available, this study assessed the genetic overlap between MDD and its risk factors and outcomes, applying a range of MR methods to investigate putatively causal associations. We observed genetic overlap between MDD and traits from all categories, with the strongest correlations observed for subjective health and pain-related disease, stress, loneliness, suicide and functional outcomes. In addition to such pleiotropic associations, there was also support for a causal effect of MDD liability on circadian, cognitive, diet, disease, endocrine, functional, inflammatory, metabolic, mortality, physical activity, reproduction, risk behavior, social, socioeconomic, suicide and traumatic injury. In the other direction (in the TSMR analyses only), there were fewer associations, with less evidence for diet, disease and endocrine traits causing MDD risk. The effects were overall robust across sensitivity analyses and consistent in different MR methods, and, to the extent that this can be assessed using current tools, most were robust to violation of the assumptions of MR.

Our TSMR results were more in line with a causal effect of MDD liability on other traits than vice versa, although for 24 traits we found evidence that they act as both consequence and cause to MDD. Especially in the medical disease category, most traits were consequences of MDD, which follows results from an earlier report based on the UK Biobank^24^ but is contrary to other work suggesting the relationship with medical disease is mostly bidirectional^26^. Potential exceptions to this pattern of unidirectional effects of MDD on disease are liability to chronic multisite pain, number of illnesses and gastroesophageal reflux disease, which also had a putatively causal effect on MDD. The first two traits were measured through self-report and may be confounded by a third variable influencing self-report as well as MDD, such as negative recall bias, even though this is not suggested by the sensitivity analyses (that is, no evidence for pleiotropy). Regardless, these findings suggest that there may be something specific about these traits that impacts MDD in a way that other disease traits do not, potentially through psychological and health behavior mechanisms^26^. The OSMR results underline the negative consequences of MDD and show a substantial impact on daily functioning. The wide range of adverse effects of MDD illustrates its universal negative impact across domains that could be mediated through chronic stress, inflammation and unhealthy behavior associated with the condition.

The TSMR analysis shows evidence for many more causal associations than previous studies, which is probably due to the inclusion of a broader range of phenotypes and the use of the newest, high-powered summary statistics for MDD as well as many other traits. For instance, to our knowledge, previous studies have not found consistent evidence for an effect of MDD on diet or infectious disease^22,24,27^. Also, by adding OSMR analyses we were able to show putatively causal effects of MDD liability on traits that have not been (reliably) captured in GWAS. For instance, this method allowed us to find evidence that MDD causally impacts quality of life. OSMR has been little leveraged in previous studies owing to the limited power associated with this method^28^. By relying on a strong GWAS source and a broad range of OSMR tools, we show that OSMR can still be used to show relative support for a causal interpretation.

Interpreting the nature of these causal associations is in many cases not straightforward. For instance, depressive mood is a well-established symptom of hypothyroidism, but we find evidence for hypothyroidism causing MDD, and not the other way around (opposing findings from ref. ^29^). This could be due to the phenotypes used in the source GWASs; especially in the case of MDD it has been argued that broad, inclusive definitions may dilute the genetic signal and amplify unimportant correlations^30^. Even if methodological and statistical issues are probably part of the explanation (for example, there could be some residual pleiotropy not picked up in the sensitivity analyses, especially given MDD’s highly polygenic nature), this Article should be viewed as a starting point to explore such potential pathways to further elucidate the etiology of MDD as well as its consequences. Triangulation research with quasi-experimental designs is needed to confirm our findings and, crucially, to investigate if these causal chains can be broken.

This study has some key strengths and limitations to consider. One important strength is the wide selection of traits, methods, sensitivity analyses and robustness checks used. The MR method is continuously being expanded and improved so that a comprehensive, robust and flexible toolbox has become available. Another key strength is the integration of statistical power, in the selection and creation of instruments, as well as in the interpretation of the findings. Our comprehensive, literature-based selection of traits is a strength as it provided a broad overview of the important risk factors and outcomes. However, it also resulted in limited space to develop hypotheses and interpretations for each tested association, a practice that is recommended in MR research^31^. Likewise, we have not triangulated our findings using different non-MR methods and observational datasets^32^. Our application of stringent selection criteria for trait inclusion ensured statistical power but led to the exclusion of key known risk factors for MDD, especially in the trauma category. Even in the light of these stringent criteria, there was a power imbalance in many cases, such that the MDD GWAS was better powered than the GWAS for the other trait, which could partly explain the pattern where we observed more effects of MDD than vice versa. Another limitation lies in the fact that we did not stratify outcome summary statistics for exposures that are measured in only a subset of individuals. For instance, the effect of alcohol dependence should ideally be tested in alcohol-exposed individuals, and the effect of ovulation should only be assessed in women (although, on the other hand, stratification can lead to collider bias)^33^. Relatedly, most binary traits included in this study are dichotomizations of underlying continuous traits. This can violate the assumption that the instrument influences the outcome solely through the exposure, as it may affect the outcome via the underlying continuous trait without altering the binary variable itself^34^. Although there is no real solution to this issue, the interpretation of results relying on dichotomized exposures as pertaining to the liability to this trait is still valid.

While mindful of these caveats, we conclude that this study provides evidence for putatively causal effects of a wide range of traits on MDD liability, and vice versa. We offer support for the causal nature of known risk factors that can provide efficient targets for prevention and intervention efforts, including a wide range of health behaviors, loneliness, cognitive traits, pain, and gastroesophageal reflux disease. Particularly relevant to highlight are potentially modifiable risk factors, including BMI, loneliness, exercise, and (maternal) smoking. The evidence for a causal effect of MDD liability on a plethora of outcomes, including disease, suicide, and mortality, emphasizes the need for better treatment and suggest that treating MDD will decrease the chances of these serious and costly consequences. Our results demonstrate the key role of MDD as risk factor cross-cutting across medical and psychosocial domains. As such, this study provides strong weight to the call for concerted action aimed at decreasing this highly prevalent and debilitating disorder^35^.

## Methods

We used instruments from publicly available GWAS summary statistics (Supplementary Table 2) to assess putative causal effects between MDD and a broad selection of traits associated with MDD in observational studies, using a quantitative study design. For MDD, we used the newest publicly available GWAS that identified 243 risk loci and explained 7% of the variability in MDD^21^. We used summary statistics leaving out the UK Biobank to limit sample overlap (UK Biobank was included in many of the source GWASs), with 166,773 cases and 507,679 controls. We investigated genetic correlations and OSMR and TSMR associations with a wide range of literature-identified traits.

### Trait selection

The selection of risk factors and outcomes of MDD was guided by literature search and restricted by the availability of GWAS with sufficient sample size and power. To identify risk and outcome factors, we relied on a selection of review papers^11–15,36–39^. For trait groups suggested by the review papers (for example, gastrointestinal disease), we searched the literature additionally for specific reports of associations (for example, gastroesophageal reflux disease). The considered traits (164 traits), their literature source and any available corresponding GWAS sources are summarized in Supplementary Table 1. Subsequently, we searched corresponding GWAS sources for each trait, and included them if they met the following criteria: *N* ≥ 10,000 for continuous traits, or *N* cases ≥5,000 for binary traits; 3% SNP-based heritability; and at least five genome-wide significant SNPs. Although not formally preregistered, these criteria and all downstream methods were established before conducting any analyses. Criteria are explained in more depth in Supplementary Section 1.

### LD score regression

SNP-based heritability (*h*^2^_SNP_), which was used as a selection criterion for the GWAS traits, was derived using linkage disequilibrium (LD) score regression^40^. We used the sample prevalence as an approximation for the population prevalence for the computation of *h*^2^_SNP_ on the liability scale for binary traits. The use of sample prevalence as an approximation for population prevalence was deemed sufficient because we used only *h*^2^_SNP_ as a selection criterion and were not interested in the heritability per se. Subsequently, we used LD score regression to estimate the genetic correlation between MDD and each of the other traits. To correct for multiple testing, we applied a conservative Bonferroni correction of *P* = 0.05/115 (the number of tested traits), resulting in a threshold of *P* < 4.4 × 10^−4^. Although causal associations may exist in the absence of genetic overlap, for polygenic traits it is expected to be accompanied by corresponding genetic correlation^41^. To investigate this, we assessed the relationship between the genetic correlation and the MR effect estimate.

### TSMR

Next, the putative causal associations between MDD and the traits were tested using TSMR. We assessed the effects in both directions, with MDD as exposure and as outcome. In TSMR, both the exposure and the outcome are proxied by GWAS summary statistics. Instruments were created by selecting the independent (clumping at *R*^2^ < 0.001, distance <10,000 kb) genome-wide significant SNPs (*P* < 5 × 10^−8^) that were present in both the exposure and outcome trait GWAS. When there were fewer than ten instruments remaining after this procedure, we increased the *P*-value threshold up to a maximum *P* < 1 × 10^−5^ to include at least ten instruments. After aligning with the outcome data the final number of instruments could still fall below ten, but was never smaller than five. We used the TSMR R package^42^ to conduct IVW meta-analysis of effects, which is the standard in the literature and is the estimate that is reported in the main text.

We use a broad selection of state-of-the-art alternative methods, sensitivity analyses and robustness checks, including MR Egger, weighted median and mode, MR-PRESSO, GSMR, LHC MR and Steiger filtering. Most of these are aimed at identifying or dealing with sources of pleiotropy (shared risk variants). For instance, MR Egger, Steiger filtering and GSMR (with heterogeneity in dependent instrument (HEIDI) filtering) remove instruments that also have an effect on the outcome (horizontal pleiotropy), and LHC models latent unmeasured confounders and corrects for vertical or horizontal pleiotropy with these. Sensitivity analyses are discussed in more detail in Supplementary Section 2.

Although it is often recommended to avoid causal language when describing results from MR analyses (that is, it is preferable to say ‘liability to *X* causes risk for *Y*’), we sometimes use causal language for brevity and clarity throughout the Article, following previous recommendations^43^. Although the scale and specific features of this study did not allow for full adherence to the STROBE checklist (a well-established protocol developed to ensure the quality of MR reports^44^), we have followed it as much as possible throughout the analyses (Supplementary Section 3).

### OSMR

Subsequently, we performed OSMR in the UK Biobank with MDD as exposure and electronic health record- and survey-based outcomes. The UK Biobank is a large-scale biomedical database containing genetic, health and lifestyle information from approximately 500,000 participants. All participants in the UK Biobank study provided informed consent. Participants’ travel expenses were compensated. The OSMR analysis is conducted using individual-level data in two stages. First, SNPs are used as instruments to predict the exposure. Then, the genetically predicted exposure variable is used to predict the outcome. The advantage with respect to TSMR is that traits that have not been captured by GWAS can still be used as outcome. The advantage compared with standard polygenic risk score (PRS) analysis is that assumptions are tested to assess if results are in line with a causal interpretation. We focus on the traits that the literature review suggested were of importance, but could not be tested with TSMR owing to the lack of suitable GWAS sources. We excluded traits that could not plausibly act as an exposure or outcome of MDD because of time-ordering (such as childhood trauma as outcome). The selected outcomes and the MDD exposure are described in Supplementary Section 4.1. Overlaying genetic, MDD and outcome data, information on *N* = 105,567 (year of birth *M* = 1950, 53% females) individuals could be included in the analysis.

For the OSMR analyses, we used a set of two-stage methods as well as MR methods that are also used in the two-sample context. To create the MDD instrument, we created a PRS. Although such polygenic measures increase risk for pleiotropy, the heterogeneous nature of MDD means few variants have strong effects, necessitating their combination to achieve sufficient statistical power. Sensitivity analyses are used to check for pleiotropy (see below). Scores were created with Plink^45^ based on the MDD GWAS leaving out UK Biobank to prevent bias due to sample overlap. Effects of birth year, sex and the first ten principal components capturing genetic ancestry differences were regressed out of the PRS. We used the same SNP selection criteria as for TSMR and created per-individual weighted sum scores of these SNPs. Because this score included only a small subset of SNPs and would still have limited power, we repeated the analyses with a PRS created with LDpred2^46^ (Supplementary Section 4.2).

For each trait, we then computed the OSMR causal estimate using 2SLS regression as implemented in the ivreg R package, relying on medical records of MDD diagnosis as the observed variable (*N* = 28,861 cases, *N* = 103,065 controls, average birth year *M* = 1950, s.d. 8.1) and the PRSs as instrumental variables. Wu–Hausman diagnostics were used to test endogeneity: if all regressors are exogenous, the 2SLS and OLS estimates are consistent. Thus, a significant Wu–Hausman indicates that one or more regressors is endogenous and the 2SLS outcome is preferable over the OLS estimate. Instrument strength *F* was derived in a parallel manner as for TSMR (Supplementary Section 4.3). As a comparison, we also derived the OLS regression estimates for the effect of observed MDD diagnosis on the outcomes (Supplementary Section 4.4). To be able to conduct sensitivity analyses including IVW, weighted median and MR Egger, we also computed the relationship between each instrument SNP and the outcomes in the UK Biobank using Plink association analysis (Supplementary Section 4.5).

### Power and multiple testing correction

We used power calculation to prioritize traits that were more likely to yield reliable results in the TSMR and OSMR^31^. For TSMR, we used the R-app (https://sb452.shinyapps.io/power/)^47^. For OSMR, we used the app (https://shiny.cnsgenomics.com/mRnd/)^48^. To arrive at an approximation of the range of effect sizes in observational studies, we used effect sizes as reported in the literature that we used for trait selection. We chose a plausible effect size range of *β* = 0.1–0.3 or OR 1.1–1.3 (based on literature summarized in Supplementary Table 1). We used our own observations of the variance explained by the instruments (*R*^2^) in the exposure as input for the power analyses.

We used false discovery rate (Benjamini–Hochberg FDR) *P* values to correct for the number of traits tested, separately for the OSMR and TSMR analyses. For interpretation of the TSMR results, we weighed the consistency of effect sizes across different sensitivity analyses rather than only relying on any single *P* value falling below the *P*_FDR_ = 0.05 threshold, which can be caused by pleiotropy or weak instruments.

### Reporting summary

Further information on research design is available in the Nature Portfolio Reporting Summary linked to this article.

## Supplementary information

Supplementary Information Supplementary Information and Tables 1–7.

Reporting Summary

## Data Availability

All GWAS summary statistics used are publicly available, with sources listed in Supplementary Table 2. The UK Biobank data source is accessible to all researchers upon application. The instrument datasets used for the TSMR are available via GitHub at https://github.com/KI-PGI.

## Code availability

Code used for this project and the TSMR is available via GitHub at https://github.com/KI-PGI/MDD-MR.
