# Supplementary material for: An encompassing Mendelian randomization study of the causes and consequences of major depressive disorder
Source: Nat Ment Health. 2025 Aug 22;3(9):1002–11. doi: 10.1038/s44220-025-00471-x (PMC12417207; doi:10.1038/s44220-025-00471-x)
Supplement: Supplementary file 1 — Supplementary Information and Tables 1–7. [file 44220_2025_471_MOESM1_ESM.pdf]

nature mental health

Supplementary information

https://doi.org/10.1038/s44220-025-00471-x

An encompassing Mendelian randomization study of the causes and consequences of major depressive disorder

In the format provided by the authors and unedited

Table of Contents

Supplementary Information

1. Methods for GWAS Trait Selection Page 1

2. Methods for Two-Sample MR Follow-Up Analyses Page 1

3. STROBE-MR Checklist Page 3

4. Methods for One-Sample MR Analyses

4.1 Outcome variable definitions Page 8

4.2 Sensitivity analysis using LDpred2 PRS Page 8

4.3 Instrument strength Page 8

4.4 Observational OLS regression methods Page 9

4.5 Per-SNP association testing for sensitivity analyses Page 9

5. Results for One-Sample MR using LDpred2 PRS Instrument Page 9

References Page 11

Supplementary Tables

Table S1: Literature Review of MDD-Associated Traits Page 12

Table S2: GWAS Traits Selected for LDSC and MR Analyses Page 14

Table S3: One-Sample MR Outcome Definitions Page 16

Table S4: Genetic Correlations with MDD (from LDSC) Page 17

Table S5: Two-Sample MR Results: MDD as Exposure Page 20

Table S6: Two-Sample MR Results: MDD as Outcome Page 31

Table S7: One-Sample MR Results Page 34

Supplementary Information

1. Methods for GWAS trait selection

GWAS catalog[1], GWAS ATLAS[2], the fastGWA repository[3], and PubMed were searched for GWAS on the identified traits by the literature review. Psychiatric traits were not included as risk factor or outcome, given the likelihood of comorbidity and pleiotropic associations with MDD. The exception is suicide, which is considered a key outcome in MDD. We manually categorized the identified traits into 18 categories, that are listed alongside their source in Table S2-S3 and are used throughout the results section to aid interpretation.

We selected GWAS for analysis if summary statistics were publicly available and N≥10,000 for continuous traits, or N cases≥5,000 for binary traits, as these sample sizes are generally needed to yield sufficient power in MR analyses[4]. Next, the summary statistics were analyzed with LDscore regression to determine SNP-based heritability. Traits that had a SNP-based heritability that was not significant and/or fell below 3% were excluded. Traits with at least 5 GW-SNPs as reported in the source GWAS were selected. The N, heritability, and number of instruments criteria serve to ensure power for the MR analyses and are based on observations and recommendations from seminal MR papers, e.g. [5–7]. In addition, we focused on European ancestry GWAS because of the limited availability of other-ancestry GWAS. If there were only trans ancestry summary statistics available for a trait, they were still included if the majority of the sample was European. Because of differing allele frequencies in different populations, results relying on mixed ancestry GWAS need to be interpreted with caution (Table S2). Because most of the considered traits are binary, effect sizes are comparable in the exposure and outcome GWASs. Wherever this is not the case (or wherever there are substantial differences in the operationalization of the binary traits), GWAS betas cannot be interpreted in the same manner. Thus, MR estimates are not directly compared between trait pairs.

2. Methods for Two-Sample MR follow-up analyses

Causal interpretation of MR findings is supported if the analyses meet 3 core assumptions, including relevance (the genetic instruments reliably capture the exposure), independence (the genetic instruments are not related to the outcome through an unmeasured confounder), and exclusion restriction (the instruments are only related to the outcome through their association with the exposure). The first assumption is met through selecting variants with a strong association in GWAS with sufficient power (based on SNP-heritability and sample size). A wide range of sensitivity analyses is employed to fulfill the other assumptions, mostly boiling down to various ways of dealing with pleiotropy. These methods combined should account for those sources of pleiotropy that can be measured, although few methods are suited to detect and adjust for pleiotropy stemming from other, unmeasurable sources (e.g., assortative mating or dynastic effects).

As a first sensitivity check, we performed Steiger filtering of the standard IVW estimate and excluded instrument SNPs that explained more variance in the outcome than in the exposure at $p<.05$ to counter horizontal pleiotropic effects. Second, to check for pleiotropy, we estimated the MR Egger intercept, and flagged results that were significantly pleiotropic according to this parameter. The Q-

heterogeneity statistic was reported to indicate if the effect was uniform across the instrument SNPs, or driven by only some of them (which can also be an indication of pleiotropy). To assess the threat of weak instrument bias we reported the *F*-statistic and excluded instruments with an $F<10$. We also reported weighted median and mode results, that are more robust to weak or pleiotropic instruments.

As an alternative TSMR method, we re-estimated the effects using Generalised Summary-data-based Mendelian Randomisation (GSMR) as implemented in the Genome-wide Complex Trait Analysis (GCTA) software package[8]. GSMR is a powerful adaptation of the TSMR method that models the linkage disequilibrium structure between SNPs to allow for correlated instrumental SNPs (avoiding the power loss associated with excluding many instruments). We applied HEIDI outlier filtering in the GSMR analysis, which removes pleiotropic instruments in a manner equivalent to Steiger filtering. Furthermore, we repeated the analyses in Latent Heritable Confounder MR (LHC-MR)[9]. This method aims to adjust for unmeasured confounders by estimating latent heritable factors. As such, it should also be able to adjust for vertical pleiotropic effects. Crucial for our purposes, it is robust to sample overlap, which was unavoidable in this study due to the wide range of selected traits. As a trade-off, the LHC-MR effect estimates seem to be less precise (i.e., have wider confidence intervals). Finally, we performed MR-PRESSO analyses using the MRPRESSO R-package. MR-PRESSO is a tool to correct for pleiotropic outliers relying on leave-one-out analysis, thereby retaining more statistical power than MR-Egger[10.]

3. STROBE-MR checklist of recommended items to address in reports of Mendelian randomization studies[1 2]

Item No.	Section		Checklist item	Page No.	Relevant text from manuscript
1	**TITLE and ABSTRACT**		Indicate Mendelian randomization (MR) as the study's design in the title and/or the abstract if that is a main purpose of the study	1	Title, methods section abstract
	**INTRODUCTION**				
2	**Background**		Explain the scientific background and rationale for the reported study. What is the exposure? Is a potential causal relationship between exposure and outcome plausible? Justify why MR is a helpful method to address the study question	2, 6, Table S1	Brief overview of risk factors and outcomes in introduction; comprehensive literature review in results section
3	**Objectives**		State specific objectives clearly, including pre-specified causal hypotheses (if any). State that MR is a method that, under specific assumptions, intends to estimate causal effects	2	Last paragraph introduction
	**METHODS**				
4	**Study design and data sources**		Present key elements of the study design early in the article. Consider including a table listing sources of data for all phases of the study. For each data source contributing to the analysis, describe the following:		*Due to the scale of this study, it was not feasible to offer a high level of detail for all aspects listed here. Key study features have been reported in Table S2.*
		a)	Setting: Describe the study design and the underlying population, if possible. Describe the setting, locations, and relevant dates, including periods of recruitment, exposure, follow-up, and data collection, when available.	NA	NA
		b)	Participants: Give the eligibility criteria, and the sources and methods of selection of participants. Report the sample size, and whether any power or sample size calculations were carried out prior to the main analysis	3	Studies had to meet strict inclusion criteria for instrument selection to ensure power
		c)	Describe measurement, quality control and selection of genetic variants	3-4	Automated selection and QC procedures were applied as implemented in the TSMR package
		d)	For each exposure, outcome, and other relevant variables, describe methods of assessment and diagnostic criteria for diseases	NA	NA
		e)	Provide details of ethics committee approval and participant informed consent, if relevant	NA	Studies have all been conducted under relevant ethical approval.
5	**Assumptions**		Explicitly state the three core IV assumptions for the main analysis (relevance, independence and exclusion restriction) as well assumptions for any additional or sensitivity analysis	Sup Info 2	Assumptions and sensitivity analyses conducted to address them have been described

6	**Statistical methods: main analysis**		Describe statistical methods and statistics used		
		a)	Describe how quantitative variables were handled in the analyses (i.e., scale, units, model)	NA	Due to the scale of the study, this has not been described for all traits, though care was taken to not directly compare effect sizes between traits (Sup Info 1)
		b)	Describe how genetic variants were handled in the analyses and, if applicable, how their weights were selected	3-4	Described under TSMR methods
		c)	Describe the MR estimator (e.g. two-stage least squares, Wald ratio) and related statistics. Detail the included covariates and, in case of two-sample MR, whether the same covariate set was used for adjustment in the two samples	4	For TSMR, the covariate set in both samples has not been detailed, though almost all GWASs have controlled for age/ birth year, sex, and ancestry
		d)	Explain how missing data were addressed	Sup Info 3.1	Only relevant for OSMR analysis
		e)	If applicable, indicate how multiple testing was addressed	3,5	Bonferroni for LDSC, FDR for MR analyses
7	**Assessment of assumptions**		Describe any methods or prior knowledge used to assess the assumptions or justify their validity	Sup Info 2	
8	**Sensitivity analyses and additional analyses**		Describe any sensitivity analyses or additional analyses performed (e.g. comparison of effect estimates from different approaches, independent replication, bias analytic techniques, validation of instruments, simulations)	Sup Info 2	
9	**Software and pre-registration**				
		a)	Name statistical software and package(s), including version and settings used	Sup Info 2	
		b)	State whether the study protocol and details were pre-registered (as well as when and where)	3	Under 'Trait selection' in Methods section
	**RESULTS**				
10	**Descriptive data**				
		a)	Report the numbers of individuals at each stage of included studies and reasons for exclusion. Consider use of a flow diagram	Table S2, S6-7	
		b)	Report summary statistics for phenotypic exposure(s), outcome(s), and other relevant variables (e.g. means, SDs, proportions)	Table S2	Due to the scope of the study, we limit ourselves to reporting SNP-based heritability per GWAS

		c)	If the data sources include meta-analyses of previous studies, provide the assessments of heterogeneity across these studies	NA	Heterogeneity reports can be found in the source GWASs, referenced in Table S2
		d)	For two-sample MR:<br>i. Provide justification of the similarity of the genetic variant-exposure associations between the exposure and outcome samples<br>ii. Provide information on the number of individuals who overlap between the exposure and outcome studies	Sup Info 1-2	i. Explained in Sup Info 1<br>ii. To limit sample overlap, we excluded UK-Biobank. In addition, we performed LHC-MR which can deal with sample overlap (Sup Info 2)
11	**Main results**				
		a)	Report the associations between genetic variant and exposure, and between genetic variant and outcome, preferably on an interpretable scale	NA	Source GWAS are referenced in Sup Table S2
		b)	Report MR estimates of the relationship between exposure and outcome, and the measures of uncertainty from the MR analysis, on an interpretable scale, such as odds ratio or relative risk per SD difference	Table S5-6	Because of the range of considered traits, we have not converted estimates to interpretable scales. Estimates are accompanied by standard errors and a range of sensitivity estimates and robustness parameters instead
		c)	If relevant, consider translating estimates of relative risk into absolute risk for a meaningful time period	NA	NA
		d)	Consider plots to visualize results (e.g. forest plot, scatterplot of associations between genetic variants and outcome versus between genetic variants and exposure)	Fig 3-4	
12	**Assessment of assumptions**				
		a)	Report the assessment of the validity of the assumptions	8,9,11, Table S5-6, Fig 3-4	Reports of sensitivity and robustness checks aimed at checking assumptions are integrated in results text, figures, and tables
		b)	Report any additional statistics (e.g., assessments of heterogeneity across genetic variants, such as I^2, Q statistic or E-value)	Fig 3-4, Table S5-6	Power estimates, Q, I^2, etc. are reported in results figures and tables
13	**Sensitivity analyses and additional analyses**				
		a)	Report any sensitivity analyses to assess the robustness of the main results to violations of the assumptions	8,9,11, Table S5-6,	Sensitivity analyses are integrated in results text, figures, and tables

				Fig 3-4, Sup figures	
		b)	Report results from other sensitivity analyses or additional analyses	8,9,11, Table S5-6, Fig 3-4, Sup figures	Sensitivity analyses are integrated in results text, figures, and tables
		c)	Report any assessment of direction of causal relationship (e.g., bidirectional MR)	8,9, Table S5, Fig 3	TSMR analyses have been conducted bidirectionally
		d)	When relevant, report and compare with estimates from non-MR analyses	Fig 4	Observational regression analyses are reported for OSMR
		e)	Consider additional plots to visualize results (e.g., leave-one-out analyses)	Sup figures	Broader overview of sensitivity analyses are presented in Fig S1-6
	**DISCUSSION**				
14	**Key results**		Summarize key results with reference to study objectives	13	
15	**Limitations**		Discuss limitations of the study, taking into account the validity of the IV assumptions, other sources of potential bias, and imprecision. Discuss both direction and magnitude of any potential bias and any efforts to address them	14	
16	**Interpretation**				
		a)	Meaning: Give a cautious overall interpretation of results in the context of their limitations and in comparison with other studies	13-14	
		b)	Mechanism: Discuss underlying biological mechanisms that could drive a potential causal relationship between the investigated exposure and the outcome, and whether the gene-environment equivalence assumption is reasonable. Use causal language carefully, clarifying that IV estimates may provide causal effects only under certain assumptions	13-14	
		c)	Clinical relevance: Discuss whether the results have clinical or public policy relevance, and to what extent they inform effect sizes of possible interventions	14	
17	**Generalizability**		Discuss the generalizability of the study results (a) to other populations, (b) across other exposure periods/timings, and (c) across other levels of exposure	14	

	**OTHER INFORMATION**		
18	**Funding**	Describe sources of funding and the role of funders in the present study and, if applicable, sources of funding for the databases and original study or studies on which the present study is based	15
19	**Data and data sharing**	Provide the data used to perform all analyses or report where and how the data can be accessed, and reference these sources in the article. Provide the statistical code needed to reproduce the results in the article, or report whether the code is publicly accessible and if so, where	15
20	**Conflicts of Interest**	All authors should declare all potential conflicts of interest	

This checklist is copyrighted by the Equator Network under the Creative Commons Attribution 3.0 Unported (CC BY 3.0) license.

1. Skrivankova VW, Richmond RC, Woolf BAR, Yarmolinsky J, Davies NM, Swanson SA, et al. Strengthening the Reporting of Observational Studies in Epidemiology using Mendelian Randomization (STROBE-MR) Statement. JAMA. 2021;under review.

2. Skrivankova VW, Richmond RC, Woolf BAR, Davies NM, Swanson SA, VanderWeele TJ, et al. Strengthening the Reporting of Observational Studies in Epidemiology using Mendelian Randomisation (STROBE-MR): Explanation and Elaboration. BMJ. 2021;375:n2233.

4. Methods for One-Sample MR analyses

4.1. Outcome variable definitions

For the OSMR analyses, we selected traits that were not captured with GWAS but were measured in the UK-Biobank. For disease traits (including MDD) we relied on hospital inpatient records (datafield 41270), that were available in maximum N=10,121 individuals for whom MDD case status could be derived. All diagnoses were coded according to International Statistical Classification of Diseases 10th edition. The codes used to define outcomes are summarized in Table S3. For all diagnoses, we looked at lifetime prevalence, because prevalence rates were too low to distinguish between a diagnosis before or after MDD onset while retaining statistical power, and because the self-report traits were also measured without taking into account MDD onset. MDD was defined as having a lifetime diagnosis coded F32 (depressive episode) or F33 (recurrent depression). All individuals without the target diagnosis are used as controls. The advantage of relying on electronic health records (EHR) for the disease outcomes is that we could use a similar, real-world measures to capture a range of outcomes, without having to rely on self-report and potentially arbitrary thresholds for diagnosing any condition. The potential drawback is that not all cases may have an EHR record, and some may end up in the control group. Still, given the seriousness of the considered outcomes, most cases should still be captured in EHR.

The self-report traits were cleaned following the UK-Biobank coding scheme. Continuous variables were winsorized, such that values more than 4 standard deviations from the average were set to the maximum (for positive outliers) or minimum value (for negative outliers), so that the outliers could not distort the results, but were not ignored either. The continuous variables were furthermore standardized to ensure comparability across outcomes. Ordinal (non-binary) outcomes were treated as continuous variables in the analysis.

4.2. Sensitivity analysis with LDpred2 PRS

Because our PRS instrument suffered from weak instrument bias, we repeated the analyses using a PRS created with LDpred2. LDpred2 by default includes information from many more SNPs, making this instrument more likely to be pleiotropic, even if it is more powerful. We preformed LDpred2 on automatic mode, using the SNPs from the European ancestry HapMap3 reference panel. Missing genotypes were imputed using the mean value from the reference data. The prior for the proportion of causal variants that were predicted to contribute to the phenotype was set at 0.2, which is the value suggested by the LDpred2 authors. Repeating procedures for the classic PRS, the LDpred2 PRS was standardized, and sex, birth year (log transformed), and the standardized first 10 principal components for genetic ancestry were regressed out.

4.3. Instrument strength

Next, we estimated R^2 (variance explained in observed MDD by the instrument PRS) and *F*, which reflects instrument strength[4]. For *F*, we used a formula taking into account the sample size *N* and the number of instrument SNPs *k*:

$$F = \frac{R^2(N-1-k)}{(1-R^2)k}$$

Our main results relied on the standard Plink PRS, for which k was the number of independent instrument SNPs for MDD as used in TSMR (k=150). For the sensitivity analyses using the LDpred2 PRS, the number of independent instrument SNPs k cannot be derived, since LDpred2 does not use clumping or p-thresholding in the traditional sense. To approach k, we counted the clumped genome-wide significant hits at p<5E-8, R^2=0.01, and distance <1,000 kb using the clumping function from the TwoSampleMR package, relying on European ancestry reference data from 1000 Genomes. This resulted in k=203.

4.4. Methods for observational OLS regression analysis

To facilitate comparison of the 2SLS results with observational associations, we performed regression analyses on the observed data, again regressing each outcome on MDD, while controlling for sex and birth year. For continuous outcomes, we used linear regression. For binary outcomes, we used logistic regression.

4.5. Per-SNP association tests for sensitivity follow-up analyses

To be able to use TSMR methods for the sensitivity analyses (including IVW, MR Egger, and weighted median and mode) association analyses needed to be performed between each SNP in the PRS and each outcome. For these analyses we used Plink --linear for continuous outcomes and --logistic for binary outcomes while controlling for sex, birth year, and the first 10 principal components capturing population stratification (as provided by UK-Biobank). Because the number of included SNPs was limited and the number of estimated parameters was high, association staetistics could not be derived for 4 (out of 48) traits. For the remainder, sensitivity analyses could be performed relying on the same methods as used for TSMR, although the number of SNPs was insufficient to estimate the weighted median for 22 outcomes.

5. Results for One-Sample MR using the LDpred2 PRS instrument

We repeated the OSMR using a putatively more powerful SNP instrument created using LDpred2. Overall, results were similar to that from the classic PRS, but confidence intervals were more narrow. The strongest negative effects were observed for age at completion of education, subjective health rating, accidents, and the strongest positive effects for pain, problems with usual activities, and health dissatisfaction. Interestingly, some of the small effects that were not significant in the OLS were significant in the OSMR using the LDPred2-based instrument (i.e., magnesium intake, use of the contraceptive pill, pregnancy terminations, birth weight of first child, war exposure, and accidents), suggesting that there may be small causal effects that OLS cannot detect due to confounding. The PRS explained high amounts of variance in MDD (Nagelkerke's R^2=7.1%, which is almost the complete SNP-heritability), and there was no weak instrument bias (average p=3.7E-176). The Wu-Hausman again indicated that OSMR estimates were preferrable in most analyses that showed a significant association (average p=.06). Assuming (conservatively) that the LDpred2 PRS relied on approximately k=203 SNP effects, the instrument strength was estimated at F=32.0, and there was hardly evidence for pleiotropy (only celiac disease again showed a significant MR-Egger intercept). Thus, using the LDpred2-based PRS resulted in better analytical power and did not detectably increase pleiotropy. Conceptually, more pleiotropy must have been introduced due to LDpred2's reliance on the entire

genome in estimating the signal. Methodological follow-up research is warranted to establish if increasing the amount of signal captured by the PRS is a viable approach to increasing the power in OSMR analyses, while avoiding increases in pleiotropy.

References

1 Buniello A, MacArthur JAL, Cerezo M, *et al.* The NHGRI-EBI GWAS Catalog of published genome-wide association studies, targeted arrays and summary statistics 2019. 2019; **47**: D1005–12.

2 Watanabe K, Stringer S, Frei O, *et al.* A global overview of pleiotropy and genetic architecture in complex traits. *Nat Genet* 2019; **51**: 1339–48.

3 Jiang L, Zheng Z, Fang H, Yang J. A generalized linear mixed model association tool for biobank-scale data. *Nat Genet* 2021; **53**: 1616–21.

4 Pierce BL, Ahsan H, VanderWeele TJ. Power and instrument strength requirements for Mendelian randomization studies using multiple genetic variants. *Int J Epidemiol* 2011; **40**: 740–52.

5 Burgess S, Davey Smith G, Davies NM, *et al.* Guidelines for performing Mendelian randomization investigations: update for summer 2023. *Wellcome Open Res* 2019; **4**: 186.

6 Brion M-JA, Shakhbazov K, Visscher PM. Calculating statistical power in Mendelian randomization studies. *Int J Epidemiol* 2013; **42**: 1497–501.

7 Burgess S. Sample size and power calculations in Mendelian randomization with a single instrumental variable and a binary outcome. *Int J Epidemiol* 2014; **43**: 922–9.

8 Zhu Z, Zheng Z, Zhang F, *et al.* Causal associations between risk factors and common diseases inferred from GWAS summary data. *Nat Commun* 2018; **9**: 224.

9 Darrous L, Mounier N, Kutalik Z. Simultaneous estimation of bi-directional causal effects and heritable confounding from GWAS summary statistics. 2020. DOI:10.1101/2020.01.27.20018929.

10 Verbanck M, Chen C-Y, Neale B, Do R. Detection of widespread horizontal pleiotropy in causal relationships inferred from Mendelian randomization between complex traits and diseases. *Nat Genet* 2018; **50**: 693–8.

Table S1. Literature review of traits associated with MDD (manually categorised), framed as exposure or outcome, with effect size (if reported). To the right corresponding GWAS sources are listed.

PMID	Trait	Parent trait	Captured in GWAS	MDD as exposure or outcome	Type effect size	Reported effect size	GWAS trait	GWAS PMID
28838903	Hiv Infection	Disease: infectious	1	outcome	NA	NA	Hiv Infection	20041166
25772898	Multiple Sclerosis	Disease: neurological	1	outcome	RR	3	Multiple Sclerosis	21833088
27629598	Psychomotor Performance	Cognition	1	exposure	NA	NA	Psychomotor Performance	22054870
22128768	Celiac Disease	Disease: gastrointestinal	1	outcome	Cohen	0.1	Celiac Disease	22057235
32348643	Pancreatic Cancer	Disease: cancer	1	both	NA	NA	Pancreatic Cancer	22523087
27629598	Cytokine	Protein: inflammation	1	both	NA	NA	Cytokine	22610502
29886003	Psoriasis	Disease: psoriasis	1	outcome	OR	1.64	Psoriasis	23143594
28786792	Mycobacterium Infection	Disease: infectious	1	outcome	RR	2.63	Mycobacterium Infection	25642632
24996130	Systemic Lupus Erythematosus	Disease: muscoloskeletal	1	outcome	HR	1.17	Systemic Lupus Erythematosus	26502338
26773921	Parental Longevity	Mortality	1	exposure	OR	2.09	Parental Longevity	27015805
22717127	Birth Weight	Birth weight	1	outcome	OR	1.15	Birth Weight	27680694
28949960	Age First Birth	Reproduction	1	outcome	prevalence	0.60%	Age First Birth	27798627
17401034	Social Deprivation	Socioeconomic status	1	outcome	OR	1.11	Social Deprivation	27818178
34860728	Self-Reported Health	Disease	1	both	NA	NA	Self-Reported Health	27864402
33343240	Il2 Levels	Protein: inflammation	1	both	NA	NA	Il2 Levels	27989323
27629598	Tnf	Protein: inflammation	1	both	NA	NA	Tnf	27989323
32348643	Crohn'S Disease	Disease: gastrointestinal	1	exposure	HR	1.4	Crohn'S Disease	28067908
19161177	Inflammatory Bowel Disease	Disease: gastrointestinal	1	both	NA	NA	Inflammatory Bowel Disease	28067908
25663206	Ulcerative Colitis	Disease: gastrointestinal	1	exposure	OR	2.055	Ulcerative Colitis	28067908
12093945	Lymphoma	Disease: cancer	1	outcome	NA	NA	Lymphoma	28112199
11879160	Nicotine Dependence	Substance use: smoking	1	exposure	OR	2.1	Nicotine Dependence	28440896
33937941	Diverticulitis	Disease: gastrointestinal	1	outcome	HR	1.34	Diverticulitis	28585551
26446894	RMSDD	Physical activity	1	both	r	-0.16	Rmsdd	28613276
29886003	25-Hydroxyvitamin D Level	Diet: vitamine D	1	outcome	OR	1.31	25-Hydroxyvitamin D Level	28757204
29552990	Susceptibility To Rubella	Disease: infectious	1	outcome	NA	0.605	Susceptibility To Rubella	28928442
36515938	Parental Lifespan	Mortality	1	exposure	NA	NA	Parental Lifespan	29227965
17707257	Offspring Birth Weight	Reproduction	1	outcome	NA	NA	Offspring Birth Weight	29309628
33799036	Amyotrophic Lateral Sclerosis	Disease: neurological	1	outcome	NA	NA	Amyotrophic Lateral Sclerosis	29566793
19161177	Irritable Bowel Disease	Disease: gastrointestinal	1	both	NA	NA	Irritable Bowel Disease	29626450
26600410	Health-Related Qol	Health: subjective	1	exposure	NA	NA	Health-Related Qol	29855537
36250518	Atrial Fibrillation	Disease: CVD	1	exposure	NA	NA	Atrial Fibrillation	29892015
30480317	Loneliness	Social: partner	1	outcome	NA	NA	Loneliness	29970889
11315232	Mother's educational attainment	Socioeconomic status	1	outcome	OR	2.855	Mother's EA	29987013
34254060	Allergic Rhinitis	Disease: ear-nose-throat	1	outcome	OR	1.4	Allergic Rhinitis	30013184
24996130	Diastolic Blood Pressure	Disease: CVD	1	both	HR	1.33	Diastolic Blood Pressure	30224653
29886003	BMI	Metabolic: BMI	1	outcome	OR	1.41	BMI	30239722
29886003	Folate Measurement	Disease: dietary deficit	1	outcome	OR	1.21	Folate Measurement	30339177
30480317	Alcohol Dependence	Substance use: alcohol	1	both	RR	3	Alcohol Dependence	30482948
16259539	Sleep Duration	Circadian	1	both	NA	NA	Sleep Duration	30531941
23415826	Sedentary Behavior	Physical activity	1	outcome	NA	NA	Sedentary Behavior	30531941
31974072	Generalized Epilepsy	Disease: neurological	1	exposure	NA	NA	Generalized Epilepsy	30531953
26271451	Meta Autoimmune Disease	Disease: autoimmune	1	exposure	IRR	1.22	Meta Autoimmune Disease	30572963
29886003	Erectile Dysfunction	Sexual disorders	1	outcome	OR	2.71	Erectile Dysfunction	30583798
12893098	Risky Behaviours	Health behavior	1	exposure	NA	NA	Risky Behaviours	30643258
28463712	Chronotype	Circadian	1	both	Cohen	0.2	Chronotype	30696823
33743882	Disease-free survival	Disease	1	exposure	R2	24%	Healthspan	30729179
29886003	Insomnia	Circadian	1	outcome	OR	2.6	Insomnia	30804565
28175262	Multisite Chronic Pain	Disease: muscoloskeletal	1	both	RR	6	Multisite Chronic Pain	31194737
24168753	Cognitive Impairment	Cognition	1	exposure	Cohen	0.495	Cognitive Impairment	31201950
34421672	Amennorhea, Dysmennorhea	Disease: obstretics/ gynaecology	1	outcome	RR	1.72	Amennorhea, Dysmennorhea	31371054
31955278	Job Involves Night Shifts	Circadian	1	outcome	NA	NA	Job Involves Night Shifts	31427789
29886003	Variation In Diet	Diet	1	outcome	RR	0.76	Variation In Diet	31427789
27629598	Number Of Self-Reported Illnesses	Disease	1	exposure	RR	1.60	Number Of Self-Reported Illnesses	31427789
28641155	Adenocarcinoma	Disease: cancer	1	both	OR	1.3	Adenocarcinoma	31427789
34706453	Malignant Neoplasm Of Breast	Disease: cancer	1	both	NA	NA	Malignant Neoplasm Of Breast	31427789
36250518	Malignant Neoplasms Of Skin	Disease: cancer	1	exposure	HR	1.1	Malignant Neoplasms Of Skin	31427789
31974072	Migraine	Disease: neurological	1	outcome	NA	NA	Migraine	31427789
16950996	Asthma	Disease: respiratory	1	exposure	OR	2.59	Asthma	31427789
36058357	Age At Menarche	Endocrine	1	outcome	Cohen	0.26	Age At Menarche	31427789
33715045	Social transfer payment	Functional: employment	1	exposure	NA	NA	Unable To Work	31427789
26458894	Job loss	Functional: employment	1	outcome	RRR	2.505	Unable To Work	31427789
33104205	Unable To Work	Functional: employment	1	exposure	RR	2.02	Unable To Work	31427789
25462417	Medication And Treatment Use	Health care use	1	exposure	NA	NA	Medication And Treatment Use	31427789
12470316	Happiness With Health	Health: subjective	1	outcome	NA	NA	Happiness With Health	31427789
21820466	Physical Activity (Device Measured)	Physical activity	1	outcome	OR	0.46	Physical Activity (Device Measured)	31427789
17895237	Number Of Children	Reproduction	1	exposure	OR	0.66	Number Of Children	31427789
30577941	Number Of Pregnancy Terminations	Reproduction	1	exposure	OR	1.38	Number Of Pregnancy Terminations	31427789
34886815	Number Of Still Births	Reproduction	1	outcome	NA	NA	Number Of Still Births	31427789
9707383	Maternal Smoking	Substance use: smoking	1	outcome	NA	NA	Maternal Smoking	31427789
22694991	Partner violence	Trauma/ stress: adult	1	exposure	RR	3	Illness, Injury, Assault	31427789
23408506	Physical injury	Trauma/ stress: adult	1	outcome	NA	NA	Illness, Injury, Assault	31427789
34672991	Childhood adversity	Trauma/ stress: childhood	1	outcome	OR	3.6125	Illness, Injury, Assault	31427789
24270310	Methamphetamine Dependence	Substance use	1	outcome	NA	NA	Methamphetamine Dependence	31462767
29886003	Preterm Birth	Birth weight	1	outcome	OR	1.08	Preterm Birth	31477735
25892882	Gastroesophageal Reflux Disease	Disease: gastrointestinal	1	outcome	NA	NA	Gastroesophageal Reflux Disease	31527586
29886003	Metabolic Syndrome	Metabolic	1	outcome	OR	1.45	Metabolic Syndrome	31589552
32891927	Attention	Cognition	1	exposure	Cohen	0.59	Attention	31596458
30480317	Household Income	Socioeconomic status	1	outcome	NA	NA	Household Income	31844048
24996130	Heart Failure	Disease: CVD	1	outcome	OR	1.39	Heart Failure	31919418
30786242	Estradiol	Endocrine	1	both	NA	NA	Estradiol	32042192
19625884	Total Testosterone Levels	Endocrine	1	outcome	NA	NA	Total Testosterone Levels	32042192
27629598	Childhood Maltreatment	Trauma/ stress: childhood	1	exposure	NA	NA	Childhood Maltreatment	32066696
25981310	Opioid Dependence	Substance use	1	exposure	OR	2.18	Opioid Dependence	32099098
24274962	Precocious Puberty (Males)	Endocrine	1	outcome	RR	1.3	Precocious Puberty (Males)	32210231
27028090	Lymphocytic Leukemia	Disease: cancer	1	outcome	NA	NA	Lymphocytic Leukemia	32887889
34860728	Suicide Death	Suicide	1	exposure	HR	15	Suicide Death	32998551
29886003	Head Injury	Disease: neurological	1	outcome	OR	3.41	Head Injury	33017352
29886003	Cannabis Dependence	Substance use	1	both	OR	1.43	Cannabis Dependence	33096046
19801582	Chronic Obstructive Pulmonary Disease In Smokers	Disease: respiratory	1	both	IRR	1.72	Chronic Obstructive Pulmonary Disease In Smokers	33106845
29886003	Anti-Varicella Zoster Virus Igg Seropositivity	Disease: infectious	1	outcome	OR	2.1	Anti-Varicella Zoster Virus Igg Seropositivity	33204752
29886003	Epstein Barr Virus Seropositivity	Disease: infectious	1	both	OR	1.98	Epstein Barr Virus Seropositivity	33204752
29886003	Herpes Simplex Virus Seropositivity	Disease: infectious	1	outcome	OR	1.98	Herpes Simplex Virus Seropositivity	33204752
29886003	Toxoplasma Gondii Seropositivity	Disease: infectious	1	outcome	OR	0.38	Toxoplasma Gondii Seropositivity	33204752
29886003	Magnesium Levels	Disease: dietary deficit	1	outcome	RR	1.34	Magnesium Levels	33441150
28165367	Total Cholesterol Levels	Metabolic: cholesterol	1	both	NA	NA	Total Cholesterol Levels	33462484
33343240	Il6 Levels	Protein: inflammation	1	both	NA	NA	Il6 Levels	33517400
28838903	Staphylococcus Aureus Infection	Disease: infectious	1	outcome	NA	NA	Staphylococcus Aureus Infection	33662382
25989342	Low Back Pain	Disease: muscoloskeletal	1	outcome	OR	1.64	Low Back Pain	33729212
30909841	Tinnitus	Disease: ear-nose-throat	1	outcome	NA	NA	Tinnitus	33742053
23647647	Clostridium Difficile Infection	Disease: infectious	1	exposure	OR	1.4	Clostridium Difficile Infection	33841421
11879160	Alcohol Abuse	Substance use: alcohol	1	exposure	OR	1.5	Alcohol Abuse	33985350
35134841	Parkinson Disease	Disease: neurological	1	exposure	HR	1.8	Parkinson Disease	34064523
29886003	Type I Diabetes	Metabolic: diabetes	1	outcome	RR	1.25	Type I Diabetes	34127860
29886003	Cytomegalovirus Infection	Disease: infectious	1	both	OR	1.98	Cytomegalovirus Infection	34269803
23770320	Age At Menopause	Reproduction	1	outcome	OR	3.11	Age At Menopause	34349265
30169168	Antisocial Behavior	Antisocial	1	outcome	NA	NA	Externalizing	34446935
35134841	Alzheimer'S Disease	Disease: neurological	1	both	HR	1.6	Alzheimer'S Disease	34493870
29094073	Chronic Sinusitis	Disease: ear-nose-throat	1	both	OR	1.35	Chronic Sinusitis	34594039
10568627	Seasonal Allergic Rhinitis	Disease: ear-nose-throat	1	outcome	OR	1.58	Seasonal Allergic Rhinitis	34594039
31945502	Anemia	Disease: hematological	1	outcome	OR	1.53	Anemia	34594039
15774953	Hepatitis B	Disease: infectious	1	outcome	NA	NA	Hepatitis B	34594039
17956625	Hepatitis C	Disease: infectious	1	outcome	OR	3.3	Hepatitis C	34594039
36064836	Hyperthyroidism	Endocrine	1	outcome	OR	1.67	Hyperthyroidism	34594039
35134841	Peripheral Artery Disease	Disease: CVD	1	both	HR	1.4	Peripheral Artery Disease	34601942
13680463	Family Relationship Score	Cognition	1	exposure	NA	NA	Family Relationship Score	34737426
29886003	Coffee Consumption	Diet: coffee	1	outcome	RR	0.71	Coffee Consumption	34737426
29886003	Fish Consumption	Diet: fish	1	outcome	RR	0.83	Fish Consumption	34737426
29886003	Fruit Consumption	Diet: fruit	1	outcome	RR	0.85	Fruit Consumption	34737426
29886003	Tea Consumption	Diet: tea	1	outcome	OR	0.68	Tea Consumption	34737426
29886003	Vegetable Consumption	Diet: vegetable	1	outcome	RR	0.87	Vegetable Consumption	34737426
29886003	Zinc Supplements	Diet: zinc	1	outcome	RR	0.65	Zinc Supplements	34737426
23486521	Kidney Failure	Disease: renal	1	outcome	NA	NA	Kidney Failure	34737426
32348643	Adrenocortical Insufficiency	Endocrine	1	both	NA	NA	Adrenocortical Insufficiency	34737426
17401034	Partner In Household	Social: partner	1	outcome	OR	0.6	Partner In Household	34737426
30169168	Separation/ Divorce	Social: partner	1	outcome	NA	NA	Separation/ Divorce	34737426
17401034	Financial Difficulties	Socioeconomic status	1	outcome	beta	0.3	Financial Difficulties	34737426

36250518	Been In Serious Accident	Trauma/ stress: adult	1	outcome	HR	1.63	Been In Serious Accident	34737426
7755111	Death Of Related	Trauma/ stress: adult	1	outcome	OR	1.6	Death Of Related	34737426
26446894	Oxygen Uptake	Physical activity	1	both	r	-0.16	Oxygen Uptake	34753499
28838903	Eczema	Disease: dermatological	1	outcome	OR	4.65	Eczema	34785669
29886003	Polycystic Ovary Syndrome	Disease	1	outcome	OR	4.3	Polycystic Ovary Syndrome	34791234
34796744	Suicidal Behavior	Suicide	1	exposure	HR	3.5	Suicidal Behavior	34861974
11879160	Suicide Attempt	Suicide	1	exposure	OR	2.9	Suicidal Behavior	34861974
28165367	High Density Lipoprotein	Metabolic: cholesterol	1	both	NA	NA	High Density Lipoprotein	34887591
28165367	Low Density Lipoprotein	Metabolic: cholesterol	1	both	NA	NA	Low Density Lipoprotein	34887591
28165367	Triglycerides	Metabolic: triglycerides	1	both	NA	NA	Triglycerides	34887591
33537324	Liver Disease	Disease: gastrointestinal	1	outcome	OR	1.43	Liver Disease	35047847
29886003	Caffeine Intake	Diet: caffeine	1	both	RR	1.35	Caffeine Intake	35050183
28122650	Management Demands	Socioeconomic status	1	outcome	HR	1.43	Management Demands	35286190
26360447	Infant Mortality Rate In Birth Year And County	Socioeconomic status	1	outcome	NA	NA	Infant Mortality Rate In Birth Year And County	35290122
28175262	Educational Attainment	Education	1	both	NA	NA	Educational Attainment	35361970
19188531	Crp Levels	Protein: inflammation	1	both	Cohen	0.15	Crp Levels	35459240
29886003	Statins Response	Disease: CVD	1	outcome	OR	0.68	Statins Response	35543701
29886003	Type Ii Diabetes	Metabolic: diabetes	1	outcome	RR	1.25	Type Ii Diabetes	35551307
29886003	Healthy Food Intake	Diet	1	both	RR	0.76	Healthy Food Intake	35653391
20333505	Pelvic Organ Prolapse	Disease: obstretics/ gynaecology	1	outcome	RR	5	Pelvic Organ Prolapse	35739095
32348643	Coronary Artery Disease	Disease: CVD	1	exposure	HR	1.5	Coronary Artery Disease	35915156
34619491	Severe Covid	Disease: infectious	1	outcome	NA	NA	Severe Covid	35922517
24168753	Verbal Learning, Verbal Short Term Memory	Cognition	1	exposure	Cohen	0.455	Verbal Learning, Verbal Short Term Memory	35974141
28100073	Hiv Susceptibility	Disease: autoimmune	1	outcome	RR	2	Hiv Susceptibility	36034232
23415826	Exercise (Self Report)	Physical activity	1	outcome	Cohen	0.37	Exercise (Self Report)	36071172
34524390	Hypothyroidism	Endocrine	1	outcome	OR	1.3	Hypothyroidism	36093044
22642228	Executive Function	Cognition	1	exposure	Cohen	0.645	Executive Function	36150907
35085232	Stroke	Disease: CVD	1	both	NA	NA	Stroke	36180795
33268766	Chagas Parasite	Disease: infectious	1	outcome	NA	NA	Chagas Parasite	36215317
34012724	Years Ovulating	Endocrine	1	exposure	NA	NA	Years Ovulating	36320039
27680324	Years Taking Contraceptive Pill	Endocrine	1	exposure	RR	1.2	Years Taking Contraceptive Pill	36320039
11879160	Age Last Birth	Reproduction	1	outcome	OR	3.7	Age Last Birth	36320039
16908510	Rheumatoid Arthritis	Disease: muscoloskeletal	1	outcome	RR	2.5	Rheumatoid Arthritis	36333501
30169168	Drinks Per Week	Substance use: alcohol	1	outcome	NA	NA	Drinks Per Week	36477530
29886003	Age At Smoking Initiation	Substance use: smoking	1	outcome	OR	1.68	Age At Smoking Initiation	36477530
29886003	Cigarettes Per Day	Substance use: smoking	1	outcome	OR	1.68	Cigarettes Per Day	36477530
29886003	Smoking Cessation	Substance use: smoking	1	outcome	OR	1.68	Smoking Cessation	36477530
29886003	Smoking Initiation	Substance use: smoking	1	outcome	OR	1.68	Smoking Initiation	36477530

Table S2. GWAS trait selection for LDSC and MR analyses, based on accessibility, sample size, SNP-heritability (h2, derived using LD-score regression), and the number of genome-wide significant SNPs reported in the GWAS publication. For binary traits, the liability-converted SNP-heritability was used; for continuous traits the liability-converted heritability column (liability_h2) has the same value as the non-converted heritability (scale_h2)

GWAS trait	Abbreviation	GWAS PMID	Category	Accessible	Adequate N	Adequate h2	Adequate hits	Reported hits	N	N_CAS	N_CON	Ancestry	liability_h2	liability_SE	scale_h2	scale_SE
Birth weight	birthweight	31097437	Birth weight	0	NA	NA	NA	NA	NA	NA	NA	NA	NA	NA	NA	NA
Preterm birth	gestationduration	31477735	Birth weight	1	1	1	0	4	84689	NA	NA	EUR	0.0742	0.0082	0.0742	0.0082
Chronotype	chronotype	30696823	Circadian	1	1	1	1	440	449734	NA	NA	EUR	0.1108	0.0038	0.1108	0.0038
Insomnia	insomnia	30804565	Circadian	1	1	1	1	364	386988	109548	277440	EUR	0.046	0.0021	0.046	0.0021
Job involves night shifts	nightshifts	31427789[a]	Circadian	1	1	0	0	3	220546	NA	NA	EUR	0.0241	0.0028	0.0241	0.0028
Sleep duration	sleepduration	30531941	Circadian	1	1	1	1	27	91105	NA	NA	EUR	0.1321	0.0075	0.1321	0.0075
Attention	attention	31596458	Cognition	0	NA	NA	NA	NA	NA	NA	NA	NA	NA	NA	NA	NA
Cognitive impairment	cogimpair	31201950	Cognition	0	NA	NA	NA	NA	NA	NA	NA	NA	NA	NA	NA	NA
Educational attainment	education	35361970	Cognition	1	1	1	1	3952	3037504	NA	NA	EUR	0.1565	0.0042	0.1565	0.0042
Executive function	executivefun	36150907	Cognition	1	1	1	1	129	427037	NA	NA	EUR	0.093	0.0042	0.093	0.0042
Fluid intelligence	fluidintelligence	34737426[b]	Cognition	1	1	1	1	10	113084	63006	50078	EUR	0.0537	0.008	0.034	0.005
Psychomotor performance	psychomotor	22054870	Cognition	0	NA	NA	NA	NA	NA	NA	NA	NA	NA	NA	NA	NA
Verbal learning	cogverbal	35974141	Cognition	0	NA	NA	NA	NA	NA	NA	NA	NA	NA	NA	NA	NA
25-Hydroxyvitamin D level	vitamind	28757204	Diet	1	1	1	1	16	42274	NA	NA	EUR	0.0445	0.0145	0.0445	0.0145
Anemia	anemia	34594039	Diet	1	1	1	0	4	480941	12317	468624	EUR/ EAS	0.0424	0.0089	0.006	0.0013
Caffeine levels	caffeine	35050183	Diet	0	NA	NA	NA	NA	NA	NA	NA	NA	NA	NA	NA	NA
Coffee consumption	coffee	34737426[b]	Diet	1	1	1	1	7	64001	45146	18855	EUR	0.0611	0.0134	0.0349	0.0077
Fish consumption	fish	34737426[b]	Diet	1	1	0	1	7	64001	18431	45570	EUR	0.0139	0.0133	0.0079	0.0075
Folate measurement	folate	30339177	Diet	0	NA	NA	NA	NA	NA	NA	NA	NA	NA	NA	NA	NA
Fruit consumption	fruit	34737426[b]	Diet	1	1	1	0	4	64001	52183	11818	EUR	0.0674	0.0148	0.0318	0.007
Healthy food intake	diethealthy	35653391	Diet	0	NA	NA	NA	NA	NA	NA	NA	NA	NA	NA	NA	NA
Magnesium levels	magnesium	33441150	Diet	1	1	1	0	0	20707	NA	NA	EUR	0.0608	0.0242	0.0608	0.0242
Tea consumption	tea	34737426[b]	Diet	1	1	1	1	5	64001	50994	13007	EUR	0.0927	0.015	0.0458	0.0074
Variation in diet	dietvariation	31427789[a]	Diet	1	1	1	1	12	384869	NA	NA	EUR	0.042	0.0022	0.042	0.0022
Vegetable consumption	vegetable	34737426[b]	Diet	1	1	1	1	7	64001	52696	11305	EUR	0.0436	0.0165	0.0201	0.0076
Zinc supplements	zinc	34737426[b]	Diet	1	1	1	1	7	454825	18478	436347	EUR	0.0588	0.0057	0.0115	0.0011
Adenocarcinoma	adenocarcinoma	31427789[a]	Disease	1	1	1	1	12	60655	12480	48175	EUR	0.0637	0.0201	0.0316	0.01
Allergic rhinitis	allergicrhin	30013184	Disease	0	NA	NA	NA	NA	NA	NA	NA	NA	NA	NA	NA	NA
Alzheimer's disease	alzheimer	34493870	Disease	1	1	1	1	38	398058	39918	358140	EUR	0.0456	0.0107	0.0456	0.0107
Amennorhea, dysmennorhea	amenorrhea	31371054	Disease	0	NA	NA	NA	NA	NA	NA	NA	NA	NA	NA	NA	NA
Amyotrophic lateral sclerosis	als	29566793	Disease	1	1	1	1	10	80610	20806	59804	EUR	0.0537	0.0116	0.0293	0.0063
Anti-varicella zoster virus	varicella	33204752	Disease	1	1	0	1	1	8735	7595	1140	EUR	-0.0144	0.0493	-0.0144	0.0493
Asthma	asthma	31427789[a]	Disease	1	1	1	1	18	97640	12362	85278	EUR	0.1707	0.0216	0.0666	0.0084
Atrial Fibrillation	atrialfib	29892015	Disease	1	1	1	1	228	537409	55114	482295	cross	0.0962	0.0075	0.0239	0.0019
Celiac disease	celiac	22057235	Disease	1	1	NA	1	43	24269	12041	12228	NA	NA	NA	NA	NA
Chagas parasite	chagas	36215317	Disease	1	0	NA	NA	NA	NA	NA	NA	NA	NA	NA	NA	NA
Chronic obstructive pulmonary dis	copd	33106845	Disease	1	1	1	1	32	129175	8631	120544	EUR	0.2277	0.0219	0.0613	0.0059
Chronic sinusitis	sinusitis	34594039	Disease	1	1	0	1	6	452495	7529	444966	EUR/ EAS	0.0293	0.0096	0.0031	0.001
Clostridium difficile infection	clostridium	33841421	Disease	0	NA	NA	NA	NA	NA	NA	NA	NA	NA	NA	NA	NA
Coronary artery disease	coronaryartdis	35915156	Disease	1	1	1	1	33	773268	190493	582775	EUR	0.0767	0.0049	0.0333	0.0021
Crohn's disease	crohndis	28067908	Disease	1	1	1	1	119	40266	12194	28072	EUR	0.3698	0.0335	0.3698	0.0335
Cytomegalovirus infection	cytomegalovirus	34269803	Disease	0	NA	NA	NA	NA	NA	NA	NA	NA	NA	NA	NA	NA
Diastolic blood pressure	diabloodpres	30224653	Disease	1	1	1	1	91	757601	NA	NA	EUR	0.1091	0.0044	0.1091	0.0044
Diverticulitis	diverticulitis	28585551	Disease	0	NA	NA	NA	NA	NA	NA	NA	NA	NA	NA	NA	NA
Eczema	eczema	34454985	Disease	1	1	0	1	30	796661	22474	774187	EUR	0.0051	0.001	0.0051	0.001
Epstein Barr virus	epsteinbarr	28654678	Disease	0	NA	NA	NA	NA	NA	NA	NA	NA	NA	NA	NA	NA
Gastroesophageal reflux disease	gerd	31527586	Disease	1	1	1	1	25	332601	71522	261079	EUR	0.0679	0.0024	0.0679	0.0024
Generalized epilepsy	epilepsy	30531953	Disease	1	1	1	1	16	44889	15212	29677	EUR	0.6012	0.045	0.6012	0.045
Head injury	headinjury	33017352	Disease	1	0	NA	NA	NA	NA	NA	NA	NA	NA	NA	NA	NA
Heart failure	heartfail	31919418	Disease	1	1	1	1	12	977323	47309	930014	EUR	0.037	0.003	0.0081	7e-04
Hepatitis B	hepatitisB	34594039	Disease	1	0	NA	NA	NA	NA	NA	NA	NA	NA	NA	NA	NA
Hepatitis C	hepatitisC	34594039	Disease	1	0	NA	NA	NA	NA	NA	NA	NA	NA	NA	NA	NA
Herpes simplex virus	herpes	33204752	Disease	1	1	0	0	2	8735	6199	2536	EUR	0.0184	0.0497	0.0184	0.0497
HIV infection	hivinfec	20041166	Disease	0	NA	NA	NA	NA	NA	NA	NA	NA	NA	NA	NA	NA
HIV susceptibility	hivsuscep	36034232	Disease	1	1	1	1	5	594646	7303	587343	EUR	0.0727	0.0112	0.0061	9e-04
Inflammatory bowel disease	ibd	28067908	Disease	1	1	1	1	135	59957	25042	34915	EUR	0.3112	0.0261	0.3112	0.0261
Irritable bowel disease	irbd	29626450	Disease	1	1	1	1	15	346075	9576	336499	EUR	0.0527	0.0099	0.0079	0.0015
Kidney failure	kidneyfail	34737426	Disease	1	0	NA	NA	NA	NA	NA	NA	NA	NA	NA	NA	NA
Low back pain	backpain	33729212	Disease	0	NA	NA	NA	NA	NA	NA	NA	NA	NA	NA	NA	NA
Lymphocytic leukemia	leukemia	32887889	Disease	1	0	NA	NA	NA	NA	NA	NA	NA	NA	NA	NA	NA
Lymphoma	hodgkin	28112199	Disease	0	NA	NA	NA	NA	NA	NA	NA	NA	NA	NA	NA	NA
Malignant neoplasm of breast	cancerbreast	31427789[a]	Disease	1	1	1	1	11	54778	10420	44358	EUR	0.0946	0.0223	0.0453	0.0107
Malignant neoplasms of skin	cancerskin	31427789[a]	Disease	1	1	1	1	32	54696	15032	39664	EUR	0.2419	0.0322	0.135	0.018
Meta autoimmune disease	autoimmune	30572963	Disease	0	NA	NA	NA	NA	NA	NA	NA	NA	NA	NA	NA	NA
Migraine	migraine	31427789[a]	Disease	1	1	0	1	14	289307	11381	277926	EUR	0.0293	0.0017	0.0293	0.0017
Multiple sclerosis	ms	31604244	Disease	1	1	1	1	200	41505	14802	26703	EUR	0.2853	0.023	0.2853	0.023
Multisite chronic pain	chronicpain	31194737	Disease	1	1	1	1	39	387649	NA	NA	EUR	0.0738	0.0026	0.0738	0.0026
Mycobacterium infection	mycobacterium	25642632	Disease	0	NA	NA	NA	NA	NA	NA	NA	NA	NA	NA	NA	NA
Non-alcoholic fatty liver disease	liverdis	35047847	Disease	0	NA	NA	NA	NA	NA	NA	NA	NA	NA	NA	NA	NA
Pancreatic cancer	cancerpanc	22523087	Disease	1	0	NA	NA	NA	7785	3851	3934	NA	NA	NA	NA	NA
Parkinson disease	parkinson	25064009	Disease	1	1	1	1	26	42783	9538	33245	EUR	0.102	0.0167	0.102	0.0167
Pelvic organ prolapse	pelvicorganprol	35739095	Disease	1	1	1	1	30	574377	28086	546291	EUR	0.081	0.006	0.0179	0.0013
Peripheral artery disease	peripheralartdis	34601942	Disease	1	1	1	1	19	461634	12086	449548	EUR	0.0638	0.0086	0.0092	0.0012
Polycystic ovary syndrome	pcos	34791234	Disease	1	0	NA	NA	NA	NA	NA	NA	NA	NA	NA	NA	NA
Psoriasis	psoriasis	23143594	Disease	1	1	NA	1	41	33394	10588	22806	NA	NA	NA	NA	NA
Rheumatoid arthritis	rheuma	36333501	Disease	1	1	1	1	91	97173	22350	74823	EUR	0.2333	0.0183	0.1214	0.0095
Rubella	rubella	28928442	Disease	0	NA	NA	NA	NA	NA	NA	NA	NA	NA	NA	NA	NA
Seasonal allergic rhinitis	seasallergicrhin	34594039	Disease	1	0	NA	NA	NA	NA	NA	NA	NA	NA	NA	NA	NA
Self-reported illnesses	illnesses	31427789[a]	Disease	1	1	1	1	42	386581	NA	NA	EUR	0.0702	0.0026	0.0702	0.0026
Severe covid 19	covid	35922517	Disease	1	1	1	1	23	1086211	13769	1072442	cross	0.0794	0.0151	0.0068	0.0013
Staphylococcus aureus	staphylococcus	33662382	Disease	1	1	1	1	5	405346	6107	399239	EUR	0.0576	0.0134	0.0056	0.0013
Statins response	statins	35543701	Disease	0	NA	NA	NA	NA	NA	NA	NA	NA	NA	NA	NA	NA
Stroke	stroke	36180795	Disease	1	1	0	1	35	1308460	73652	1234808	EUR	0.0077	6e-04	0.0077	6e-04
Systemic lupus erythematosus	lupus	26502338	Disease	1	1	1	1	47	14267	5201	9066	EUR	0.8454	0.0925	0.5152	0.0564
Systolic blood pressure	sysbloodpres	30224653	Disease	1	1	1	1	130	757601	NA	NA	EUR	0.116	0.0043	0.116	0.0043
Tinnitus	tinnitus	33742053	Disease	1	1	1	0	1	126851	NA	NA	EUR	0.0633	0.0049	0.0633	0.0049
Toxoplasma gondii seropositivity	toxoplasma	33204752	Disease	1	0	NA	0	0	8735	2449	6286	NA	NA	NA	NA	NA
Ulcerative colitis	ulcercolit	28067908	Disease	1	1	1	1	80	40438	12366	28072	EUR	0.2662	0.0264	0.2662	0.0264
Adrenocortical insufficiency	adrenocort	34737426	Endocrine	1	0	NA	NA	NA	NA	NA	NA	NA	NA	NA	NA	NA
Age at menarche	menarche	31427789[a]	Endocrine	1	1	1	1	215	202636	NA	NA	EUR	0.2342	0.0098	0.2342	0.0098
Contraceptive pill	contrapill	36320039	Endocrine	1	1	1	0	0	178233	NA	NA	EUR	0.0301	0.0031	0.0301	0.0031
Estradiol	estradiol	32042192	Endocrine	1	1	0	1	22	206927	NA	NA	EUR	0.023	0.0061	0.023	0.0061
Hyperthyroidism	hyperthyroidism	34594039	Endocrine	1	0	NA	1	11	460499	3557	456942	NA	NA	NA	NA	NA
Hypothyroidism	hypothyroidism	36093044	Endocrine	1	1	1	1	69	494577	51194	443383	EUR	0.1399	0.013	0.0488	0.0045
Precocious puberty (males)	precociouspuber	32210231	Endocrine	1	1	NA	1	76	205354	NA	NA	NA	NA	NA	NA	NA
Total testosterone levels	testosterone	32042192	Endocrine	1	1	1	1	254	230454	NA	NA	EUR	0.1489	0.0096	0.1489	0.0096
Years ovulating	ovulating	36320039	Endocrine	1	1	1	1	19	117193	NA	NA	EUR	0.0829	0.0067	0.0829	0.0067
Health satisfaction	healthhappy	31427789[a]	Functional	1	1	1	0	2	128745	NA	NA	EUR	0.074	0.0045	0.074	0.0045
Health-related quality of life	qol	29855537	Functional	0	NA	NA	NA	NA	NA	NA	NA	NA	NA	NA	NA	NA
Long illness or disability	disability	31427789[a]	Functional	1	1	1	1	17	377498	122262	255236	EUR	0.079	0.0037	0.0466	0.0022
Medication and treatment use	treatmentuse	31427789[a]	Functional	1	1	1	1	56	386581	NA	NA	EUR	0.0777	0.0027	0.0777	0.0027
Self-reported health	health	27864402	Functional	1	1	1	1	13	111749	NA	NA	EUR	0.0971	0.0057	0.0971	0.0057
Unable to work	unablework	31427789[a]	Functional	1	1	0	0	0	385138	370400	370400	EUR	0.0131	0.0012	0.0084	7e-04
CRP levels	crp	35459240	Inflammation	1	1	1	1	266	575531	NA	NA	EUR	0.1145	0.0091	0.1145	0.0091
Cytokine	cytokine	22610502	Inflammation	0	NA	NA	NA	NA	NA	NA	NA	NA	NA	NA	NA	NA
IL2 levels	il2	27989323	Inflammation	1	0	NA	NA	NA	NA	NA	NA	NA	NA	NA	NA	NA
IL6 levels	il6	33517400	Inflammation	1	1	1	1	9	52654	NA	NA	EUR	0.0528	0.0098	0.0528	0.0098
TNF	tnf	27989323	Inflammation	1	0	NA	NA	NA	NA	NA	NA	NA	NA	NA	NA	NA
Early onset major depressive disor	MDD_earlyonset	NA	MDD	NA	NA	NA	NA	NA	156433	46930	109503	NA	NA	NA	NA	NA
Late onset major depressive disor	MDD_lateonset	NA	MDD	NA	NA	NA	NA	NA	247342	74203	173139	NA	NA	NA	NA	NA
Major depressive disorder	MDD_Nordic	NA	MDD	NA	NA	NA	NA	NA	512914	148745	364169	NA	NA	NA	NA	NA
Major depressive disorder	MDD_PGC3	NA	MDD	NA	NA	NA	NA	NA	2000702	412305	1588397	NA	NA	NA	NA	NA
Major depressive disorder	MDD_Als	37464041	MDD	NA	NA	1	NA	NA	1035760	166773	507679	EUR	0.0809	0.0029	0.0457	0.0016
BMI	BMI	30239722	Metabolic	1	1	1	1	670	795640	NA	NA	EUR	0.2094	0.0062	0.2094	0.0062
Cholesterol	cholesterol	33462484	Metabolic	1	1	1	1	596	1320016	NA	NA	cross	0.0893	0.0083	0.0893	0.0083

High density lipoprotein	hdl	34887591	Metabolic	1	1	1	1	380	1244580	NA	NA	EUR	0.1072	0.0071	0.1072	0.0071
Low density lipoprotein	ldl	34887591	Metabolic	1	1	1	1	403	1320016	NA	NA	EUR	0.0765	0.0074	0.0765	0.0074
Metabolic syndrome	metabolicsyn	31589552	Metabolic	1	1	1	1	93	291108	NA	NA	EUR	0.0923	0.0049	0.0923	0.0049
Triglycerides	tgl	34887591	Metabolic	1	1	1	1	388	1253277	NA	NA	EUR	0.0919	0.0082	0.0919	0.0082
Type I diabetes	t1d	34127860	Metabolic	1	1	0	1	33	520580	18942	501638	EUR	0.0293	0.0039	0.0293	0.0039
Type II diabetes	t2d	35551307	Metabolic	1	1	1	1	338	933970	80154	853816	EUR	0.0469	0.0023	0.0469	0.0023
Healthspan	healthspan	30729179	Mortality	1	1	0	1	12	300447	NA	NA	EUR	0.0291	0.0025	0.0291	0.0025
Parental longevity	parentlongevity	27015805	Mortality	1	1	1	1	51	45627	NA	NA	EUR	0.0472	0.0116	0.0472	0.0116
Parental mortality	parentlife	29227965	Mortality	1	1	1	1	38	389166	NA	NA	EUR	0.0317	0.0023	0.0317	0.0023
Exercise (self report)	exercise	36071172	Phys activity	1	1	1	1	8	608595	291813	316782	EUR	0.0443	0.0025	0.0282	0.0016
Heart rate variability	heartvaria	28613276	Phys activity	1	1	1	1	22	26523	NA	NA	EUR	0.1069	0.0288	0.1069	0.0288
Oxygen uptake	oxyuptake	34753499	Phys activity	1	1	1	1	12	70783	NA	NA	EUR	0.1052	0.0083	0.1052	0.0083
Physical activity	physactivity	30531941	Phys activity	1	1	1	1	6	91105	NA	NA	EUR	0.1435	0.0082	0.1435	0.0082
Sedentary behavior	sedentary	30531941	Phys activity	1	1	1	1	6	91105	NA	NA	EUR	0.1144	0.0081	0.1144	0.0081
Age at menopause	menopause	34349265	Reproduction	1	1	1	1	290	201323	NA	NA	EUR	0.1722	0.0129	0.1722	0.0129
Age first birth	agefirstbirth	27798627	Reproduction	1	1	1	1	10	238064	NA	NA	EUR	0.0579	0.0034	0.0579	0.0034
Age last birth	agelastbirth	36320039	Reproduction	1	1	1	1	11	201371	NA	NA	EUR	0.0883	0.0045	0.0883	0.0045
Erectile dysfunction	erectile	30583798	Reproduction	1	1	1	1	23	223805	6175	217630	EUR	0.0465	0.0134	0.007	0.002
Number of children	numberchildren	31427789[a]	Reproduction	1	1	1	1	9	208434	NA	NA	EUR	0.0626	0.0039	0.0626	0.0039
Offspring birth weight	offspringbirthweigh	29309628	Reproduction	1	1	1	0	2	68258	NA	NA	EUR	0.0851	0.0085	0.0851	0.0085
Pregnancy terminations	pregnantermin	31427789[a]	Reproduction	1	1	1	1	14	65225	NA	NA	EUR	0.0532	0.0111	0.0532	0.0111
Still births	stillbirths	31427789[a]	Reproduction	1	1	0	1	32	65639	NA	NA	EUR	0.0124	0.0086	0.0124	0.0086
Age at smoking initiation	smokingage	36477530	Risk behav	1	1	1	1	26	618541	NA	NA	EUR	0.0479	0.0027	0.0479	0.0027
Alcohol abuse	alcoholabuse18	30336701	Risk behav	1	1	1	1	5	121604	NA	NA	EUR	0.0601	0.005	0.0601	0.005
Alcohol dependence	alcoholdep	30482948	Risk behav	1	1	1	1	5	46568	11569	34999	cross	0.1121	0.0176	0.0602	0.0094
Alcohol per week	alcoholweek	36477530	Risk behav	1	1	1	1	501	2428851	NA	NA	EUR	0.0546	0.0024	0.0546	0.0024
Cannabis dependence	cannabisdep	33096046	Risk behav	1	1	1	0	4	374287	17068	357219	cross	0.0904	0.0081	0.0191	0.0017
Cigarettes per day	cigperday	36477530	Risk behav	1	1	1	1	153	618489	NA	NA	EUR	0.0745	0.004	0.0745	0.004
Externalizing	externalizing	34446935	Risk behav	1	1	1	1	579	1045957	NA	NA	EUR	0.0628	0.0024	0.0628	0.0024
Lifetime cannabis use	cannabisever	NA	Risk behav	0	NA	NA	NA	NA	NA	NA	NA	NA	NA	NA	NA	NA
Maternal smoking	smokingmaternal	31427789[a]	Risk behav	1	1	1	1	16	331862	100733	231129	EUR	0.08	0.0042	0.0462	0.0024
Methamphetamine dependence	meth	31462767	Risk behav	0	NA	NA	NA	NA	NA	NA	NA	NA	NA	NA	NA	NA
Nicotine dependence	nicdep	28440896	Risk behav	0	NA	NA	NA	NA	NA	NA	NA	NA	NA	NA	NA	NA
Opioid dependence	opioid	32099098	Risk behav	0	NA	NA	NA	NA	NA	NA	NA	NA	NA	NA	NA	NA
Risky behaviours	riskbehavior	30643258	Risk behav	1	1	1	1	312	975353	NA	NA	EUR	0.0367	0.0015	0.0367	0.0015
Smoking cessation	smokingces	36477530	Risk behav	1	1	1	1	125	1147272	304244.14	843027.86	EUR	0.0487	0.0023	0.0487	0.0023
Smoking initiation	smokingever	36477530	Risk behav	1	1	1	1	1752	2669029	1175108	1493921	EUR	0.0879	0.0029	0.0879	0.0029
Living with partner	partner	34737426[b]	Social	1	1	1	1	20	453446	333559	119887	EUR	0.0421	0.0026	0.0232	0.0015
Loneliness	loneliness	29970889	Social	1	1	1	1	15	445024	80134	364890	EUR	0.0374	0.0019	0.0374	0.0019
Separation/ divorce	divorce	34737426[b]	Social	1	1	0	1	6	453246	13871	439375	EUR	0.0164	0.0067	0.0026	0.0011
Financial difficulties	financialdiff	34737426[b]	Socioecon	1	1	1	1	42	453246	52374	400872	EUR	0.0884	0.0046	0.0328	0.0017
Household income	income	31844048	Socioecon	1	1	1	1	343	505541	NA	NA	EUR	0.0841	0.003	0.0841	0.003
Infant mortality rate birth region	infantmortal	35290122	Socioecon	1	1	0	0	2	330340	NA	NA	EUR	0.016	0.0025	0.016	0.0025
Management demands	manager	35286190	Socioecon	1	1	1	1	8	219474	NA	NA	EUR	0.0514	0.0032	0.0514	0.0032
Mother's education	mothereducation	29987013	Socioecon	0	NA	NA	NA	NA	NA	NA	NA	NA	NA	NA	NA	NA
Social deprivation	deprivation	27818178	Socioecon	1	1	NA	0	0	112005	NA	NA	NA	NA	NA	NA	NA
Suicidal behavior	suicbehav	34861974	Suicide	1	1	1	0	2	549743	29782	519961	EUR	0.0876	0.0061	0.0199	0.0014
Suicidal thought	suicthought	36515925	Suicide	1	1	1	NA	NA	633778	121211	512567	EUR	0.0441	0.0024	0.0212	0.0011
Suicide death	suicdeath	32998551	Suicide	1	0	1	0	2	18223	3413	14810	EUR	0.2223	0.0332	0.2223	0.0332
Childhood maltreatment	childmal	32066696	Trauma	1	1	1	0	3	124713	NA	NA	EUR	0.0585	0.0045	0.0585	0.0045
Death of relative	deathofrelative	34737426[b]	Trauma	1	1	0	0	3	453246	95788	357458	EUR	0.0118	0.0023	0.0059	0.0012
Serious accident	accident	34737426[b]	Trauma	1	1	0	1	9	266534	133267	133267	EUR	0.0129	0.0029	0.0082	0.0018
Traumatic injury	traumainjury	34737426[b]	Trauma	1	1	1	1	9	453246	42167	411079	EUR	0.0416	0.0039	0.0137	0.0013

[a] From the GWAS Atlas repository, including 2019 GWAS on all UK-Biobank phenotypes, https://atlas.ctglab.nl/

[b] From the Yang lab fastGWA repository of 2019 GWAS on all UK-Biobank phenotypes, https://yanglab.westlake.edu.cn/software/gcta/#UKBiobankGWASresults

Table S3. Trait selection for OSMR. The reason why the trait was not included in TSMR is listed, along with its availability for OSMR in the UK-Biobank. Whenever hospital records were used to capture the trait, the code as used in the International Classification of Disease (ICD) is extracted (10th edition).

Reason	Trait	Category	Available for OSMR	shorttraitname	UKB trait	datafield	ICD code
insufficient hits	Preterm birth	Birth weight	0	NA	NA	NA	NA
insufficient h2	Job involves night shifts	Circadian	1	nightshifts	Job involves night shifts	3426	NA
no GWAS	school leaving age	Cognition	1	educationage	Age completed education	845	NA
insufficient hits	Anemia	Diet	1	anemia	Anemia*	NA	D50
no GWAS	calcium intake	Diet	1	calcium	Calcium intake	100024	NA
insufficient h2	Fish consumption	Diet	1	fish	Fish consumer	103140	NA
insufficient hits	Fruit consumption	Diet	1	fruit	Fruit consumer	104400	NA
no GWAS	iron intake	Diet	1	iron	Iron intake	26019	NA
insufficient hits	Magnesium levels	Diet	1	magnesium	Magnesium intake	26025	NA
no GWAS	omega 3	Diet	1	oilyfish	Oily fish intake	1329	NA
no GWAS	adherence to mediterranean diet	Diet	0	NA	NA	NA	NA
no GWAS	dietary inflammation index	Diet	0	NA	NA	NA	NA
insufficient h2	Celiac disease	Disease	1	celiac	Celiac disease*	NA	K90.0
insufficient h2	Chronic sinusitis	Disease	1	sinusitis	Chronic sinusitis*	NA	J32
insufficient h2	Herpes simplex virus	Disease	1	herpes	Herpes simplex infection*	NA	B00
insufficient h2	Psoriasis	Disease	1	psoriasis	Psoriasis*	NA	L40
insufficient h2	Stroke	Disease	1	stroke	Stroke*	NA	I63
insufficient hits	Tinnitus	Disease	1	tinnitus	Tinnitus*	NA	H93.1
insufficient h2	Anti-varicella zoster virus	Disease	1	varicella	Varicella*	NA	B01
no GWAS	Borna disease virus infection	Disease: infectious	0	NA	NA	NA	NA
insufficient h2	Precocious puberty (males)	Endocrine	1	puberty	Age voice broke	2385	NA
no GWAS	cushing's disease	Endocrine	1	cushing	Cushing's syndrome*	NA	E24
insufficient hits	Contraceptive pill	Endocrine	1	contrapill	Ever used contraceptive pill	2784	NA
insufficient h2	Estradiol	Endocrine	1	oestradiol	Oestradiol levels	30800	NA
insufficient hits	Health satisfaction	Functional	1	healthsatis	Health dissatisfaction	4548	NA
no GWAS	drop out high school	Functional	1	dropout	Highschool drop-out	6138	NA
no GWAS	any hospitalization	Functional	1	hospitalizations	Hospitalizations	41235	NA
no GWAS	overtime work	Functional	1	workhours	Length working week	767	NA
insufficient h2	Unable to work	Functional	1	sicknessleave	Unable to work	6142	NA
no GWAS	sickness absence	Functional	1	sicknessleave	Unable to work	6142	NA
no GWAS	employment	Functional	1	employment	Employment status	6142	NA
no GWAS	school absence	Functional	0	NA	NA	NA	NA
no GWAS	global assessment of functioning	Functional	1	mobility	Mobility problems	120098	NA
no GWAS	global assessment of functioning	Functional	1	pain	Pain/discomfort	120101	NA
no GWAS	global assessment of functioning	Functional	1	dailyactiv	Problems doing usual activities	120100	NA
no GWAS	global assessment of functioning	Functional	1	selfcare	Self-care problems	120099	NA
no GWAS	global assessment of functioning	Functional	1	subhealth	Subjective health rating	120103	NA
no GWAS	accidents	Mortality	1	accidents	Accidents*	NA	V, W, X0-X56
no GWAS	alcohol death	Mortality	1	alcdeath	Alcohol-related death*	NA	E24.4 F10 G31.2 G62.1 G72.1 I42.6 K29.2 K70 K85.2 K86.0 Q86.0 R78.0 X45 X65 Y15
insufficient N	Healthspan	Mortality	0	NA	NA	NA	NA
insufficient h2	Healthspan	Mortality	0	NA	NA	NA	NA
insufficient hits	Offspring birth weight	Reproduction	1	offspringbirthweight	Birth weight first child	2744	NA
no GWAS	sickness absence during pregnancy	Reproduction	0	NA	NA	NA	NA
insufficient h2	Still births	Reproduction	1	stillbirths	Number of stillbirths	3829	NA
no GWAS	unintended pregnancy	Reproduction	1	pregnancytermin	Pregnancy terminations	3849	NA
insufficient hits	Cannabis dependence	Risk behav	1	cannabisabuse	Cannabis use disorder*	NA	F12
no GWAS	civic participation	Social	0	NA	NA	NA	NA
insufficient h2	Separation/ divorce	Social	1	divorce	Marital separation/ divorce	6145	NA
no GWAS	lower relationship quality	Social: partner	0	NA	NA	NA	NA
insufficient h2	Social deprivation	Socioecon	1	deprivation	Townsend deprivation index	189	NA
insufficient h2	Infant mortality rate birth region	Socioecon	0	NA	NA	NA	NA
no GWAS	minority ethnic group	Socioeconomic status	0	NA	NA	NA	NA
no GWAS	parental income	Socioeconomic status	0	NA	NA	NA	NA
insufficient hits	Suicidal behavior	Suicide	1	suicattempt	Attempted suicide	20483	NA
insufficient hits	Suicidal behavior	Suicide	1	selfharm	Self-harm*	NA	X6 X7 X81 X82 X83 X84 E95
insufficient h2	Serious accident	Trauma	1	accidents_sr	Been in serious accident	20526	NA
no GWAS	military combat	Trauma	1	combat	Exposure to war	20527	NA
no GWAS	relationship conflict	Trauma	1	familyrel	Family relationship satisfaction	20522	NA
no GWAS	intimate partner violence against women	Trauma	1	partnerabuse	Partner violence	20523	NA
no GWAS	academic stress	Trauma/ stress: adult	0	NA	NA	NA	NA
no GWAS	life events	Trauma/ stress: adult	0	NA	NA	NA	NA
no GWAS	natural disaster	Trauma/ stress: adult	0	NA	NA	NA	NA
no GWAS	prenatal stress	Trauma/ stress: adult	0	NA	NA	NA	NA
no GWAS	separation	Trauma/ stress: adult	0	NA	NA	NA	NA
no GWAS	sole parenthood	Trauma/ stress: adult	0	NA	NA	NA	NA
no GWAS	terrorist act	Trauma/ stress: adult	0	NA	NA	NA	NA
no GWAS	bullying	Trauma/ stress: childhood	0	NA	NA	NA	NA
no GWAS	parental divorce	Trauma/ stress: childhood	0	NA	NA	NA	NA

Table S4. Genetic correlation and SNP-heritability results from Ldscore regression. The rg_MDD

GWAS trai	Category	rg_MDD	SE	P	liability_h2	liability_SE	scale_h2	scale_SE
Preterm bi	Birth weigh	-0.079	0.033	0.018	0.074	0.008	0.074	0.008
Chronotyp	Circadian	-0.043	0.021	0.040	0.111	0.004	0.111	0.004
Insomnia	Circadian	0.485	0.027	2.95E-73	0.046	0.002	0.046	0.002
Job involve	Circadian	0.262	0.040	7.28E-11	0.024	0.003	0.024	0.003
Sleep dura	Circadian	0.091	0.027	9.10E-04	0.132	0.008	0.132	0.008
Educationa	Cognition	-0.249	0.017	2.77E-46	0.157	0.004	0.157	0.004
Executive f	Cognition	-0.213	0.021	3.18E-25	0.093	0.004	0.093	0.004
Fluid intelli	Cognition	-0.174	0.046	1.48E-04	0.054	0.008	0.034	0.005
Anemia	Diet	0.307	0.048	1.77E-10	0.042	0.009	0.006	0.001
Coffee con	Diet	-0.156	0.051	0.002	0.061	0.013	0.035	0.008
Variation ir	Diet	0.199	0.024	2.42E-16	0.042	0.002	0.042	0.002
Fish consu	Diet	-0.224	0.106	0.034	0.014	0.013	0.008	0.008
Fruit consu	Diet	-0.427	0.058	1.31E-13	0.067	0.015	0.032	0.007
Magnesiun	Diet	0.016	0.071	0.821	0.061	0.024	0.061	0.024
Tea consur	Diet	-0.058	0.046	0.211	0.093	0.015	0.046	0.007
Vegetable	Diet	-0.155	0.068	0.023	0.044	0.017	0.020	0.008
25-Hydroxy	Diet	-0.049	0.058	0.401	0.045	0.015	0.045	0.015
Zinc supple	Diet	0.253	0.035	4.54E-13	0.059	0.006	0.012	0.001
Adenocarc	Disease	0.039	0.055	0.476	0.064	0.020	0.032	0.010
Amyotroph	Disease	0.151	0.050	0.002	0.054	0.012	0.029	0.006
Alzheimer'	Disease	-0.098	0.050	0.048	0.046	0.011	0.046	0.011
Asthma	Disease	0.153	0.031	5.65E-07	0.171	0.022	0.067	0.008
Atrial Fibril	Disease	0.112	0.020	4.48E-08	0.096	0.008	0.024	0.002
Malignant	Disease	-0.098	0.049	0.048	0.095	0.022	0.045	0.011
Malignant	Disease	-0.083	0.031	0.008	0.242	0.032	0.135	0.018
Multisite c	Disease	0.580	0.026	1.13E-111	0.074	0.003	0.074	0.003
Chronic ob	Disease	0.010	0.030	0.738	0.228	0.022	0.061	0.006
Coronary a	Disease	0.248	0.018	5.97E-42	0.077	0.005	0.033	0.002
Severe cov	Disease	0.133	0.030	1.15E-05	0.079	0.015	0.007	0.001
Crohn's dis	Disease	0.144	0.024	3.09E-09	0.370	0.034	0.370	0.034
Diastolic bl	Disease	0.025	0.015	0.082	0.109	0.004	0.109	0.004
Eczema	Disease	0.207	0.040	2.27E-07	0.005	1.00E-03	0.005	1.00E-03
Generalize	Disease	-0.015	0.031	0.640	0.601	0.045	0.601	0.045
Gastroesor	Disease	0.567	0.025	1.30E-117	0.068	0.002	0.068	0.002
Heart failu	Disease	0.286	0.032	5.20E-19	0.008	7.00E-04	0.008	7.00E-04
Herpes sim	Disease	0.013	0.173	0.940	0.018	0.050	0.018	0.050
HIV suscep	Disease	0.236	0.043	3.93E-08	0.073	0.011	0.006	9.00E-04
Inflammat	Disease	0.136	0.024	2.96E-08	0.311	0.026	0.311	0.026
Self-report	Disease	0.588	0.026	1.85E-114	0.070	0.003	0.070	0.003
Irritable bc	Disease	0.549	0.051	4.19E-27	0.053	0.010	0.008	0.002

Systemic lu	Disease	0.040	0.035	0.252	0.845	0.093	0.515	0.056
Migraine	Disease	0.297	0.022	1.59E-40	0.029	0.002	0.029	0.002
Multiple sc	Disease	0.105	0.026	4.63E-05	0.285	0.023	0.285	0.023
Parkinson (	Disease	4.11E-04	0.040	0.992	0.102	0.017	0.102	0.017
Pelvic orga	Disease	0.215	0.027	1.03E-15	0.081	0.006	0.018	0.001
Peripheral	Disease	0.304	0.040	4.20E-14	0.064	0.009	0.009	0.001
Rheumatoi	Disease	0.044	0.024	0.071	0.233	0.018	0.121	0.010
Chronic sin	Disease	0.445	0.064	2.67E-12	0.029	0.010	0.003	1.00E-03
Staphyloco	Disease	0.179	0.051	4.95E-04	0.058	0.013	0.006	0.001
Stroke	Disease	0.165	0.025	3.87E-11	0.008	6.00E-04	0.008	6.00E-04
Systolic blo	Disease	0.023	0.015	0.121	0.116	0.004	0.116	0.004
Tinnitus	Disease	0.315	0.030	3.39E-25	0.063	0.005	0.063	0.005
Ulcerative	Disease	0.084	0.028	0.003	0.266	0.026	0.266	0.026
Contracept	Endocrine	-0.138	0.039	3.57E-04	0.030	0.003	0.030	0.003
Estradiol	Endocrine	-0.068	0.039	0.084	0.023	0.006	0.023	0.006
Hypothyro	Endocrine	0.240	0.019	1.23E-35	0.140	0.013	0.049	0.005
Age at mer	Endocrine	-0.052	0.019	0.006	0.234	0.010	0.234	0.010
Years ovula	Endocrine	-0.037	0.029	0.198	0.083	0.007	0.083	0.007
Total testo	Endocrine	-0.065	0.018	3.67E-04	0.149	0.010	0.149	0.010
Long illness	Functional	0.543	0.028	5.07E-81	0.079	0.004	0.047	0.002
Self-report	Functional	0.590	0.035	8.11E-65	0.097	0.006	0.097	0.006
Health sati	Functional	-0.605	0.033	5.05E-73	0.074	0.005	0.074	0.005
Medicatior	Functional	0.493	0.023	1.43E-104	0.078	0.003	0.078	0.003
Unable to v	Functional	0.655	0.039	1.01E-63	0.013	0.001	0.008	7.00E-04
CRP levels	Inflammati	0.162	0.016	3.41E-23	0.115	0.009	0.115	0.009
IL6 levels	Inflammati	0.226	0.051	9.25E-06	0.053	0.010	0.053	0.010
BMI	Metabolic	0.135	0.017	3.29E-16	0.209	0.006	0.209	0.006
Cholestero	Metabolic	0.045	0.013	3.86E-04	0.089	0.008	0.089	0.008
High densit	Metabolic	-0.136	0.014	4.77E-21	0.107	0.007	0.107	0.007
Low densit	Metabolic	0.034	0.013	0.011	0.077	0.007	0.077	0.007
Metabolic	Metabolic	0.225	0.021	9.99E-28	0.092	0.005	0.092	0.005
Type I diab	Metabolic	-0.032	0.024	0.178	0.029	0.004	0.029	0.004
Type II diat	Metabolic	0.193	0.018	5.01E-27	0.047	0.002	0.047	0.002
Triglyceride	Metabolic	0.190	0.016	3.40E-34	0.092	0.008	0.092	0.008
Healthspar	Mortality	0.366	0.032	5.92E-31	0.029	0.003	0.029	0.003
Parental m	Mortality	0.230	0.030	8.13E-15	0.032	0.002	0.032	0.002
Parental lo	Mortality	-0.217	0.054	6.59E-05	0.047	0.012	0.047	0.012
Exercise (se	Phys activit	-0.332	0.027	7.76E-35	0.044	0.003	0.028	0.002
Heart rate	Phys activit	-0.005	0.055	0.924	0.107	0.029	0.107	0.029
Oxygen up	Phys activit	-0.118	0.034	4.25E-04	0.105	0.008	0.105	0.008
Physical ac	Phys activit	-0.137	0.026	2.14E-07	0.144	0.008	0.144	0.008
Sedentary	Phys activit	0.021	0.029	0.481	0.114	0.008	0.114	0.008
Age first bi	Reproducti	-0.014	0.008	0.093	0.058	0.003	0.058	0.003

Age last bi	Reproducti	-0.330	0.027	5.81E-34	0.088	0.005	0.088	0.005
Erectile dy	Reproducti	0.366	0.060	1.39E-09	0.047	0.013	0.007	0.002
Age at mer	Reproducti	-0.113	0.019	3.57E-09	0.172	0.013	0.172	0.013
Number of	Reproducti	0.192	0.028	1.01E-11	0.063	0.004	0.063	0.004
Offspring b	Reproducti	-0.016	0.032	0.629	0.085	0.009	0.085	0.009
Pregnancy	Reproducti	0.167	0.048	4.62E-04	0.053	0.011	0.053	0.011
Still births	Reproducti	0.245	0.093	0.009	0.012	0.009	0.012	0.009
Alcohol ab	Risk behav	0.289	0.035	2.75E-16	0.060	0.005	0.060	0.005
Alcohol de	Risk behav	0.002	0.015	0.897	0.112	0.018	0.060	0.009
Alcohol pe	Risk behav	0.083	0.023	3.49E-04	0.055	0.002	0.055	0.002
Cannabis d	Risk behav	0.439	0.033	1.85E-41	0.090	0.008	0.019	0.002
Cigarettes	Risk behav	0.272	0.024	1.99E-30	0.075	0.004	0.075	0.004
Externalizi	Risk behav	0.389	0.025	6.29E-56	0.063	0.002	0.063	0.002
Risky beha	Risk behav	0.212	0.022	5.32E-22	0.037	0.002	0.037	0.002
Age at smc	Risk behav	-0.313	0.024	2.03E-37	0.048	0.003	0.048	0.003
Smoking ce	Risk behav	0.283	0.026	1.36E-27	0.049	0.002	0.049	0.002
Smoking in	Risk behav	0.362	0.022	5.56E-62	0.088	0.003	0.088	0.003
Maternal s	Risk behav	0.296	0.027	1.26E-28	0.080	0.004	0.046	0.002
Separation	Social	0.710	0.081	1.43E-18	0.016	0.007	0.003	0.001
Loneliness	Social	0.689	0.031	5.85E-112	0.037	0.002	0.037	0.002
Living with	Social	-0.223	0.032	5.96E-12	0.042	0.003	0.023	0.002
Financial d	Socioecon	0.552	0.032	3.76E-67	0.088	0.005	0.033	0.002
Household	Socioecon	-0.326	0.021	6.78E-54	0.084	0.003	0.084	0.003
Infant mor	Socioecon	0.174	0.041	2.35E-05	0.016	0.003	0.016	0.003
Manageme	Socioecon	0.015	0.021	0.471	0.051	0.003	0.051	0.003
Suicidal be	Suicide	0.751	0.034	1.00E-105	0.088	0.006	0.020	0.001
Suicide dea	Suicide	0.507	0.047	5.83E-27	0.222	0.033	0.222	0.033
Suicidal th	Suicide	0.839	0.036	9.32E-119	0.044	0.002	0.021	0.001
Serious acc	Trauma	0.445	0.060	1.86E-13	0.013	0.003	0.008	0.002
Childhood	Trauma	0.615	0.036	5.28E-65	0.059	0.005	0.059	0.005
Death of re	Trauma	0.282	0.046	8.11E-10	0.012	0.002	0.006	0.001
Traumatic	Trauma	0.577	0.039	1.68E-49	0.042	0.004	0.014	0.001

Table S4. Full two-sample MR (TSMR) results from main and sensitivity analyses. The p_fdr column contains the p-value corrected for multiple testing within the set of analyses (MDD as exposure or MDD as outcome). When availbale, the number of SNP instruments included in the analysis is given in 'nsnp'. The power is given for an effect size of β =0.1 (or OR=1.1) and β =0.3 (or OR=1.3), as well as the effect size that would be needed to achieve 80% power (β for continous traits, OR for binary traits). R^2 is the explained variance in the exposure by the instrument and F is the instrument strength. I^2 is used to assess the no measurement error assumption violation to determine if the MR Egger estimate can be interpreted; if it is between 0.6-0.8 the MR Egger estimate is corrected with a SIMEX estimator. The MR Egger intercept indicates the presence of pleiotropy (if the p is below .05 the result should not be interpreted).

Outcome	Exposure	Abbreviation	Method	nsnp	b	se	p	p_fdr	Power_at_effect_size_0	Power_at_effect_size_0	Effect_size_at_80_pow	R^2	F	I^2	I^2_weight	egger_intercept	egger_se	egger_p	Q	Q_pval
25-Hydroxyvitamin D level	MDD	vitamind	MR Egger	151	0.029	0.130	0.824	0.884	45.465	99.983	0.152					-0.001	0.003	0.824		
25-Hydroxyvitamin D level	MDD	vitamind	Weighted median	151	-0.028	0.039	0.470	0.550	45.465	99.983	0.152									
25-Hydroxyvitamin D level	MDD	vitamind	Inverse variance weighte	151	6.83E-04	0.028	0.980	0.988	45.465	99.983	0.152	0.0091	40.720	0.466	unweighted				173.489	0.092
25-Hydroxyvitamin D level	MDD	vitamind	Simple mode	151	-0.087	0.121	0.471	0.699	45.465	99.983	0.152									
25-Hydroxyvitamin D level	MDD	vitamind	Weighted mode	151	-0.076	0.103	0.461	0.632	45.465	99.983	0.152									
25-Hydroxyvitamin D level	MDD	vitamind	Steiger-filtered IVW	151	6.83E-04	0.028	0.980	0.980	45.465	99.983	0.152									
25-Hydroxyvitamin D level	MDD	vitamind	GSMR	151	2.66E-05	0.026	0.999	0.999	45.465	99.983	0.152									
25-Hydroxyvitamin D level	MDD	vitamind	LHC		0.032	0.096	0.739	0.793	45.465	99.983	0.152									
Adenocarcinoma	MDD	adenocarcinoma	MR Egger	154	0.180	0.284	0.528	0.788	12.767	61.949	1.384					-0.003	0.007	0.633		
Adenocarcinoma	MDD	adenocarcinoma	Weighted median	154	0.067	0.090	0.459	0.544	12.767	61.949	1.384									
Adenocarcinoma	MDD	adenocarcinoma	Inverse variance weighte	154	0.047	0.061	0.443	0.532	12.767	61.949	1.384	0.0093	40.626	0.459	unweighted				159.138	0.350
Adenocarcinoma	MDD	adenocarcinoma	Simple mode	154	0.051	0.268	0.851	0.958	12.767	61.949	1.384									
Adenocarcinoma	MDD	adenocarcinoma	Weighted mode	154	0.089	0.257	0.730	0.902	12.767	61.949	1.384									
Adenocarcinoma	MDD	adenocarcinoma	Steiger-filtered IVW	154	0.047	0.061	0.443	0.525	12.767	61.949	1.384									
Adenocarcinoma	MDD	adenocarcinoma	GSMR	154	0.042	0.061	0.492	0.554	12.767	61.949	1.384									
Adenocarcinoma	MDD	adenocarcinoma	LHC		-0.030	0.029	0.300	0.428	12.767	61.949	1.384									
Age at menarche	MDD	menarche	MR Egger	154	0.136	0.188	0.469	0.746	96.905	100.000	0.073					-0.005	0.004	0.264		
Age at menarche	MDD	menarche	Weighted median	154	-0.056	0.036	0.115	0.157	96.905	100.000	0.073									
Age at menarche	MDD	menarche	Inverse variance weighte	154	-0.069	0.041	0.090	0.128	96.905	100.000	0.073	0.0093	40.626	0.459	unweighted				639.784	9.60E-61
Age at menarche	MDD	menarche	Simple mode	154	-0.137	0.137	0.320	0.499	96.905	100.000	0.073									
Age at menarche	MDD	menarche	Weighted mode	154	-0.112	0.117	0.340	0.522	96.905	100.000	0.073									
Age at menarche	MDD	menarche	Steiger-filtered IVW	150	-0.041	0.034	0.224	0.281	96.905	100.000	0.073									
Age at menarche	MDD	menarche	GSMR	143	-0.054	0.021	0.011	0.017	96.905	100.000	0.073									
Age at menarche	MDD	menarche	PRESSO	154	-0.044	0.032	0.174	0.205	96.905	100.000	0.073									
Age at menarche	MDD	menarche	LHC		-0.579	0.212	0.006	0.013	96.905	100.000	0.073									
Age at menopause	MDD	menopause	MR Egger	152	0.754	0.374	0.045	0.193	79.865	100.000	0.100					-0.021	0.009	0.015		
Age at menopause	MDD	menopause	Weighted median	152	-0.249	0.079	0.002	0.003	79.865	100.000	0.100									
Age at menopause	MDD	menopause	Inverse variance weighte	152	-0.147	0.081	0.068	0.102	79.865	100.000	0.100	0.0092	40.701	0.463	unweighted				420.065	3.36E-27
Age at menopause	MDD	menopause	Simple mode	152	-0.370	0.255	0.148	0.346	79.865	100.000	0.100									
Age at menopause	MDD	menopause	Weighted mode	152	-0.410	0.272	0.133	0.282	79.865	100.000	0.100									
Age at menopause	MDD	menopause	Steiger-filtered IVW	152	-0.147	0.081	0.068	0.100	79.865	100.000	0.100									
Age at menopause	MDD	menopause	GSMR	146	-0.251	0.051	8.32E-07	1.68E-06	79.865	100.000	0.100									
Age at menopause	MDD	menopause	PRESSO	152	-0.191	0.071	0.008	0.012	79.865	100.000	0.100									
Age at menopause	MDD	menopause	LHC		-0.165	0.028	7.16E-09	3.28E-08	79.865	100.000	0.100									
Age at smoking initiation	MDD	smokingage	MR Egger	154	-0.026	0.067	0.694	0.835	100.000	100.000	0.037					-0.002	0.002	0.180		
Age at smoking initiation	MDD	smokingage	Weighted median	154	-0.109	0.016	4.84E-12	1.87E-11	100.000	100.000	0.037									
Age at smoking initiation	MDD	smokingage	Inverse variance weighte	154	-0.115	0.015	4.19E-15	1.78E-14	100.000	100.000	0.037	0.0093	40.626	0.459	unweighted				350.985	1.36E-17
Age at smoking initiation	MDD	smokingage	Simple mode	154	-0.115	0.046	0.014	0.054	100.000	100.000	0.037									
Age at smoking initiation	MDD	smokingage	Weighted mode	154	-0.098	0.043	0.026	0.083	100.000	100.000	0.037									
Age at smoking initiation	MDD	smokingage	Steiger-filtered IVW	154	-0.115	0.015	4.19E-15	1.78E-14	100.000	100.000	0.037									
Age at smoking initiation	MDD	smokingage	GSMR	150	-0.111	0.010	5.20E-28	1.85E-27	100.000	100.000	0.037									
Age at smoking initiation	MDD	smokingage	PRESSO	154	-0.117	0.014	6.55E-15	2.13E-14	100.000	100.000	0.037									
Age at smoking initiation	MDD	smokingage	LHC		-0.300	0.061	7.92E-07	2.76E-06	100.000	100.000	0.037									
Age first birth	MDD	agefirstbirth	MR Egger	69	0.290	0.179	0.111	0.291	83.302	100.000	0.096					-0.006	0.004	0.122		
Age first birth	MDD	agefirstbirth	Weighted median	69	-0.008	0.026	0.749	0.813	83.302	100.000	0.096									
Age first birth	MDD	agefirstbirth	Inverse variance weighte	69	0.013	0.030	0.658	0.732	83.302	100.000	0.096	0.0042	40.604	0.920	weighted				317.334	7.41E-34
Age first birth	MDD	agefirstbirth	Simple mode	69	-0.068	0.055	0.223	0.397	83.302	100.000	0.096									
Age first birth	MDD	agefirstbirth	Weighted mode	69	-0.032	0.030	0.283	0.467	83.302	100.000	0.096									
Age first birth	MDD	agefirstbirth	Steiger-filtered IVW	69	0.013	0.030	0.658	0.741	83.302	100.000	0.096									
Age first birth	MDD	agefirstbirth	GSMR	57	-0.007	0.015	0.640	0.678	83.302	100.000	0.096									
Age first birth	MDD	agefirstbirth	PRESSO	69	0.031	0.025	0.219	0.255	83.302	100.000	0.096									
Age first birth	MDD	agefirstbirth	LHC		0.282	0.007	0.00E+00	0.00E+00	83.302	100.000	0.096									
Age last birth	MDD	agelastbirth	MR Egger	153	-1.167	0.457	0.012	0.087	55.005	99.999	0.134					0.006	0.011	0.543		
Age last birth	MDD	agelastbirth	Weighted median	153	-0.887	0.107	9.42E-17	4.66E-16	55.005	99.999	0.134									
Age last birth	MDD	agelastbirth	Inverse variance weighte	153	-0.895	0.099	1.11E-19	6.17E-19	55.005	99.999	0.134	0.0092	40.635	0.457	unweighted				370.861	2.57E-20
Age last birth	MDD	agelastbirth	Simple mode	153	-0.955	0.345	0.006	0.027	55.005	99.999	0.134									
Age last birth	MDD	agelastbirth	Weighted mode	153	-0.885	0.322	0.007	0.031	55.005	99.999	0.134									
Age last birth	MDD	agelastbirth	Steiger-filtered IVW	153	-0.895	0.099	1.11E-19	6.17E-19	55.005	99.999	0.134									
Age last birth	MDD	agelastbirth	GSMR	148	-0.930	0.067	1.17E-43	6.49E-43	55.005	99.999	0.134									
Age last birth	MDD	agelastbirth	PRESSO	153	-0.979	0.093	1.23E-19	6.14E-19	55.005	99.999	0.134									
Age last birth	MDD	agelastbirth	LHC		-0.359	0.619	0.562	0.662	55.005	99.999	0.134									
Alcohol abuse	MDD	alcoholabuse18	MR Egger	150	-0.014	0.025	0.566	0.788	91.291	100.000	0.084					0.001	######	0.075		
Alcohol abuse	MDD	alcoholabuse18	Weighted median	150	0.025	0.007	1.17E-04	2.81E-04	91.291	100.000	0.084									
Alcohol abuse	MDD	alcoholabuse18	Inverse variance weighte	150	0.029	0.005	8.48E-08	2.60E-07	91.291	100.000	0.084	0.0091	40.730	0.464	unweighted				256.411	1.07E-07
Alcohol abuse	MDD	alcoholabuse18	Simple mode	150	0.008	0.022	0.695	0.857	91.291	100.000	0.084									
Alcohol abuse	MDD	alcoholabuse18	Weighted mode	150	0.004	0.019	0.852	0.948	91.291	100.000	0.084									
Alcohol abuse	MDD	alcoholabuse18	Steiger-filtered IVW	150	0.029	0.005	8.48E-08	2.36E-07	91.291	100.000	0.084									
Alcohol abuse	MDD	alcoholabuse18	GSMR	148	0.029	0.004	1.28E-11	3.35E-11	91.291	100.000	0.084									
Alcohol abuse	MDD	alcoholabuse18	PRESSO	150	0.027	0.005	8.68E-07	1.82E-06	91.291	100.000	0.084									
Alcohol abuse	MDD	alcoholabuse18	LHC		0.466	0.070	2.03E-11	1.36E-10	91.291	100.000	0.084									
Alcohol dependence	MDD	alcoholdep	MR Egger	152	0.072	0.153	0.641	0.810	10.609	50.041	1.455					-0.003	0.004	0.372		
Alcohol dependence	MDD	alcoholdep	Weighted median	152	-0.035	0.040	0.381	0.458	10.609	50.041	1.455									
Alcohol dependence	MDD	alcoholdep	Inverse variance weighte	152	-0.063	0.032	0.054	0.082	10.609	50.041	1.455	0.0092	40.701	0.463	unweighted				234.576	1.54E-05
Alcohol dependence	MDD	alcoholdep	Simple mode	152	0.018	0.128	0.889	0.975	10.609	50.041	1.455									
Alcohol dependence	MDD	alcoholdep	Weighted mode	152	0.034	0.128	0.790	0.904	10.609	50.041	1.455									
Alcohol dependence	MDD	alcoholdep	Steiger-filtered IVW	152	-0.063	0.032	0.054	0.081	10.609	50.041	1.455									
Alcohol dependence	MDD	alcoholdep	GSMR	152	-0.056	0.026	0.033	0.046	10.609	50.041	1.455									
Alcohol dependence	MDD	alcoholdep	LHC		-0.051	0.010	1.46E-07	5.31E-07	10.609	50.041	1.455									
Alcohol per week	MDD	alcoholweek	MR Egger	45	-0.115	0.113	0.311	0.576	100.000	100.000	0.034					0.004	0.003	0.092		
Alcohol per week	MDD	alcoholweek	Weighted median	45	0.072	0.023	0.001	0.003	100.000	100.000	0.034									
Alcohol per week	MDD	alcoholweek	Inverse variance weighte	45	0.073	0.026	0.005	0.009	100.000	100.000	0.034	0.0028	42.298	0.504	unweighted				165.618	5.40E-16
Alcohol per week	MDD	alcoholweek	Simple mode	45	0.108	0.048	0.029	0.096	100.000	100.000	0.034									
Alcohol per week	MDD	alcoholweek	Weighted mode	45	0.079	0.042	0.066	0.184	100.000	100.000	0.034									
Alcohol per week	MDD	alcoholweek	Steiger-filtered IVW	45	0.073	0.026	0.005	0.009	100.000	100.000	0.034									
Alcohol per week	MDD	alcoholweek	GSMR	41	0.076	0.015	1.70E-07	3.53E-07	100.000	100.000	0.034									
Alcohol per week	MDD	alcoholweek	PRESSO	45	0.084	0.018	3.01E-05	5.44E-05	100.000	100.000	0.034									
Alcohol per week	MDD	alcoholweek	LHC		-0.280	0.247	0.256	0.377	100.000	100.000	0.034									
Alzheimer's disease	MDD	alzheimer	MR Egger	154	-0.144	0.332	0.664	0.810	22.218	90.821	1.250					0.001	0.008	0.872		
Alzheimer's disease	MDD	alzheimer	Weighted median	154	-0.054	0.102	0.593	0.660	22.218	90.821	1.250									
Alzheimer's disease	MDD	alzheimer	Inverse variance weighte	154	-0.092	0.072	0.200	0.262	22.218	90.821	1.250	0.0093	40.626	0.459	unweighted				173.300	0.125
Alzheimer's disease	MDD	alzheimer	Simple mode	154	-0.127	0.316	0.689	0.857	22.218	90.821	1.250									
Alzheimer's disease	MDD	alzheimer	Weighted mode	154	-0.080	0.296	0.788	0.904	22.218	90.821	1.250									
Alzheimer's disease	MDD	alzheimer	Steiger-filtered IVW	154	-0.092	0.072	0.200	0.254	22.218	90.821	1.250									
Alzheimer's disease	MDD	alzheimer	GSMR	154	-0.084	0.068	0.222	0.267	22.218	90.821	1.250									
Alzheimer's disease	MDD	alzheimer	LHC		0.057	0.106	0.591	0.685	22.218	90.821	1.250									
Amyotrophic lateral sclerosis	MDD	als	MR Egger	152	-0.525	0.285	0.068	0.215	17.293	79.968	1.300					0.013	0.007	0.046		
Amyotrophic lateral sclerosis	MDD	als	Weighted median	152	0.043	0.080	0.591	0.660	17.293	79.968	1.300									
Amyotrophic lateral sclerosis	MDD	als	Inverse variance weighte	152	0.035	0.061	0.564	0.644	17.293	79.968	1.300	0.0092	40.701	0.463	unweighted				188.362	0.021
Amyotrophic lateral sclerosis	MDD	als	Simple mode	152	0.065	0.272	0.812	0.938	17.293	79.968	1.300									
Amyotrophic lateral sclerosis	MDD	als	Weighted mode	152	-0.425	0.275	0.124	0.282	17.293	79.968	1.300									
Amyotrophic lateral sclerosis	MDD	als	Steiger-filtered IVW	152	0.035	0.061	0.564	0.652	17.293	79.968	1.300									
Amyotrophic lateral sclerosis	MDD	als	GSMR	152	0.030	0.056	0.591	0.641	17.293	79.968	1.300									
Amyotrophic lateral sclerosis	MDD	als	LHC		0.079	0.111	0.478	0.602	17.293	79.968	1.300									
Asthma	MDD	asthma	MR Egger	154	0.191	0.291	0.512	0.786	14.192	68.618	1.351					5.13E-04	0.007	0.939		
Asthma	MDD	asthma	Weighted median	154	0.216	0.084	0.011	0.019	14.192	68.618	1.351									
Asthma	MDD	asthma	Inverse variance weighte	154	0.213	0.063	6.94E-04	0.001	14.192	68.618	1.351	0.0093	40.626	0.459	unweighted				207.654	0.002
Asthma	MDD	asthma	Simple mode	154	0.309	0.267	0.250	0.429	14.192	68.618	1.351									
Asthma	MDD	asthma	Weighted mode	154	0.346	0.258	0.181	0.343	14.192	68.618	1.351									
Asthma	MDD	asthma	Steiger-filtered IVW	154	0.213	0.063	6.94E-04	0.001	14.192	68.618	1.351									
Asthma	MDD	asthma	GSMR	151	0.244	0.055	9.56E-06	1.81E-05	14.192	68.618	1.351									
Asthma	MDD	asthma	PRESSO	154	0.244	0.059	5.72E-05	9.78E-05	14.192	68.618	1.351									
Asthma	MDD	asthma	LHC		0.140	0.104	0.179	0.268	14.192	68.618	1.351									
Atrial Fibrillation	MDD	atrialfib	MR Egger	154	0.501	0.165	0.003	0.049	49.783	99.969	1.146					-0.009	0.004	0.017		
Atrial Fibrillation	MDD	atrialfib	Weighted median	154	0.132	0.039	8.18E-04	0.002	49.783	99.969	1.146									
Atrial Fibrillation	MDD	atrialfib	Inverse variance weighte	154	0.111	0.035	0.001	0.003	49.783	99.969	1.146	0.0093	40.626	0.459	unweighted				265.598	4.46E-08
Atrial Fibrillation	MDD	atrialfib	Simple mode	154	0.143	0.124	0.251	0.429	49.783	99.969	1.146									
Atrial Fibrillation	MDD	atrialfib	Weighted mode	154	0.135	0.121	0.268	0.458	49.783	99.969	1.146									
Atrial Fibrillation	MDD	atrialfib	Steiger-filtered IVW	154	0.111	0.035	0.001	0.003	49.783	99.969	1.146									
Atrial Fibrillation	MDD	atrialfib	GSMR	151	0.103	0.027	1.39E-04	2.34E-04	49.783	99.969	1.146									
Atrial Fibrillation	MDD	atrialfib	PRESSO	154	0.105	0.030	6.78E-04	0.001	49.783	99.969	1.146									
Atrial Fibrillation	MDD	atrialfib	LHC		0.026	0.027	0.331	0.464	49.783	99.969	1.146									
BMI	MDD	BMI	MR Egger	69	0.246	0.213	0.251	0.520	99.978	100.000	0.051					-0.003	0.005	0.506		
BMI	MDD	BMI	Weighted median	69	0.071	0.021	8.56E-04	0.002	99.978	100.000	0.051									
BMI	MDD	BMI	Inverse variance weighte	69	0.106	0.035	0.003	0.005	99.978	100.000	0.051	0.0042	40.604	0.113	unweighted				846.110	#######
BMI	MDD	BMI	Simple mode	69	0.052	0.047	0.278	0.458	99.978	100.000	0.051									
BMI	MDD	BMI	Weighted mode	69	0.040	0.037	0.283	0.467	99.978	100.000	0.051									
BMI	MDD	BMI	Steiger-filtered IVW	68	0.078	0.032	0.015	0.024	99.978	100.000	0.051									
BMI	MDD	BMI	GSMR	53	0.091	0.012	9.63E-14	2.68E-13	99.978	100.000	0.051									
BMI	MDD	BMI	PRESSO	69	0.116	0.021	1.37E-06	2.77E-06	99.978	100.000	0.051									
BMI	MDD	BMI	LHC		0.390	0.227	0.086	0.137	99.978	100.000	0.051									
Cholesterol	MDD	cholesterol	MR Egger	154	0.103	0.087	0.238	0.505	100.000	100.000	0.026					-0.002	0.002	0.353		
Cholesterol	MDD	cholesterol	Weighted median	154	0.018	0.009	0.042	0.066	100.000	100.000	0.026									
Cholesterol	MDD	cholesterol	Inverse variance weighte	154	0.024	0.018	0.188	0.254	100.000	100.000	0.026	0.0093	40.626	0.459	unweighted				1736.540	#######
Cholesterol	MDD	cholesterol	Simple mode	154	-0.003	0.029	0.920	0.975	100.000	100.000	0.026									
Cholesterol	MDD	cholesterol	Weighted mode	154	-0.003	0.028	0.917	0.972	100.000	100.000	0.026									
Cholesterol	MDD	cholesterol	Steiger-filtered IVW	153	0.035	0.013	0.005	0.009	100.000	100.000	0.026									
Cholesterol	MDD	cholesterol	GSMR	140	0.026	0.006	7.09E-06	1.37E-05	100.000	100.000	0.026									
Cholesterol	MDD	cholesterol	PRESSO	154	0.030	0.008	2.00E-04	3.17E-04	100.000	100.000	0.026									
Cholesterol	MDD	cholesterol	LHC		0.203	0.037	2.57E-08	1.06E-07	100.000	100.000	0.026									
Chronic obstructive pulmonary dis	MDD	copd	MR Egger	151	0.037	0.335	0.912	0.933	12.023	58.084	1.404					-0.002	0.008	0.812		
Chronic obstructive pulmonary dis	MDD	copd	Weighted median	151	0.108	0.097	0.264	0.324	12.023	58.084	1.404									
Chronic obstructive pulmonary dis	MDD	copd	Inverse variance weighte	151	-0.041	0.073	0.577	0.650	12.023	58.084	1.404	0.0091	40.711	0.461	unweighted				188.791	0.017
Chronic obstructive pulmonary dis	MDD	copd	Simple mode	151	0.398	0.305	0.194	0.375	12.023	58.084	1.404									

Chronic obstructive pulmonary dis	MDD	copd	Weighted mode	151	0.409	0.268	0.129	0.282	12.023	58.084	1.404									
Chronic obstructive pulmonary dis	MDD	copd	Steiger-filtered IVW	151	-0.041	0.073	0.577	0.659	12.023	58.084	1.404									
Chronic obstructive pulmonary dis	MDD	copd	GSMR	150	0.018	0.067	0.792	0.819	12.023	58.084	1.404									
Chronic obstructive pulmonary dis	MDD	copd	PRESSO	151	0.010	0.069	0.885	0.885	12.023	58.084	1.404									
Chronic obstructive pulmonary dis	MDD	copd	LHC		0.071	0.105	0.499	0.612	12.023	58.084	1.404									
Chronotype	MDD	chronotype	MR Egger	154	0.062	0.114	0.588	0.788	99.999	100.000	0.045					-0.002	0.003	0.470		
Chronotype	MDD	chronotype	Weighted median	154	-0.003	0.019	0.880	0.911	99.999	100.000	0.045									
Chronotype	MDD	chronotype	Inverse variance weighte	154	-0.019	0.025	0.448	0.532	99.999	100.000	0.045	0.0093	40.626	0.459	unweighted				838.079	5.56E-95
Chronotype	MDD	chronotype	Simple mode	154	0.022	0.056	0.701	0.857	99.999	100.000	0.045									
Chronotype	MDD	chronotype	Weighted mode	154	0.006	0.048	0.902	0.967	99.999	100.000	0.045									
Chronotype	MDD	chronotype	Steiger-filtered IVW	152	-0.003	0.022	0.890	0.911	99.999	100.000	0.045									
Chronotype	MDD	chronotype	GSMR	135	-0.008	0.012	0.517	0.569	99.999	100.000	0.045									
Chronotype	MDD	chronotype	PRESSO	154	-0.014	0.018	0.443	0.480	99.999	100.000	0.045									
Chronotype	MDD	chronotype	LHC		-0.358	0.197	0.068	0.112	99.999	100.000	0.045									
Cigarettes per day	MDD	cigperday	MR Egger	45	-0.081	0.139	0.562	0.788	98.384	100.000	0.068					0.005	0.003	0.108		
Cigarettes per day	MDD	cigperday	Weighted median	45	0.111	0.032	4.78E-04	0.001	98.384	100.000	0.068									
Cigarettes per day	MDD	cigperday	Inverse variance weighte	45	0.141	0.032	1.02E-05	2.32E-05	98.384	100.000	0.068	0.0028	42.298	0.599	weighted				157.117	1.28E-14
Cigarettes per day	MDD	cigperday	Simple mode	45	0.227	0.078	0.005	0.024	98.384	100.000	0.068									
Cigarettes per day	MDD	cigperday	Weighted mode	45	0.241	0.080	0.004	0.021	98.384	100.000	0.068									
Cigarettes per day	MDD	cigperday	Steiger-filtered IVW	45	0.141	0.032	1.02E-05	2.27E-05	98.384	100.000	0.068									
Cigarettes per day	MDD	cigperday	GSMR	43	0.125	0.018	5.54E-12	1.49E-11	98.384	100.000	0.068									
Cigarettes per day	MDD	cigperday	PRESSO	45	0.163	0.027	4.42E-07	9.92E-07	98.384	100.000	0.068									
Cigarettes per day	MDD	cigperday	LHC		0.282	0.110	0.010	0.021	98.384	100.000	0.068									
Coffee consumption	MDD	coffee	MR Egger	152	-0.415	0.273	0.131	0.333	18.501	83.340	1.285					0.008	0.006	0.185		
Coffee consumption	MDD	coffee	Weighted median	152	-0.108	0.077	0.161	0.214	18.501	83.340	1.285									
Coffee consumption	MDD	coffee	Inverse variance weighte	152	-0.060	0.060	0.317	0.387	18.501	83.340	1.285	0.0092	40.654	0.460	unweighted				221.481	1.64E-04
Coffee consumption	MDD	coffee	Simple mode	152	-0.312	0.244	0.202	0.375	18.501	83.340	1.285									
Coffee consumption	MDD	coffee	Weighted mode	152	-0.301	0.206	0.145	0.295	18.501	83.340	1.285									
Coffee consumption	MDD	coffee	Steiger-filtered IVW	152	-0.060	0.060	0.317	0.387	18.501	83.340	1.285									
Coffee consumption	MDD	coffee	GSMR	152	-0.057	0.050	0.251	0.294	18.501	83.340	1.285									
Coffee consumption	MDD	coffee	LHC		-0.133	0.059	0.024	0.043	18.501	83.340	1.285									
Coronary artery disease	MDD	coronaryartdis	MR Egger	153	0.276	0.162	0.090	0.257	89.340	100.000	1.087					0.000	0.004	0.970		
Coronary artery disease	MDD	coronaryartdis	Weighted median	153	0.281	0.032	7.15E-19	3.74E-18	89.340	100.000	1.087									
Coronary artery disease	MDD	coronaryartdis	Inverse variance weighte	153	0.270	0.033	4.38E-16	2.05E-15	89.340	100.000	1.087	0.0092	40.644	0.462	unweighted				433.154	7.96E-29
Coronary artery disease	MDD	coronaryartdis	Simple mode	153	0.309	0.090	7.78E-04	0.005	89.340	100.000	1.087									
Coronary artery disease	MDD	coronaryartdis	Weighted mode	153	0.309	0.082	2.51E-04	0.002	89.340	100.000	1.087									
Coronary artery disease	MDD	coronaryartdis	Steiger-filtered IVW	153	0.270	0.033	4.38E-16	2.05E-15	89.340	100.000	1.087									
Coronary artery disease	MDD	coronaryartdis	GSMR	150	0.260	0.021	1.81E-36	8.46E-36	89.340	100.000	1.087									
Coronary artery disease	MDD	coronaryartdis	PRESSO	153	0.278	0.028	4.50E-18	1.95E-17	89.340	100.000	1.087									
Coronary artery disease	MDD	coronaryartdis	LHC		0.192	0.030	1.06E-10	6.61E-10	89.340	100.000	1.087									
Crohn's disease	MDD	crohndis	MR Egger	146	0.455	0.437	0.300	0.568	6.562	23.604	1.808					-0.003	0.010	0.802		
Crohn's disease	MDD	crohndis	Weighted median	146	0.170	0.105	0.104	0.145	6.562	23.604	1.808									
Crohn's disease	MDD	crohndis	Inverse variance weighte	146	0.348	0.094	2.26E-04	4.57E-04	6.562	23.604	1.808	0.0088	40.800	0.480	unweighted				304.418	2.26E-13
Crohn's disease	MDD	crohndis	Simple mode	146	0.129	0.318	0.685	0.857	6.562	23.604	1.808									
Crohn's disease	MDD	crohndis	Weighted mode	146	0.077	0.291	0.792	0.904	6.562	23.604	1.808									
Crohn's disease	MDD	crohndis	Steiger-filtered IVW	141	0.294	0.082	3.56E-04	7.05E-04	6.562	23.604	1.808									
Crohn's disease	MDD	crohndis	GSMR	141	0.278	0.068	4.00E-05	7.12E-05	6.562	23.604	1.808									
Crohn's disease	MDD	crohndis	PRESSO	146	0.311	0.084	3.11E-04	4.81E-04	6.562	23.604	1.808									
Crohn's disease	MDD	crohndis	LHC		0.362	0.050	4.09E-13	3.55E-12	6.562	23.604	1.808									
CRP levels	MDD	crp	MR Egger	150	0.240	0.115	0.039	0.173	100.000	100.000	0.040					-0.004	0.003	0.141		
CRP levels	MDD	crp	Weighted median	150	0.078	0.014	5.33E-08	1.83E-07	100.000	100.000	0.040									
CRP levels	MDD	crp	Inverse variance weighte	150	0.073	0.022	0.001	0.002	100.000	100.000	0.040	0.0089	40.239	0.349	unweighted				1128.762	#######
CRP levels	MDD	crp	Simple mode	150	0.148	0.048	0.002	0.013	100.000	100.000	0.040									
CRP levels	MDD	crp	Weighted mode	150	0.065	0.040	0.105	0.260	100.000	100.000	0.040									
CRP levels	MDD	crp	Steiger-filtered IVW	148	0.091	0.016	2.68E-08	7.95E-08	100.000	100.000	0.040									
CRP levels	MDD	crp	GSMR	135	0.097	0.009	2.18E-28	8.09E-28	100.000	100.000	0.040									
CRP levels	MDD	crp	PRESSO	150	0.089	0.014	3.49E-09	8.40E-09	100.000	100.000	0.040									
CRP levels	MDD	crp	LHC		0.283	0.053	7.35E-08	2.91E-07	100.000	100.000	0.040									
Diastolic blood pressure	MDD	diabloodpres	MR Egger	81	0.469	1.063	0.660	0.810	89.535	100.000	0.087					-0.009	0.024	0.699		
Diastolic blood pressure	MDD	diabloodpres	Weighted median	81	0.231	0.150	0.124	0.167	89.535	100.000	0.087									
Diastolic blood pressure	MDD	diabloodpres	Inverse variance weighte	81	0.063	0.180	0.728	0.790	89.535	100.000	0.087	0.0048	40.098	0.164	unweighted				392.277	1.03E-42
Diastolic blood pressure	MDD	diabloodpres	Simple mode	81	0.531	0.380	0.166	0.375	89.535	100.000	0.087									
Diastolic blood pressure	MDD	diabloodpres	Weighted mode	81	0.457	0.311	0.146	0.295	89.535	100.000	0.087									
Diastolic blood pressure	MDD	diabloodpres	Steiger-filtered IVW	81	0.063	0.180	0.728	0.790	89.535	100.000	0.087									
Diastolic blood pressure	MDD	diabloodpres	GSMR	72	0.111	0.089	0.211	0.264	89.535	100.000	0.087									
Diastolic blood pressure	MDD	diabloodpres	PRESSO	81	0.078	0.142	0.582	0.610	89.535	100.000	0.087									
Diastolic blood pressure	MDD	diabloodpres	LHC		0.004	0.127	0.974	0.985	89.535	100.000	0.087									
Educational attainment	MDD	education	MR Egger	150	-0.266	0.111	0.018	0.112	100.000	100.000	0.018					0.002	0.003	0.383		
Educational attainment	MDD	education	Weighted median	150	-0.158	0.014	3.07E-31	3.04E-30	100.000	100.000	0.018									
Educational attainment	MDD	education	Inverse variance weighte	150	-0.171	0.022	1.29E-14	5.22E-14	100.000	100.000	0.018	0.0090	40.347	0.392	unweighted				1734.439	#######
Educational attainment	MDD	education	Simple mode	150	-0.172	0.035	2.15E-06	9.59E-05	100.000	100.000	0.018									
Educational attainment	MDD	education	Weighted mode	150	-0.172	0.032	4.36E-07	1.94E-05	100.000	100.000	0.018									
Educational attainment	MDD	education	Steiger-filtered IVW	148	-0.161	0.021	7.33E-14	2.89E-13	100.000	100.000	0.018									
Educational attainment	MDD	education	GSMR	114	-0.205	0.008	6.23E-131	2.77E-129	100.000	100.000	0.018									
Educational attainment	MDD	education	PRESSO	150	-0.186	0.014	1.01E-24	5.99E-24	100.000	100.000	0.018									
Educational attainment	MDD	education	LHC		-0.144	0.187	0.441	0.573	100.000	100.000	0.018									
Erectile dysfunction	MDD	erectile	MR Egger	152	0.597	0.367	0.106	0.291	10.349	48.465	1.466					-0.006	0.008	0.491		
Erectile dysfunction	MDD	erectile	Weighted median	152	0.323	0.118	0.006	0.011	10.349	48.465	1.466									
Erectile dysfunction	MDD	erectile	Inverse variance weighte	152	0.350	0.079	9.22E-06	2.16E-05	10.349	48.465	1.466	0.0092	40.701	0.463	unweighted				158.390	0.324
Erectile dysfunction	MDD	erectile	Simple mode	152	0.264	0.327	0.421	0.635	10.349	48.465	1.466									
Erectile dysfunction	MDD	erectile	Weighted mode	152	0.295	0.288	0.307	0.487	10.349	48.465	1.466									
Erectile dysfunction	MDD	erectile	Steiger-filtered IVW	152	0.350	0.079	9.22E-06	2.11E-05	10.349	48.465	1.466									
Erectile dysfunction	MDD	erectile	GSMR	152	0.337	0.078	1.58E-05	2.92E-05	10.349	48.465	1.466									
Erectile dysfunction	MDD	erectile	LHC		0.176	0.494	0.722	0.786	10.349	48.465	1.466									
Executive function	MDD	executivefun	MR Egger	149	-0.035	0.051	0.498	0.777	99.999	100.000	0.046					-0.001	0.001	0.629		
Executive function	MDD	executivefun	Weighted median	149	-0.043	0.008	3.16E-07	9.69E-07	99.999	100.000	0.046									
Executive function	MDD	executivefun	Inverse variance weighte	149	-0.059	0.010	1.31E-09	4.50E-09	99.999	100.000	0.046	0.0089	40.402	0.346	unweighted				676.571	1.50E-68
Executive function	MDD	executivefun	Simple mode	149	-0.037	0.029	0.200	0.375	99.999	100.000	0.046									
Executive function	MDD	executivefun	Weighted mode	149	-0.037	0.024	0.131	0.282	99.999	100.000	0.046									
Executive function	MDD	executivefun	Steiger-filtered IVW	148	-0.056	0.009	1.55E-09	4.75E-09	99.999	100.000	0.046									
Executive function	MDD	executivefun	GSMR	137	-0.049	0.005	5.00E-23	1.65E-22	99.999	100.000	0.046									
Executive function	MDD	executivefun	PRESSO	149	-0.057	0.008	1.60E-10	4.15E-10	99.999	100.000	0.046									
Executive function	MDD	executivefun	LHC		0.007	0.186	0.968	0.985	99.999	100.000	0.046									
Exercise (self report)	MDD	exercise	MR Egger	154	-0.035	0.115	0.759	0.877	91.799	100.000	1.083					-0.003	0.003	0.216		
Exercise (self report)	MDD	exercise	Weighted median	154	-0.165	0.024	6.40E-12	2.37E-11	91.799	100.000	1.083									
Exercise (self report)	MDD	exercise	Inverse variance weighte	154	-0.175	0.024	1.89E-13	7.01E-13	91.799	100.000	1.083	0.0093	40.626	0.459	unweighted				437.484	3.27E-29
Exercise (self report)	MDD	exercise	Simple mode	154	-0.155	0.072	0.033	0.103	91.799	100.000	1.083									
Exercise (self report)	MDD	exercise	Weighted mode	154	-0.159	0.061	0.010	0.042	91.799	100.000	1.083									
Exercise (self report)	MDD	exercise	Steiger-filtered IVW	154	-0.175	0.024	1.89E-13	7.01E-13	91.799	100.000	1.083									
Exercise (self report)	MDD	exercise	GSMR	145	-0.169	0.015	2.36E-29	9.14E-29	91.799	100.000	1.083									
Exercise (self report)	MDD	exercise	PRESSO	154	-0.192	0.021	8.84E-16	3.19E-15	91.799	100.000	1.083									
Exercise (self report)	MDD	exercise	LHC		-0.284	0.111	0.011	0.021	91.799	100.000	1.083									
Externalizing	MDD	externalizing	MR Egger	145	0.198	0.072	0.006	0.058	100.000	100.000	0.030					0.000	0.002	0.783		
Externalizing	MDD	externalizing	Weighted median	145	0.138	0.011	1.46E-34	1.63E-33	100.000	100.000	0.030									
Externalizing	MDD	externalizing	Inverse variance weighte	145	0.178	0.015	3.28E-32	2.24E-31	100.000	100.000	0.030	0.0088	41.046	0.440	unweighted				1065.024	#######
Externalizing	MDD	externalizing	Simple mode	145	0.150	0.036	5.50E-05	6.12E-04	100.000	100.000	0.030									
Externalizing	MDD	externalizing	Weighted mode	145	0.131	0.029	1.57E-05	2.33E-04	100.000	100.000	0.030									
Externalizing	MDD	externalizing	Steiger-filtered IVW	144	0.174	0.014	1.01E-33	7.53E-33	100.000	100.000	0.030									
Externalizing	MDD	externalizing	GSMR	125	0.144	0.007	1.58E-108	3.51E-107	100.000	100.000	0.030									
Externalizing	MDD	externalizing	PRESSO	145	0.168	0.011	3.08E-31	2.50E-30	100.000	100.000	0.030									
Externalizing	MDD	externalizing	LHC		0.633	0.106	2.41E-09	1.24E-08	100.000	100.000	0.030									
Financial difficulties	MDD	financialdiff	MR Egger	152	0.530	0.192	0.007	0.058	46.269	99.926	1.154					7.08E-04	0.004	0.873		
Financial difficulties	MDD	financialdiff	Weighted median	152	0.506	0.046	1.21E-27	9.79E-27	46.269	99.926	1.154									
Financial difficulties	MDD	financialdiff	Inverse variance weighte	152	0.560	0.042	2.39E-41	2.66E-40	46.269	99.926	1.154	0.0092	40.654	0.460	unweighted				366.804	5.50E-20
Financial difficulties	MDD	financialdiff	Simple mode	152	0.472	0.166	0.005	0.023	46.269	99.926	1.154									
Financial difficulties	MDD	financialdiff	Weighted mode	152	0.406	0.133	0.003	0.015	46.269	99.926	1.154									
Financial difficulties	MDD	financialdiff	Steiger-filtered IVW	152	0.560	0.042	2.39E-41	2.66E-40	46.269	99.926	1.154									
Financial difficulties	MDD	financialdiff	GSMR	149	0.540	0.028	1.17E-80	1.15E-79	46.269	99.926	1.154									
Financial difficulties	MDD	financialdiff	PRESSO	152	0.586	0.039	5.36E-32	4.98E-31	46.269	99.926	1.154									
Financial difficulties	MDD	financialdiff	LHC		0.379	0.118	0.001	0.003	46.269	99.926	1.154									
Fluid intelligence	MDD	fluidintelligence	MR Egger	152	-0.254	0.177	0.153	0.377	30.634	97.941	1.202					0.004	0.004	0.338		
Fluid intelligence	MDD	fluidintelligence	Weighted median	152	-0.101	0.050	0.043	0.066	30.634	97.941	1.202									
Fluid intelligence	MDD	fluidintelligence	Inverse variance weighte	152	-0.088	0.038	0.021	0.035	30.634	97.941	1.202	0.0092	40.654	0.460	unweighted				193.574	0.011
Fluid intelligence	MDD	fluidintelligence	Simple mode	152	-0.082	0.158	0.606	0.843	30.634	97.941	1.202									
Fluid intelligence	MDD	fluidintelligence	Weighted mode	152	-0.121	0.139	0.385	0.566	30.634	97.941	1.202									
Fluid intelligence	MDD	fluidintelligence	Steiger-filtered IVW	152	-0.088	0.038	0.021	0.034	30.634	97.941	1.202									
Fluid intelligence	MDD	fluidintelligence	GSMR	151	-0.093	0.034	0.007	0.011	30.634	97.941	1.202									
Fluid intelligence	MDD	fluidintelligence	PRESSO	152	-0.097	0.037	0.010	0.013	30.634	97.941	1.202									
Fluid intelligence	MDD	fluidintelligence	LHC		0.060	0.074	0.420	0.554	30.634	97.941	1.202									
Gastroesophageal reflux disease	MDD	gerd	MR Egger	151	0.719	0.142	1.17E-06	1.04E-04	28.084	96.695	1.214					-0.004	0.003	0.203		
Gastroesophageal reflux disease	MDD	gerd	Weighted median	151	0.524	0.037	2.34E-45	5.21E-44	28.084	96.695	1.214									
Gastroesophageal reflux disease	MDD	gerd	Inverse variance weighte	151	0.542	0.031	1.43E-68	4.24E-67	28.084	96.695	1.214	0.0091	40.711	0.461	unweighted				263.189	3.19E-08
Gastroesophageal reflux disease	MDD	gerd	Simple mode	151	0.549	0.123	1.46E-05	2.17E-04	28.084	96.695	1.214									
Gastroesophageal reflux disease	MDD	gerd	Weighted mode	151	0.522	0.132	1.14E-04	0.001	28.084	96.695	1.214									
Gastroesophageal reflux disease	MDD	gerd	Steiger-filtered IVW	151	0.542	0.031	1.43E-68	4.24E-67	28.084	96.695	1.214									
Gastroesophageal reflux disease	MDD	gerd	GSMR	151	0.505	0.025	7.77E-93	8.64E-92	28.084	96.695	1.214									
Gastroesophageal reflux disease	MDD	gerd	LHC		0.574	0.041	1.11E-43	2.42E-42	28.084	96.695	1.214									
Generalized epilepsy	MDD	epilepsy	MR Egger	120	0.190	0.373	0.611	0.788	8.818	38.723	1.552					-0.005	0.008	0.576		
Generalized epilepsy	MDD	epilepsy	Weighted median	120	-0.007	0.064	0.909	0.927	8.818	38.723	1.552									
Generalized epilepsy	MDD	epilepsy	Inverse variance weighte	120	-0.016	0.065	0.807	0.855	8.818	38.723	1.552	0.0072	40.388	0.205	unweighted				294.828	6.30E-17
Generalized epilepsy	MDD	epilepsy	Simple mode	120	0.012	0.190	0.951	0.984	8.818	38.723	1.552									
Generalized epilepsy	MDD	epilepsy	Weighted mode	120	-0.004	0.172	0.982	0.997	8.818	38.723	1.552									
Generalized epilepsy	MDD	epilepsy	Steiger-filtered IVW	115	-0.020	0.049	0.681	0.749	8.818	38.723	1.552									
Generalized epilepsy	MDD	epilepsy	GSMR	115	-0.022	0.043	0.606	0.650	8.818	38.723	1.552									
Generalized epilepsy	MDD	epilepsy	PRESSO	120	-0.020	0.049	0.682	0.693	8.818	38.723	1.552									
Generalized epilepsy	MDD	epilepsy	LHC		0.000	0.181	0.998	0.998	8.818	38.723	1.552									
Heart failure	MDD	heartfail	MR Egger	154	0.247	0.179	0.169	0.397	47.784	99.949	1.151					-0.001	0.004	0.758		
Heart failure	MDD	heartfail	Weighted median	154	0.142	0.047	0.003	0.005	47.784	99.949	1.151									
Heart failure	MDD	heartfail	Inverse variance weighte	154	0.193	0.037	2.32E-07	6.08E-07	47.784	99.949	1.151	0.0093	40.626	0.459	unweighted				219.537	3.40E-04

Heart failure	MDD	heartfail	Simple mode	154	0.142	0.156	0.363	0.557	47.784	99.949	1.151									
Heart failure	MDD	heartfail	Weighted mode	154	0.122	0.141	0.388	0.566	47.784	99.949	1.151									
Heart failure	MDD	heartfail	Steiger-filtered IVW	154	0.193	0.037	2.32E-07	6.08E-07	47.784	99.949	1.151									
Heart failure	MDD	heartfail	GSMR	154	0.181	0.032	1.28E-08	2.91E-08	47.784	99.949	1.151									
Heart failure	MDD	heartfail	LHC		0.090	0.042	0.034	0.060	47.784	99.949	1.151									
Heart rate variability	MDD	heartvaria	MR Egger	73	-0.039	0.184	0.834	0.884	18.083	88.166	0.267					0.002	0.004	0.659		
Heart rate variability	MDD	heartvaria	Weighted median	73	0.013	0.044	0.763	0.818	18.083	88.166	0.267									
Heart rate variability	MDD	heartvaria	Inverse variance weighte	73	0.041	0.037	0.259	0.329	18.083	88.166	0.267	0.0044	41.084	0.392	unweighted				112.764	0.002
Heart rate variability	MDD	heartvaria	Simple mode	73	-0.001	0.106	0.993	0.993	18.083	88.166	0.267									
Heart rate variability	MDD	heartvaria	Weighted mode	73	0.015	0.109	0.890	0.966	18.083	88.166	0.267									
Heart rate variability	MDD	heartvaria	Steiger-filtered IVW	72	0.024	0.032	0.449	0.526	18.083	88.166	0.267									
Heart rate variability	MDD	heartvaria	GSMR	72	0.021	0.030	0.478	0.545	18.083	88.166	0.267									
Heart rate variability	MDD	heartvaria	PRESSO	73	0.024	0.032	0.452	0.481	18.083	88.166	0.267									
Heart rate variability	MDD	heartvaria	LHC		0.173	0.386	0.655	0.721	18.083	88.166	0.267									
High density lipoprotein	MDD	hdl	MR Egger	154	-0.012	0.094	0.902	0.933	100.000	100.000	0.026					-0.002	0.002	0.327		
High density lipoprotein	MDD	hdl	Weighted median	154	-0.087	0.011	3.25E-16	1.52E-15	100.000	100.000	0.026									
High density lipoprotein	MDD	hdl	Inverse variance weighte	154	-0.102	0.019	1.50E-07	4.31E-07	100.000	100.000	0.026	0.0093	40.626	0.459	unweighted				1908.980	#######
High density lipoprotein	MDD	hdl	Simple mode	154	-0.115	0.028	8.58E-05	7.63E-04	100.000	100.000	0.026									
High density lipoprotein	MDD	hdl	Weighted mode	154	-0.097	0.027	5.43E-04	0.003	100.000	100.000	0.026									
High density lipoprotein	MDD	hdl	Steiger-filtered IVW	153	-0.090	0.014	1.39E-10	4.59E-10	100.000	100.000	0.026									
High density lipoprotein	MDD	hdl	GSMR	126	-0.079	0.006	9.19E-36	4.09E-35	100.000	100.000	0.026									
High density lipoprotein	MDD	hdl	PRESSO	154	-0.085	0.010	2.18E-14	6.75E-14	100.000	100.000	0.026									
High density lipoprotein	MDD	hdl	LHC		-0.290	0.035	1.73E-16	1.88E-15	100.000	100.000	0.026									
HIV susceptibility	MDD	hivsuscep	MR Egger	144	0.774	0.278	0.006	0.058	11.340	54.307	1.427					-0.014	0.006	0.027		
HIV susceptibility	MDD	hivsuscep	Weighted median	144	0.165	0.069	0.016	0.027	11.340	54.307	1.427									
HIV susceptibility	MDD	hivsuscep	Inverse variance weighte	144	0.166	0.053	0.002	0.003	11.340	54.307	1.427	0.0087	40.838	0.371	unweighted				195.990	0.002
HIV susceptibility	MDD	hivsuscep	Simple mode	144	0.258	0.192	0.182	0.375	11.340	54.307	1.427									
HIV susceptibility	MDD	hivsuscep	Weighted mode	144	0.245	0.181	0.177	0.343	11.340	54.307	1.427									
HIV susceptibility	MDD	hivsuscep	Steiger-filtered IVW	144	0.166	0.053	0.002	0.003	11.340	54.307	1.427									
HIV susceptibility	MDD	hivsuscep	GSMR	143	0.144	0.046	0.002	0.003	11.340	54.307	1.427									
HIV susceptibility	MDD	hivsuscep	PRESSO	144	0.151	0.050	0.003	0.004	11.340	54.307	1.427									
HIV susceptibility	MDD	hivsuscep	LHC		0.163	0.152	0.284	0.411	11.340	54.307	1.427									
Household income	MDD	income	MR Egger	148	-0.193	0.092	0.036	0.170	100.000	100.000	0.043					6.86E-04	0.002	0.742		
Household income	MDD	income	Weighted median	148	-0.163	0.015	2.34E-27	1.74E-26	100.000	100.000	0.043									
Household income	MDD	income	Inverse variance weighte	148	-0.164	0.018	2.30E-20	1.41E-19	100.000	100.000	0.043	0.0088	40.302	0.345	unweighted				686.196	1.57E-70
Household income	MDD	income	Simple mode	148	-0.194	0.049	1.16E-04	9.36E-04	100.000	100.000	0.043									
Household income	MDD	income	Weighted mode	148	-0.180	0.044	6.07E-05	6.76E-04	100.000	100.000	0.043									
Household income	MDD	income	Steiger-filtered IVW	148	-0.164	0.018	2.30E-20	1.41E-19	100.000	100.000	0.043									
Household income	MDD	income	GSMR	134	-0.165	0.009	1.08E-71	7.99E-71	100.000	100.000	0.043									
Household income	MDD	income	PRESSO	148	-0.165	0.015	1.36E-20	7.35E-20	100.000	100.000	0.043									
Household income	MDD	income	LHC		-0.019	0.430	0.966	0.985	100.000	100.000	0.043									
Hypothyroidism	MDD	hypothyroidism	MR Egger	145	0.434	0.222	0.052	0.204	44.439	99.886	1.158					-0.001	0.005	0.777		
Hypothyroidism	MDD	hypothyroidism	Weighted median	145	0.343	0.047	5.16E-13	2.09E-12	44.439	99.886	1.158									
Hypothyroidism	MDD	hypothyroidism	Inverse variance weighte	145	0.372	0.046	3.75E-16	1.85E-15	44.439	99.886	1.158	0.0088	40.792	0.476	unweighted				283.291	3.86E-11
Hypothyroidism	MDD	hypothyroidism	Simple mode	145	0.302	0.141	0.033	0.103	44.439	99.886	1.158									
Hypothyroidism	MDD	hypothyroidism	Weighted mode	145	0.314	0.121	0.010	0.042	44.439	99.886	1.158									
Hypothyroidism	MDD	hypothyroidism	Steiger-filtered IVW	144	0.356	0.042	2.81E-17	1.47E-16	44.439	99.886	1.158									
Hypothyroidism	MDD	hypothyroidism	GSMR	142	0.358	0.034	2.03E-26	6.96E-26	44.439	99.886	1.158									
Hypothyroidism	MDD	hypothyroidism	PRESSO	145	0.371	0.039	9.13E-17	3.49E-16	44.439	99.886	1.158									
Hypothyroidism	MDD	hypothyroidism	LHC		0.156	0.036	1.15E-05	3.22E-05	44.439	99.886	1.158									
IL6 levels	MDD	il6	MR Egger	71	-0.165	0.131	0.214	0.463	31.486	99.331	0.190					0.005	0.003	0.113		
IL6 levels	MDD	il6	Weighted median	71	0.009	0.039	0.826	0.865	31.486	99.331	0.190									
IL6 levels	MDD	il6	Inverse variance weighte	71	0.042	0.027	0.121	0.169	31.486	99.331	0.190	0.0043	41.271	0.399	unweighted				72.427	0.398
IL6 levels	MDD	il6	Simple mode	71	-0.028	0.094	0.771	0.902	31.486	99.331	0.190									
IL6 levels	MDD	il6	Weighted mode	71	-0.024	0.086	0.777	0.904	31.486	99.331	0.190									
IL6 levels	MDD	il6	Steiger-filtered IVW	71	0.042	0.027	0.121	0.161	31.486	99.331	0.190									
IL6 levels	MDD	il6	GSMR	71	0.038	0.027	0.154	0.198	31.486	99.331	0.190									
IL6 levels	MDD	il6	LHC		0.310	0.101	0.002	0.005	31.486	99.331	0.190									
Inflammatory bowel disease	MDD	ibd	MR Egger	146	0.357	0.333	0.286	0.553	10.781	51.063	1.448					-0.005	0.008	0.541		
Inflammatory bowel disease	MDD	ibd	Weighted median	146	0.153	0.077	0.049	0.073	10.781	51.063	1.448									
Inflammatory bowel disease	MDD	ibd	Inverse variance weighte	146	0.158	0.072	0.028	0.045	10.781	51.063	1.448	0.0088	40.800	0.480	unweighted				295.373	2.47E-12
Inflammatory bowel disease	MDD	ibd	Simple mode	146	0.235	0.222	0.292	0.472	10.781	51.063	1.448									
Inflammatory bowel disease	MDD	ibd	Weighted mode	146	0.196	0.230	0.396	0.568	10.781	51.063	1.448									
Inflammatory bowel disease	MDD	ibd	Steiger-filtered IVW	144	0.122	0.069	0.076	0.109	10.781	51.063	1.448									
Inflammatory bowel disease	MDD	ibd	GSMR	143	0.127	0.052	0.014	0.021	10.781	51.063	1.448									
Inflammatory bowel disease	MDD	ibd	PRESSO	146	0.137	0.067	0.044	0.057	10.781	51.063	1.448									
Inflammatory bowel disease	MDD	ibd	LHC		0.478	0.082	6.16E-09	2.98E-08	10.781	51.063	1.448									
Insomnia	MDD	insomnia	MR Egger	154	0.294	0.135	0.031	0.151	49.585	99.967	1.147					0.001	0.003	0.693		
Insomnia	MDD	insomnia	Weighted median	154	0.312	0.032	7.49E-22	4.17E-21	49.585	99.967	1.147									
Insomnia	MDD	insomnia	Inverse variance weighte	154	0.346	0.029	1.21E-32	8.99E-32	49.585	99.967	1.147	0.0093	40.626	0.459	unweighted				319.751	7.79E-14
Insomnia	MDD	insomnia	Simple mode	154	0.399	0.119	9.65E-04	0.006	49.585	99.967	1.147									
Insomnia	MDD	insomnia	Weighted mode	154	0.240	0.104	0.022	0.075	49.585	99.967	1.147									
Insomnia	MDD	insomnia	Steiger-filtered IVW	154	0.346	0.029	1.21E-32	8.30E-32	49.585	99.967	1.147									
Insomnia	MDD	insomnia	GSMR	151	0.315	0.021	2.08E-50	1.23E-49	49.585	99.967	1.147									
Insomnia	MDD	insomnia	PRESSO	154	0.345	0.027	3.56E-25	2.32E-24	49.585	99.967	1.147									
Insomnia	MDD	insomnia	LHC		0.417	0.054	1.40E-14	1.35E-13	49.585	99.967	1.147									
Irritable bowel disease	MDD	irbd	MR Egger	153	0.610	0.331	0.067	0.215	13.727	66.549	1.361					-0.003	0.008	0.718		
Irritable bowel disease	MDD	irbd	Weighted median	153	0.396	0.087	5.06E-06	1.36E-05	13.727	66.549	1.361									
Irritable bowel disease	MDD	irbd	Inverse variance weighte	153	0.493	0.066	7.47E-14	2.89E-13	13.727	66.549	1.361	0.0091	40.260	0.385	unweighted				191.689	0.016
Irritable bowel disease	MDD	irbd	Simple mode	153	0.003	0.274	0.990	0.993	13.727	66.549	1.361									
Irritable bowel disease	MDD	irbd	Weighted mode	153	0.003	0.260	0.990	0.997	13.727	66.549	1.361									
Irritable bowel disease	MDD	irbd	Steiger-filtered IVW	153	0.493	0.066	7.47E-14	2.89E-13	13.727	66.549	1.361									
Irritable bowel disease	MDD	irbd	GSMR	152	0.477	0.060	1.65E-15	4.73E-15	13.727	66.549	1.361									
Irritable bowel disease	MDD	irbd	PRESSO	153	0.508	0.064	3.70E-13	1.05E-12	13.727	66.549	1.361									
Irritable bowel disease	MDD	irbd	LHC		0.214	0.127	0.091	0.141	13.727	66.549	1.361									
Living with partner	MDD	partner	MR Egger	152	-0.064	0.125	0.610	0.788	85.897	100.000	1.092					-0.001	0.003	0.706		
Living with partner	MDD	partner	Weighted median	152	-0.115	0.031	1.87E-04	4.15E-04	85.897	100.000	1.092									
Living with partner	MDD	partner	Inverse variance weighte	152	-0.110	0.027	4.80E-05	1.02E-04	85.897	100.000	1.092	0.0092	40.654	0.460	unweighted				302.764	3.19E-12
Living with partner	MDD	partner	Simple mode	152	-0.124	0.095	0.195	0.375	85.897	100.000	1.092									
Living with partner	MDD	partner	Weighted mode	152	-0.119	0.090	0.190	0.351	85.897	100.000	1.092									
Living with partner	MDD	partner	Steiger-filtered IVW	152	-0.110	0.027	4.80E-05	9.94E-05	85.897	100.000	1.092									
Living with partner	MDD	partner	GSMR	149	-0.097	0.020	8.49E-07	1.68E-06	85.897	100.000	1.092									
Living with partner	MDD	partner	PRESSO	152	-0.105	0.025	4.26E-05	7.48E-05	85.897	100.000	1.092									
Living with partner	MDD	partner	LHC		-0.217	0.237	0.360	0.489	85.897	100.000	1.092									
Loneliness	MDD	loneliness	MR Egger	154	0.094	0.020	3.99E-06	1.78E-04	59.562	99.998	1.129					0.000	######	0.415		
Loneliness	MDD	loneliness	Weighted median	154	0.067	0.005	1.49E-37	2.65E-36	59.562	99.998	1.129									
Loneliness	MDD	loneliness	Inverse variance weighte	154	0.078	0.004	3.29E-75	1.47E-73	59.562	99.998	1.129	0.0093	40.626	0.459	unweighted				273.413	7.72E-09
Loneliness	MDD	loneliness	Simple mode	154	0.056	0.019	0.003	0.014	59.562	99.998	1.129									
Loneliness	MDD	loneliness	Weighted mode	154	0.051	0.017	0.003	0.017	59.562	99.998	1.129									
Loneliness	MDD	loneliness	Steiger-filtered IVW	154	0.078	0.004	3.29E-75	1.47E-73	59.562	99.998	1.129									
Loneliness	MDD	loneliness	GSMR	152	0.074	0.003	7.61E-107	1.35E-105	59.562	99.998	1.129									
Loneliness	MDD	loneliness	PRESSO	154	0.078	0.004	2.28E-42	7.41E-41	59.562	99.998	1.129									
Loneliness	MDD	loneliness	LHC		0.469	0.040	2.37E-31	4.12E-30	59.562	99.998	1.129									
Long illness or disability	MDD	disability	MR Egger	154	0.258	0.132	0.053	0.204	67.169	100.000	1.117					0.003	0.003	0.274		
Long illness or disability	MDD	disability	Weighted median	154	0.365	0.031	3.57E-31	3.18E-30	67.169	100.000	1.117									
Long illness or disability	MDD	disability	Inverse variance weighte	154	0.400	0.029	3.77E-44	4.79E-43	67.169	100.000	1.117	0.0093	40.626	0.459	unweighted				321.797	4.51E-14
Long illness or disability	MDD	disability	Simple mode	154	0.198	0.102	0.054	0.142	67.169	100.000	1.117									
Long illness or disability	MDD	disability	Weighted mode	154	0.192	0.093	0.041	0.119	67.169	100.000	1.117									
Long illness or disability	MDD	disability	Steiger-filtered IVW	154	0.400	0.029	3.77E-44	4.79E-43	67.169	100.000	1.117									
Long illness or disability	MDD	disability	GSMR	151	0.399	0.021	1.87E-79	1.66E-78	67.169	100.000	1.117									
Long illness or disability	MDD	disability	PRESSO	154	0.418	0.026	8.91E-35	9.65E-34	67.169	100.000	1.117									
Long illness or disability	MDD	disability	LHC		0.373	0.070	1.04E-07	3.92E-07	67.169	100.000	1.117									
Low density lipoprotein	MDD	ldl	MR Egger	153	0.064	0.088	0.468	0.746	100.000	100.000	0.026					-0.001	0.002	0.648		
Low density lipoprotein	MDD	ldl	Weighted median	153	0.033	0.009	1.63E-04	3.72E-04	100.000	100.000	0.026									
Low density lipoprotein	MDD	ldl	Inverse variance weighte	153	0.024	0.018	0.178	0.243	100.000	100.000	0.026	0.0092	40.644	0.462	unweighted				1616.019	#######
Low density lipoprotein	MDD	ldl	Simple mode	153	0.018	0.026	0.505	0.725	100.000	100.000	0.026									
Low density lipoprotein	MDD	ldl	Weighted mode	153	0.024	0.024	0.321	0.501	100.000	100.000	0.026									
Low density lipoprotein	MDD	ldl	Steiger-filtered IVW	152	0.036	0.012	0.003	0.005	100.000	100.000	0.026									
Low density lipoprotein	MDD	ldl	GSMR	140	0.033	0.006	2.06E-08	4.48E-08	100.000	100.000	0.026									
Low density lipoprotein	MDD	ldl	PRESSO	153	0.034	0.008	1.24E-05	2.30E-05	100.000	100.000	0.026									
Low density lipoprotein	MDD	ldl	LHC		0.049	0.028	0.085	0.137	100.000	100.000	0.026									
Malignant neoplasm of breast	MDD	cancerbreast	MR Egger	154	-0.144	0.332	0.664	0.810	11.318	54.182	1.428					0.001	0.008	0.872		
Malignant neoplasm of breast	MDD	cancerbreast	Weighted median	154	-0.054	0.098	0.579	0.660	11.318	54.182	1.428									
Malignant neoplasm of breast	MDD	cancerbreast	Inverse variance weighte	154	-0.092	0.072	0.200	0.262	11.318	54.182	1.428	0.0093	40.626	0.459	unweighted				173.300	0.125
Malignant neoplasm of breast	MDD	cancerbreast	Simple mode	154	-0.127	0.313	0.686	0.857	11.318	54.182	1.428									
Malignant neoplasm of breast	MDD	cancerbreast	Weighted mode	154	-0.080	0.286	0.781	0.904	11.318	54.182	1.428									
Malignant neoplasm of breast	MDD	cancerbreast	Steiger-filtered IVW	154	-0.092	0.072	0.200	0.254	11.318	54.182	1.428									
Malignant neoplasm of breast	MDD	cancerbreast	GSMR	154	-0.084	0.068	0.222	0.267	11.318	54.182	1.428									
Malignant neoplasm of breast	MDD	cancerbreast	LHC		-0.122	0.149	0.412	0.552	11.318	54.182	1.428									
Malignant neoplasms of skin	MDD	cancerskin	MR Egger	154	-0.067	0.280	0.810	0.884	12.933	62.774	1.379					4.15E-05	0.006	0.995		
Malignant neoplasms of skin	MDD	cancerskin	Weighted median	154	-0.195	0.079	0.014	0.024	12.933	62.774	1.379									
Malignant neoplasms of skin	MDD	cancerskin	Inverse variance weighte	154	-0.066	0.061	0.278	0.344	12.933	62.774	1.379	0.0093	40.626	0.459	unweighted				191.409	0.019
Malignant neoplasms of skin	MDD	cancerskin	Simple mode	154	-0.420	0.222	0.060	0.152	12.933	62.774	1.379									
Malignant neoplasms of skin	MDD	cancerskin	Weighted mode	154	-0.370	0.220	0.094	0.248	12.933	62.774	1.379									
Malignant neoplasms of skin	MDD	cancerskin	Steiger-filtered IVW	153	-0.087	0.058	0.134	0.175	12.933	62.774	1.379									
Malignant neoplasms of skin	MDD	cancerskin	GSMR	153	-0.087	0.055	0.114	0.149	12.933	62.774	1.379									
Malignant neoplasms of skin	MDD	cancerskin	PRESSO	154	-0.087	0.058	0.136	0.164	12.933	62.774	1.379									
Malignant neoplasms of skin	MDD	cancerskin	LHC		-0.288	0.103	0.005	0.012	12.933	62.774	1.379									
Management demands	MDD	manager	MR Egger	151	0.109	0.087	0.212	0.463	99.295	100.000	0.063					-0.003	0.002	0.181		
Management demands	MDD	manager	Weighted median	151	-0.035	0.020	0.088	0.124	99.295	100.000	0.063									
Management demands	MDD	manager	Inverse variance weighte	151	-0.005	0.018	0.769	0.825	99.295	100.000	0.063	0.0091	40.711	0.461	unweighted				349.580	5.66E-18
Management demands	MDD	manager	Simple mode	151	-0.072	0.069	0.297	0.472	99.295	100.000	0.063									
Management demands	MDD	manager	Weighted mode	151	-0.065	0.063	0.300	0.485	99.295	100.000	0.063									
Management demands	MDD	manager	Steiger-filtered IVW	151	-0.005	0.018	0.769	0.825	99.295	100.000	0.063									
Management demands	MDD	manager	GSMR	146	-0.017	0.013	0.167	0.212	99.295	100.000	0.063									
Management demands	MDD	manager	PRESSO	151	-0.015	0.017	0.376	0.414	99.295	100.000	0.063									
Management demands	MDD	manager	LHC		0.038	0.053	0.477	0.602	99.295	100.000	0.063									
Maternal smoking	MDD	smokingmaternal	MR Egger	154	0.283	0.148	0.058	0.215	60.338	99.998	1.128					3.01E-04	0.003	0.930		
Maternal smoking	MDD	smokingmaternal	Weighted median	154	0.267	0.037	3.86E-13	1.64E-12	60.338	99.998	1.128									

Maternal smoking	MDD	smokingmaternal	Inverse variance weighted	154	0.295	0.032	2.38E-20	1.41E-19	60.338	99.998	1.128	0.0093	40.626	0.459	unweighted				346.018	5.61E-17
Maternal smoking	MDD	smokingmaternal	Simple mode	154	0.279	0.113	0.014	0.054	60.338	99.998	1.128									
Maternal smoking	MDD	smokingmaternal	Weighted mode	154	0.273	0.111	0.015	0.056	60.338	99.998	1.128									
Maternal smoking	MDD	smokingmaternal	Steiger-filtered IVW	154	0.295	0.032	2.38E-20	1.41E-19	60.338	99.998	1.128									
Maternal smoking	MDD	smokingmaternal	GSMR	152	0.271	0.022	2.36E-34	9.57E-34	60.338	99.998	1.128									
Maternal smoking	MDD	smokingmaternal	PRESSO	154	0.294	0.031	3.62E-17	1.47E-16	60.338	99.998	1.128									
Maternal smoking	MDD	smokingmaternal	LHC		0.414	0.202	0.040	0.069	60.338	99.998	1.128									
Medication and treatment use	MDD	treatmentuse	MR Egger	154	0.429	0.195	0.029	0.151	97.126	100.000	0.073					0.002	0.004	0.606		
Medication and treatment use	MDD	treatmentuse	Weighted median	154	0.554	0.039	1.15E-45	3.42E-44	97.126	100.000	0.073									
Medication and treatment use	MDD	treatmentuse	Inverse variance weighted	154	0.528	0.042	5.04E-36	4.49E-35	97.126	100.000	0.073	0.0093	40.626	0.459	unweighted				525.908	2.06E-42
Medication and treatment use	MDD	treatmentuse	Simple mode	154	0.662	0.103	1.61E-09	1.44E-07	97.126	100.000	0.073									
Medication and treatment use	MDD	treatmentuse	Weighted mode	154	0.643	0.106	1.07E-08	9.48E-07	97.126	100.000	0.073									
Medication and treatment use	MDD	treatmentuse	Steiger-filtered IVW	153	0.519	0.041	1.74E-36	1.54E-35	97.126	100.000	0.073									
Medication and treatment use	MDD	treatmentuse	GSMR	143	0.538	0.025	9.61E-102	1.22E-100	97.126	100.000	0.073									
Medication and treatment use	MDD	treatmentuse	PRESSO	154	0.582	0.034	3.96E-36	5.15E-35	97.126	100.000	0.073									
Medication and treatment use	MDD	treatmentuse	LHC		0.500	0.048	3.87E-25	4.81E-24	97.126	100.000	0.073									
Metabolic syndrome	MDD	metabolicsyn	MR Egger	153	-0.039	0.221	0.859	0.900	98.329	100.000	0.069					0.008	0.005	0.137		
Metabolic syndrome	MDD	metabolicsyn	Weighted median	153	0.235	0.044	9.68E-08	3.19E-07	98.329	100.000	0.069									
Metabolic syndrome	MDD	metabolicsyn	Inverse variance weighted	153	0.283	0.048	4.41E-09	1.46E-08	98.329	100.000	0.069	0.0092	40.635	0.457	unweighted				532.318	1.10E-43
Metabolic syndrome	MDD	metabolicsyn	Simple mode	153	0.256	0.129	0.050	0.134	98.329	100.000	0.069									
Metabolic syndrome	MDD	metabolicsyn	Weighted mode	153	0.193	0.123	0.118	0.276	98.329	100.000	0.069									
Metabolic syndrome	MDD	metabolicsyn	Steiger-filtered IVW	152	0.263	0.043	8.77E-10	2.79E-09	98.329	100.000	0.069									
Metabolic syndrome	MDD	metabolicsyn	GSMR	149	0.231	0.027	9.74E-18	2.99E-17	98.329	100.000	0.069									
Metabolic syndrome	MDD	metabolicsyn	PRESSO	153	0.255	0.040	2.03E-09	5.07E-09	98.329	100.000	0.069									
Metabolic syndrome	MDD	metabolicsyn	LHC		0.267	0.089	0.003	0.006	98.329	100.000	0.069									
Multiple sclerosis	MDD	ms	MR Egger	150	-0.784	0.422	0.066	0.215	8.002	33.286	1.618					0.022	0.010	0.021		
Multiple sclerosis	MDD	ms	Weighted median	150	0.120	0.103	0.246	0.309	8.002	33.286	1.618									
Multiple sclerosis	MDD	ms	Inverse variance weighted	150	0.183	0.084	0.030	0.047	8.002	33.286	1.618	0.0091	40.774	0.864	weighted				237.687	5.22E-06
Multiple sclerosis	MDD	ms	Simple mode	150	0.060	0.305	0.843	0.958	8.002	33.286	1.618									
Multiple sclerosis	MDD	ms	Weighted mode	150	0.080	0.280	0.776	0.904	8.002	33.286	1.618									
Multiple sclerosis	MDD	ms	Steiger-filtered IVW	148	0.115	0.073	0.118	0.158	8.002	33.286	1.618									
Multiple sclerosis	MDD	ms	MR Egger SIMEX		-1.239	0.605	0.042	0.042	8.002	33.286	1.618									
Multiple sclerosis	MDD	ms	GSMR	148	0.110	0.068	0.108	0.143	8.002	33.286	1.618									
Multiple sclerosis	MDD	ms	PRESSO	150	0.158	0.070	0.026	0.035	8.002	33.286	1.618									
Multisite chronic pain	MDD	chronicpain	MR Egger	153	0.174	0.074	0.019	0.114	99.992	100.000	0.049					0.002	0.002	0.252		
Multisite chronic pain	MDD	chronicpain	Weighted median	153	0.252	0.017	3.60E-49	1.60E-47	99.992	100.000	0.049									
Multisite chronic pain	MDD	chronicpain	Inverse variance weighted	153	0.257	0.016	8.00E-58	1.42E-56	99.992	100.000	0.049	0.0092	40.635	0.457	unweighted				423.569	1.81E-27
Multisite chronic pain	MDD	chronicpain	Simple mode	153	0.290	0.064	1.12E-05	1.99E-04	99.992	100.000	0.049									
Multisite chronic pain	MDD	chronicpain	Weighted mode	153	0.276	0.058	3.69E-06	6.56E-05	99.992	100.000	0.049									
Multisite chronic pain	MDD	chronicpain	Steiger-filtered IVW	153	0.257	0.016	8.00E-58	1.42E-56	99.992	100.000	0.049									
Multisite chronic pain	MDD	chronicpain	GSMR	147	0.250	0.011	2.16E-125	6.42E-124	99.992	100.000	0.049									
Multisite chronic pain	MDD	chronicpain	PRESSO	153	0.260	0.015	1.43E-37	2.32E-36	99.992	100.000	0.049									
Multisite chronic pain	MDD	chronicpain	LHC		0.437	0.910	0.631	0.710	99.992	100.000	0.049									
Number of children	MDD	numberchildren	MR Egger	154	0.086	0.093	0.354	0.618	99.051	100.000	0.065					1.87E-04	0.002	0.930		
Number of children	MDD	numberchildren	Weighted median	154	0.114	0.022	2.39E-07	7.58E-07	99.051	100.000	0.065									
Number of children	MDD	numberchildren	Inverse variance weighted	154	0.094	0.020	2.59E-06	6.41E-06	99.051	100.000	0.065	0.0093	40.626	0.459	unweighted				308.254	1.59E-12
Number of children	MDD	numberchildren	Simple mode	154	0.164	0.071	0.023	0.079	99.051	100.000	0.065									
Number of children	MDD	numberchildren	Weighted mode	154	0.154	0.063	0.015	0.056	99.051	100.000	0.065									
Number of children	MDD	numberchildren	Steiger-filtered IVW	153	0.085	0.019	5.36E-06	1.29E-05	99.051	100.000	0.065									
Number of children	MDD	numberchildren	GSMR	152	0.083	0.014	8.19E-09	1.92E-08	99.051	100.000	0.065									
Number of children	MDD	numberchildren	PRESSO	154	0.086	0.018	2.19E-06	4.31E-06	99.051	100.000	0.065									
Number of children	MDD	numberchildren	LHC		0.144	0.030	1.46E-06	4.71E-06	99.051	100.000	0.065									
Oxygen uptake	MDD	oxyuptake	MR Egger	148	-0.066	0.110	0.550	0.788	67.632	100.000	0.116					3.45E-05	0.003	0.989		
Oxygen uptake	MDD	oxyuptake	Weighted median	148	-0.059	0.030	0.050	0.074	67.632	100.000	0.116									
Oxygen uptake	MDD	oxyuptake	Inverse variance weighted	148	-0.064	0.024	0.007	0.013	67.632	100.000	0.116	0.0089	40.413	0.466	unweighted				239.171	2.35E-06
Oxygen uptake	MDD	oxyuptake	Simple mode	148	-0.043	0.092	0.639	0.857	67.632	100.000	0.116									
Oxygen uptake	MDD	oxyuptake	Weighted mode	148	-0.031	0.079	0.699	0.889	67.632	100.000	0.116									
Oxygen uptake	MDD	oxyuptake	Steiger-filtered IVW	148	-0.064	0.024	0.007	0.012	67.632	100.000	0.116									
Oxygen uptake	MDD	oxyuptake	GSMR	146	-0.062	0.019	0.001	0.002	67.632	100.000	0.116									
Oxygen uptake	MDD	oxyuptake	LHC		-0.091	1.944	0.962	0.985	67.632	100.000	0.116									
Parental longevity	MDD	parentlongevity	MR Egger	147	0.040	0.161	0.802	0.884	44.593	99.978	0.154					-0.002	0.004	0.601		
Parental longevity	MDD	parentlongevity	Weighted median	147	0.013	0.045	0.782	0.828	44.593	99.978	0.154									
Parental longevity	MDD	parentlongevity	Inverse variance weighted	147	-0.042	0.034	0.223	0.288	44.593	99.978	0.154	0.0088	40.486	0.438	unweighted				187.979	0.011
Parental longevity	MDD	parentlongevity	Simple mode	147	0.093	0.134	0.490	0.715	44.593	99.978	0.154									
Parental longevity	MDD	parentlongevity	Weighted mode	147	0.085	0.125	0.497	0.670	44.593	99.978	0.154									
Parental longevity	MDD	parentlongevity	Steiger-filtered IVW	146	-0.032	0.033	0.332	0.399	44.593	99.978	0.154									
Parental longevity	MDD	parentlongevity	GSMR	145	-0.020	0.031	0.515	0.569	44.593	99.978	0.154									
Parental longevity	MDD	parentlongevity	PRESSO	147	-0.032	0.033	0.333	0.380	44.593	99.978	0.154									
Parental longevity	MDD	parentlongevity	LHC		-0.238	0.082	0.004	0.008	44.593	99.978	0.154									
Parental mortality	MDD	parentlife	MR Egger	151	0.106	0.066	0.111	0.291	99.996	100.000	0.048					-0.001	0.002	0.475		
Parental mortality	MDD	parentlife	Weighted median	151	0.063	0.014	5.62E-06	1.47E-05	99.996	100.000	0.048									
Parental mortality	MDD	parentlife	Inverse variance weighted	151	0.060	0.013	6.84E-06	1.64E-05	99.996	100.000	0.048	0.0090	40.343	0.388	unweighted				328.161	2.47E-15
Parental mortality	MDD	parentlife	Simple mode	151	0.058	0.044	0.193	0.375	99.996	100.000	0.048									
Parental mortality	MDD	parentlife	Weighted mode	151	0.066	0.039	0.096	0.248	99.996	100.000	0.048									
Parental mortality	MDD	parentlife	Steiger-filtered IVW	151	0.060	0.013	6.84E-06	1.60E-05	99.996	100.000	0.048									
Parental mortality	MDD	parentlife	GSMR	146	0.054	0.009	4.89E-09	1.18E-08	99.996	100.000	0.048									
Parental mortality	MDD	parentlife	PRESSO	151	0.055	0.012	7.32E-06	1.40E-05	99.996	100.000	0.048									
Parental mortality	MDD	parentlife	LHC		0.191	0.040	1.93E-06	6.00E-06	99.996	100.000	0.048									
Parkinson disease	MDD	parkinson	MR Egger	55	-0.344	0.655	0.602	0.788	4.730	12.175	2.525					0.007	0.015	0.655		
Parkinson disease	MDD	parkinson	Weighted median	55	-0.021	0.198	0.917	0.927	4.730	12.175	2.525									
Parkinson disease	MDD	parkinson	Inverse variance weighted	55	-0.056	0.136	0.681	0.748	4.730	12.175	2.525	0.0035	42.628	0.453	unweighted				50.680	0.603
Parkinson disease	MDD	parkinson	Simple mode	55	-0.151	0.464	0.746	0.897	4.730	12.175	2.525									
Parkinson disease	MDD	parkinson	Weighted mode	55	0.014	0.448	0.975	0.997	4.730	12.175	2.525									
Parkinson disease	MDD	parkinson	Steiger-filtered IVW	55	-0.056	0.136	0.681	0.749	4.730	12.175	2.525									
Parkinson disease	MDD	parkinson	GSMR	55	-0.054	0.137	0.693	0.725	4.730	12.175	2.525									
Parkinson disease	MDD	parkinson	LHC		0.394	0.428	0.358	0.489	4.730	12.175	2.525									
Pelvic organ prolapse	MDD	pelvicorganprol	MR Egger	151	0.189	0.209	0.365	0.625	30.533	97.902	1.202					9.81E-04	0.005	0.838		
Pelvic organ prolapse	MDD	pelvicorganprol	Weighted median	151	0.212	0.056	1.54E-04	3.61E-04	30.533	97.902	1.202									
Pelvic organ prolapse	MDD	pelvicorganprol	Inverse variance weighted	151	0.231	0.045	2.22E-07	5.99E-07	30.533	97.902	1.202	0.0091	40.545	0.464	unweighted				211.475	7.06E-04
Pelvic organ prolapse	MDD	pelvicorganprol	Simple mode	151	0.226	0.165	0.173	0.375	30.533	97.902	1.202									
Pelvic organ prolapse	MDD	pelvicorganprol	Weighted mode	151	0.234	0.167	0.163	0.323	30.533	97.902	1.202									
Pelvic organ prolapse	MDD	pelvicorganprol	Steiger-filtered IVW	151	0.231	0.045	2.22E-07	5.99E-07	30.533	97.902	1.202									
Pelvic organ prolapse	MDD	pelvicorganprol	GSMR	151	0.215	0.038	1.92E-08	4.28E-08	30.533	97.902	1.202									
Pelvic organ prolapse	MDD	pelvicorganprol	LHC		0.158	0.065	0.016	0.030	30.533	97.902	1.202									
Peripheral artery disease	MDD	peripheralartdis	MR Egger	154	0.592	0.331	0.076	0.233	16.439	77.249	1.312					-0.007	0.008	0.323		
Peripheral artery disease	MDD	peripheralartdis	Weighted median	154	0.335	0.097	5.17E-04	0.001	16.439	77.249	1.312									
Peripheral artery disease	MDD	peripheralartdis	Inverse variance weighted	154	0.271	0.069	9.10E-05	1.88E-04	16.439	77.249	1.312	0.0093	40.626	0.511	weighted				183.749	0.046
Peripheral artery disease	MDD	peripheralartdis	Simple mode	154	0.381	0.292	0.194	0.375	16.439	77.249	1.312									
Peripheral artery disease	MDD	peripheralartdis	Weighted mode	154	0.314	0.252	0.214	0.374	16.439	77.249	1.312									
Peripheral artery disease	MDD	peripheralartdis	Steiger-filtered IVW	154	0.271	0.069	9.10E-05	1.84E-04	16.439	77.249	1.312									
Peripheral artery disease	MDD	peripheralartdis	GSMR	154	0.260	0.064	5.00E-05	8.57E-05	16.439	77.249	1.312									
Peripheral artery disease	MDD	peripheralartdis	LHC		0.133	0.030	8.94E-06	2.59E-05	16.439	77.249	1.312									
Physical activity	MDD	physactivity	MR Egger	154	-0.110	0.110	0.317	0.576	79.156	100.000	0.101					0.001	0.003	0.654		
Physical activity	MDD	physactivity	Weighted median	154	-0.037	0.027	0.171	0.224	79.156	100.000	0.101									
Physical activity	MDD	physactivity	Inverse variance weighted	154	-0.062	0.024	0.009	0.015	79.156	100.000	0.101	0.0093	40.626	0.459	unweighted				285.131	5.00E-10
Physical activity	MDD	physactivity	Simple mode	154	-0.008	0.088	0.931	0.975	79.156	100.000	0.101									
Physical activity	MDD	physactivity	Weighted mode	154	-0.012	0.082	0.883	0.966	79.156	100.000	0.101									
Physical activity	MDD	physactivity	Steiger-filtered IVW	153	-0.057	0.023	0.014	0.023	79.156	100.000	0.101									
Physical activity	MDD	physactivity	GSMR	151	-0.042	0.018	0.020	0.029	79.156	100.000	0.101									
Physical activity	MDD	physactivity	PRESSO	154	-0.051	0.023	0.025	0.035	79.156	100.000	0.101									
Pregnancy terminations	MDD	pregnantermin	MR Egger	154	0.188	0.085	0.029	0.151	67.221	100.000	0.116					-0.002	0.002	0.280		
Pregnancy terminations	MDD	pregnantermin	Weighted median	154	0.106	0.023	4.54E-06	1.26E-05	67.221	100.000	0.116									
Pregnancy terminations	MDD	pregnantermin	Inverse variance weighted	154	0.098	0.018	1.23E-07	3.65E-07	67.221	100.000	0.116	0.0093	40.626	0.459	unweighted				224.947	1.37E-04
Pregnancy terminations	MDD	pregnantermin	Simple mode	154	0.151	0.072	0.037	0.110	67.221	100.000	0.116									
Pregnancy terminations	MDD	pregnantermin	Weighted mode	154	0.151	0.063	0.018	0.065	67.221	100.000	0.116									
Pregnancy terminations	MDD	pregnantermin	Steiger-filtered IVW	152	0.089	0.018	3.76E-07	9.55E-07	67.221	100.000	0.116									
Pregnancy terminations	MDD	pregnantermin	GSMR	152	0.085	0.016	5.05E-08	1.07E-07	67.221	100.000	0.116									
Pregnancy terminations	MDD	pregnantermin	PRESSO	154	0.093	0.018	7.29E-07	1.58E-06	67.221	100.000	0.116									
Pregnancy terminations	MDD	pregnantermin	LHC		0.224	0.087	0.010	0.021	67.221	100.000	0.116									
Rheumatoid arthritis	MDD	rheuma	MR Egger	151	-0.541	0.408	0.188	0.428	18.960	84.485	1.280					0.014	0.009	0.134		
Rheumatoid arthritis	MDD	rheuma	Weighted median	151	0.201	0.094	0.032	0.051	18.960	84.485	1.280									
Rheumatoid arthritis	MDD	rheuma	Inverse variance weighted	151	0.059	0.091	0.511	0.599	18.960	84.485	1.280	0.0091	40.728	0.466	unweighted				343.409	3.35E-17
Rheumatoid arthritis	MDD	rheuma	Simple mode	151	0.338	0.276	0.223	0.397	18.960	84.485	1.280									
Rheumatoid arthritis	MDD	rheuma	Weighted mode	151	0.358	0.283	0.208	0.374	18.960	84.485	1.280									
Rheumatoid arthritis	MDD	rheuma	Steiger-filtered IVW	148	0.123	0.073	0.092	0.128	18.960	84.485	1.280									
Rheumatoid arthritis	MDD	rheuma	GSMR	146	0.157	0.062	0.011	0.017	18.960	84.485	1.280									
Rheumatoid arthritis	MDD	rheuma	PRESSO	151	0.144	0.071	0.045	0.057	18.960	84.485	1.280									
Rheumatoid arthritis	MDD	rheuma	LHC		-0.142	0.245	0.563	0.662	18.960	84.485	1.280									
Risky behaviours	MDD	riskbehavior	MR Egger	153	0.076	0.100	0.449	0.740	100.000	100.000	0.031					0.002	0.002	0.506		
Risky behaviours	MDD	riskbehavior	Weighted median	153	0.128	0.019	7.51E-12	2.67E-11	100.000	100.000	0.031									
Risky behaviours	MDD	riskbehavior	Inverse variance weighted	153	0.141	0.022	1.12E-10	3.97E-10	100.000	100.000	0.031	0.0092	40.644	0.462	unweighted				702.680	1.13E-71
Risky behaviours	MDD	riskbehavior	Simple mode	153	0.111	0.062	0.075	0.185	100.000	100.000	0.031									
Risky behaviours	MDD	riskbehavior	Weighted mode	153	0.154	0.058	0.009	0.038	100.000	100.000	0.031									
Risky behaviours	MDD	riskbehavior	Steiger-filtered IVW	153	0.141	0.022	1.12E-10	3.82E-10	100.000	100.000	0.031									
Risky behaviours	MDD	riskbehavior	GSMR	139	0.143	0.011	2.41E-38	1.19E-37	100.000	100.000	0.031									
Risky behaviours	MDD	riskbehavior	PRESSO	153	0.142	0.016	1.85E-15	6.34E-15	100.000	100.000	0.031									
Risky behaviours	MDD	riskbehavior	LHC		-0.408	0.064	2.52E-10	1.46E-09	100.000	100.000	0.031									
Sedentary behavior	MDD	sedentary	MR Egger	154	-0.008	0.110	0.940	0.940	79.759	100.000	0.100					2.92E-04	0.003	0.908		
Sedentary behavior	MDD	sedentary	Weighted median	154	-0.010	0.028	0.712	0.782	79.759	100.000	0.100									
Sedentary behavior	MDD	sedentary	Inverse variance weighted	154	0.004	0.024	0.866	0.906	79.759	100.000	0.100	0.0093	40.626	0.459	unweighted				259.820	1.56E-07
Sedentary behavior	MDD	sedentary	Simple mode	154	-0.004	0.091	0.962	0.984	79.759	100.000	0.100									
Sedentary behavior	MDD	sedentary	Weighted mode	154	0.000	0.082	0.997	0.997	79.759	100.000	0.100									
Sedentary behavior	MDD	sedentary	Steiger-filtered IVW	154	0.004	0.024	0.866	0.906	79.759	100.000	0.100									
Sedentary behavior	MDD	sedentary	GSMR	154	0.002	0.019	0.896	0.917	79.759	100.000	0.100									
Sedentary behavior	MDD	sedentary	LHC		0.463	0.103	7.59E-06	2.28E-05	79.759	100.000	0.100									
Self-reported health	MDD	health	MR Egger	152	0.287	0.101	0.005	0.058	83.184	100.000	0.096					1.48E-04	0.002	0.950		
Self-reported health	MDD	health	Weighted median	152	0.273	0.026	3.65E-25	2.50E-24	83.184	100.000	0.096									
Self-reported health	MDD	health	Inverse variance weighted	152	0.293	0.022	4.42E-40	4.37E-39	83.184	100.000	0.096	0.0092	40.701	0.463	unweighted				264.000	3.56E-08
Self-reported health	MDD	health	Simple mode	152	0.388	0.090	2.90E-05	3.69E-04	83.184	100.000	0.096									

Self-reported health	MDD	health	Weighted mode	152	0.257	0.070	3.43E-04	0.002	83.184	100.000	0.096									
Self-reported health	MDD	health	Steiger-filtered IVW	150	0.282	0.021	4.26E-41	4.21E-40	83.184	100.000	0.096									
Self-reported health	MDD	health	GSMR	150	0.265	0.017	4.69E-52	2.98E-51	83.184	100.000	0.096									
Self-reported health	MDD	health	PRESSO	152	0.282	0.021	1.95E-27	1.41E-26	83.184	100.000	0.096									
Self-reported health	MDD	health	LHC		0.728	0.067	9.58E-28	1.39E-26	83.184	100.000	0.096									
Self-reported illnesses	MDD	illnesses	MR Egger	154	0.313	0.121	0.011	0.087	99.780	100.000	0.058					0.002	0.003	0.422		
Self-reported illnesses	MDD	illnesses	Weighted median	154	0.348	0.028	1.48E-36	2.19E-35	99.780	100.000	0.058									
Self-reported illnesses	MDD	illnesses	Inverse variance weighte	154	0.408	0.026	1.09E-54	1.62E-53	99.780	100.000	0.058	0.0093	40.626	0.459	unweighted				407.537	5.12E-25
Self-reported illnesses	MDD	illnesses	Simple mode	154	0.317	0.089	5.06E-04	0.003	99.780	100.000	0.058									
Self-reported illnesses	MDD	illnesses	Weighted mode	154	0.311	0.083	2.34E-04	0.002	99.780	100.000	0.058									
Self-reported illnesses	MDD	illnesses	Steiger-filtered IVW	154	0.408	0.026	1.09E-54	1.62E-53	99.780	100.000	0.058									
Self-reported illnesses	MDD	illnesses	GSMR	148	0.384	0.018	7.29E-106	1.08E-104	99.780	100.000	0.058									
Self-reported illnesses	MDD	illnesses	PRESSO	154	0.410	0.022	2.15E-41	4.65E-40	99.780	100.000	0.058									
Self-reported illnesses	MDD	illnesses	LHC		0.694	0.193	3.15E-04	7.84E-04	99.780	100.000	0.058									
Severe covid 19	MDD	covid	MR Egger	153	0.191	0.324	0.558	0.788	18.418	83.124	1.286					0.002	0.007	0.768		
Severe covid 19	MDD	covid	Weighted median	153	0.230	0.091	0.011	0.019	18.418	83.124	1.286									
Severe covid 19	MDD	covid	Inverse variance weighte	153	0.284	0.068	3.22E-05	7.16E-05	18.418	83.124	1.286	0.0092	40.644	0.462	unweighted				203.782	0.003
Severe covid 19	MDD	covid	Simple mode	153	0.115	0.279	0.681	0.857	18.418	83.124	1.286									
Severe covid 19	MDD	covid	Weighted mode	153	0.152	0.251	0.546	0.714	18.418	83.124	1.286									
Severe covid 19	MDD	covid	Steiger-filtered IVW	153	0.284	0.068	3.22E-05	6.98E-05	18.418	83.124	1.286									
Severe covid 19	MDD	covid	GSMR	152	0.246	0.060	4.52E-05	7.89E-05	18.418	83.124	1.286									
Severe covid 19	MDD	covid	PRESSO	153	0.262	0.066	1.12E-04	1.87E-04	18.418	83.124	1.286									
Severe covid 19	MDD	covid	LHC		0.070	0.018	1.60E-04	4.09E-04	18.418	83.124	1.286									
Sleep duration	MDD	sleepduration	MR Egger	154	0.193	0.112	0.088	0.257	79.066	100.000	0.101					-0.004	0.003	0.178		
Sleep duration	MDD	sleepduration	Weighted median	154	0.048	0.028	0.082	0.117	79.066	100.000	0.101									
Sleep duration	MDD	sleepduration	Inverse variance weighte	154	0.044	0.024	0.070	0.103	79.066	100.000	0.101	0.0093	40.626	0.459	unweighted				280.570	1.47E-09
Sleep duration	MDD	sleepduration	Simple mode	154	0.026	0.086	0.767	0.902	79.066	100.000	0.101									
Sleep duration	MDD	sleepduration	Weighted mode	154	0.049	0.078	0.536	0.712	79.066	100.000	0.101									
Sleep duration	MDD	sleepduration	Steiger-filtered IVW	154	0.044	0.024	0.070	0.102	79.066	100.000	0.101									
Sleep duration	MDD	sleepduration	GSMR	151	0.044	0.019	0.018	0.026	79.066	100.000	0.101									
Sleep duration	MDD	sleepduration	PRESSO	154	0.037	0.023	0.113	0.139	79.066	100.000	0.101									
Sleep duration	MDD	sleepduration	LHC		0.465	0.067	5.58E-12	4.41E-11	79.066	100.000	0.101									
Smoking cessation	MDD	smokingces	MR Egger	45	-0.012	0.151	0.939	0.940	58.913	99.997	1.130					0.002	0.003	0.478		
Smoking cessation	MDD	smokingces	Weighted median	45	0.110	0.026	1.97E-05	4.88E-05	58.913	99.997	1.130									
Smoking cessation	MDD	smokingces	Inverse variance weighte	45	0.094	0.032	0.003	0.005	58.913	99.997	1.130	0.0028	42.298	0.504	unweighted				204.193	1.74E-22
Smoking cessation	MDD	smokingces	Simple mode	45	0.157	0.067	0.023	0.079	58.913	99.997	1.130									
Smoking cessation	MDD	smokingces	Weighted mode	45	0.132	0.057	0.026	0.083	58.913	99.997	1.130									
Smoking cessation	MDD	smokingces	Steiger-filtered IVW	45	0.094	0.032	0.003	0.005	58.913	99.997	1.130									
Smoking cessation	MDD	smokingces	GSMR	42	0.101	0.016	2.57E-10	6.36E-10	58.913	99.997	1.130									
Smoking cessation	MDD	smokingces	PRESSO	45	0.103	0.024	1.29E-04	2.09E-04	58.913	99.997	1.130									
Smoking cessation	MDD	smokingces	LHC		0.466	0.069	1.28E-11	9.31E-11	58.913	99.997	1.130									
Smoking initiation	MDD	smokingever	MR Egger	45	0.204	0.106	0.062	0.215	96.001	100.000	1.075					0.000	0.002	0.954		
Smoking initiation	MDD	smokingever	Weighted median	45	0.215	0.021	1.33E-23	8.44E-23	96.001	100.000	1.075									
Smoking initiation	MDD	smokingever	Inverse variance weighte	45	0.198	0.024	6.52E-17	3.42E-16	96.001	100.000	1.075	0.0028	42.298	0.504	unweighted				141.332	3.84E-12
Smoking initiation	MDD	smokingever	Simple mode	45	0.250	0.049	6.10E-06	1.36E-04	96.001	100.000	1.075									
Smoking initiation	MDD	smokingever	Weighted mode	45	0.237	0.045	3.30E-06	6.56E-05	96.001	100.000	1.075									
Smoking initiation	MDD	smokingever	Steiger-filtered IVW	45	0.198	0.024	6.52E-17	3.23E-16	96.001	100.000	1.075									
Smoking initiation	MDD	smokingever	GSMR	42	0.193	0.015	1.38E-39	7.20E-39	96.001	100.000	1.075									
Smoking initiation	MDD	smokingever	PRESSO	45	0.200	0.020	8.02E-13	2.17E-12	96.001	100.000	1.075									
Smoking initiation	MDD	smokingever	LHC		0.279	0.074	1.53E-04	4.04E-04	96.001	100.000	1.075									
Staphylococcus aureus	MDD	staphylococcus	MR Egger	147	0.096	0.409	0.815	0.884	10.138	47.174	1.476					0.002	0.009	0.846		
Staphylococcus aureus	MDD	staphylococcus	Weighted median	147	0.130	0.101	0.199	0.253	10.138	47.174	1.476									
Staphylococcus aureus	MDD	staphylococcus	Inverse variance weighte	147	0.174	0.079	0.027	0.044	10.138	47.174	1.476	0.0087	40.134	0.340	unweighted				203.144	0.001
Staphylococcus aureus	MDD	staphylococcus	Simple mode	147	0.035	0.306	0.910	0.975	10.138	47.174	1.476									
Staphylococcus aureus	MDD	staphylococcus	Weighted mode	147	0.063	0.297	0.832	0.937	10.138	47.174	1.476									
Staphylococcus aureus	MDD	staphylococcus	Steiger-filtered IVW	147	0.174	0.079	0.027	0.042	10.138	47.174	1.476									
Staphylococcus aureus	MDD	staphylococcus	GSMR	147	0.169	0.068	0.013	0.019	10.138	47.174	1.476									
Staphylococcus aureus	MDD	staphylococcus	LHC		0.016	0.034	0.637	0.710	10.138	47.174	1.476									
Suicidal behavior	MDD	suicbehav	MR Egger	147	0.890	0.259	7.60E-04	0.017	30.994	98.077	1.200					-0.002	0.006	0.774		
Suicidal behavior	MDD	suicbehav	Weighted median	147	0.770	0.062	5.20E-35	6.61E-34	30.994	98.077	1.200									
Suicidal behavior	MDD	suicbehav	Inverse variance weighte	147	0.817	0.047	3.84E-67	8.54E-66	30.994	98.077	1.200	0.0088	40.392	0.280	unweighted				201.279	0.002
Suicidal behavior	MDD	suicbehav	Simple mode	147	0.787	0.193	7.77E-05	7.63E-04	30.994	98.077	1.200									
Suicidal behavior	MDD	suicbehav	Weighted mode	147	0.814	0.195	5.26E-05	6.69E-04	30.994	98.077	1.200									
Suicidal behavior	MDD	suicbehav	Steiger-filtered IVW	147	0.817	0.047	3.84E-67	8.54E-66	30.994	98.077	1.200									
Suicidal behavior	MDD	suicbehav	GSMR	146	0.779	0.042	1.95E-76	1.58E-75	30.994	98.077	1.200									
Suicidal behavior	MDD	suicbehav	LHC		0.313	0.052	1.81E-09	9.83E-09	30.994	98.077	1.200									
Suicidal thought	MDD	suicthought	MR Egger	154	0.564	0.131	3.13E-05	9.29E-04	76.509	100.000	1.105					0.000	0.003	0.874		
Suicidal thought	MDD	suicthought	Weighted median	154	0.503	0.033	4.94E-53	4.40E-51	76.509	100.000	1.105									
Suicidal thought	MDD	suicthought	Inverse variance weighte	154	0.543	0.026	8.32E-94	7.41E-92	76.509	100.000	1.105	0.0093	40.626	0.459	unweighted				277.812	2.81E-09
Suicidal thought	MDD	suicthought	Simple mode	154	0.499	0.103	3.24E-06	9.61E-05	76.509	100.000	1.105									
Suicidal thought	MDD	suicthought	Weighted mode	154	0.484	0.100	3.14E-06	6.56E-05	76.509	100.000	1.105									
Suicidal thought	MDD	suicthought	Steiger-filtered IVW	154	0.543	0.026	8.32E-94	7.41E-92	76.509	100.000	1.105									
Suicidal thought	MDD	suicthought	GSMR	152	0.521	0.021	8.14E-133	7.25E-131	76.509	100.000	1.105									
Suicidal thought	MDD	suicthought	PRESSO	154	0.541	0.025	1.50E-47	9.72E-46	76.509	100.000	1.105									
Suicidal thought	MDD	suicthought	LHC		0.414	0.022	7.89E-79	2.29E-77	76.509	100.000	1.105									
Suicide death	MDD	suicdeath	MR Egger	146	0.382	0.696	0.584	0.788	3.975	8.197	3.648					0.017	0.016	0.280		
Suicide death	MDD	suicdeath	Weighted median	146	0.829	0.185	7.07E-06	1.80E-05	3.975	8.197	3.648									
Suicide death	MDD	suicdeath	Inverse variance weighte	146	1.120	0.141	2.48E-15	1.10E-14	3.975	8.197	3.648	0.0089	40.895	0.537	weighted				207.062	5.51E-04
Suicide death	MDD	suicdeath	Simple mode	146	0.232	0.577	0.688	0.857	3.975	8.197	3.648									
Suicide death	MDD	suicdeath	Weighted mode	146	0.421	0.565	0.457	0.632	3.975	8.197	3.648									
Suicide death	MDD	suicdeath	Steiger-filtered IVW	140	1.026	0.129	1.99E-15	8.85E-15	3.975	8.197	3.648									
Suicide death	MDD	suicdeath	GSMR	145	1.082	0.121	4.92E-19	1.56E-18	3.975	8.197	3.648									
Suicide death	MDD	suicdeath	PRESSO	146	1.155	0.137	3.68E-14	1.09E-13	3.975	8.197	3.648									
Suicide death	MDD	suicdeath	LHC		1.000	0.003	0.00E+00	0.00E+00	3.975	8.197	3.648									
Systemic lupus erythematosus	MDD	lupus	MR Egger	134	-2.542	0.859	0.004	0.054	5.092	14.275	2.280					0.061	0.020	0.003		
Systemic lupus erythematosus	MDD	lupus	Weighted median	134	0.396	0.182	0.030	0.048	5.092	14.275	2.280									
Systemic lupus erythematosus	MDD	lupus	Inverse variance weighte	134	0.003	0.195	0.988	0.988	5.092	14.275	2.280	0.0081	41.011	0.632	weighted				366.507	1.02E-23
Systemic lupus erythematosus	MDD	lupus	Simple mode	134	1.127	0.551	0.043	0.123	5.092	14.275	2.280									
Systemic lupus erythematosus	MDD	lupus	Weighted mode	134	0.898	0.430	0.039	0.119	5.092	14.275	2.280									
Systemic lupus erythematosus	MDD	lupus	Steiger-filtered IVW	133	0.228	0.139	0.102	0.140	5.092	14.275	2.280									
Systemic lupus erythematosus	MDD	lupus	MR Egger SIMEX		-3.788	1.181	0.002	0.003	5.092	14.275	2.280									
Systemic lupus erythematosus	MDD	lupus	GSMR	133	0.221	0.120	0.066	0.089	5.092	14.275	2.280									
Systemic lupus erythematosus	MDD	lupus	PRESSO	134	0.228	0.139	0.104	0.131	5.092	14.275	2.280									
Systemic lupus erythematosus	MDD	lupus	LHC		0.843	0.174	1.29E-06	4.33E-06	5.092	14.275	2.280									
Systolic blood pressure	MDD	sysbloodpres	MR Egger	81	0.735	2.038	0.719	0.854	67.635	100.000	0.116					-0.012	0.045	0.791		
Systolic blood pressure	MDD	sysbloodpres	Weighted median	81	0.314	0.231	0.174	0.225	67.635	100.000	0.116									
Systolic blood pressure	MDD	sysbloodpres	Inverse variance weighte	81	0.202	0.345	0.559	0.644	67.635	100.000	0.116	0.0048	40.098	0.164	unweighted				562.608	1.27E-73
Systolic blood pressure	MDD	sysbloodpres	Simple mode	81	0.698	0.627	0.270	0.453	67.635	100.000	0.116									
Systolic blood pressure	MDD	sysbloodpres	Weighted mode	81	0.645	0.514	0.213	0.374	67.635	100.000	0.116									
Systolic blood pressure	MDD	sysbloodpres	Steiger-filtered IVW	80	0.058	0.312	0.853	0.904	67.635	100.000	0.116									
Systolic blood pressure	MDD	sysbloodpres	GSMR	72	0.168	0.142	0.238	0.283	67.635	100.000	0.116									
Systolic blood pressure	MDD	sysbloodpres	PRESSO	81	0.212	0.229	0.358	0.401	67.635	100.000	0.116									
Systolic blood pressure	MDD	sysbloodpres	LHC		-0.018	0.084	0.828	0.879	67.63503078	99.99999397	0.116									
Tea consumption	MDD	tea	MR Egger	152	-0.274	0.284	0.337	0.600	19.63011821	86.03672097	1.273					0.007	0.007	0.311		
Tea consumption	MDD	tea	Weighted median	152	-0.053	0.086	0.543	0.628	19.63011821	86.03672097	1.273									
Tea consumption	MDD	tea	Inverse variance weighte	152	0.009	0.062	0.890	0.921	19.63011821	86.03672097	1.273	0.0092	40.654	0.460	unweighted				186.387	0.027
Tea consumption	MDD	tea	Simple mode	152	-0.425	0.260	0.105	0.253	19.63011821	86.03672097	1.273									
Tea consumption	MDD	tea	Weighted mode	152	-0.425	0.265	0.110	0.266	19.63011821	86.03672097	1.273									
Tea consumption	MDD	tea	Steiger-filtered IVW	152	0.009	0.062	0.890	0.911	19.63011821	86.03672097	1.273									
Tea consumption	MDD	tea	GSMR	152	0.004	0.056	0.947	0.958	19.63011821	86.03672097	1.273									
Tea consumption	MDD	tea	LHC		-0.030	0.046	0.512	0.619	19.63011821	86.03672097	1.273									
Total testosterone levels	MDD	testosterone	MR Egger	153	-0.042	0.079	0.600	0.788	99.53560428	100	0.061					3.21E-04	0.002	0.861		
Total testosterone levels	MDD	testosterone	Weighted median	153	-0.033	0.018	0.064	0.094	99.53560428	100	0.061									
Total testosterone levels	MDD	testosterone	Inverse variance weighte	153	-0.028	0.017	0.101	0.143	99.53560428	100	0.061	0.0092	40.635	0.457	unweighted				367.445	7.13E-20
Total testosterone levels	MDD	testosterone	Simple mode	153	-0.007	0.057	0.908	0.975	99.53560428	100	0.061									
Total testosterone levels	MDD	testosterone	Weighted mode	153	0.020	0.047	0.676	0.873	99.53560428	100	0.061									
Total testosterone levels	MDD	testosterone	Steiger-filtered IVW	152	-0.033	0.016	0.044	0.068	99.53560428	100	0.061									
Total testosterone levels	MDD	testosterone	GSMR	148	-0.033	0.012	0.005	0.007	99.53560428	100	0.061									
Total testosterone levels	MDD	testosterone	PRESSO	153	-0.032	0.014	0.027	0.036	99.53560428	100	0.061									
Total testosterone levels	MDD	testosterone	LHC		-0.051	0.031	0.104	0.159	99.53560428	100	0.061									
Traumatic injury	MDD	traumainjury	MR Egger	152	0.354	0.145	0.016	0.111	40.27994021	99.70907551	1.169					6.95E-04	0.003	0.836		
Traumatic injury	MDD	traumainjury	Weighted median	152	0.415	0.043	6.32E-22	3.75E-21	40.27994021	99.70907551	1.169									
Traumatic injury	MDD	traumainjury	Inverse variance weighte	152	0.383	0.031	4.37E-34	3.53E-33	40.27994021	99.70907551	1.169	0.0092	40.654	0.460	unweighted				179.978	0.054
Traumatic injury	MDD	traumainjury	Simple mode	152	0.477	0.127	2.37E-04	0.002	40.27994021	99.70907551	1.169									
Traumatic injury	MDD	traumainjury	Weighted mode	152	0.483	0.129	2.49E-04	0.002	40.27994021	99.70907551	1.169									
Traumatic injury	MDD	traumainjury	Steiger-filtered IVW	152	0.383	0.031	4.37E-34	3.53E-33	40.27994021	99.70907551	1.169									
Traumatic injury	MDD	traumainjury	GSMR	152	0.366	0.030	3.30E-35	1.40E-34	40.27994021	99.70907551	1.169									
Traumatic injury	MDD	traumainjury	LHC		0.277	0.065	1.72E-05	4.66E-05	40.27994021	99.70907551	1.169									
Triglycerides	MDD	tgl	MR Egger	153	0.113	0.103	0.274	0.542	100	100	0.026					0.000	0.002	0.984		
Triglycerides	MDD	tgl	Weighted median	153	0.082	0.010	2.08E-15	9.25E-15	100	100	0.026									
Triglycerides	MDD	tgl	Inverse variance weighte	153	0.111	0.021	1.86E-07	5.18E-07	100	100	0.026	0.0092	40.644	0.462	unweighted				2300.850	0.00E+00
Triglycerides	MDD	tgl	Simple mode	153	0.082	0.027	0.003	0.014	100	100	0.026									
Triglycerides	MDD	tgl	Weighted mode	153	0.075	0.021	5.92E-04	0.004	100	100	0.026									
Triglycerides	MDD	tgl	Steiger-filtered IVW	151	0.092	0.014	1.50E-11	5.33E-11	100	100	0.026									
Triglycerides	MDD	tgl	GSMR	130	0.097	0.006	5.50E-54	3.77E-53	100	100	0.026									
Triglycerides	MDD	tgl	PRESSO	153	0.101	0.010	2.65E-18	1.23E-17	100	100	0.026									
Triglycerides	MDD	tgl	LHC		0.271	0.100	0.007	0.014	100	100	0.026									
Type II diabetes	MDD	t2d	MR Egger	154	0.257	0.229	0.264	0.534	47.00520423	99.93777315	1.152					2.61E-04	0.005	0.961		
Type II diabetes	MDD	t2d	Weighted median	154	0.220	0.044	5.31E-07	1.57E-06	47.00520423	99.93777315	1.152									
Type II diabetes	MDD	t2d	Inverse variance weighte	154	0.268	0.049	3.46E-08	1.10E-07	47.00520423	99.93777315	1.152	0.0093	40.626	0.459	unweighted				565.438	1.24E-48
Type II diabetes	MDD	t2d	Simple mode	154	0.211	0.153	0.171	0.375	47.00520423	99.93777315	1.152									
Type II diabetes	MDD	t2d	Weighted mode	154	0.135	0.155	0.386	0.566	47.00520423	99.93777315	1.152									
Type II diabetes	MDD	t2d	Steiger-filtered IVW	154	0.268	0.049	3.46E-08	9.93E-08	47.00520423	99.93777315	1.152									
Type II diabetes	MDD	t2d	GSMR	143	0.219	0.027	4.65E-16	1.38E-15	47.00520423	99.93777315	1.152									
Type II diabetes	MDD	t2d	PRESSO	154	0.251	0.041	8.67E-09	2.01E-08	47.00520423	99.93777315	1.152									
Type II diabetes	MDD	t2d	LHC		0.152	0.077	0.049	0.082	47.00520423	99.93777315	1.152									
Ulcerative colitis	MDD	ulcercolit	MR Egger	145	0.123	0.373	0.741	0.868	7.521641574	30.04370067	1.668					-0.003	0.009	0.755		
Ulcerative colitis	MDD	ulcercolit	Weighted median	145	0.008	0.098	0.932	0.932	7.521641574	30.04370067	1.668									
Ulcerative colitis	MDD	ulcercolit	Inverse variance weighte	145	0.009	0.081	0.907	0.928	7.521641574	30.04370067	1.668	0.0088	40.726	0.483	unweighted				226.083	1.48E-05

Ulcerative colitis	MDD	ulcercolit	Simple mode	145	-0.041	0.280	0.884	0.975	7.521641574	30.04370067	1.668									
Ulcerative colitis	MDD	ulcercolit	Weighted mode	145	-0.012	0.240	0.961	0.997	7.521641574	30.04370067	1.668									
Ulcerative colitis	MDD	ulcercolit	Steiger-filtered IVW	145	0.009	0.081	0.907	0.918	7.521641574	30.04370067	1.668									
Ulcerative colitis	MDD	ulcercolit	GSMR	142	0.065	0.066	0.324	0.375	7.521641574	30.04370067	1.668									
Ulcerative colitis	MDD	ulcercolit	PRESSO	145	0.037	0.079	0.640	0.660	7.521641574	30.04370067	1.668									
Ulcerative colitis	MDD	ulcercolit	LHC		0.152	0.066	0.022	0.041	7.521641574	30.04370067	1.668									
Variation in diet	MDD	dietvariation	MR Egger	154	-0.009	0.036	0.802	0.884	99.99702681	100	0.047					0.001	######	0.193		
Variation in diet	MDD	dietvariation	Weighted median	154	0.044	0.009	9.64E-07	2.77E-06	99.99702681	100	0.047									
Variation in diet	MDD	dietvariation	Inverse variance weighte	154	0.037	0.008	2.37E-06	6.03E-06	99.99702681	100	0.047	0.0093	40.626	0.459	unweighted				328.326	7.72E-15
Variation in diet	MDD	dietvariation	Simple mode	154	0.083	0.032	0.010	0.042	99.99702681	100	0.047									
Variation in diet	MDD	dietvariation	Weighted mode	154	0.072	0.035	0.041	0.119	99.99702681	100	0.047									
Variation in diet	MDD	dietvariation	Steiger-filtered IVW	154	0.037	0.008	2.37E-06	5.86E-06	99.99702681	100	0.047									
Variation in diet	MDD	dietvariation	GSMR	153	0.035	0.005	1.77E-10	4.50E-10	99.99702681	100	0.047									
Variation in diet	MDD	dietvariation	LHC		0.143	0.204	0.484	0.602	99.99702681	100	0.047									
Vegetable consumption	MDD	vegetable	MR Egger	153	-0.092	0.314	0.771	0.880	19.88398976	86.58784366	1.271					-0.001	0.007	0.934		
Vegetable consumption	MDD	vegetable	Weighted median	153	-0.242	0.090	0.007	0.013	19.88398976	86.58784366	1.271									
Vegetable consumption	MDD	vegetable	Inverse variance weighte	153	-0.117	0.068	0.086	0.125	19.88398976	86.58784366	1.271	0.0092	40.644	0.462	unweighted				205.355	0.003
Vegetable consumption	MDD	vegetable	Simple mode	153	-0.575	0.289	0.048	0.133	19.88398976	86.58784366	1.271									
Vegetable consumption	MDD	vegetable	Weighted mode	153	-0.538	0.322	0.097	0.248	19.88398976	86.58784366	1.271									
Vegetable consumption	MDD	vegetable	Steiger-filtered IVW	153	-0.117	0.068	0.086	0.121	19.88398976	86.58784366	1.271									
Vegetable consumption	MDD	vegetable	GSMR	153	-0.117	0.059	0.049	0.067	19.88398976	86.58784366	1.271									
Vegetable consumption	MDD	vegetable	LHC		-0.080	0.037	0.033	0.059	19.88398976	86.58784366	1.271									
Years ovulating	MDD	ovulating	MR Egger	153	1.158	0.826	0.163	0.393	36.51590399	99.80480271	0.173					-0.032	0.019	0.096		
Years ovulating	MDD	ovulating	Weighted median	153	-0.241	0.216	0.266	0.324	36.51590399	99.80480271	0.173									
Years ovulating	MDD	ovulating	Inverse variance weighte	153	-0.195	0.180	0.278	0.344	36.51590399	99.80480271	0.173	0.0092	40.635	0.457	unweighted				234.730	1.89E-05
Years ovulating	MDD	ovulating	Simple mode	153	-0.242	0.633	0.703	0.857	36.51590399	99.80480271	0.173									
Years ovulating	MDD	ovulating	Weighted mode	153	-0.242	0.679	0.722	0.902	36.51590399	99.80480271	0.173									
Years ovulating	MDD	ovulating	Steiger-filtered IVW	153	-0.195	0.180	0.278	0.343	36.51590399	99.80480271	0.173									
Years ovulating	MDD	ovulating	GSMR	153	-0.182	0.147	0.216	0.267	36.51590399	99.80480271	0.173									
Years ovulating	MDD	ovulating	LHC		-0.039	0.081	0.631	0.710	36.51590399	99.80480271	0.173									
Zinc supplements	MDD	zinc	MR Egger	153	0.188	0.212	0.375	0.630	22.22783068	90.83693369	1.25					0.000	0.005	0.995		
Zinc supplements	MDD	zinc	Weighted median	153	0.195	0.061	0.001	0.003	22.22783068	90.83693369	1.25									
Zinc supplements	MDD	zinc	Inverse variance weighte	153	0.187	0.046	4.58E-05	9.95E-05	22.22783068	90.83693369	1.25	0.0092	40.644	0.462	unweighted				179.449	0.063
Zinc supplements	MDD	zinc	Simple mode	153	0.114	0.205	0.579	0.818	22.22783068	90.83693369	1.25									
Zinc supplements	MDD	zinc	Weighted mode	153	0.145	0.191	0.447	0.632	22.22783068	90.83693369	1.25									
Zinc supplements	MDD	zinc	Steiger-filtered IVW	153	0.187	0.046	4.58E-05	9.71E-05	22.22783068	90.83693369	1.25									
Zinc supplements	MDD	zinc	GSMR	153	0.179	0.043	2.85E-05	5.17E-05	22.22783068	90.83693369	1.25									
Zinc supplements	MDD	zinc	LHC		0.176	0.031	1.76E-08	7.65E-08	22.22783068	90.83693369	1.25									
MDD	25-Hydroxyvitamin D level	vitamind	MR Egger	18	-0.011	0.023	0.639	0.980	99.99999887	100	1.036		136.039			2.94E-04	0.002	0.907		
MDD	25-Hydroxyvitamin D level	vitamind	Weighted median	18	-0.023	0.018	0.186	0.460	99.99999887	100	1.036		136.039							
MDD	25-Hydroxyvitamin D level	vitamind	Inverse variance weighte	18	-0.009	0.014	0.535	0.755	99.99999887	100	1.036	0.0586	136.039	0.984	weighted				18.461	0.360
MDD	25-Hydroxyvitamin D level	vitamind	Simple mode	18	0.045	0.031	0.173	0.702	99.99999887	100	1.036		136.039							
MDD	25-Hydroxyvitamin D level	vitamind	Weighted mode	18	-0.019	0.018	0.285	0.902	99.99999887	100	1.036		136.039							
MDD	25-Hydroxyvitamin D level	vitamind	Steiger-filtered IVW	18	-0.009	0.014	0.535	0.755	99.99999887	100	1.036		136.039							
MDD	25-Hydroxyvitamin D level	vitamind	GSMR	18	-0.009	0.014	0.494	0.638												
MDD	25-Hydroxyvitamin D level	vitamind	LHC		0.012	0.052	0.815	0.933												
MDD	Adenocarcinoma	adenocarcinoma	MR Egger	12	0.030	0.076	0.703	0.980	84.23010297	99.99999997	1.094		45.634			-0.003	0.010	0.778		
MDD	Adenocarcinoma	adenocarcinoma	Weighted median	12	3.60E-04	0.017	0.984	0.995	84.23010297	99.99999997	1.094		45.634							
MDD	Adenocarcinoma	adenocarcinoma	Inverse variance weighte	12	0.008	0.017	0.627	0.836	84.23010297	99.99999997	1.094	0.0090	45.634	0.670	unweighted				21.020	0.033
MDD	Adenocarcinoma	adenocarcinoma	Simple mode	12	-0.009	0.030	0.775	0.975	84.23010297	99.99999997	1.094		45.634							
MDD	Adenocarcinoma	adenocarcinoma	Weighted mode	12	0.004	0.023	0.870	0.967	84.23010297	99.99999997	1.094		45.634							
MDD	Adenocarcinoma	adenocarcinoma	Steiger-filtered IVW	12	0.008	0.017	0.627	0.836	84.23010297	99.99999997	1.094		45.634							
MDD	Adenocarcinoma	adenocarcinoma	MR Egger SIMEX		-0.020	0.051	0.701	0.897	84.23010297	99.99999997	1.094		45.634							
MDD	Adenocarcinoma	adenocarcinoma	GSMR	12	0.007	0.013	0.601	0.747												
MDD	Adenocarcinoma	adenocarcinoma	PRESSO	12	-0.003	0.014	0.813	0.855												
MDD	Adenocarcinoma	adenocarcinoma	LHC		-0.003	0.011	0.777	0.932												
MDD	Age at menarche	menarche	MR Egger	181	0.004	0.035	0.906	0.980	99.99999744	100	1.037		62.700			-0.002	0.002	0.295		
MDD	Age at menarche	menarche	Weighted median	181	-0.029	0.014	0.036	0.137	99.99999744	100	1.037		62.700							
MDD	Age at menarche	menarche	Inverse variance weighte	181	-0.030	0.012	0.013	0.048	99.99999744	100	1.037	0.0563	62.700	0.870	unweighted				420.551	2.66E-21
MDD	Age at menarche	menarche	Simple mode	181	-0.018	0.037	0.622	0.975	99.99999744	100	1.037		62.700							
MDD	Age at menarche	menarche	Weighted mode	181	-0.027	0.022	0.217	0.742	99.99999744	100	1.037		62.700							
MDD	Age at menarche	menarche	Steiger-filtered IVW	181	-0.030	0.012	0.013	0.048	99.99999744	100	1.037		62.700							
MDD	Age at menarche	menarche	MR Egger SIMEX		0.037	0.035	0.289	0.842	99.99999744	100	1.037		62.700							
MDD	Age at menarche	menarche	GSMR	170	-0.036	0.008	1.33E-05	6.24E-05												
MDD	Age at menarche	menarche	PRESSO	181	-0.034	0.010	0.001	0.005												
MDD	Age at menarche	menarche	LHC		-0.106	0.080	0.186	0.345												
MDD	Age at menopause	menopause	MR Egger	182	-0.008	0.006	0.199	0.864	100	100	1.028		101.492			8.08E-04	######	0.380		
MDD	Age at menopause	menopause	Weighted median	182	-0.006	0.005	0.182	0.460	100	100	1.028		101.492							
MDD	Age at menopause	menopause	Inverse variance weighte	182	-0.003	0.003	0.311	0.512	100	100	1.028	0.0936	101.492	0.966	unweighted				297.470	1.08E-07
MDD	Age at menopause	menopause	Simple mode	182	-0.011	0.010	0.278	0.916	100	100	1.028		101.492							
MDD	Age at menopause	menopause	Weighted mode	182	-0.008	0.005	0.094	0.641	100	100	1.028		101.492							
MDD	Age at menopause	menopause	Steiger-filtered IVW	182	-0.003	0.003	0.311	0.522	100	100	1.028		101.492							
MDD	Age at menopause	menopause	GSMR	175	-0.004	0.003	0.106	0.219												
MDD	Age at menopause	menopause	PRESSO	182	-0.002	0.003	0.426	0.578												
MDD	Age at menopause	menopause	LHC		0.044	0.017	0.008	0.028												
MDD	Age at smoking initiation	smokingage	MR Egger	10	0.844	0.763	0.301	0.864	18.52892204	83.41092336	1.285		37.353			-0.021	0.014	0.174		
MDD	Age at smoking initiation	smokingage	Weighted median	10	0.030	0.128	0.816	0.947	18.52892204	83.41092336	1.285		37.353							
MDD	Age at smoking initiation	smokingage	Inverse variance weighte	10	-0.268	0.180	0.137	0.298	18.52892204	83.41092336	1.285	0.0012	37.353	0.540	unweighted				38.923	1.19E-05
MDD	Age at smoking initiation	smokingage	Simple mode	10	0.055	0.170	0.753	0.975	18.52892204	83.41092336	1.285		37.353							
MDD	Age at smoking initiation	smokingage	Weighted mode	10	0.081	0.134	0.562	0.943	18.52892204	83.41092336	1.285		37.353							
MDD	Age at smoking initiation	smokingage	Steiger-filtered IVW	10	-0.268	0.180	0.137	0.298	18.52892204	83.41092336	1.285		37.353							
MDD	Age at smoking initiation	smokingage	GSMR	10	-0.187	0.091	0.040	0.100												
MDD	Age at smoking initiation	smokingage	PRESSO	10	-0.067	0.128	0.615	0.722												
MDD	Age at smoking initiation	smokingage	LHC		-0.156	0.092	0.088	0.196												
MDD	Age first birth	agefirstbirth	MR Egger	10	0.588	0.612	0.364	0.864	22.55375892	91.32081587	1.248		35.581			-0.018	0.014	0.234		
MDD	Age first birth	agefirstbirth	Weighted median	10	-0.204	0.135	0.129	0.339	22.55375892	91.32081587	1.248		35.581							
MDD	Age first birth	agefirstbirth	Inverse variance weighte	10	-0.135	0.251	0.589	0.806	22.55375892	91.32081587	1.248	0.0015	35.581	0.887	unweighted				133.296	2.49E-24
MDD	Age first birth	agefirstbirth	Simple mode	10	0.146	0.162	0.390	0.935	22.55375892	91.32081587	1.248		35.581							
MDD	Age first birth	agefirstbirth	Weighted mode	10	-0.038	0.111	0.739	0.967	22.55375892	91.32081587	1.248		35.581							
MDD	Age first birth	agefirstbirth	Steiger-filtered IVW	10	-0.135	0.251	0.589	0.806	22.55375892	91.32081587	1.248		35.581							
MDD	Age first birth	agefirstbirth	MR Egger SIMEX		0.665	0.659	0.342	0.842	22.55375892	91.32081587	1.248		35.581							
MDD	Age first birth	agefirstbirth	GSMR	5	-0.251	0.098	0.011	0.031												
MDD	Age first birth	agefirstbirth	PRESSO	10	-0.228	0.155	0.236	0.369												
MDD	Age first birth	agefirstbirth	LHC		-0.086	0.037	0.021	0.060												
MDD	Age last birth	agelastbirth	MR Egger	22	-0.002	0.061	0.976	0.980	42.05419128	99.80327434	1.164		28.685			-0.006	0.006	0.296		
MDD	Age last birth	agelastbirth	Weighted median	22	-0.050	0.016	0.002	0.013	42.05419128	99.80327434	1.164		28.685							
MDD	Age last birth	agelastbirth	Inverse variance weighte	22	-0.066	0.015	6.41E-06	5.41E-05	42.05419128	99.80327434	1.164	0.0032	28.685	0.232	unweighted				41.562	0.005
MDD	Age last birth	agelastbirth	Simple mode	22	-0.092	0.035	0.016	0.178	42.05419128	99.80327434	1.164		28.685							
MDD	Age last birth	agelastbirth	Weighted mode	22	-0.019	0.033	0.579	0.943	42.05419128	99.80327434	1.164		28.685							
MDD	Age last birth	agelastbirth	Steiger-filtered IVW	22	-0.066	0.015	6.41E-06	5.41E-05	42.05419128	99.80327434	1.164		28.685							
MDD	Age last birth	agelastbirth	GSMR	22	-0.058	0.011	8.62E-08	5.90E-07												
MDD	Age last birth	agelastbirth	PRESSO	22	-0.057	0.013	2.34E-04	0.001												
MDD	Age last birth	agelastbirth	LHC		-0.179	2.284	0.937	0.980												
MDD	Alcohol abuse	alcoholabuse18	MR Egger	24	-0.936	0.904	0.312	0.864	59.8529993	99.99812397	1.128		24.963			0.007	0.006	0.248		
MDD	Alcohol abuse	alcoholabuse18	Weighted median	24	0.009	0.256	0.972	0.995	59.8529993	99.99812397	1.128		24.963							
MDD	Alcohol abuse	alcoholabuse18	Inverse variance weighte	24	0.069	0.319	0.828	0.917	59.8529993	99.99812397	1.128	0.0050	24.963	0.721	unweighted				77.994	6.69E-08
MDD	Alcohol abuse	alcoholabuse18	Simple mode	24	-0.070	0.437	0.874	0.985	59.8529993	99.99812397	1.128		24.963							
MDD	Alcohol abuse	alcoholabuse18	Weighted mode	24	-0.092	0.387	0.815	0.967	59.8529993	99.99812397	1.128		24.963							
MDD	Alcohol abuse	alcoholabuse18	Steiger-filtered IVW	24	0.069	0.319	0.828	0.917	59.8529993	99.99812397	1.128		24.963							
MDD	Alcohol abuse	alcoholabuse18	MR Egger SIMEX		2.204	0.668	0.003	0.083	59.8529993	99.99812397	1.128		24.963							
MDD	Alcohol abuse	alcoholabuse18	GSMR	23	-0.137	0.182	0.452	0.632												
MDD	Alcohol abuse	alcoholabuse18	PRESSO	24	-0.068	0.196	0.731	0.783												
MDD	Alcohol abuse	alcoholabuse18	LHC		-0.283	0.144	0.049	0.134												
MDD	Alcohol dependence	alcoholdep	MR Egger	13	-0.014	0.102	0.893	0.980	74.20479175	99.99999123	1.108		25.065			0.001	0.005	0.787		
MDD	Alcohol dependence	alcoholdep	Weighted median	13	-0.012	0.050	0.819	0.947	74.20479175	99.99999123	1.108		25.065							
MDD	Alcohol dependence	alcoholdep	Inverse variance weighte	13	0.012	0.039	0.755	0.917	74.20479175	99.99999123	1.108	0.0070	25.065	0.749	unweighted				6.549	0.886
MDD	Alcohol dependence	alcoholdep	Simple mode	13	-0.015	0.085	0.860	0.981	74.20479175	99.99999123	1.108		25.065							
MDD	Alcohol dependence	alcoholdep	Weighted mode	13	-0.037	0.075	0.628	0.943	74.20479175	99.99999123	1.108		25.065							
MDD	Alcohol dependence	alcoholdep	Steiger-filtered IVW	13	0.012	0.039	0.755	0.917	74.20479175	99.99999123	1.108		25.065							
MDD	Alcohol dependence	alcoholdep	MR Egger SIMEX		-0.083	0.066	0.230	0.839	74.20479175	99.99999123	1.108		25.065							
MDD	Alcohol dependence	alcoholdep	GSMR	13	0.011	0.039	0.785	0.865												
MDD	Alcohol dependence	alcoholdep	LHC		-0.206	0.339	0.543	0.738												
MDD	Alcohol per week	alcoholweek	MR Egger	75	-0.116	0.287	0.687	0.980	58.89425714	99.99748466	1.13		43.680			0.003	0.004	0.395		
MDD	Alcohol per week	alcoholweek	Weighted median	75	0.094	0.077	0.224	0.540	58.89425714	99.99748466	1.13		43.680							
MDD	Alcohol per week	alcoholweek	Inverse variance weighte	75	0.118	0.085	0.165	0.333	58.89425714	99.99748466	1.13	0.0049	43.680	0.734	unweighted				327.847	1.18E-33
MDD	Alcohol per week	alcoholweek	Simple mode	75	0.096	0.174	0.583	0.975	58.89425714	99.99748466	1.13		43.680							
MDD	Alcohol per week	alcoholweek	Weighted mode	75	0.096	0.140	0.497	0.943	58.89425714	99.99748466	1.13		43.680							
MDD	Alcohol per week	alcoholweek	Steiger-filtered IVW	75	0.118	0.085	0.165	0.333	58.89425714	99.99748466	1.13		43.680							
MDD	Alcohol per week	alcoholweek	MR Egger SIMEX		0.120	0.357	0.737	0.897	58.89425714	99.99748466	1.13		43.680							
MDD	Alcohol per week	alcoholweek	GSMR	63	0.160	0.046	5.53E-04	0.002												
MDD	Alcohol per week	alcoholweek	PRESSO	75	0.145	0.067	0.034	0.079												
MDD	Alcohol per week	alcoholweek	LHC		0.403	0.139	0.004	0.017												
MDD	Alzheimer's disease	alzheimer	MR Egger	11	-0.006	0.045	0.898	0.980	96.55048512	100	1.073		72.722			4.89E-04	0.007	0.948		
MDD	Alzheimer's disease	alzheimer	Weighted median	11	0.000	0.012	0.982	0.995	96.55048512	100	1.073		72.722							
MDD	Alzheimer's disease	alzheimer	Inverse variance weighte	11	-0.003	0.013	0.821	0.917	96.55048512	100	1.073	0.0147	72.722	0.876	weighted				23.409	0.009
MDD	Alzheimer's disease	alzheimer	Simple mode	11	0.000	0.021	0.984	0.995	96.55048512	100	1.073		72.722							
MDD	Alzheimer's disease	alzheimer	Weighted mode	11	-0.006	0.013	0.640	0.943	96.55048512	100	1.073		72.722							
MDD	Alzheimer's disease	alzheimer	Steiger-filtered IVW	11	-0.003	0.013	0.821	0.917	96.55048512	100	1.073		72.722							
MDD	Alzheimer's disease	alzheimer	MR Egger SIMEX		-0.006	0.049	0.909	0.909	96.55048512	100	1.073		72.722							
MDD	Alzheimer's disease	alzheimer	GSMR	11	-0.003	0.009	0.751	0.865												
MDD	Alzheimer's disease	alzheimer	LHC		0.041	0.045	0.365	0.586												
MDD	Amyotrophic lateral sclerosis	als	MR Egger	8	-0.034	0.040	0.427	0.878	59.26980264	99.99775597	1.129		49.871			0.006	0.005	0.327		
MDD	Amyotrophic lateral sclerosis	als	Weighted median	8	0.012	0.023	0.604	0.868	59.26980264	99.99775597	1.129		49.871							
MDD	Amyotrophic lateral sclerosis	als	Inverse variance weighte	8	0.005	0.017	0.785	0.917	59.26980264	99.99775597	1.129	0.0049	49.871	0.896	unweighted				6.739	0.457
MDD	Amyotrophic lateral sclerosis	als	Simple mode	8	0.027	0.034	0.449	0.935	59.26980264	99.99775597	1.129		49.871							
MDD	Amyotrophic lateral sclerosis	als	Weighted mode	8	0.012	0.028	0.691	0.943	59.26980264	99.99775597	1.129		49.871							
MDD	Amyotrophic lateral sclerosis	als	Steiger-filtered IVW	8	0.005	0.017	0.785	0.917	59.26980264	99.99775597	1.129		49.871							
MDD	Amyotrophic lateral sclerosis	als	MR Egger SIMEX		0.019	0.023	0.450	0.842	59.26980264	99.99775597	1.129		49.871							
MDD	Amyotrophic lateral sclerosis	als	GSMR	8	0.005	0.017	0.785	0.865												

MDD	Amyotrophic lateral sclerosis	als	LHC		0.048	0.201	0.811	0.933												
MDD	Asthma	asthma	MR Egger	20	-0.002	0.052	0.963	0.980	92.3129056	100	1.082		57.531			0.001	0.007	0.839		
MDD	Asthma	asthma	Weighted median	20	0.007	0.015	0.646	0.898	92.3129056	100	1.082		57.531							
MDD	Asthma	asthma	Inverse variance weighte	20	0.008	0.012	0.510	0.733	92.3129056	100	1.082	0.0118	57.531	0.710	unweighted				33.495	0.021
MDD	Asthma	asthma	Simple mode	20	-0.015	0.034	0.671	0.975	92.3129056	100	1.082		57.531							
MDD	Asthma	asthma	Weighted mode	20	0.043	0.031	0.175	0.704	92.3129056	100	1.082		57.531							
MDD	Asthma	asthma	Steiger-filtered IVW	20	0.008	0.012	0.510	0.733	92.3129056	100	1.082		57.531							
MDD	Asthma	asthma	MR Egger SIMEX		-0.157	0.068	0.033	0.264	92.3129056	100	1.082		57.531							
MDD	Asthma	asthma	GSMR	20	0.008	0.009	0.382	0.576												
MDD	Asthma	asthma	LHC		-0.048	0.071	0.498	0.710												
MDD	Atrial Fibrillation	atrialfib	MR Egger	111	0.003	0.019	0.855	0.980	83.79196695	99.99999996	1.095		82.779			5.42E-05	0.001	0.968		
MDD	Atrial Fibrillation	atrialfib	Weighted median	111	0.009	0.012	0.458	0.798	83.79196695	99.99999996	1.095		82.779							
MDD	Atrial Fibrillation	atrialfib	Inverse variance weighte	111	0.004	0.008	0.629	0.836	83.79196695	99.99999996	1.095	0.0089	82.779	0.942	unweighted				176.480	5.95E-05
MDD	Atrial Fibrillation	atrialfib	Simple mode	111	0.062	0.027	0.021	0.188	83.79196695	99.99999996	1.095		82.779							
MDD	Atrial Fibrillation	atrialfib	Weighted mode	111	0.006	0.014	0.660	0.943	83.79196695	99.99999996	1.095		82.779							
MDD	Atrial Fibrillation	atrialfib	Steiger-filtered IVW	111	0.004	0.008	0.629	0.836	83.79196695	99.99999996	1.095		82.779							
MDD	Atrial Fibrillation	atrialfib	GSMR	108	0.006	0.007	0.403	0.588												
MDD	Atrial Fibrillation	atrialfib	PRESSO	111	0.007	0.008	0.377	0.547												
MDD	Atrial Fibrillation	atrialfib	LHC		0.130	0.062	0.034	0.096												
MDD	BMI	BMI	MR Egger	499	0.115	0.060	0.054	0.605	99.99994564	100	1.04		70.414			3.07E-04	######	0.746		
MDD	BMI	BMI	Weighted median	499	0.112	0.024	3.43E-06	4.36E-05	99.99994564	100	1.04		70.414							
MDD	BMI	BMI	Inverse variance weighte	499	0.133	0.021	2.28E-10	2.89E-09	99.99994564	100	1.04	0.0480	70.414	0.888	unweighted				1205.020	1.90E-60
MDD	BMI	BMI	Simple mode	499	0.089	0.100	0.374	0.935	99.99994564	100	1.04		70.414							
MDD	BMI	BMI	Weighted mode	499	0.081	0.063	0.204	0.725	99.99994564	100	1.04		70.414							
MDD	BMI	BMI	Steiger-filtered IVW	499	0.133	0.021	2.28E-10	2.89E-09	99.99994564	100	1.04		70.414							
MDD	BMI	BMI	MR Egger SIMEX		-0.029	0.062	0.640	0.897	99.99994564	100	1.04		70.414							
MDD	BMI	BMI	GSMR	476	0.127	0.014	3.19E-19	4.06E-18												
MDD	BMI	BMI	PRESSO	499	0.121	0.020	2.23E-09	2.72E-08												
MDD	BMI	BMI	LHC		-0.177	0.063	0.005	0.020												
MDD	Cholesterol	cholesterol	MR Egger	426	-0.029	0.024	0.231	0.864	99.99999917	100	1.036		182.599			0.000	######	0.904		
MDD	Cholesterol	cholesterol	Weighted median	426	-0.039	0.019	0.045	0.159	99.99999917	100	1.036		182.599							
MDD	Cholesterol	cholesterol	Inverse variance weighte	426	-0.031	0.015	0.038	0.122	99.99999917	100	1.036	0.0594	182.599	0.986	unweighted				750.990	1.99E-20
MDD	Cholesterol	cholesterol	Simple mode	426	-0.037	0.045	0.414	0.935	99.99999917	100	1.036		182.599							
MDD	Cholesterol	cholesterol	Weighted mode	426	-0.037	0.024	0.118	0.704	99.99999917	100	1.036		182.599							
MDD	Cholesterol	cholesterol	Steiger-filtered IVW	426	-0.031	0.015	0.038	0.122	99.99999917	100	1.036		182.599							
MDD	Cholesterol	cholesterol	GSMR	411	-0.022	0.012	0.053	0.124												
MDD	Cholesterol	cholesterol	PRESSO	426	-0.025	0.014	0.084	0.151												
MDD	Cholesterol	cholesterol	LHC		-0.037	0.059	0.523	0.722												
MDD	Chronic obstructive pulmonary dis	copd	MR Egger	8	-0.026	0.064	0.700	0.980	52.30034546	99.98372575	1.142		67.542			0.002	0.009	0.818		
MDD	Chronic obstructive pulmonary dis	copd	Weighted median	8	-0.006	0.017	0.735	0.947	52.30034546	99.98372575	1.142		67.542							
MDD	Chronic obstructive pulmonary dis	copd	Inverse variance weighte	8	-0.011	0.016	0.478	0.709	52.30034546	99.98372575	1.142	0.0042	67.542	0.813	unweighted				13.416	0.063
MDD	Chronic obstructive pulmonary dis	copd	Simple mode	8	0.027	0.035	0.475	0.961	52.30034546	99.98372575	1.142		67.542							
MDD	Chronic obstructive pulmonary dis	copd	Weighted mode	8	0.028	0.036	0.471	0.943	52.30034546	99.98372575	1.142		67.542							
MDD	Chronic obstructive pulmonary dis	copd	Steiger-filtered IVW	8	-0.011	0.016	0.478	0.709	52.30034546	99.98372575	1.142		67.542							
MDD	Chronic obstructive pulmonary dis	copd	MR Egger SIMEX		-0.024	0.072	0.753	0.897	52.30034546	99.98372575	1.142		67.542							
MDD	Chronic obstructive pulmonary dis	copd	GSMR	8	-0.010	0.011	0.359	0.551												
MDD	Chronic obstructive pulmonary dis	copd	LHC		-0.092	0.112	0.412	0.634												
MDD	Chronotype	chronotype	MR Egger	149	-0.281	0.109	0.011	0.235	97.55736342	100	1.07		47.873			0.005	0.002	0.030		
MDD	Chronotype	chronotype	Weighted median	149	-0.049	0.031	0.122	0.329	97.55736342	100	1.07		47.873							
MDD	Chronotype	chronotype	Inverse variance weighte	149	-0.055	0.034	0.108	0.247	97.55736342	100	1.07	0.0159	47.873	0.793	unweighted				472.862	1.28E-35
MDD	Chronotype	chronotype	Simple mode	149	-0.056	0.088	0.523	0.975	97.55736342	100	1.07		47.873							
MDD	Chronotype	chronotype	Weighted mode	149	-0.056	0.073	0.440	0.943	97.55736342	100	1.07		47.873							
MDD	Chronotype	chronotype	Steiger-filtered IVW	149	-0.055	0.034	0.108	0.247	97.55736342	100	1.07		47.873							
MDD	Chronotype	chronotype	MR Egger SIMEX		-0.044	0.072	0.544	0.842	97.55736342	100	1.07		47.873							
MDD	Chronotype	chronotype	GSMR	137	-0.048	0.020	0.018	0.052												
MDD	Chronotype	chronotype	PRESSO	149	-0.056	0.028	0.047	0.096												
MDD	Chronotype	chronotype	LHC		0.184	0.143	0.197	0.358												
MDD	Cigarettes per day	cigperday	MR Egger	37	-0.130	0.118	0.278	0.864	78.90799287	99.99999918	1.101		68.964			0.007	0.003	0.023		
MDD	Cigarettes per day	cigperday	Weighted median	37	-0.029	0.047	0.533	0.832	78.90799287	99.99999918	1.101		68.964							
MDD	Cigarettes per day	cigperday	Inverse variance weighte	37	0.108	0.066	0.102	0.241	78.90799287	99.99999918	1.101	0.0078	68.964	0.945	unweighted				150.879	5.18E-16
MDD	Cigarettes per day	cigperday	Simple mode	37	0.021	0.115	0.857	0.981	78.90799287	99.99999918	1.101		68.964							
MDD	Cigarettes per day	cigperday	Weighted mode	37	-0.023	0.045	0.608	0.943	78.90799287	99.99999918	1.101		68.964							
MDD	Cigarettes per day	cigperday	Steiger-filtered IVW	37	0.108	0.066	0.102	0.241	78.90799287	99.99999918	1.101		68.964							
MDD	Cigarettes per day	cigperday	GSMR	32	0.048	0.034	0.162	0.301												
MDD	Cigarettes per day	cigperday	PRESSO	37	0.178	0.071	0.018	0.048												
MDD	Cigarettes per day	cigperday	LHC		0.223	0.084	0.008	0.028												
MDD	Coffee consumption	coffee	MR Egger	16	-0.113	0.111	0.329	0.864	72.01731776	99.99997686	1.111		26.581			0.010	0.009	0.290		
MDD	Coffee consumption	coffee	Weighted median	16	3.38E-05	0.025	0.999	0.999	72.01731776	99.99997686	1.111		26.581							
MDD	Coffee consumption	coffee	Inverse variance weighte	16	0.006	0.028	0.825	0.917	72.01731776	99.99997686	1.111	0.0066	26.581	0.525	unweighted				37.175	0.001
MDD	Coffee consumption	coffee	Simple mode	16	-0.004	0.038	0.909	0.987	72.01731776	99.99997686	1.111		26.581							
MDD	Coffee consumption	coffee	Weighted mode	16	-0.017	0.037	0.650	0.943	72.01731776	99.99997686	1.111		26.581							
MDD	Coffee consumption	coffee	Steiger-filtered IVW	16	0.006	0.028	0.825	0.917	72.01731776	99.99997686	1.111		26.581							
MDD	Coffee consumption	coffee	GSMR	16	4.38E-04	0.018	0.981	0.981												
MDD	Coffee consumption	coffee	PRESSO	16	6.54E-04	0.018	0.971	0.971												
MDD	Coffee consumption	coffee	LHC		0.264	0.100	0.008	0.028												
MDD	Coronary artery disease	coronaryartdis	MR Egger	172	-0.002	0.022	0.944	0.980	90.94926747	100	1.084		75.346			8.24E-04	0.001	0.483		
MDD	Coronary artery disease	coronaryartdis	Weighted median	172	-0.006	0.013	0.616	0.870	90.94926747	100	1.084		75.346							
MDD	Coronary artery disease	coronaryartdis	Inverse variance weighte	172	0.012	0.011	0.267	0.476	90.94926747	100	1.084	0.0112	75.346	0.942	unweighted				365.159	3.81E-16
MDD	Coronary artery disease	coronaryartdis	Simple mode	172	-0.054	0.032	0.092	0.509	90.94926747	100	1.084		75.346							
MDD	Coronary artery disease	coronaryartdis	Weighted mode	172	-0.018	0.022	0.413	0.943	90.94926747	100	1.084		75.346							
MDD	Coronary artery disease	coronaryartdis	Steiger-filtered IVW	171	0.010	0.010	0.353	0.561	90.94926747	100	1.084		75.346							
MDD	Coronary artery disease	coronaryartdis	GSMR	162	-0.001	0.008	0.941	0.960												
MDD	Coronary artery disease	coronaryartdis	PRESSO	172	0.008	0.009	0.339	0.504												
MDD	Coronary artery disease	coronaryartdis	LHC		0.088	0.046	0.057	0.139												
MDD	Crohn's disease	crohndis	MR Egger	81	-0.006	0.012	0.617	0.980	100	100	1.022		78.662			0.001	0.002	0.596		
MDD	Crohn's disease	crohndis	Weighted median	81	0.003	0.005	0.483	0.798	100	100	1.022		78.662							
MDD	Crohn's disease	crohndis	Inverse variance weighte	81	0.000	0.004	0.987	0.999	100	100	1.022	0.1577	78.662	0.910	unweighted				139.748	4.09E-05
MDD	Crohn's disease	crohndis	Simple mode	81	0.002	0.010	0.839	0.981	100	100	1.022		78.662							
MDD	Crohn's disease	crohndis	Weighted mode	81	7.54E-04	0.007	0.916	0.967	100	100	1.022		78.662							
MDD	Crohn's disease	crohndis	Steiger-filtered IVW	81	0.000	0.004	0.987	0.999	100	100	1.022		78.662							
MDD	Crohn's disease	crohndis	GSMR	79	0.003	0.003	0.328	0.521												
MDD	Crohn's disease	crohndis	PRESSO	81	0.003	0.004	0.492	0.625												
MDD	Crohn's disease	crohndis	LHC		0.010	0.013	0.434	0.639												
MDD	CRP levels	crp	MR Egger	238	-0.011	0.033	0.746	0.980	99.99998564	100	1.038		125.013			8.61E-04	######	0.324		
MDD	CRP levels	crp	Weighted median	238	-0.013	0.023	0.562	0.847	99.99998564	100	1.038		125.013							
MDD	CRP levels	crp	Inverse variance weighte	238	0.015	0.020	0.431	0.661	99.99998564	100	1.038	0.0516	125.013	0.977	unweighted				592.463	2.23E-32
MDD	CRP levels	crp	Simple mode	238	0.086	0.050	0.084	0.509	99.99998564	100	1.038		125.013							
MDD	CRP levels	crp	Weighted mode	238	-0.025	0.025	0.321	0.933	99.99998564	100	1.038		125.013							
MDD	CRP levels	crp	Steiger-filtered IVW	238	0.015	0.020	0.431	0.661	99.99998564	100	1.038		125.013							
MDD	CRP levels	crp	GSMR	221	0.004	0.013	0.727	0.863												
MDD	CRP levels	crp	PRESSO	238	0.008	0.017	0.642	0.725												
MDD	CRP levels	crp	LHC		0.059	0.031	0.055	0.139												
MDD	Diastolic blood pressure	diabloodpres	MR Egger	476	1.15E-04	0.005	0.980	0.980	99.99733787	100	1.046		82.216			7.27E-04	######	0.302		
MDD	Diastolic blood pressure	diabloodpres	Weighted median	476	0.002	0.003	0.345	0.731	99.99733787	100	1.046		82.216							
MDD	Diastolic blood pressure	diabloodpres	Inverse variance weighte	476	0.004	0.002	0.020	0.068	99.99733787	100	1.046	0.0370	82.216	0.929	unweighted				800.019	5.83E-19
MDD	Diastolic blood pressure	diabloodpres	Simple mode	476	0.004	0.008	0.638	0.975	99.99733787	100	1.046		82.216							
MDD	Diastolic blood pressure	diabloodpres	Weighted mode	476	7.55E-04	0.006	0.896	0.967	99.99733787	100	1.046		82.216							
MDD	Diastolic blood pressure	diabloodpres	Steiger-filtered IVW	476	0.004	0.002	0.020	0.068	99.99733787	100	1.046		82.216							
MDD	Diastolic blood pressure	diabloodpres	GSMR	458	0.003	0.001	0.033	0.084												
MDD	Diastolic blood pressure	diabloodpres	PRESSO	476	0.005	0.002	0.009	0.026												
MDD	Diastolic blood pressure	diabloodpres	LHC		-0.154	0.029	1.29E-07	9.36E-07												
MDD	Educational attainment	education	MR Egger	484	-0.402	0.097	3.99E-05	0.004	99.99424035	100	1.047		54.929			0.001	0.001	0.312		
MDD	Educational attainment	education	Weighted median	484	-0.284	0.029	2.64E-23	2.35E-21	99.99424035	100	1.047		54.929							
MDD	Educational attainment	education	Inverse variance weighte	484	-0.308	0.027	6.82E-31	6.07E-29	99.99424035	100	1.047	0.0347	54.929	0.759	unweighted				1305.105	8.20E-77
MDD	Educational attainment	education	Simple mode	484	-0.394	0.132	0.003	0.064	99.99424035	100	1.047		54.929							
MDD	Educational attainment	education	Weighted mode	484	-0.435	0.133	0.001	0.052	99.99424035	100	1.047		54.929							
MDD	Educational attainment	education	Steiger-filtered IVW	484	-0.308	0.027	6.82E-31	6.07E-29	99.99424035	100	1.047		54.929							
MDD	Educational attainment	education	MR Egger SIMEX		-0.109	0.106	0.304	0.842	99.99424035	100	1.047		54.929							
MDD	Educational attainment	education	GSMR	459	-0.275	0.017	9.50E-58	2.82E-56												
MDD	Educational attainment	education	PRESSO	484	-0.302	0.023	1.19E-32	7.27E-31												
MDD	Educational attainment	education	LHC		-0.214	0.082	0.009	0.032												
MDD	Erectile dysfunction	erectile	MR Egger	8	-0.154	0.058	0.037	0.605	16.02109177	75.81018279	1.318		26.831			0.030	0.010	0.022		
MDD	Erectile dysfunction	erectile	Weighted median	8	-0.002	0.022	0.926	0.993	16.02109177	75.81018279	1.318		26.831							
MDD	Erectile dysfunction	erectile	Inverse variance weighte	8	0.011	0.031	0.709	0.915	16.02109177	75.81018279	1.318	9.59E-04	26.831	0.759	unweighted				27.165	3.11E-04
MDD	Erectile dysfunction	erectile	Simple mode	8	-0.024	0.030	0.452	0.935	16.02109177	75.81018279	1.318		26.831							
MDD	Erectile dysfunction	erectile	Weighted mode	8	0.009	0.025	0.732	0.967	16.02109177	75.81018279	1.318		26.831							
MDD	Erectile dysfunction	erectile	Steiger-filtered IVW	8	0.011	0.031	0.709	0.915	16.02109177	75.81018279	1.318		26.831							
MDD	Erectile dysfunction	erectile	MR Egger SIMEX		-0.057	0.073	0.464	0.842	16.02109177	75.81018279	1.318		26.831							
MDD	Erectile dysfunction	erectile	GSMR	7	-0.012	0.017	0.485	0.635												
MDD	Erectile dysfunction	erectile	LHC		0.217	4.256	0.959	0.980												
MDD	Executive function	executivefun	MR Egger	75	-0.547	0.583	0.351	0.864	76.08937413	99.99999643	1.105		41.659			3.95E-05	0.005	0.993		
MDD	Executive function	executivefun	Weighted median	75	-0.514	0.117	1.05E-05	1.04E-04	76.08937413	99.99999643	1.105		41.659							
MDD	Executive function	executivefun	Inverse variance weighte	75	-0.543	0.121	6.69E-06	5.41E-05	76.08937413	99.99999643	1.105	0.0073	41.659	0.469	unweighted				232.207	3.16E-18
MDD	Executive function	executivefun	Simple mode	75	-0.880	0.364	0.018	0.178	76.08937413	99.99999643	1.105		41.659							
MDD	Executive function	executivefun	Weighted mode	75	-0.730	0.292	0.015	0.186	76.08937413	99.99999643	1.105		41.659							
MDD	Executive function	executivefun	Steiger-filtered IVW	75	-0.543	0.121	6.69E-06	5.41E-05	76.08937413	99.99999643	1.105		41.659							
MDD	Executive function	executivefun	GSMR	72	-0.402	0.071	1.78E-08	1.44E-07												
MDD	Executive function	executivefun	PRESSO	75	-0.442	0.108	1.16E-04	5.52E-04												
MDD	Executive function	executivefun	LHC		-0.292	0.122	0.017	0.052												
MDD	Exercise (self report)	exercise	MR Egger	14	0.633	0.503	0.232	0.864	14.93062553	71.70409904	1.337		37.831			-0.024	0.012	0.083		
MDD	Exercise (self report)	exercise	Weighted median	14	-0.155	0.074	0.037	0.137	14.93062553	71.70409904	1.337		37.831							
MDD	Exercise (self report)	exercise	Inverse variance weighte	14	-0.298	0.108	0.006	0.027	14.93062553	71.70409904	1.337	8.70E-04	37.831	0.389	unweighted				64.818	7.11E-09
MDD	Exercise (self report)	exercise	Simple mode	14	-0.174	0.105	0.121	0.585	14.93062553	71.70409904	1.337		37.831							
MDD	Exercise (self report)	exercise	Weighted mode	14	-0.130	0.082	0.137	0.704	14.93062553	71.70409904	1.337		37.831							
MDD	Exercise (self report)	exercise	Steiger-filtered IVW	14	-0.298	0.108	0.006	0.027	14.93062553	71.70409904	1.337		37.831							
MDD	Exercise (self report)	exercise	GSMR	12	-0.174	0.053	0.001	0.004												
MDD	Exercise (self report)	exercise	PRESSO	14	-0.238	0.067	0.005	0.019												
MDD	Exercise (self report)	exercise	LHC		-0.281	0.429	0.512	0.718												
MDD	Externalizing	externalizing	MR Egger	175	0.563	0.274	0.041	0.605	79.09060126	99.99999926	1.101		47.779			0.001	0.003	0.700		

MDD	Externalizing	externalizing	Weighted median	175	0.582	0.060	4.02E-22	1.79E-20	79.09060126	99.99999926	1.101		47.779							
MDD	Externalizing	externalizing	Inverse variance weighte	175	0.666	0.065	6.65E-25	2.96E-23	79.09060126	99.99999926	1.101	0.0080	47.779	0.619	unweighted				640.762	1.25E-54
MDD	Externalizing	externalizing	Simple mode	175	0.642	0.208	0.002	0.064	79.09060126	99.99999926	1.101		47.779							
MDD	Externalizing	externalizing	Weighted mode	175	0.613	0.225	0.007	0.127	79.09060126	99.99999926	1.101		47.779							
MDD	Externalizing	externalizing	Steiger-filtered IVW	175	0.666	0.065	6.65E-25	2.96E-23	79.09060126	99.99999926	1.101		47.779							
MDD	Externalizing	externalizing	MR Egger SIMEX		-0.711	0.412	0.086	0.431	79.09060126	99.99999926	1.101		47.779							
MDD	Externalizing	externalizing	GSMR	161	0.594	0.037	2.87E-59	1.28E-57												
MDD	Externalizing	externalizing	PRESSO	175	0.699	0.055	3.77E-26	1.15E-24												
MDD	Externalizing	externalizing	LHC		0.223	0.089	0.012	0.039												
MDD	Financial difficulties	financialdiff	MR Egger	7	-0.152	0.192	0.464	0.898	10.39987419	48.77775801	1.464		32.663			0.018	0.009	0.108		
MDD	Financial difficulties	financialdiff	Weighted median	7	0.209	0.062	6.90E-04	0.005	10.39987419	48.77775801	1.464		32.663							
MDD	Financial difficulties	financialdiff	Inverse variance weighte	7	0.209	0.064	0.001	0.006	10.39987419	48.77775801	1.464	5.04E-04	32.663	0.659	unweighted				14.276	0.027
MDD	Financial difficulties	financialdiff	Simple mode	7	0.316	0.115	0.034	0.250	10.39987419	48.77775801	1.464		32.663							
MDD	Financial difficulties	financialdiff	Weighted mode	7	0.297	0.119	0.047	0.378	10.39987419	48.77775801	1.464		32.663							
MDD	Financial difficulties	financialdiff	Steiger-filtered IVW	7	0.209	0.064	0.001	0.006	10.39987419	48.77775801	1.464		32.663							
MDD	Financial difficulties	financialdiff	MR Egger SIMEX		-0.103	0.166	0.563	0.842	10.39987419	48.77775801	1.464		32.663							
MDD	Financial difficulties	financialdiff	GSMR	7	0.190	0.045	2.05E-05	8.71E-05												
MDD	Financial difficulties	financialdiff	LHC		0.770	0.513	0.133	0.270												
MDD	Fluid intelligence	fluidintelligence	MR Egger	15	-0.124	0.112	0.289	0.864	40.34804711	99.71335671	1.168		22.790			0.007	0.006	0.268		
MDD	Fluid intelligence	fluidintelligence	Weighted median	15	-0.040	0.039	0.301	0.653	40.34804711	99.71335671	1.168		22.790							
MDD	Fluid intelligence	fluidintelligence	Inverse variance weighte	15	3.91E-05	0.033	0.999	0.999	40.34804711	99.71335671	1.168	0.0030	22.790	0.517	unweighted				20.701	0.110
MDD	Fluid intelligence	fluidintelligence	Simple mode	15	-0.062	0.064	0.353	0.935	40.34804711	99.71335671	1.168		22.790							
MDD	Fluid intelligence	fluidintelligence	Weighted mode	15	-0.065	0.067	0.347	0.933	40.34804711	99.71335671	1.168		22.790							
MDD	Fluid intelligence	fluidintelligence	Steiger-filtered IVW	15	3.91E-05	0.033	0.999	0.999	40.34804711	99.71335671	1.168		22.790							
MDD	Fluid intelligence	fluidintelligence	GSMR	15	-0.004	0.028	0.878	0.942												
MDD	Fluid intelligence	fluidintelligence	LHC		-0.200	0.085	0.018	0.055												
MDD	Gastroesophageal reflux disease	gerd	MR Egger	20	0.386	0.425	0.376	0.864	29.1653395	97.29017918	1.208		34.022			-0.008	0.016	0.627		
MDD	Gastroesophageal reflux disease	gerd	Weighted median	20	0.130	0.047	0.006	0.029	29.1653395	97.29017918	1.208		34.022							
MDD	Gastroesophageal reflux disease	gerd	Inverse variance weighte	20	0.179	0.067	0.007	0.033	29.1653395	97.29017918	1.208	0.0020	34.022	######	unweighted				107.964	1.89E-14
MDD	Gastroesophageal reflux disease	gerd	Simple mode	20	0.124	0.079	0.131	0.585	29.1653395	97.29017918	1.208		34.022							
MDD	Gastroesophageal reflux disease	gerd	Weighted mode	20	0.113	0.073	0.139	0.704	29.1653395	97.29017918	1.208		34.022							
MDD	Gastroesophageal reflux disease	gerd	Steiger-filtered IVW	20	0.179	0.067	0.007	0.033	29.1653395	97.29017918	1.208		34.022							
MDD	Gastroesophageal reflux disease	gerd	GSMR	17	0.179	0.032	2.23E-08	1.65E-07												
MDD	Gastroesophageal reflux disease	gerd	PRESSO	20	0.225	0.042	8.11E-05	4.50E-04												
MDD	Gastroesophageal reflux disease	gerd	LHC		0.247	0.056	9.04E-06	5.62E-05												
MDD	Generalized epilepsy	epilepsy	MR Egger	18	0.077	0.209	0.719	0.980	99.97998712	100	1.05		39.996			-0.005	0.013	0.727		
MDD	Generalized epilepsy	epilepsy	Weighted median	18	-0.017	0.027	0.531	0.832	99.97998712	100	1.05		39.996							
MDD	Generalized epilepsy	epilepsy	Inverse variance weighte	18	0.003	0.027	0.915	0.958	99.97998712	100	1.05	0.0311	39.996	######	unweighted				41.202	8.73E-04
MDD	Generalized epilepsy	epilepsy	Simple mode	18	-0.033	0.056	0.567	0.975	99.97998712	100	1.05		39.996							
MDD	Generalized epilepsy	epilepsy	Weighted mode	18	-0.031	0.053	0.561	0.943	99.97998712	100	1.05		39.996							
MDD	Generalized epilepsy	epilepsy	Steiger-filtered IVW	18	0.003	0.027	0.915	0.958	99.97998712	100	1.05		39.996							
MDD	Generalized epilepsy	epilepsy	GSMR	18	0.001	0.018	0.942	0.960												
MDD	Generalized epilepsy	epilepsy	LHC		0.078	0.009	3.17E-19	5.52E-18												
MDD	Heart failure	heartfail	MR Egger	10	-0.082	0.125	0.528	0.940	9.368891613	42.31513301	1.516		40.440			0.006	0.008	0.480		
MDD	Heart failure	heartfail	Weighted median	10	-0.020	0.037	0.587	0.856	9.368891613	42.31513301	1.516		40.440							
MDD	Heart failure	heartfail	Inverse variance weighte	10	0.005	0.040	0.901	0.958	9.368891613	42.31513301	1.516	4.23E-04	40.440	0.799	unweighted				20.715	0.014
MDD	Heart failure	heartfail	Simple mode	10	-0.060	0.055	0.309	0.935	9.368891613	42.31513301	1.516		40.440							
MDD	Heart failure	heartfail	Weighted mode	10	-0.023	0.053	0.673	0.943	9.368891613	42.31513301	1.516		40.440							
MDD	Heart failure	heartfail	Steiger-filtered IVW	10	0.005	0.040	0.901	0.958	9.368891613	42.31513301	1.516		40.440							
MDD	Heart failure	heartfail	MR Egger SIMEX		0.062	0.070	0.396	0.842	9.368891613	42.31513301	1.516		40.440							
MDD	Heart failure	heartfail	GSMR	10	-0.002	0.027	0.950	0.960												
MDD	Heart failure	heartfail	PRESSO	10	-0.023	0.027	0.408	0.578												
MDD	Heart failure	heartfail	LHC		0.654	0.363	0.071	0.168												
MDD	Heart rate variability	heartvaria	MR Egger	17	0.089	0.103	0.397	0.864	99.87232857	100	1.055		39.773			-0.001	0.004	0.732		
MDD	Heart rate variability	heartvaria	Weighted median	17	0.034	0.048	0.484	0.798	99.87232857	100	1.055		39.773							
MDD	Heart rate variability	heartvaria	Inverse variance weighte	17	0.057	0.042	0.175	0.338	99.87232857	100	1.055	0.0254	39.773	0.862	unweighted				28.152	0.030
MDD	Heart rate variability	heartvaria	Simple mode	17	-0.084	0.080	0.306	0.935	99.87232857	100	1.055		39.773							
MDD	Heart rate variability	heartvaria	Weighted mode	17	0.003	0.056	0.956	0.968	99.87232857	100	1.055		39.773							
MDD	Heart rate variability	heartvaria	Steiger-filtered IVW	17	0.057	0.042	0.175	0.338	99.87232857	100	1.055		39.773							
MDD	Heart rate variability	heartvaria	MR Egger SIMEX		0.023	0.084	0.785	0.897	99.87232857	100	1.055		39.773							
MDD	Heart rate variability	heartvaria	GSMR	16	0.024	0.034	0.473	0.633												
MDD	Heart rate variability	heartvaria	PRESSO	17	0.029	0.038	0.454	0.589												
MDD	Heart rate variability	heartvaria	LHC		-0.139	0.038	2.34E-04	0.001												
MDD	High density lipoprotein	hdl	MR Egger	478	0.009	0.023	0.684	0.980	99.99999999	100	1.033		181.469			-0.002	######	0.003		
MDD	High density lipoprotein	hdl	Weighted median	478	-0.012	0.019	0.529	0.832	99.99999999	100	1.033		181.469							
MDD	High density lipoprotein	hdl	Inverse variance weighte	478	-0.041	0.016	0.008	0.034	99.99999999	100	1.033	0.0703	181.469	0.988	unweighted				1055.315	9.91E-46
MDD	High density lipoprotein	hdl	Simple mode	478	-0.047	0.038	0.226	0.804	99.99999999	100	1.033		181.469							
MDD	High density lipoprotein	hdl	Weighted mode	478	-0.023	0.017	0.171	0.704	99.99999999	100	1.033		181.469							
MDD	High density lipoprotein	hdl	Steiger-filtered IVW	478	-0.041	0.016	0.008	0.034	99.99999999	100	1.033		181.469							
MDD	High density lipoprotein	hdl	GSMR	442	-0.032	0.011	0.003	0.011												
MDD	High density lipoprotein	hdl	PRESSO	478	-0.030	0.014	0.032	0.078												
MDD	High density lipoprotein	hdl	LHC		0.025	0.024	0.314	0.526												
MDD	HIV susceptibility	hivsuscep	MR Egger	16	0.017	0.046	0.715	0.980	12.30708199	59.59033539	1.396		24.438			0.001	0.004	0.715		
MDD	HIV susceptibility	hivsuscep	Weighted median	16	0.015	0.027	0.583	0.856	12.30708199	59.59033539	1.396		24.438							
MDD	HIV susceptibility	hivsuscep	Inverse variance weighte	16	0.032	0.021	0.123	0.273	12.30708199	59.59033539	1.396	6.58E-04	24.438	0.814	weighted				18.802	0.223
MDD	HIV susceptibility	hivsuscep	Simple mode	16	0.142	0.046	0.007	0.094	12.30708199	59.59033539	1.396		24.438							
MDD	HIV susceptibility	hivsuscep	Weighted mode	16	5.49E-04	0.030	0.986	0.986	12.30708199	59.59033539	1.396		24.438							
MDD	HIV susceptibility	hivsuscep	Steiger-filtered IVW	16	0.032	0.021	0.123	0.273	12.30708199	59.59033539	1.396		24.438							
MDD	HIV susceptibility	hivsuscep	MR Egger SIMEX		0.017	0.051	0.741	0.897	12.30708199	59.59033539	1.396		24.438							
MDD	HIV susceptibility	hivsuscep	GSMR	16	0.029	0.019	0.126	0.249												
MDD	HIV susceptibility	hivsuscep	LHC		0.459	3.508	0.896	0.980												
MDD	Household income	income	MR Egger	115	-0.655	0.230	0.005	0.235	87.87542807	100	1.089		44.194			0.004	0.003	0.251		
MDD	Household income	income	Weighted median	115	-0.299	0.051	5.95E-09	1.06E-07	87.87542807	100	1.089		44.194							
MDD	Household income	income	Inverse variance weighte	115	-0.396	0.052	4.35E-14	9.68E-13	87.87542807	100	1.089	0.0101	44.194	0.563	unweighted				362.887	9.92E-28
MDD	Household income	income	Simple mode	115	-0.246	0.166	0.142	0.600	87.87542807	100	1.089		44.194							
MDD	Household income	income	Weighted mode	115	-0.230	0.129	0.078	0.576	87.87542807	100	1.089		44.194							
MDD	Household income	income	Steiger-filtered IVW	115	-0.396	0.052	4.35E-14	9.68E-13	87.87542807	100	1.089		44.194							
MDD	Household income	income	GSMR	109	-0.338	0.031	3.11E-27	5.54E-26												
MDD	Household income	income	PRESSO	115	-0.386	0.047	5.84E-13	8.90E-12												
MDD	Household income	income	LHC		0.288	0.321	0.370	0.586												
MDD	Hypothyroidism	hypothyroidism	MR Egger	120	0.004	0.015	0.798	0.980	99.38341749	100	1.062		84.326			9.06E-04	0.001	0.488		
MDD	Hypothyroidism	hypothyroidism	Weighted median	120	0.017	0.009	0.061	0.210	99.38341749	100	1.062		84.326							
MDD	Hypothyroidism	hypothyroidism	Inverse variance weighte	120	0.013	0.007	0.062	0.163	99.38341749	100	1.062	0.0204	84.326	0.949	weighted				222.254	3.01E-08
MDD	Hypothyroidism	hypothyroidism	Simple mode	120	0.006	0.020	0.778	0.975	99.38341749	100	1.062		84.326							
MDD	Hypothyroidism	hypothyroidism	Weighted mode	120	0.010	0.011	0.339	0.933	99.38341749	100	1.062		84.326							
MDD	Hypothyroidism	hypothyroidism	Steiger-filtered IVW	120	0.013	0.007	0.062	0.163	99.38341749	100	1.062		84.326							
MDD	Hypothyroidism	hypothyroidism	GSMR	116	0.009	0.005	0.079	0.175												
MDD	Hypothyroidism	hypothyroidism	PRESSO	120	0.012	0.007	0.082	0.151												
MDD	Hypothyroidism	hypothyroidism	LHC		0.013	0.029	0.646	0.815												
MDD	IL6 levels	il6	MR Egger	9	0.118	0.166	0.501	0.927	92.11142706	100	1.082		65.743			-0.005	0.007	0.559		
MDD	IL6 levels	il6	Weighted median	9	0.036	0.043	0.402	0.794	92.11142706	100	1.082		65.743							
MDD	IL6 levels	il6	Inverse variance weighte	9	0.032	0.087	0.710	0.915	92.11142706	100	1.082	0.0117	65.743	0.962	weighted				42.293	1.19E-06
MDD	IL6 levels	il6	Simple mode	9	0.037	0.086	0.680	0.975	92.11142706	100	1.082		65.743							
MDD	IL6 levels	il6	Weighted mode	9	0.041	0.043	0.360	0.933	92.11142706	100	1.082		65.743							
MDD	IL6 levels	il6	Steiger-filtered IVW	9	0.032	0.087	0.710	0.915	92.11142706	100	1.082		65.743							
MDD	IL6 levels	il6	GSMR	9	0.029	0.039	0.458	0.632												
MDD	IL6 levels	il6	LHC		-0.018	0.072	0.799	0.933												
MDD	Inflammatory bowel disease	ibd	MR Egger	106	-0.016	0.013	0.218	0.864	100	100	1.023		70.169			0.002	0.002	0.211		
MDD	Inflammatory bowel disease	ibd	Weighted median	106	0.002	0.007	0.781	0.947	100	100	1.023		70.169							
MDD	Inflammatory bowel disease	ibd	Inverse variance weighte	106	-0.001	0.005	0.845	0.917	100	100	1.023	0.1397	70.169	0.890	weighted				151.998	0.002
MDD	Inflammatory bowel disease	ibd	Simple mode	106	0.006	0.014	0.651	0.975	100	100	1.023		70.169							
MDD	Inflammatory bowel disease	ibd	Weighted mode	106	0.002	0.010	0.832	0.967	100	100	1.023		70.169							
MDD	Inflammatory bowel disease	ibd	Steiger-filtered IVW	106	-0.001	0.005	0.845	0.917	100	100	1.023		70.169							
MDD	Inflammatory bowel disease	ibd	MR Egger SIMEX		-0.017	0.014	0.222	0.839	100	100	1.023		70.169							
MDD	Inflammatory bowel disease	ibd	GSMR	104	0.002	0.004	0.684	0.834												
MDD	Inflammatory bowel disease	ibd	PRESSO	106	0.003	0.004	0.519	0.629												
MDD	Inflammatory bowel disease	ibd	LHC		0.023	0.018	0.202	0.358												
MDD	Insomnia	insomnia	MR Egger	13	-0.163	0.222	0.479	0.907	21.39512927	89.48926604	1.257		41.191			0.015	0.008	0.111		
MDD	Insomnia	insomnia	Weighted median	13	0.009	0.062	0.879	0.955	21.39512927	89.48926604	1.257		41.191							
MDD	Insomnia	insomnia	Inverse variance weighte	13	0.201	0.079	0.011	0.044	21.39512927	89.48926604	1.257	0.0014	41.191	0.780	unweighted				59.494	2.79E-08
MDD	Insomnia	insomnia	Simple mode	13	0.000	0.151	1.000	1.000	21.39512927	89.48926604	1.257		41.191							
MDD	Insomnia	insomnia	Weighted mode	13	-0.004	0.070	0.957	0.968	21.39512927	89.48926604	1.257		41.191							
MDD	Insomnia	insomnia	Steiger-filtered IVW	13	0.201	0.079	0.011	0.044	21.39512927	89.48926604	1.257		41.191							
MDD	Insomnia	insomnia	MR Egger SIMEX		-0.100	0.146	0.506	0.842	21.39512927	89.48926604	1.257		41.191							
MDD	Insomnia	insomnia	GSMR	12	0.182	0.039	2.91E-06	1.44E-05												
MDD	Insomnia	insomnia	PRESSO	13	0.171	0.072	0.042	0.088												
MDD	Insomnia	insomnia	LHC		-0.174	0.349	0.618	0.803												
MDD	Irritable bowel disease	irbd	MR Egger	14	-0.047	0.053	0.389	0.864	15.56093076	74.14091653	1.326		22.780			0.005	0.005	0.349		
MDD	Irritable bowel disease	irbd	Weighted median	14	0.009	0.026	0.719	0.947	15.56093076	74.14091653	1.326		22.780							
MDD	Irritable bowel disease	irbd	Inverse variance weighte	14	2.52E-04	0.020	0.990	0.999	15.56093076	74.14091653	1.326	9.21E-04	22.780	0.763	unweighted				17.382	0.182
MDD	Irritable bowel disease	irbd	Simple mode	14	0.004	0.044	0.932	0.988	15.56093076	74.14091653	1.326		22.780							
MDD	Irritable bowel disease	irbd	Weighted mode	14	0.011	0.046	0.821	0.967	15.56093076	74.14091653	1.326		22.780							
MDD	Irritable bowel disease	irbd	Steiger-filtered IVW	14	2.52E-04	0.020	0.990	0.999	15.56093076	74.14091653	1.326		22.780							
MDD	Irritable bowel disease	irbd	MR Egger SIMEX		0.010	0.055	0.861	0.909	15.56093076	74.14091653	1.326		22.780							
MDD	Irritable bowel disease	irbd	GSMR	14	0.002	0.018	0.909	0.951												
MDD	Irritable bowel disease	irbd	LHC		0.371	4.796	0.938	0.980												
MDD	Living with partner	partner	MR Egger	38	-0.106	0.231	0.649	0.980	27.34593028	96.22243239	1.217		23.946			0.002	0.007	0.804		
MDD	Living with partner	partner	Weighted median	38	-0.086	0.048	0.074	0.240	27.34593028	96.22243239	1.217		23.946							
MDD	Living with partner	partner	Inverse variance weighte	38	-0.051	0.061	0.406	0.634	27.34593028	96.22243239	1.217	0.0020	23.946	0.557	unweighted				148.166	3.06E-15
MDD	Living with partner	partner	Simple mode	38	-0.060	0.103	0.564	0.975	27.34593028	96.22243239	1.217		23.946							
MDD	Living with partner	partner	Weighted mode	38	-0.066	0.101	0.520	0.943	27.34593028	96.22243239	1.217		23.946							
MDD	Living with partner	partner	Steiger-filtered IVW	38	-0.051	0.061	0.406	0.634	27.34593028	96.22243239	1.217		23.946							
MDD	Living with partner	partner	GSMR	36	-0.060	0.033	0.066	0.151												
MDD	Living with partner	partner	PRESSO	38	-0.067	0.043	0.123	0.215												
MDD	Living with partner	partner	LHC		-0.347	3.448	0.920	0.980												
MDD	Loneliness	loneliness	MR Egger	11	-0.375	3.024	0.904	0.980	15.11487077	72.43470206	1.333		36.400			0.015	0.017	0.411		
MDD	Loneliness	loneliness	Weighted median	11	1.897	0.532	3.66E-04	0.003	15.11487077	72.43470206	1.333		36.400							
MDD	Loneliness	loneliness	Inverse variance weighte	11	2.172	0.627	5.28E-04	0.003	15.11487077	72.43470206	1.333	8.85E-04	36.400	0.479	unweighted				53.971	4.91E-08
MDD	Loneliness	loneliness	Simple mode	11	0.730	1.021	0.491	0.971	15.11487077	72.43470206	1.333		36.400							

MDD	Loneliness	loneliness	Weighted mode	11	1.307	0.847	0.154	0.704	15.11487077	72.43470206	1.333		36.400							
MDD	Loneliness	loneliness	Steiger-filtered IVW	11	2.172	0.627	5.28E-04	0.003	15.11487077	72.43470206	1.333		36.400							
MDD	Loneliness	loneliness	GSMR	10	1.538	0.308	6.11E-07	3.34E-06												
MDD	Loneliness	loneliness	PRESSO	11	1.855	0.593	0.012	0.035												
MDD	Loneliness	loneliness	LHC		0.630	0.100	2.62E-10	2.54E-09												
MDD	Long illness or disability	disability	MR Egger	17	-0.008	0.213	0.972	0.980	29.2173969	97.3161676	1.208		45.530			0.008	0.008	0.298		
MDD	Long illness or disability	disability	Weighted median	17	0.130	0.050	0.009	0.042	29.2173969	97.3161676	1.208		45.530							
MDD	Long illness or disability	disability	Inverse variance weighte	17	0.213	0.060	4.11E-04	0.003	29.2173969	97.3161676	1.208	0.0020	45.530	0.736	unweighted				60.814	3.81E-07
MDD	Long illness or disability	disability	Simple mode	17	0.202	0.086	0.031	0.250	29.2173969	97.3161676	1.208		45.530							
MDD	Long illness or disability	disability	Weighted mode	17	0.143	0.059	0.027	0.299	29.2173969	97.3161676	1.208		45.530							
MDD	Long illness or disability	disability	Steiger-filtered IVW	17	0.213	0.060	4.11E-04	0.003	29.2173969	97.3161676	1.208		45.530							
MDD	Long illness or disability	disability	MR Egger SIMEX		0.267	0.287	0.367	0.842	29.2173969	97.3161676	1.208		45.530							
MDD	Long illness or disability	disability	GSMR	16	0.164	0.033	6.39E-07	3.34E-06												
MDD	Long illness or disability	disability	PRESSO	17	0.150	0.042	0.003	0.011												
MDD	Long illness or disability	disability	LHC		0.609	0.163	1.95E-04	0.001												
MDD	Low density lipoprotein	ldl	MR Egger	364	-0.049	0.025	0.054	0.605	99.99999669	100	1.037		186.766			5.57E-04	######	0.378		
MDD	Low density lipoprotein	ldl	Weighted median	364	-0.044	0.020	0.030	0.121	99.99999669	100	1.037		186.766							
MDD	Low density lipoprotein	ldl	Inverse variance weighte	364	-0.032	0.016	0.049	0.150	99.99999669	100	1.037	0.0556	186.766	0.987	unweighted				678.906	1.90E-21
MDD	Low density lipoprotein	ldl	Simple mode	364	-0.015	0.040	0.710	0.975	99.99999669	100	1.037		186.766							
MDD	Low density lipoprotein	ldl	Weighted mode	364	-0.032	0.022	0.159	0.704	99.99999669	100	1.037		186.766							
MDD	Low density lipoprotein	ldl	Steiger-filtered IVW	364	-0.032	0.016	0.049	0.150	99.99999669	100	1.037		186.766							
MDD	Low density lipoprotein	ldl	GSMR	351	-0.011	0.012	0.343	0.536												
MDD	Low density lipoprotein	ldl	PRESSO	364	-0.020	0.015	0.188	0.302												
MDD	Low density lipoprotein	ldl	LHC		-0.046	0.024	0.057	0.139												
MDD	Malignant neoplasm of breast	cancerbreast	MR Egger	11	-0.006	0.045	0.898	0.980	96.46476269	100	1.073		72.722			4.89E-04	0.007	0.948		
MDD	Malignant neoplasm of breast	cancerbreast	Weighted median	11	0.000	0.013	0.982	0.995	96.46476269	100	1.073		72.722							
MDD	Malignant neoplasm of breast	cancerbreast	Inverse variance weighte	11	-0.003	0.013	0.821	0.917	96.46476269	100	1.073	0.0146	72.722	0.876	weighted				23.409	0.009
MDD	Malignant neoplasm of breast	cancerbreast	Simple mode	11	0.000	0.020	0.983	0.995	96.46476269	100	1.073		72.722							
MDD	Malignant neoplasm of breast	cancerbreast	Weighted mode	11	-0.006	0.014	0.657	0.943	96.46476269	100	1.073		72.722							
MDD	Malignant neoplasm of breast	cancerbreast	Steiger-filtered IVW	11	-0.003	0.013	0.821	0.917	96.46476269	100	1.073		72.722							
MDD	Malignant neoplasm of breast	cancerbreast	MR Egger SIMEX		-0.007	0.049	0.896	0.909	96.46476269	100	1.073		72.722							
MDD	Malignant neoplasm of breast	cancerbreast	GSMR	11	-0.003	0.009	0.751	0.865												
MDD	Malignant neoplasm of breast	cancerbreast	LHC		0.003	0.089	0.977	0.980												
MDD	Malignant neoplasms of skin	cancerskin	MR Egger	35	0.001	0.020	0.946	0.980	99.99995451	100	1.04		75.915			-0.003	0.003	0.402		
MDD	Malignant neoplasms of skin	cancerskin	Weighted median	35	-0.005	0.009	0.558	0.847	99.99995451	100	1.04		75.915							
MDD	Malignant neoplasms of skin	cancerskin	Inverse variance weighte	35	-0.014	0.008	0.060	0.163	99.99995451	100	1.04	0.0485	75.915	0.913	unweighted				51.809	0.026
MDD	Malignant neoplasms of skin	cancerskin	Simple mode	35	-0.003	0.020	0.893	0.987	99.99995451	100	1.04		75.915							
MDD	Malignant neoplasms of skin	cancerskin	Weighted mode	35	-0.001	0.017	0.935	0.968	99.99995451	100	1.04		75.915							
MDD	Malignant neoplasms of skin	cancerskin	Steiger-filtered IVW	35	-0.014	0.008	0.060	0.163	99.99995451	100	1.04		75.915							
MDD	Malignant neoplasms of skin	cancerskin	GSMR	35	-0.013	0.006	0.031	0.081												
MDD	Malignant neoplasms of skin	cancerskin	LHC		0.056	0.034	0.096	0.208												
MDD	Management demands	manager	MR Egger	14	-1.768	1.101	0.134	0.864	27.83135641	96.53980881	1.215		30.311			0.033	0.021	0.143		
MDD	Management demands	manager	Weighted median	14	-0.025	0.122	0.840	0.950	27.83135641	96.53980881	1.215		30.311							
MDD	Management demands	manager	Inverse variance weighte	14	-0.067	0.194	0.731	0.917	27.83135641	96.53980881	1.215	0.0019	30.311	0.660	weighted				96.057	9.59E-15
MDD	Management demands	manager	Simple mode	14	0.036	0.182	0.845	0.981	27.83135641	96.53980881	1.215		30.311							
MDD	Management demands	manager	Weighted mode	14	0.036	0.166	0.831	0.967	27.83135641	96.53980881	1.215		30.311							
MDD	Management demands	manager	Steiger-filtered IVW	14	-0.067	0.194	0.731	0.917	27.83135641	96.53980881	1.215		30.311							
MDD	Management demands	manager	MR Egger SIMEX		-3.203	1.678	0.081	0.431	27.83135641	96.53980881	1.215		30.311							
MDD	Management demands	manager	GSMR	12	-0.042	0.081	0.604	0.747												
MDD	Management demands	manager	PRESSO	14	0.014	0.114	0.908	0.923												
MDD	Management demands	manager	LHC		-0.132	1.120	0.906	0.980												
MDD	Maternal smoking	smokingmaternal	MR Egger	15	-0.376	0.319	0.260	0.864	23.83667713	93.010219	1.238		35.411			0.018	0.012	0.174		
MDD	Maternal smoking	smokingmaternal	Weighted median	15	0.043	0.053	0.419	0.794	23.83667713	93.010219	1.238		35.411							
MDD	Maternal smoking	smokingmaternal	Inverse variance weighte	15	0.074	0.065	0.254	0.462	23.83667713	93.010219	1.238	0.0016	35.411	0.365	unweighted				47.352	1.68E-05
MDD	Maternal smoking	smokingmaternal	Simple mode	15	0.032	0.094	0.736	0.975	23.83667713	93.010219	1.238		35.411							
MDD	Maternal smoking	smokingmaternal	Weighted mode	15	0.035	0.089	0.699	0.943	23.83667713	93.010219	1.238		35.411							
MDD	Maternal smoking	smokingmaternal	Steiger-filtered IVW	15	0.074	0.065	0.254	0.462	23.83667713	93.010219	1.238		35.411							
MDD	Maternal smoking	smokingmaternal	GSMR	14	0.031	0.038	0.416	0.597												
MDD	Maternal smoking	smokingmaternal	PRESSO	15	0.036	0.056	0.526	0.629												
MDD	Maternal smoking	smokingmaternal	LHC		0.211	0.423	0.619	0.803												
MDD	Medication and treatment use	treatmentuse	MR Egger	52	0.035	0.119	0.767	0.980	65.88908715	99.99974712	1.119		42.652			0.004	0.005	0.449		
MDD	Medication and treatment use	treatmentuse	Weighted median	52	0.080	0.030	0.006	0.032	65.88908715	99.99974712	1.119		42.652							
MDD	Medication and treatment use	treatmentuse	Inverse variance weighte	52	0.123	0.031	5.97E-05	4.09E-04	65.88908715	99.99974712	1.119	0.0058	42.652	0.688	unweighted				186.467	2.54E-17
MDD	Medication and treatment use	treatmentuse	Simple mode	52	0.078	0.082	0.348	0.935	65.88908715	99.99974712	1.119		42.652							
MDD	Medication and treatment use	treatmentuse	Weighted mode	52	0.069	0.053	0.197	0.725	65.88908715	99.99974712	1.119		42.652							
MDD	Medication and treatment use	treatmentuse	Steiger-filtered IVW	52	0.123	0.031	5.97E-05	4.09E-04	65.88908715	99.99974712	1.119		42.652							
MDD	Medication and treatment use	treatmentuse	MR Egger SIMEX		-0.021	0.129	0.869	0.909	65.88908715	99.99974712	1.119		42.652							
MDD	Medication and treatment use	treatmentuse	GSMR	48	0.088	0.017	4.43E-07	2.63E-06												
MDD	Medication and treatment use	treatmentuse	PRESSO	52	0.097	0.026	4.74E-04	0.002												
MDD	Medication and treatment use	treatmentuse	LHC		-0.292	0.092	0.002	0.008												
MDD	Metabolic syndrome	metabolicsyn	MR Egger	75	0.024	0.028	0.393	0.864	98.97059761	100	1.064		72.888			4.32E-04	0.002	0.815		
MDD	Metabolic syndrome	metabolicsyn	Weighted median	75	0.024	0.014	0.097	0.280	98.97059761	100	1.064		72.888							
MDD	Metabolic syndrome	metabolicsyn	Inverse variance weighte	75	0.030	0.012	0.017	0.061	98.97059761	100	1.064	0.0188	72.888	0.932	unweighted				148.386	6.60E-07
MDD	Metabolic syndrome	metabolicsyn	Simple mode	75	0.028	0.030	0.354	0.935	98.97059761	100	1.064		72.888							
MDD	Metabolic syndrome	metabolicsyn	Weighted mode	75	0.017	0.020	0.382	0.933	98.97059761	100	1.064		72.888							
MDD	Metabolic syndrome	metabolicsyn	Steiger-filtered IVW	75	0.030	0.012	0.017	0.061	98.97059761	100	1.064		72.888							
MDD	Metabolic syndrome	metabolicsyn	GSMR	73	0.024	0.009	0.007	0.022												
MDD	Metabolic syndrome	metabolicsyn	PRESSO	75	0.024	0.012	0.050	0.099												
MDD	Metabolic syndrome	metabolicsyn	LHC		0.130	0.067	0.053	0.139												
MDD	Multiple sclerosis	ms	MR Egger	64	-0.010	0.004	0.009	0.235	100	100	1.027		66.229			0.003	######	0.007		
MDD	Multiple sclerosis	ms	Weighted median	64	-0.009	0.004	0.009	0.042	100	100	1.027		66.229							
MDD	Multiple sclerosis	ms	Inverse variance weighte	64	-0.004	0.003	0.208	0.385	100	100	1.027	0.1014	66.229	0.951	unweighted				110.846	1.88E-04
MDD	Multiple sclerosis	ms	Simple mode	64	0.000	0.010	0.976	0.995	100	100	1.027		66.229							
MDD	Multiple sclerosis	ms	Weighted mode	64	-0.009	0.003	0.003	0.101	100	100	1.027		66.229							
MDD	Multiple sclerosis	ms	Steiger-filtered IVW	64	-0.004	0.003	0.208	0.385	100	100	1.027		66.229							
MDD	Multiple sclerosis	ms	GSMR	62	-0.004	0.003	0.090	0.191												
MDD	Multiple sclerosis	ms	PRESSO	64	-0.004	0.003	0.136	0.231												
MDD	Multisite chronic pain	chronicpain	MR Egger	30	0.028	0.488	0.955	0.980	37.31011052	99.45264363	1.177		35.531			0.012	0.008	0.157		
MDD	Multisite chronic pain	chronicpain	Weighted median	30	0.686	0.095	3.95E-13	8.78E-12	37.31011052	99.45264363	1.177		35.531							
MDD	Multisite chronic pain	chronicpain	Inverse variance weighte	30	0.719	0.108	2.91E-11	4.32E-10	37.31011052	99.45264363	1.177	0.0027	35.531	0.411	unweighted				115.506	2.81E-12
MDD	Multisite chronic pain	chronicpain	Simple mode	30	0.654	0.227	0.007	0.094	37.31011052	99.45264363	1.177		35.531							
MDD	Multisite chronic pain	chronicpain	Weighted mode	30	0.631	0.211	0.006	0.127	37.31011052	99.45264363	1.177		35.531							
MDD	Multisite chronic pain	chronicpain	Steiger-filtered IVW	30	0.719	0.108	2.91E-11	4.32E-10	37.31011052	99.45264363	1.177		35.531							
MDD	Multisite chronic pain	chronicpain	GSMR	30	0.614	0.060	8.29E-25	1.23E-23												
MDD	Multisite chronic pain	chronicpain	PRESSO	30	0.714	0.088	1.83E-08	1.59E-07												
MDD	Multisite chronic pain	chronicpain	LHC		0.355	1.711	0.836	0.937												
MDD	Number of children	numberchildren	MR Egger	14	0.671	0.723	0.371	0.864	33.20016683	98.74395726	1.191		35.286			-0.014	0.017	0.430		
MDD	Number of children	numberchildren	Weighted median	14	0.066	0.085	0.441	0.798	33.20016683	98.74395726	1.191		35.286							
MDD	Number of children	numberchildren	Inverse variance weighte	14	0.090	0.126	0.474	0.709	33.20016683	98.74395726	1.191	0.0024	35.286	0.552	weighted				68.593	1.46E-09
MDD	Number of children	numberchildren	Simple mode	14	0.065	0.130	0.625	0.975	33.20016683	98.74395726	1.191		35.286							
MDD	Number of children	numberchildren	Weighted mode	14	0.016	0.109	0.884	0.967	33.20016683	98.74395726	1.191		35.286							
MDD	Number of children	numberchildren	Steiger-filtered IVW	14	0.090	0.126	0.474	0.709	33.20016683	98.74395726	1.191		35.286							
MDD	Number of children	numberchildren	GSMR	12	0.068	0.061	0.262	0.441												
MDD	Number of children	numberchildren	PRESSO	14	0.034	0.069	0.635	0.725												
MDD	Number of children	numberchildren	LHC		0.305	0.041	1.85E-13	2.01E-12												
MDD	Oxygen uptake	oxyuptake	MR Egger	13	-0.113	0.266	0.679	0.980	70.80962825	99.9999616	1.112		35.181			0.006	0.009	0.477		
MDD	Oxygen uptake	oxyuptake	Weighted median	13	0.100	0.064	0.116	0.322	70.80962825	99.9999616	1.112		35.181							
MDD	Oxygen uptake	oxyuptake	Inverse variance weighte	13	0.076	0.070	0.281	0.490	70.80962825	99.9999616	1.112	0.0065	35.181	0.642	unweighted				32.380	0.001
MDD	Oxygen uptake	oxyuptake	Simple mode	13	0.099	0.117	0.418	0.935	70.80962825	99.9999616	1.112		35.181							
MDD	Oxygen uptake	oxyuptake	Weighted mode	13	0.088	0.113	0.452	0.943	70.80962825	99.9999616	1.112		35.181							
MDD	Oxygen uptake	oxyuptake	Steiger-filtered IVW	13	0.076	0.070	0.281	0.500	70.80962825	99.9999616	1.112		35.181							
MDD	Oxygen uptake	oxyuptake	MR Egger SIMEX		-0.067	0.194	0.737	0.897	70.80962825	99.9999616	1.112		35.181							
MDD	Oxygen uptake	oxyuptake	GSMR	13	0.060	0.044	0.170	0.303												
MDD	Oxygen uptake	oxyuptake	PRESSO	13	0.038	0.057	0.521	0.629												
MDD	Oxygen uptake	oxyuptake	LHC		0.031	1.244	0.980	0.980												
MDD	Parental longevity	parentlongevity	MR Egger	15	-0.035	0.098	0.722	0.980	79.33989408	99.99999936	1.101		24.121			-0.002	0.006	0.728		
MDD	Parental longevity	parentlongevity	Weighted median	15	-0.031	0.043	0.477	0.798	79.33989408	99.99999936	1.101		24.121							
MDD	Parental longevity	parentlongevity	Inverse variance weighte	15	-0.067	0.039	0.083	0.205	79.33989408	99.99999936	1.101	0.0079	24.121	0.768	unweighted				21.765	0.084
MDD	Parental longevity	parentlongevity	Simple mode	15	0.007	0.076	0.926	0.988	79.33989408	99.99999936	1.101		24.121							
MDD	Parental longevity	parentlongevity	Weighted mode	15	0.013	0.069	0.854	0.967	79.33989408	99.99999936	1.101		24.121							
MDD	Parental longevity	parentlongevity	Steiger-filtered IVW	15	-0.067	0.039	0.083	0.205	79.33989408	99.99999936	1.101		24.121							
MDD	Parental longevity	parentlongevity	MR Egger SIMEX		-0.103	0.133	0.454	0.842	79.33989408	99.99999936	1.101		24.121							
MDD	Parental longevity	parentlongevity	GSMR	15	-0.055	0.032	0.084	0.183												
MDD	Parental longevity	parentlongevity	LHC		0.368	0.064	8.88E-09	7.73E-08												
MDD	Parental mortality	parentlife	MR Egger	10	0.215	0.312	0.510	0.927	29.39228444	97.40180442	1.207		80.391			-0.006	0.008	0.441		
MDD	Parental mortality	parentlife	Weighted median	10	0.018	0.092	0.844	0.950	29.39228444	97.40180442	1.207		80.391							
MDD	Parental mortality	parentlife	Inverse variance weighte	10	-0.004	0.152	0.976	0.999	29.39228444	97.40180442	1.207	0.0021	80.391	0.955	unweighted				43.881	1.48E-06
MDD	Parental mortality	parentlife	Simple mode	10	-0.067	0.164	0.692	0.975	29.39228444	97.40180442	1.207		80.391							
MDD	Parental mortality	parentlife	Weighted mode	10	0.079	0.088	0.397	0.933	29.39228444	97.40180442	1.207		80.391							
MDD	Parental mortality	parentlife	Steiger-filtered IVW	10	-0.004	0.152	0.976	0.999	29.39228444	97.40180442	1.207		80.391							
MDD	Parental mortality	parentlife	GSMR	9	0.071	0.071	0.319	0.516												
MDD	Parental mortality	parentlife	PRESSO	10	0.038	0.087	0.674	0.734												
MDD	Parental mortality	parentlife	LHC		-0.293	0.233	0.209	0.363												
MDD	Parkinson disease	parkinson	MR Egger	14	-0.032	0.023	0.201	0.864	96.97488682	100	1.072		46.284			0.002	0.004	0.636		
MDD	Parkinson disease	parkinson	Weighted median	14	-0.019	0.011	0.086	0.256	96.97488682	100	1.072		46.284							
MDD	Parkinson disease	parkinson	Inverse variance weighte	14	-0.021	0.009	0.024	0.078	96.97488682	100	1.072	0.0151	46.284	0.858	unweighted				19.905	0.098
MDD	Parkinson disease	parkinson	Simple mode	14	-0.017	0.020	0.398	0.935	96.97488682	100	1.072		46.284							
MDD	Parkinson disease	parkinson	Weighted mode	14	-0.017	0.015	0.282	0.902	96.97488682	100	1.072		46.284							
MDD	Parkinson disease	parkinson	Steiger-filtered IVW	14	-0.021	0.009	0.024	0.078	96.97488682	100	1.072		46.284							
MDD	Parkinson disease	parkinson	MR Egger SIMEX		0.004	0.019	0.837	0.909	96.97488682	100	1.072		46.284							
MDD	Parkinson disease	parkinson	GSMR	14	-0.020	0.008	0.010	0.029												
MDD	Parkinson disease	parkinson	LHC		0.043	0.096	0.656	0.815												
MDD	Pelvic organ prolapse	pelvicorganprol	MR Egger	24	-0.051	0.046	0.277	0.864	31.30094428	98.18608579	1.199		53.301			0.005	0.004	0.219		
MDD	Pelvic organ prolapse	pelvicorganprol	Weighted median	24	0.003	0.019	0.869	0.954	31.30094428	98.18608579	1.199		53.301							
MDD	Pelvic organ prolapse	pelvicorganprol	Inverse variance weighte	24	0.004	0.014	0.775	0.917	31.30094428	98.18608579	1.199	0.0022	53.301	0.816	unweighted				29.262	0.172
MDD	Pelvic organ prolapse	pelvicorganprol	Simple mode	24	0.013	0.030	0.677	0.975	31.30094428	98.18608579	1.199		53.301							
MDD	Pelvic organ prolapse	pelvicorganprol	Weighted mode	24	0.006	0.026	0.817	0.967	31.30094428	98.18608579	1.199		53.301							
MDD	Pelvic organ prolapse	pelvicorganprol	Steiger-filtered IVW	24	0.004	0.014	0.775	0.917	31.30094428	98.18608579	1.199		53.301							
MDD	Pelvic organ prolapse	pelvicorganprol	MR Egger SIMEX		0.058	0.047	0.231	0.839	31.30094428	98.18608579	1.199		53.301							

MDD	Pelvic organ prolapse	pelvicorganprol	GSMR	24	0.004	0.013	0.771	0.865												
MDD	Pelvic organ prolapse	pelvicorganprol	LHC		0.036	0.177	0.840	0.937												
MDD	Peripheral artery disease	peripheralartdis	MR Egger	27	-0.050	0.053	0.354	0.864	29.0904397	97.25238051	1.209		34.871			0.005	0.006	0.366		
MDD	Peripheral artery disease	peripheralartdis	Weighted median	27	-0.012	0.016	0.462	0.798	29.0904397	97.25238051	1.209		34.871							
MDD	Peripheral artery disease	peripheralartdis	Inverse variance weighte	27	-0.004	0.019	0.812	0.917	29.0904397	97.25238051	1.209	0.0020	34.871	0.722	unweighted				89.518	6.65E-09
MDD	Peripheral artery disease	peripheralartdis	Simple mode	27	-0.027	0.030	0.367	0.935	29.0904397	97.25238051	1.209		34.871							
MDD	Peripheral artery disease	peripheralartdis	Weighted mode	27	-0.023	0.027	0.399	0.933	29.0904397	97.25238051	1.209		34.871							
MDD	Peripheral artery disease	peripheralartdis	Steiger-filtered IVW	27	-0.004	0.019	0.812	0.917	29.0904397	97.25238051	1.209		34.871							
MDD	Peripheral artery disease	peripheralartdis	MR Egger SIMEX		0.128	0.043	0.006	0.083	29.0904397	97.25238051	1.209		34.871							
MDD	Peripheral artery disease	peripheralartdis	GSMR	26	-0.015	0.011	0.156	0.298												
MDD	Peripheral artery disease	peripheralartdis	PRESSO	27	-0.011	0.014	0.421	0.578												
MDD	Peripheral artery disease	peripheralartdis	LHC		0.038	0.041	0.356	0.584												
MDD	Physical activity	physactivity	MR Egger	12	-0.096	0.370	0.800	0.980	47.71814861	99.9476854	1.151		28.233			-0.002	0.011	0.874		
MDD	Physical activity	physactivity	Weighted median	12	-0.144	0.081	0.076	0.240	47.71814861	99.9476854	1.151		28.233							
MDD	Physical activity	physactivity	Inverse variance weighte	12	-0.155	0.083	0.061	0.163	47.71814861	99.9476854	1.151	0.0037	28.233	0.439	unweighted				27.321	0.004
MDD	Physical activity	physactivity	Simple mode	12	-0.054	0.119	0.657	0.975	47.71814861	99.9476854	1.151		28.233							
MDD	Physical activity	physactivity	Weighted mode	12	-0.062	0.123	0.626	0.943	47.71814861	99.9476854	1.151		28.233							
MDD	Physical activity	physactivity	Steiger-filtered IVW	12	-0.155	0.083	0.061	0.163	47.71814861	99.9476854	1.151		28.233							
MDD	Physical activity	physactivity	GSMR	12	-0.124	0.055	0.023	0.062												
MDD	Physical activity	physactivity	PRESSO	12	-0.096	0.067	0.185	0.302												
MDD	Pregnancy terminations	pregnantermin	MR Egger	18	0.134	0.105	0.221	0.864	71.97227719	99.99997641	1.111		23.774			0.002	0.003	0.538		
MDD	Pregnancy terminations	pregnantermin	Weighted median	18	0.221	0.074	0.003	0.014	71.97227719	99.99997641	1.111		23.774							
MDD	Pregnancy terminations	pregnantermin	Inverse variance weighte	18	0.189	0.056	6.84E-04	0.004	71.97227719	99.99997641	1.111	0.0066	23.774	0.796	unweighted				21.431	0.208
MDD	Pregnancy terminations	pregnantermin	Simple mode	18	0.214	0.119	0.091	0.509	71.97227719	99.99997641	1.111		23.774							
MDD	Pregnancy terminations	pregnantermin	Weighted mode	18	0.221	0.097	0.036	0.334	71.97227719	99.99997641	1.111		23.774							
MDD	Pregnancy terminations	pregnantermin	Steiger-filtered IVW	18	0.189	0.056	6.84E-04	0.004	71.97227719	99.99997641	1.111		23.774							
MDD	Pregnancy terminations	pregnantermin	MR Egger SIMEX		0.211	0.065	0.005	0.083	71.97227719	99.99997641	1.111		23.774							
MDD	Pregnancy terminations	pregnantermin	GSMR	18	0.173	0.052	8.55E-04	0.003												
MDD	Pregnancy terminations	pregnantermin	LHC		-0.092	0.051	0.074	0.170												
MDD	Rheumatoid arthritis	rheuma	MR Egger	66	0.007	0.007	0.322	0.864	100	100	1.031		116.440			-0.001	0.001	0.669		
MDD	Rheumatoid arthritis	rheuma	Weighted median	66	0.001	0.006	0.815	0.947	100	100	1.031		116.440							
MDD	Rheumatoid arthritis	rheuma	Inverse variance weighte	66	0.005	0.005	0.319	0.516	100	100	1.031	0.0787	116.440	0.984	unweighted				108.680	5.57E-04
MDD	Rheumatoid arthritis	rheuma	Simple mode	66	-0.010	0.012	0.403	0.935	100	100	1.031		116.440							
MDD	Rheumatoid arthritis	rheuma	Weighted mode	66	0.005	0.006	0.456	0.943	100	100	1.031		116.440							
MDD	Rheumatoid arthritis	rheuma	Steiger-filtered IVW	66	0.005	0.005	0.319	0.526	100	100	1.031		116.440							
MDD	Rheumatoid arthritis	rheuma	GSMR	62	0.003	0.004	0.403	0.588												
MDD	Rheumatoid arthritis	rheuma	PRESSO	66	0.005	0.004	0.270	0.412												
MDD	Rheumatoid arthritis	rheuma	LHC		0.000	0.007	0.975	0.980												
MDD	Risky behaviours	riskbehavior	MR Egger	74	-0.016	0.248	0.948	0.980	41.74823706	99.789384	1.165		41.964			0.004	0.005	0.355		
MDD	Risky behaviours	riskbehavior	Weighted median	74	0.181	0.055	0.001	0.007	41.74823706	99.789384	1.165		41.964							
MDD	Risky behaviours	riskbehavior	Inverse variance weighte	74	0.207	0.063	9.45E-04	0.005	41.74823706	99.789384	1.165	0.0032	41.964	0.644	unweighted				317.962	2.64E-32
MDD	Risky behaviours	riskbehavior	Simple mode	74	0.500	0.179	0.007	0.094	41.74823706	99.789384	1.165		41.964							
MDD	Risky behaviours	riskbehavior	Weighted mode	74	0.212	0.158	0.182	0.704	41.74823706	99.789384	1.165		41.964							
MDD	Risky behaviours	riskbehavior	Steiger-filtered IVW	74	0.207	0.063	9.45E-04	0.005	41.74823706	99.789384	1.165		41.964							
MDD	Risky behaviours	riskbehavior	MR Egger SIMEX		-0.134	0.156	0.395	0.842	41.74823706	99.789384	1.165		41.964							
MDD	Risky behaviours	riskbehavior	GSMR	66	0.212	0.033	1.69E-10	1.50E-09												
MDD	Risky behaviours	riskbehavior	PRESSO	74	0.231	0.049	1.21E-05	7.41E-05												
MDD	Risky behaviours	riskbehavior	LHC		1.000	8.74E-04	0.00E+00	0.00E+00												
MDD	Sedentary behavior	sedentary	MR Egger	12	0.022	0.277	0.939	0.980	49.26448742	99.96435323	1.147		29.397			0.000	0.008	0.957		
MDD	Sedentary behavior	sedentary	Weighted median	12	0.064	0.073	0.382	0.772	49.26448742	99.96435323	1.147		29.397							
MDD	Sedentary behavior	sedentary	Inverse variance weighte	12	0.007	0.058	0.905	0.958	49.26448742	99.96435323	1.147	0.0039	29.397	0.360	unweighted				14.765	0.193
MDD	Sedentary behavior	sedentary	Simple mode	12	0.082	0.142	0.575	0.975	49.26448742	99.96435323	1.147		29.397							
MDD	Sedentary behavior	sedentary	Weighted mode	12	0.094	0.134	0.496	0.943	49.26448742	99.96435323	1.147		29.397							
MDD	Sedentary behavior	sedentary	Steiger-filtered IVW	12	0.007	0.058	0.905	0.958	49.26448742	99.96435323	1.147		29.397							
MDD	Sedentary behavior	sedentary	GSMR	12	0.006	0.051	0.906	0.951												
MDD	Sedentary behavior	sedentary	LHC		-0.462	0.163	0.005	0.020												
MDD	Self-reported health	health	MR Egger	15	0.809	0.955	0.412	0.874	49.89310719	99.96959034	1.146		29.315			-0.015	0.025	0.559		
MDD	Self-reported health	health	Weighted median	15	0.311	0.090	5.72E-04	0.004	49.89310719	99.96959034	1.146		29.315							
MDD	Self-reported health	health	Inverse variance weighte	15	0.243	0.138	0.079	0.201	49.89310719	99.96959034	1.146	0.0039	29.315	0.353	weighted				108.094	1.31E-16
MDD	Self-reported health	health	Simple mode	15	0.296	0.182	0.127	0.585	49.89310719	99.96959034	1.146		29.315							
MDD	Self-reported health	health	Weighted mode	15	0.232	0.146	0.133	0.704	49.89310719	99.96959034	1.146		29.315							
MDD	Self-reported health	health	Steiger-filtered IVW	15	0.243	0.138	0.079	0.201	49.89310719	99.96959034	1.146		29.315							
MDD	Self-reported health	health	GSMR	14	0.300	0.057	1.19E-07	7.56E-07												
MDD	Self-reported health	health	PRESSO	15	0.264	0.094	0.017	0.047												
MDD	Self-reported health	health	LHC		0.112	0.091	0.214	0.366												
MDD	Self-reported illnesses	illnesses	MR Egger	39	0.254	0.230	0.277	0.864	50.08046799	99.9710068	1.146		38.809			0.003	0.007	0.624		
MDD	Self-reported illnesses	illnesses	Weighted median	39	0.236	0.051	4.65E-06	5.18E-05	50.08046799	99.9710068	1.146		38.809							
MDD	Self-reported illnesses	illnesses	Inverse variance weighte	39	0.364	0.053	7.41E-12	1.32E-10	50.08046799	99.9710068	1.146	0.0040	38.809	0.586	unweighted				145.716	1.57E-14
MDD	Self-reported illnesses	illnesses	Simple mode	39	0.205	0.102	0.052	0.357	50.08046799	99.9710068	1.146		38.809							
MDD	Self-reported illnesses	illnesses	Weighted mode	39	0.200	0.093	0.037	0.334	50.08046799	99.9710068	1.146		38.809							
MDD	Self-reported illnesses	illnesses	Steiger-filtered IVW	39	0.364	0.053	7.41E-12	1.32E-10	50.08046799	99.9710068	1.146		38.809							
MDD	Self-reported illnesses	illnesses	GSMR	35	0.258	0.031	3.73E-17	4.15E-16												
MDD	Self-reported illnesses	illnesses	PRESSO	39	0.332	0.042	3.49E-09	3.55E-08												
MDD	Self-reported illnesses	illnesses	LHC		-0.132	0.471	0.779	0.932												
MDD	Severe covid 19	covid	MR Egger	27	-0.0052774	0.0150884	0.7294436	0.979695	30.86776054	98.03023625	1.201		87.240			1.28E-04	0.002	0.958		
MDD	Severe covid 19	covid	Weighted median	27	-0.0014726	0.0088998	0.8685829	0.9543689	30.86776054	98.03023625	1.201		87.240							
MDD	Severe covid 19	covid	Inverse variance weighte	27	-0.0046086	0.0081088	0.569798	0.7923754	30.86776054	98.03023625	1.201	0.00219	87.240	0.964	unweighted				44.535	0.013
MDD	Severe covid 19	covid	Simple mode	27	0.00113715	0.0182101	0.9506849	0.9948465	30.86776054	98.03023625	1.201		87.240							
MDD	Severe covid 19	covid	Weighted mode	27	-0.0013167	0.0087936	0.882125	0.966799	30.86776054	98.03023625	1.201		87.240							
MDD	Severe covid 19	covid	Steiger-filtered IVW	27	-0.0046086	0.0081088	0.569798	0.7923754	30.86776054	98.03023625	1.201		87.240							
MDD	Severe covid 19	covid	GSMR	26	-0.0010479	0.0063182	0.8682763	0.9417235												
MDD	Severe covid 19	covid	PRESSO	27	-0.0009956	0.0068426	0.8854878	0.9155043												
MDD	Severe covid 19	covid	LHC		-0.1554514	0.1134846	0.1707489	0.3301146												
MDD	Sleep duration	sleepduration	MR Egger	11	0.06233929	0.1726801	0.7264206	0.979695	63.46545436	99.99941424	1.123		45.179			0.000	0.006	0.939		
MDD	Sleep duration	sleepduration	Weighted median	11	0.01360448	0.0582579	0.8153563	0.9469578	63.46545436	99.99941424	1.123		45.179							
MDD	Sleep duration	sleepduration	Inverse variance weighte	11	0.04946108	0.0529607	0.3503453	0.5567987	63.46545436	99.99941424	1.123	0.0054519	45.179	0.804	unweighted				15.438	0.117
MDD	Sleep duration	sleepduration	Simple mode	11	0.01768585	0.0964428	0.8581634	0.9809143	63.46545436	99.99941424	1.123		45.179							
MDD	Sleep duration	sleepduration	Weighted mode	11	0.00898624	0.0757867	0.9079619	0.966799	63.46545436	99.99941424	1.123		45.179							
MDD	Sleep duration	sleepduration	Steiger-filtered IVW	11	0.04946108	0.0529607	0.3503453	0.5605509	63.46545436	99.99941424	1.123		45.179							
MDD	Sleep duration	sleepduration	MR Egger SIMEX		0.06721225	0.1134522	0.5681477	0.8417003	63.46545436	99.99941424	1.123		45.179							
MDD	Sleep duration	sleepduration	GSMR	11	0.04962923	0.0433405	0.2521678	0.4400575												
MDD	Sleep duration	sleepduration	LHC		-0.2406124	0.0421437	1.13E-08	8.97E-08												
MDD	Smoking cessation	smokingces	MR Egger	19	-0.4893899	0.3701906	0.2036835	0.86419	38.05813716	99.53197929	1.175		57.436			0.014	0.007	0.048		
MDD	Smoking cessation	smokingces	Weighted median	19	-0.0023737	0.103888	0.981771	0.9947166	38.05813716	99.53197929	1.175		57.436							
MDD	Smoking cessation	smokingces	Inverse variance weighte	19	0.24431916	0.1498881	0.1030992	0.2414693	38.05813716	99.53197929	1.175	0.0028161	57.436	0.865	unweighted				104.989	2.68E-14
MDD	Smoking cessation	smokingces	Simple mode	19	0.09487802	0.1961389	0.6344107	0.9748822	38.05813716	99.53197929	1.175		57.436							
MDD	Smoking cessation	smokingces	Weighted mode	19	0.00957081	0.0980858	0.9233474	0.966799	38.05813716	99.53197929	1.175		57.436							
MDD	Smoking cessation	smokingces	Steiger-filtered IVW	19	0.24431916	0.1498881	0.1030992	0.2414693	38.05813716	99.53197929	1.175		57.436							
MDD	Smoking cessation	smokingces	MR Egger SIMEX		-0.2373369	0.3727829	0.5328246	0.8417003	38.05813716	99.53197929	1.175		57.436							
MDD	Smoking cessation	smokingces	GSMR	17	0.28500411	0.0670845	2.15E-05	8.71E-05												
MDD	Smoking cessation	smokingces	PRESSO	19	0.24596673	0.109357	0.0411151	0.088309												
MDD	Smoking cessation	smokingces	LHC		0.53074709	0.1157449	4.53E-06	3.03E-05												
MDD	Smoking initiation	smokingever	MR Egger	193	0.37978073	0.2161731	0.0805481	0.7965316	83.31518331	99.99999995	1.096		36.893			0.001	0.003	0.659		
MDD	Smoking initiation	smokingever	Weighted median	193	0.47152754	0.0527006	3.64E-19	1.08E-17	83.31518331	99.99999995	1.096		36.893							
MDD	Smoking initiation	smokingever	Inverse variance weighte	193	0.47251046	0.0509433	1.77E-20	5.26E-19	83.31518331	99.99999995	1.096	0.0088872	36.893	0.526	unweighted				633.865	1.23E-48
MDD	Smoking initiation	smokingever	Simple mode	193	0.76704385	0.1897514	7.65E-05	0.006812	83.31518331	99.99999995	1.096		36.893							
MDD	Smoking initiation	smokingever	Weighted mode	193	0.64274556	0.1562025	5.75E-05	0.0051142	83.31518331	99.99999995	1.096		36.893							
MDD	Smoking initiation	smokingever	Steiger-filtered IVW	193	0.47251046	0.0509433	1.77E-20	5.26E-19	83.31518331	99.99999995	1.096		36.893							
MDD	Smoking initiation	smokingever	GSMR	176	0.51664853	0.0310261	2.92E-62	2.60E-60												
MDD	Smoking initiation	smokingever	PRESSO	193	0.49640749	0.0466601	5.98E-21	1.21E-19												
MDD	Smoking initiation	smokingever	LHC		0.62504741	0.0808111	1.04E-14	1.50E-13												
MDD	Staphylococcus aureus	staphylococcus	MR Egger	15	-0.012512	0.0489505	0.8022556	0.979695	16.44030056	77.25280447	1.312		26.840			0.001	0.006	0.872		
MDD	Staphylococcus aureus	staphylococcus	Weighted median	15	0.01458681	0.0203475	0.4734445	0.7975867	16.44030056	77.25280447	1.312		26.840							
MDD	Staphylococcus aureus	staphylococcus	Inverse variance weighte	15	-0.0050604	0.0179774	0.7783376	0.9174889	16.44030056	77.25280447	1.312	9.93E-04	26.840	0.758	unweighted				24.598	0.039
MDD	Staphylococcus aureus	staphylococcus	Simple mode	15	-0.057059	0.0416036	0.1918038	0.7421972	16.44030056	77.25280447	1.312		26.840							
MDD	Staphylococcus aureus	staphylococcus	Weighted mode	15	0.01809819	0.0316488	0.5764943	0.9427278	16.44030056	77.25280447	1.312		26.840							
MDD	Staphylococcus aureus	staphylococcus	Steiger-filtered IVW	15	-0.0050604	0.0179774	0.7783376	0.9174889	16.44030056	77.25280447	1.312		26.840							
MDD	Staphylococcus aureus	staphylococcus	MR Egger SIMEX		-0.1113727	0.0380775	0.0118288	0.1182884	16.44030056	77.25280447	1.312		26.840							
MDD	Staphylococcus aureus	staphylococcus	GSMR	15	-0.005507	0.0139579	0.6931777	0.8336867												
MDD	Staphylococcus aureus	staphylococcus	LHC		-0.1252897	0.1539263	0.4156683	0.6344411												
MDD	Suicidal behavior	suicbehav	MR Egger	42	0.06611912	0.0752467	0.3848131	0.86419	29.28012857	97.34717987	1.208		24.951			0.005	0.005	0.299		
MDD	Suicidal behavior	suicbehav	Weighted median	42	0.10264097	0.0249694	3.95E-05	3.51E-04	29.28012857	97.34717987	1.208		24.951							
MDD	Suicidal behavior	suicbehav	Inverse variance weighte	42	0.14134221	0.0235855	2.06E-09	2.30E-08	29.28012857	97.34717987	1.208	0.0020553	24.951	0.568	unweighted				124.139	2.70E-10
MDD	Suicidal behavior	suicbehav	Simple mode	42	0.10219142	0.0791563	0.2039329	0.7562512	29.28012857	97.34717987	1.208		24.951							
MDD	Suicidal behavior	suicbehav	Weighted mode	42	0.02535046	0.0628881	0.6889658	0.9427278	29.28012857	97.34717987	1.208		24.951							
MDD	Suicidal behavior	suicbehav	Steiger-filtered IVW	42	0.14134221	0.0235855	2.06E-09	2.30E-08	29.28012857	97.34717987	1.208		24.951							
MDD	Suicidal behavior	suicbehav	GSMR	42	0.11663436	0.0147209	2.32E-15	2.29E-14												
MDD	Suicidal behavior	suicbehav	PRESSO	42	0.12492033	0.0224019	2.01E-06	1.36E-05												
MDD	Suicidal behavior	suicbehav	LHC		0.97131408	0.0053615	0.00E+00	0.00E+00												
MDD	Suicidal thought	suicthought	MR Egger	21	0.64473477	0.5385707	0.2459824	0.86419	13.32240423	64.66326055	1.37		30.876			-0.006	0.017	0.743		
MDD	Suicidal thought	suicthought	Weighted median	21	0.40680375	0.0755871	7.37E-08	1.09E-06	13.32240423	64.66326055	1.37		30.876							
MDD	Suicidal thought	suicthought	Inverse variance weighte	21	0.46903818	0.1014916	3.81E-06	3.77E-05	13.32240423	64.66326055	1.37	0.001023	30.876	0.178	unweighted				182.337	3.38E-28
MDD	Suicidal thought	suicthought	Simple mode	21	0.74948789	0.1630178	1.74E-04	0.0077573	13.32240423	64.66326055	1.37		30.876							
MDD	Suicidal thought	suicthought	Weighted mode	21	0.45410689	0.1623004	0.0111076	0.164762	13.32240423	64.66326055	1.37		30.876							
MDD	Suicidal thought	suicthought	Steiger-filtered IVW	21	0.46903818	0.1014916	3.81E-06	3.77E-05	13.32240423	64.66326055	1.37		30.876							
MDD	Suicidal thought	suicthought	GSMR	17	0.54198121	0.0455667	1.27E-32	2.82E-31												
MDD	Suicidal thought	suicthought	PRESSO	21	0.56741374	0.0727866	1.85E-06	1.36E-05												
MDD	Suicidal thought	suicthought	LHC		0.95494287	0.0015398	0.00E+00	0.00E+00												
MDD	Suicide death	suicdeath	MR Egger	9	-0.0183965	0.0158153	0.2828586	0.86419	98.69536414	100	1.066		33.789			0.013	0.006	0.055		
MDD	Suicide death	suicdeath	Weighted median	9	-0.0087453	0.0108086	0.4184562	0.7940679	98.69536414	100	1.066		33.789							
MDD	Suicide death	suicdeath	Inverse variance weighte	9	0.01153087	0.011085	0.2982345	0.5104398	98.69536414	100	1.066	0.0179832	33.789	0.909	unweighted				15.221	0.055
MDD	Suicide death	suicdeath	Simple mode	9	-0.0058678	0.0165574	0.7322044	0.9748822	98.69536414	100	1.066		33.789							
MDD	Suicide death	suicdeath	Weighted mode	9	-0.0074457	0.0122987	0.5616842	0.9427278	98.69536414	100	1.066		33.789							
MDD	Suicide death	suicdeath	Steiger-filtered IVW	9	0.01153087	0.011085	0.2982345	0.5204484	98.69536414	100	1.066		33.789							
MDD	Suicide death	suicdeath	GSMR	9	0.00921133	0.0082185	0.2623718	0.4405867												
MDD	Suicide death	suicdeath	LHC		-0.1051528	0.2323612	0.6508798	0.8151302												
MDD	Systemic lupus erythematosus	lupus	MR Egger	39	-0.0052906	0.0061885	0.39811	0.86419	100	100	1.017		96.064			6.00E-04	0.002	0.779		
MDD	Systemic lupus erythematosus	lupus	Weighted median	39	-0.004701	0.0041181	0.2536414	0.5643521	100	100	1.017		96.064							
MDD	Systemic lupus erythematosus	lupus	Inverse variance weighte	39	-0.0037414	0.0028206	0.1846916	0.3497351	100	100	1.017	0.2590066	96.064	0.956	unweighted				45.413	0.191

MDD	Systemic lupus erythematosus	lupus	Simple mode	39	-0.0042185	0.0089891	0.6415417	0.9748822	100	100	1.017		96.064							
MDD	Systemic lupus erythematosus	lupus	Weighted mode	39	-0.0078363	0.0056249	0.1716763	0.7035805	100	100	1.017		96.064							
MDD	Systemic lupus erythematosus	lupus	Steiger-filtered IVW	39	-0.0037414	0.0028206	0.1846916	0.3497351	100	100	1.017		96.064							
MDD	Systemic lupus erythematosus	lupus	GSMR	39	-0.0036887	0.0026073	0.1571472	0.2975765												
MDD	Systemic lupus erythematosus	lupus	LHC		0.04022608	0.0054257	1.23E-13	1.52E-12												
MDD	Systolic blood pressure	sysbloodpres	MR Egger	504	0.00244643	0.00318	0.4420717	0.8781345	99.99849212	100	1.045		80.353			2.49E-07	######	1.000		
MDD	Systolic blood pressure	sysbloodpres	Weighted median	504	0.00273696	0.0015959	0.0863487	0.2561678	99.99849212	100	1.045		80.353							
MDD	Systolic blood pressure	sysbloodpres	Inverse variance weighte	504	0.00244736	0.0012755	0.0550099	0.1579318	99.99849212	100	1.045	0.038615	80.353	0.921	unweighted				1031.437	1.16E-38
MDD	Systolic blood pressure	sysbloodpres	Simple mode	504	0.00229958	0.0054197	0.67153	0.9748822	99.99849212	100	1.045		80.353							
MDD	Systolic blood pressure	sysbloodpres	Weighted mode	504	0.00290705	0.0032567	0.3724781	0.9334112	99.99849212	100	1.045		80.353							
MDD	Systolic blood pressure	sysbloodpres	Steiger-filtered IVW	504	0.00244736	0.0012755	0.0550099	0.1579318	99.99849212	100	1.045		80.353							
MDD	Systolic blood pressure	sysbloodpres	GSMR	480	0.00180888	9.22E-04	0.0498595	0.1199323												
MDD	Systolic blood pressure	sysbloodpres	PRESSO	504	0.00260275	0.0012448	0.0370355	0.0836728												
MDD	Systolic blood pressure	sysbloodpres	LHC		-0.0105913	0.0382427	0.7818186	0.9317564												
MDD	Tea consumption	tea	MR Egger	13	0.01897601	0.0681745	0.7859106	0.979695	56.60197819	99.99504842	1.134		22.862			-0.002	0.006	0.692		
MDD	Tea consumption	tea	Weighted median	13	-0.0096378	0.0251205	0.7012279	0.9455952	56.60197819	99.99504842	1.134		22.862							
MDD	Tea consumption	tea	Inverse variance weighte	13	-0.0066561	0.0249978	0.790032	0.9174889	56.60197819	99.99504842	1.134	0.0046423	22.862	0.694	unweighted				24.898	0.015
MDD	Tea consumption	tea	Simple mode	13	-0.0071549	0.03776	0.8528811	0.9809143	56.60197819	99.99504842	1.134		22.862							
MDD	Tea consumption	tea	Weighted mode	13	-0.011662	0.0361772	0.7527279	0.966799	56.60197819	99.99504842	1.134		22.862							
MDD	Tea consumption	tea	Steiger-filtered IVW	13	-0.0066561	0.0249978	0.790032	0.9174889	56.60197819	99.99504842	1.134		22.862							
MDD	Tea consumption	tea	MR Egger SIMEX		-0.027134	0.0231796	0.2664954	0.8417003	56.60197819	99.99504842	1.134		22.862							
MDD	Tea consumption	tea	GSMR	13	-0.0048296	0.0179262	0.7876109	0.8653997												
MDD	Tea consumption	tea	PRESSO	13	-0.0062909	0.0136698	0.6552134	0.7266912												
MDD	Tea consumption	tea	LHC		-0.2158479	0.0780643	0.0056923	0.0225103												
MDD	Total testosterone levels	testosterone	MR Egger	193	-0.0290782	0.0289224	0.3159839	0.86419	100	100	1.032		88.324			3.25E-04	######	0.727		
MDD	Total testosterone levels	testosterone	Weighted median	193	-0.0056476	0.0204728	0.7826582	0.9469578	100	100	1.032		88.324							
MDD	Total testosterone levels	testosterone	Inverse variance weighte	193	-0.0204271	0.0149979	0.1731977	0.3380833	100	100	1.032	0.0738692	88.324	0.959	unweighted				311.460	1.06E-07
MDD	Total testosterone levels	testosterone	Simple mode	193	-0.0103081	0.0398433	0.7961311	0.9809143	100	100	1.032		88.324							
MDD	Total testosterone levels	testosterone	Weighted mode	193	-0.0103081	0.0239216	0.667015	0.9427278	100	100	1.032		88.324							
MDD	Total testosterone levels	testosterone	Steiger-filtered IVW	193	-0.0204271	0.0149979	0.1731977	0.3380833	100	100	1.032		88.324							
MDD	Total testosterone levels	testosterone	GSMR	186	-0.0185011	0.0120107	0.123467	0.2487362												
MDD	Total testosterone levels	testosterone	PRESSO	193	-0.0259932	0.0142521	0.0697508	0.1329625												
MDD	Total testosterone levels	testosterone	LHC		0.01306321	0.0231756	0.5729838	0.7669168												
MDD	Traumatic injury	traumainjury	MR Egger	9	-0.0642687	0.3566575	0.8621034	0.979695	10.23533057	47.77225419	1.471		24.744			0.007	0.015	0.670		
MDD	Traumatic injury	traumainjury	Weighted median	9	-0.0174101	0.058758	0.7670003	0.9469578	10.23533057	47.77225419	1.471		24.744							
MDD	Traumatic injury	traumainjury	Inverse variance weighte	9	0.08882397	0.0871327	0.3080084	0.5119775	10.23533057	47.77225419	1.471	4.91E-04	24.744	0.419	unweighted				40.900	2.18E-06
MDD	Traumatic injury	traumainjury	Simple mode	9	0.02559323	0.0867369	0.7754549	0.9748822	10.23533057	47.77225419	1.471		24.744							
MDD	Traumatic injury	traumainjury	Weighted mode	9	0.02559323	0.0860525	0.773727	0.966799	10.23533057	47.77225419	1.471		24.744							
MDD	Traumatic injury	traumainjury	Steiger-filtered IVW	9	0.08882397	0.0871327	0.3080084	0.5216374	10.23533057	47.77225419	1.471		24.744							
MDD	Traumatic injury	traumainjury	GSMR	8	0.03135586	0.0425616	0.4612949	0.6316192												
MDD	Traumatic injury	traumainjury	LHC		0.92261652	0.0092508	0.00E+00	0.00E+00												
MDD	Triglycerides	tgl	MR Egger	448	-0.0082057	0.029524	0.7811919	0.979695	99.9999608	100	1.039		135.686			0.002	######	######		
MDD	Triglycerides	tgl	Weighted median	448	0.02566996	0.0219811	0.2428799	0.5542643	99.9999608	100	1.039		135.686							
MDD	Triglycerides	tgl	Inverse variance weighte	448	0.07261689	0.0179659	5.30E-05	3.93E-04	99.9999608	100	1.039	0.0489348	135.686	0.980	unweighted				915.118	1.99E-34
MDD	Triglycerides	tgl	Simple mode	448	0.06201752	0.054667	0.25721	0.8804495	99.9999608	100	1.039		135.686							
MDD	Triglycerides	tgl	Weighted mode	448	0.01035548	0.0226345	0.6475281	0.9427278	99.9999608	100	1.039		135.686							
MDD	Triglycerides	tgl	Steiger-filtered IVW	448	0.07261689	0.0179659	5.30E-05	3.93E-04	99.9999608	100	1.039		135.686							
MDD	Triglycerides	tgl	GSMR	428	0.04353216	0.0129239	7.56E-04	0.0028042												
MDD	Triglycerides	tgl	PRESSO	448	0.06464835	0.0166367	1.18E-04	5.52E-04												
MDD	Triglycerides	tgl	LHC		0.05661352	0.0352309	0.1080697	0.2238587												
MDD	Type II diabetes	t2d	MR Egger	181	0.0073756	0.0186338	0.6927099	0.979695	96.62040774	100	1.073		76.271			9.53E-04	0.001	0.440		
MDD	Type II diabetes	t2d	Weighted median	181	0.00392431	0.0096851	0.6853387	0.9383869	96.62040774	100	1.073		76.271							
MDD	Type II diabetes	t2d	Inverse variance weighte	181	0.02035809	0.0081276	0.0122516	0.0474084	96.62040774	100	1.073	0.0147771	76.271	0.932	unweighted				369.737	3.36E-15
MDD	Type II diabetes	t2d	Simple mode	181	0.00915569	0.0249509	0.7140885	0.9748822	96.62040774	100	1.073		76.271							
MDD	Type II diabetes	t2d	Weighted mode	181	-0.0023152	0.0137336	0.8663185	0.966799	96.62040774	100	1.073		76.271							
MDD	Type II diabetes	t2d	Steiger-filtered IVW	181	0.02035809	0.0081276	0.0122516	0.0474084	96.62040774	100	1.073		76.271							
MDD	Type II diabetes	t2d	GSMR	176	0.01316142	0.0057779	0.0227342	0.0623381												
MDD	Type II diabetes	t2d	PRESSO	181	0.01673037	0.0074354	0.0256666	0.0652359												
MDD	Type II diabetes	t2d	LHC		-0.1408313	0.0452844	0.0018713	0.0090447												
MDD	Ulcerative colitis	ulcercolit	MR Egger	56	-0.0116837	0.0151517	0.4440006	0.8781345	100	100	1.028		71.567			0.002	0.002	0.457		
MDD	Ulcerative colitis	ulcercolit	Weighted median	56	0.00206894	0.0062408	0.7402516	0.9469578	100	100	1.028		71.567							
MDD	Ulcerative colitis	ulcercolit	Inverse variance weighte	56	-0.0010268	0.0052214	0.8441051	0.9174889	100	100	1.028	0.0988252	71.567	0.882	unweighted				85.689	0.005
MDD	Ulcerative colitis	ulcercolit	Simple mode	56	0.01033469	0.0127949	0.4227322	0.9353691	100	100	1.028		71.567							
MDD	Ulcerative colitis	ulcercolit	Weighted mode	56	-0.0040903	0.0103418	0.6939977	0.9427278	100	100	1.028		71.567							
MDD	Ulcerative colitis	ulcercolit	Steiger-filtered IVW	56	-0.0010268	0.0052214	0.8441051	0.9174889	100	100	1.028		71.567							
MDD	Ulcerative colitis	ulcercolit	MR Egger SIMEX		-0.0221322	0.0108353	0.045986	0.3065734	100	100	1.028		71.567							
MDD	Ulcerative colitis	ulcercolit	GSMR	55	0.00303091	0.0042575	0.4765266	0.6329981												
MDD	Ulcerative colitis	ulcercolit	PRESSO	56	0.00294783	0.0038795	0.450651	0.5891987												
MDD	Ulcerative colitis	ulcercolit	LHC		0.01530491	0.0112783	0.1747743	0.3305514												
MDD	Variation in diet	dietvariation	MR Egger	11	0.76326884	2.1265739	0.7279313	0.979695	17.08481078	79.33073534	1.303		36.260			-0.001	0.019	0.947		
MDD	Variation in diet	dietvariation	Weighted median	11	0.6234875	0.2733634	0.0225602	0.0956124	17.08481078	79.33073534	1.303		36.260							
MDD	Variation in diet	dietvariation	Inverse variance weighte	11	0.62103003	0.3215434	0.0534332	0.1579318	17.08481078	79.33073534	1.303	0.0010457	36.260	0.062	unweighted				42.081	7.25E-06
MDD	Variation in diet	dietvariation	Simple mode	11	0.82588598	0.5017806	0.1308053	0.5845679	17.08481078	79.33073534	1.303		36.260							
MDD	Variation in diet	dietvariation	Weighted mode	11	0.6228119	0.6213781	0.3398313	0.9334112	17.08481078	79.33073534	1.303		36.260							
MDD	Variation in diet	dietvariation	Steiger-filtered IVW	11	0.62103003	0.3215434	0.0534332	0.1579318	17.08481078	79.33073534	1.303		36.260							
MDD	Variation in diet	dietvariation	GSMR	10	0.74509001	0.1752928	2.13E-05	8.71E-05												
MDD	Variation in diet	dietvariation	PRESSO	11	0.90020525	0.2321436	0.0060703	0.0194887												
MDD	Variation in diet	dietvariation	LHC		0.26940587	0.3958687	0.4961604	0.7098188												
MDD	Vegetable consumption	vegetable	MR Egger	28	0.00130535	0.0292638	0.9647619	0.979695	85.26310598	99.99999999	1.093		21.240			0.002	0.003	0.555		
MDD	Vegetable consumption	vegetable	Weighted median	28	0.0044805	0.0172406	0.7949558	0.9469578	85.26310598	99.99999999	1.093		21.240							
MDD	Vegetable consumption	vegetable	Inverse variance weighte	28	0.01716611	0.0123145	0.1633253	0.3332108	85.26310598	99.99999999	1.093	0.0092897	21.240	0.757	unweighted				26.675	0.481
MDD	Vegetable consumption	vegetable	Simple mode	28	-0.0036541	0.0294836	0.9022846	0.986524	85.26310598	99.99999999	1.093		21.240							
MDD	Vegetable consumption	vegetable	Weighted mode	28	-0.0036541	0.0299658	0.9038488	0.966799	85.26310598	99.99999999	1.093		21.240							
MDD	Vegetable consumption	vegetable	Steiger-filtered IVW	28	0.01716611	0.0123145	0.1633253	0.3332108	85.26310598	99.99999999	1.093		21.240							
MDD	Vegetable consumption	vegetable	MR Egger SIMEX		-0.0104117	0.0361363	0.7755361	0.8971611	85.26310598	99.99999999	1.093		21.240							
MDD	Vegetable consumption	vegetable	GSMR	28	0.01343741	0.012563	0.284799	0.4693909												
MDD	Vegetable consumption	vegetable	LHC		-0.0319557	0.0402204	0.4268959	0.6393529												
MDD	Years ovulating	ovulating	MR Egger	15	-0.0099351	0.0095355	0.3164449	0.86419	77.11396455	99.99999787	1.104		58.394			0.002	0.003	0.628		
MDD	Years ovulating	ovulating	Weighted median	15	-0.0062592	0.0053164	0.2390628	0.5542643	77.11396455	99.99999787	1.104		58.394							
MDD	Years ovulating	ovulating	Inverse variance weighte	15	-0.0056287	0.0039328	0.1523632	0.3228648	77.11396455	99.99999787	1.104	0.0075002	58.394	0.907	unweighted				12.562	0.561
MDD	Years ovulating	ovulating	Simple mode	15	-0.0066367	0.00852	0.4489703	0.9353691	77.11396455	99.99999787	1.104		58.394							
MDD	Years ovulating	ovulating	Weighted mode	15	-0.0073932	0.0067786	0.2938339	0.9017663	77.11396455	99.99999787	1.104		58.394							
MDD	Years ovulating	ovulating	Steiger-filtered IVW	15	-0.0056287	0.0039328	0.1523632	0.3228648	77.11396455	99.99999787	1.104		58.394							
MDD	Years ovulating	ovulating	GSMR	15	-0.0055026	0.0039758	0.1663571	0.3021588												
MDD	Years ovulating	ovulating	LHC		-0.1293317	0.0934978	0.1665853	0.3293845												
MDD	Zinc supplements	zinc	MR Egger	11	0.05890795	0.0637137	0.379311	0.86419	11.55666676	55.5296567	1.419		24.688			-0.004	0.005	0.496		
MDD	Zinc supplements	zinc	Weighted median	11	0.03248626	0.0351043	0.3547474	0.7342446	11.55666676	55.5296567	1.419		24.688							
MDD	Zinc supplements	zinc	Inverse variance weighte	11	0.01800598	0.0265033	0.4968925	0.7249744	11.55666676	55.5296567	1.419	5.97E-04	24.688	0.795	unweighted				11.332	0.332
MDD	Zinc supplements	zinc	Simple mode	11	0.0388242	0.0630643	0.5518926	0.9748822	11.55666676	55.5296567	1.419		24.688							
MDD	Zinc supplements	zinc	Weighted mode	11	0.03239328	0.0621054	0.6133143	0.9427278	11.55666676	55.5296567	1.419		24.688							
MDD	Zinc supplements	zinc	Steiger-filtered IVW	11	0.01800598	0.0265033	0.4968925	0.7249744	11.55666676	55.5296567	1.419		24.688							
MDD	Zinc supplements	zinc	MR Egger SIMEX		-0.0433282	0.0543231	0.4456337	0.8417003	11.55666676	55.5296567	1.419		24.688							
MDD	Zinc supplements	zinc	GSMR	11	0.01552207	0.0254046	0.5412026	0.6881005												
MDD	Zinc supplements	zinc	LHC		0.52347621	0.3194474	0.1012772	0.2149053												

Table S6. Full results for the one-sample MR (OSMR) analyses. All associations have been derived using a classic PRS which contained the same instruments as the TSMR MDD exposure, as well as an LDpred2 PRS takes into account a larger a number of SNP effects. The p-value for the effect is FDR-corrected within the set of results (classic or LDpred2 PRS). Power is derived for effect size =0.1-0.3 (or OR=1.1-1.3). The weak instrument columns indicate if there was weak instrument bias (if p<0.05). The Wu-Hausman column indicates if the result is in line with a causal interpretation of the observed association (p<0.05). The Egger intercept indicates if there is signfiicant pleiotropy (p<0.05).

Trait	Category	Instrument	method	beta	se	p	p_fdr	R2	power	N	N_cases	weakinstrument	p_weakinstruments	wuhausman	p_wuhausman	eggerintercept	p_eggerintercept
Celiac disease*	Disease	PRS_classic	OSMR (2SLS)	-0.095	0.043	0.026	0.073	0.025	4-7	105567	3167	86.606	1.35E-20	2.727	0.099		
Celiac disease*	Disease	PRS_LDPred	OSMR (2SLS)	-0.027	0.006	1.18E-05	2.17E-05	0.094	28-97	105567	3167	4375.730	0.00E+00	0.037	0.848		
Chronic sinusitis*	Disease	PRS_classic	OSMR (2SLS)	-0.016	0.048	0.741	0.852	0.00E+00	4-8	105567	4327	86.606	1.35E-20	0.095	0.758		
Chronic sinusitis*	Disease	PRS_LDPred	OSMR (2SLS)	-0.017	0.007	0.016	0.019	0.024	36-99	105567	4327	4375.730	0.00E+00	4.260	0.039		
Herpes simplex infection*	Disease	PRS_classic	OSMR (2SLS)	-0.038	0.019	0.051	0.116	0.065	3-4	105567	620	86.606	1.35E-20	3.071	0.080		
Herpes simplex infection*	Disease	PRS_LDPred	OSMR (2SLS)	-0.005	0.003	0.053	0.063	0.062	9-41	105567	620	4375.730	0.00E+00	0.099	0.753		
Psoriasis*	Disease	PRS_classic	OSMR (2SLS)	0.074	0.055	0.177	0.278	0.007	4-8	105567	5243	86.606	1.35E-20	3.960	0.047		
Psoriasis*	Disease	PRS_LDPred	OSMR (2SLS)	0.008	0.008	0.278	0.306	0.004	42-100	105567	5243	4375.730	0.00E+00	29.647	5.20E-08		
Stroke*	Disease	PRS_classic	OSMR (2SLS)	-0.034	0.068	0.615	0.752	1.00E-03	4-10	105567	8441	86.606	1.35E-20	0.109	0.742		
Stroke*	Disease	PRS_LDPred	OSMR (2SLS)	-0.028	0.010	0.005	0.006	0.024	58-100	105567	8441	4375.730	0.00E+00	9.108	0.003		
Tinnitus*	Disease	PRS_classic	OSMR (2SLS)	0.053	0.030	0.079	0.159	0.027	3-5	105567	1563	86.606	1.35E-20	4.240	0.039		
Tinnitus*	Disease	PRS_LDPred	OSMR (2SLS)	0.027	0.004	3.76E-10	7.89E-10	0.330	17-78	105567	1563	4375.730	0.00E+00	69.405	8.14E-17		
Varicella*	Disease	PRS_classic	OSMR (2SLS)	-0.001	0.008	0.851	0.936	0.003	3-3	105567	118	86.606	1.35E-20	0.014	0.905		
Varicella*	Disease	PRS_LDPred	OSMR (2SLS)	-0.001	0.001	0.547	0.587	0.023	5-11	105567	118	4375.730	0.00E+00	0.015	0.903		
Cushing's syndrome*	Endocrine	PRS_classic	OSMR (2SLS)	-0.018	0.011	0.097	0.185	0.121	3-3	105567	203	86.606	1.35E-20	2.585	0.108		
Cushing's syndrome*	Endocrine	PRS_LDPred	OSMR (2SLS)	-0.003	0.002	0.082	0.095	0.127	5-17	105567	203	4375.730	0.00E+00	1.493	0.222		
Highschool drop-out	Functional	PRS_classic	OSMR (2SLS)	0.242	0.108	0.025	0.073	0.009	6-18	105567	23570	86.606	1.35E-20	4.100	0.043		
Highschool drop-out	Functional	PRS_LDPred	OSMR (2SLS)	0.368	0.016	1.81E-115	1.33E-114	1.032	89-100	105567	23570	4375.730	0.00E+00	526.037	4.63E-116		
Accidents*	Mortality	PRS_classic	OSMR (2SLS)	-0.503	0.110	4.84E-06	6.81E-05	0.028	7-26	105567	63534	86.606	1.35E-20	0.318	0.573		
Accidents*	Mortality	PRS_LDPred	OSMR (2SLS)	-0.555	0.016	1.13E-263	4.95E-262	1.644	98-100	105567	63534	4375.730	0.00E+00	54.076	1.95E-13		
Alcohol-related death*	Mortality	PRS_classic	OSMR (2SLS)	0.203	0.080	0.012	0.039	0.016	5-12	105567	11505	86.606	1.35E-20	6.774	0.009		
Alcohol-related death*	Mortality	PRS_LDPred	OSMR (2SLS)	0.151	0.011	8.03E-40	2.21E-39	0.434	69-100	105567	11505	4375.730	0.00E+00	187.542	1.21E-42		
Cannabis use disorder*	Risk behav	PRS_classic	OSMR (2SLS)	0.021	0.013	0.105	0.192	0.089	3-4	105567	360	86.606	1.35E-20	2.393	0.122		
Cannabis use disorder*	Risk behav	PRS_LDPred	OSMR (2SLS)	0.005	0.002	0.009	0.012	0.243	7-26	105567	360	4375.730	0.00E+00	3.926	0.048		
Self-harm*	Suicide	PRS_classic	OSMR (2SLS)	0.160	0.045	3.99E-04	0.002	0.061	4-7	105567	3984	86.606	1.35E-20	4.555	0.033		
Self-harm*	Suicide	PRS_LDPred	OSMR (2SLS)	0.140	0.006	4.53E-103	2.49E-102	2.124	34-99	105567	3984	4375.730	0.00E+00	139.925	2.93E-32		
Townsend deprivation index	Socioecon	PRS_classic	OSMR (2SLS)	1.196	0.265	6.19E-06	6.81E-05	0.029	18-87	105441		86.504	1.43E-20	17.421	3.00E-05		
Townsend deprivation index	Socioecon	PRS_LDPred	OSMR (2SLS)	0.847	0.036	2.45E-120	2.15E-119	0.691	100-100	105441		4374.179	0.00E+00	377.775	5.78E-84		
Oily fish intake	Diet	PRS_classic	OSMR (2SLS)	-0.157	0.245	0.521	0.674	0.00E+00	17-87	104509		86.015	1.83E-20	0.031	0.860		
Oily fish intake	Diet	PRS_LDPred	OSMR (2SLS)	-0.193	0.035	4.86E-08	9.30E-08	0.035	100-100	104509		4347.295	0.00E+00	5.237	0.022		
Employment status	Functional	PRS_classic	OSMR (2SLS)	-0.408	0.131	0.002	0.008	0.017	7-24	104201	49725	82.415	1.13E-19	7.694	0.006		
Employment status	Functional	PRS_LDPred	OSMR (2SLS)	-0.326	0.018	2.26E-71	8.28E-71	0.549	97-100	104201	49725	4321.318	0.00E+00	237.134	1.96E-53		
Unable to work	Functional	PRS_classic	OSMR (2SLS)	0.384	0.078	7.42E-07	1.63E-05	0.076	5-11	104201	9889	82.415	1.13E-19	15.680	7.51E-05		
Unable to work	Functional	PRS_LDPred	OSMR (2SLS)	0.315	0.011	4.33E-193	6.35E-192	2.520	64-100	104201	9889	4321.318	0.00E+00	470.034	6.07E-104		
Hospitalizations	Functional	PRS_classic	OSMR (2SLS)	1.770	0.318	2.58E-08	1.14E-06	0.061	15-80	87587		70.863	3.90E-17	30.331	3.66E-08		
Hospitalizations	Functional	PRS_LDPred	OSMR (2SLS)	1.247	0.041	2.44E-201	5.37E-200	1.517	100-100	87587		3681.423	0.00E+00	656.326	4.30E-144		
Age completed education	Cognition	PRS_classic	OSMR (2SLS)	-0.273	0.285	0.338	0.464	1.00E-03	14-74	74507		58.674	1.89E-14	0.552	0.457		
Age completed education	Cognition	PRS_LDPred	OSMR (2SLS)	-0.879	0.043	8.80E-91	3.87E-90	0.794	100-100	74507		2991.805	0.00E+00	425.525	3.17E-94		
Ever used contraceptive pill	Endocrine	PRS_classic	OSMR (2SLS)	0.185	0.128	0.149	0.252	0.007	5-16	55620	43781	48.706	3.02E-12	1.426	0.232		
Ever used contraceptive pill	Endocrine	PRS_LDPred	OSMR (2SLS)	0.056	0.018	0.001	0.002	0.014	84-100	55620	43781	2663.441	0.00E+00	1.654	0.198		
Marital separation/ divorce	Social	PRS_classic	OSMR (2SLS)	0.201	0.103	0.053	0.116	0.027	4-7	54857	4044	31.833	1.69E-08	3.074	0.080		
Marital separation/ divorce	Social	PRS_LDPred	OSMR (2SLS)	0.086	0.012	1.10E-12	2.55E-12	0.283	32-99	54857	4044	2288.148	0.00E+00	24.935	5.95E-07		
Length working week	Functional	PRS_classic	OSMR (2SLS)	-0.342	0.447	0.444	0.592	1.00E-03	10-56	49080		27.888	1.29E-07	0.140	0.708		
Length working week	Functional	PRS_LDPred	OSMR (2SLS)	-0.184	0.056	0.001	0.002	0.013	100-100	49080		1826.459	0.00E+00	0.023	0.880		
Birth weight first child	Reproduction	PRS_classic	OSMR (2SLS)	-0.144	0.360	0.690	0.820	0.00E+00	10-52	44509		35.389	2.73E-09	0.128	0.721		
Birth weight first child	Reproduction	PRS_LDPred	OSMR (2SLS)	-0.157	0.048	0.001	0.002	0.030	100-100	44509		2131.395	0.00E+00	9.301	0.002		
Age voice broke	Endocrine	PRS_classic	OSMR (2SLS)	-0.029	0.394	0.941	0.947	0.00E+00	10-52	44355		39.523	3.28E-10	1.17E-04	0.991		
Age voice broke	Endocrine	PRS_LDPred	OSMR (2SLS)	-0.221	0.063	4.18E-04	6.80E-04	0.033	100-100	44355		1632.503	0.00E+00	10.304	0.001		
Health dissatisfaction	Functional	PRS_classic	OSMR (2SLS)	2.052	0.508	5.44E-05	3.99E-04	0.074	9-49	41707		27.698	1.43E-07	14.491	1.41E-04		
Health dissatisfaction	Functional	PRS_LDPred	OSMR (2SLS)	1.524	0.060	5.76E-140	6.34E-139	2.361	100-100	41707		1676.822	0.00E+00	390.594	2.04E-86		
Fish consumer	Diet	PRS_classic	OSMR (2SLS)	-0.352	0.192	0.068	0.141	0.016	5-11	38238	16663	38.863	4.61E-10	2.273	0.132		
Fish consumer	Diet	PRS_LDPred	OSMR (2SLS)	-0.198	0.030	3.75E-11	8.24E-11	0.210	63-100	38238	16663	1625.611	0.00E+00	19.563	9.77E-06		
Fruit consumer	Diet	PRS_classic	OSMR (2SLS)	-0.344	0.141	0.015	0.047	0.042	5-12	38238	33047	38.863	4.61E-10	5.160	0.023		
Fruit consumer	Diet	PRS_LDPred	OSMR (2SLS)	-0.197	0.021	4.00E-20	1.04E-19	0.565	70-100	38238	33047	1625.611	0.00E+00	57.193	4.07E-14		
Calcium intake	Diet	PRS_classic	OSMR (2SLS)	-0.555	0.408	0.174	0.278	0.007	9-43	35651		35.552	2.51E-09	2.065	0.151		
Calcium intake	Diet	PRS_LDPred	OSMR (2SLS)	-0.023	0.062	0.709	0.726	0.00E+00	100-100	35651		1513.131	0.00E+00	0.387	0.534		
Iron intake	Diet	PRS_classic	OSMR (2SLS)	-0.834	0.415	0.044	0.108	0.015	9-43	35651		35.552	2.51E-09	3.245	0.072		
Iron intake	Diet	PRS_LDPred	OSMR (2SLS)	-0.351	0.063	2.30E-08	4.60E-08	0.112	100-100	35651		1513.131	0.00E+00	14.524	1.39E-04		
Magnesium intake	Diet	PRS_classic	OSMR (2SLS)	-0.503	0.401	0.210	0.309	0.005	9-43	35651		35.552	2.51E-09	1.168	0.280		
Magnesium intake	Diet	PRS_LDPred	OSMR (2SLS)	-0.184	0.062	0.003	0.004	0.030	100-100	35651		1513.131	0.00E+00	3.173	0.075		
Mobility problems	Functional	PRS_classic	OSMR (2SLS)	1.455	0.458	0.002	0.007	0.063	8-35	27669		32.646	1.12E-08	5.736	0.017		
Mobility problems	Functional	PRS_LDPred	OSMR (2SLS)	1.426	0.082	5.58E-67	1.89E-66	1.855	99-100	27669		1063.886	6.03E-227	177.900	2.14E-40		
Pain/discomfort	Functional	PRS_classic	OSMR (2SLS)	2.199	0.511	1.66E-05	1.46E-04	0.137	8-35	27669		32.646	1.12E-08	15.608	7.82E-05		
Pain/discomfort	Functional	PRS_LDPred	OSMR (2SLS)	1.841	0.086	1.51E-100	7.37E-100	2.979	99-100	27669		1063.886	6.03E-227	317.948	1.58E-70		
Problems doing usual activities	Functional	PRS_classic	OSMR (2SLS)	1.686	0.466	3.01E-04	0.002	0.082	8-35	27669		32.646	1.12E-08	7.360	0.007		
Problems doing usual activities	Functional	PRS_LDPred	OSMR (2SLS)	1.616	0.083	1.36E-83	5.44E-83	2.330	99-100	27669		1063.886	6.03E-227	213.871	3.65E-48		
Self-care problems	Functional	PRS_classic	OSMR (2SLS)	0.939	0.432	0.030	0.077	0.026	8-35	27669		32.646	1.12E-08	1.526	0.217		
Self-care problems	Functional	PRS_LDPred	OSMR (2SLS)	1.155	0.080	1.48E-47	4.66E-47	1.206	99-100	27669		1063.886	6.03E-227	100.050	1.70E-23		
Subjective health rating	Functional	PRS_classic	OSMR (2SLS)	-1.735	0.455	1.40E-04	8.77E-04	0.088	8-35	27534		32.731	1.07E-08	6.969	0.008		
Subjective health rating	Functional	PRS_LDPred	OSMR (2SLS)	-1.870	0.084	2.75E-108	1.73E-107	3.136	99-100	27534		1061.977	1.59E-226	289.914	1.62E-64		
Exposure to war	Trauma	PRS_classic	OSMR (2SLS)	0.035	0.112	0.755	0.852	0.006	3-5	26598	1112	21.906	2.89E-06	0.197	0.657		
Exposure to war	Trauma	PRS_LDPred	OSMR (2SLS)	0.064	0.019	5.69E-04	8.94E-04	0.354	13-61	26598	1112	842.104	4.53E-180	19.367	1.09E-05		
Been in serious accident	Trauma	PRS_classic	OSMR (2SLS)	-0.098	0.188	0.602	0.752	0.002	4-7	26583	3824	22.526	2.09E-06	0.234	0.628		
Been in serious accident	Trauma	PRS_LDPred	OSMR (2SLS)	0.113	0.032	3.88E-04	6.57E-04	0.185	28-97	26583	3824	844.472	1.49E-180	15.445	8.53E-05		
Partner violence	Trauma	PRS_classic	OSMR (2SLS)	1.810	0.594	0.002	0.009	0.066	7-34	26524		22.533	2.09E-06	6.739	0.009		
Partner violence	Trauma	PRS_LDPred	OSMR (2SLS)	1.366	0.094	2.14E-47	6.28E-47	1.404	99-100	26524		827.697	4.27E-177	112.567	3.34E-26		
Family relationship satisfaction	Trauma	PRS_classic	OSMR (2SLS)	-0.042	0.556	0.940	0.947	0.00E+00	7-33	25922		21.296	3.97E-06	0.166	0.684		
Family relationship satisfaction	Trauma	PRS_LDPred	OSMR (2SLS)	-0.736	0.093	3.41E-15	8.33E-15	0.434	99-100	25922		816.161	1.31E-174	27.662	1.47E-07		
Number of stillbirths	Reproduction	PRS_classic	OSMR (2SLS)	0.052	0.774	0.947	0.947	0.00E+00	6-26	18976		6.673	0.010	0.005	0.946		
Number of stillbirths	Reproduction	PRS_LDPred	OSMR (2SLS)	0.173	0.069	0.012	0.015	0.053	96-100	18976		906.153	3.07E-193	6.824	0.009		
Pregnancy terminations	Reproduction	PRS_classic	OSMR (2SLS)	0.570	0.462	0.218	0.309	0.025	4-8	18847	8298	6.398	0.011	1.695	0.193		
Pregnancy terminations	Reproduction	PRS_LDPred	OSMR (2SLS)	0.139	0.036	1.15E-04	2.03E-04	0.112	36-99	18847	8298	896.786	2.75E-191	8.925	0.003		
Oestradiol levels	Endocrine	PRS_classic	OSMR (2SLS)	-0.989	0.632	0.118	0.207	0.027	6-21	14342		14.752	1.23E-04	4.512	0.034		
Oestradiol levels	Endocrine	PRS_LDPred	OSMR (2SLS)	-0.036	0.089	0.682	0.714	1.00E-03	89-100	14342		606.914	1.39E-130	6.600	0.010		
Job involves night shifts	Circadian	PRS_classic	OSMR (2SLS)	-1.579	1.278	0.216	0.309	0.022	5-16	10121		4.575	0.032	2.051	0.152		
Job involves night shifts	Circadian	PRS_LDPred	OSMR (2SLS)	-0.019	0.114	0.868	0.868	0.00E+00	76-100	10121		415.813	3.62E-90	0.131	0.717		
Attempted suicide	Suicide	PRS_classic	OSMR (2SLS)	-6.279	84.881	0.941	0.947	0.039	3-4	2124	1355	0.006	0.939	0.257	0.613		
Attempted suicide	Suicide	PRS_LDPred	OSMR (2SLS)	0.291	0.179	0.104	0.117	0.277	8-35	2124	1355	30.740	3.67E-08	0.886	0.347		
Job involves night shifts	Circadian	PRS_classic	MR Egger	-1.161	1.080	0.296			5-16							0.024	0.330
Job involves night shifts	Circadian	PRS_classic	Inverse variance weighted	-0.102	0.209	0.624			5-16								
Job involves night shifts	Circadian	PRS_LDPred	MR Egger	-1.161	1.080	0.296			76-100							0.024	0.330
Job involves night shifts	Circadian	PRS_LDPred	Inverse variance weighted	-0.102	0.209	0.624			76-100								
Age completed education	Cognition	PRS_classic	MR Egger	-0.766	0.486	0.132			14-74							0.015	0.180
Age completed education	Cognition	PRS_classic	Inverse variance weighted	-0.102	0.096	0.286			14-74								
Age completed education	Cognition	PRS_LDPred	MR Egger	-0.766	0.486	0.132			100-100							0.015	0.180
Age completed education	Cognition	PRS_LDPred	Inverse variance weighted	-0.102	0.096	0.286			100-100								
Calcium intake	Diet	PRS_classic	MR Egger	0.374	1.403	0.793			9-43							-0.008	0.780
Calcium intake	Diet	PRS_classic	Inverse variance weighted	-0.020	0.160	0.899			9-43								
Calcium intake	Diet	PRS_LDPred	MR Egger	0.374	1.403	0.793			100-100							-0.008	0.780
Calcium intake	Diet	PRS_LDPred	Inverse variance weighted	-0.020	0.160	0.899			100-100								
Fish consumer	Diet	PRS_classic	MR Egger	0.171	0.323	0.598			5-11							-0.008	0.298
Fish consumer	Diet	PRS_classic	Weighted median	-0.083	0.103	0.421			5-11								
Fish consumer	Diet	PRS_classic	Inverse variance weighted	-0.159	0.070	0.024			5-11								
Fish consumer	Diet	PRS_LDPred	MR Egger	0.178	0.327	0.587			63-100							-0.008	0.282
Fish consumer	Diet	PRS_LDPred	Weighted median	-0.163	0.100	0.103			63-100								
Fish consumer	Diet	PRS_LDPred	Inverse variance weighted	-0.166	0.071	0.020			63-100								
Fruit consumer	Diet	PRS_classic	MR Egger	-0.386	0.444	0.386			5-12							0.002	0.870
Fruit consumer	Diet	PRS_classic	Weighted median	-0.241	0.145	0.096			5-12								
Fruit consumer	Diet	PRS_classic	Inverse variance weighted	-0.315	0.097	0.001			5-12								
Fruit consumer	Diet	PRS_LDPred	MR Egger	-0.333	0.446	0.456			70-100							5.93E-04	0.954
Fruit consumer	Diet	PRS_LDPred	Weighted median	-0.237	0.142	0.095			70-100								
Fruit consumer	Diet	PRS_LDPred	Inverse variance weighted	-0.308	0.098	0.002			70-100								
Iron intake	Diet	PRS_classic	MR Egger	0.320	1.234	0.798			9-43							-0.008	0.770
Iron intake	Diet	PRS_classic	Inverse variance weighted	-0.043	0.140	0.758			9-43								
Iron intake	Diet	PRS_LDPred	MR Egger	0.320	1.234	0.798			100-100							-0.008	0.770
Iron intake	Diet	PRS_LDPred	Inverse variance weighted	-0.043	0.140	0.758			100-100								
Magnesium intake	Diet	PRS_classic	MR Egger	0.578	1.218	0.641			9-43							-0.012	0.643
Magnesium intake	Diet	PRS_classic	Inverse variance weighted	0.009	0.140	0.951			9-43								
Magnesium intake	Diet	PRS_LDPred	MR Egger	0.578	1.218	0.641			100-100							-0.012	0.643
Magnesium intake	Diet	PRS_LDPred	Inverse variance weighted	0.009	0.140	0.951			100-100								
Oily fish intake	Diet	PRS_classic	MR Egger	-0.599	0.484	0.231			17-87							0.014	0.222
Oily fish intake	Diet	PRS_classic	Inverse variance weighted	2.43E-04	0.095	0.998			17-87								
Oily fish intake	Diet	PRS_LDPred	MR Egger	-0.599	0.484	0.231			100-100							0.014	0.222
Oily fish intake	Diet	PRS_LDPred	Inverse variance weighted	2.43E-04	0.095	0.998			100-100								
Celiac disease*	Disease	PRS_classic	MR Egger	-8.781	1.270	1.28E-10			4-7							0.184	4.27E-09
Celiac disease*	Disease	PRS_classic	Weighted median	-0.249	0.170	0.144			4-7								

Celiac disease*	Disease	PRS_classic	Inverse variance weighted	-1.065	0.325	0.001	4-7		
Celiac disease*	Disease	PRS_LDPred	MR Egger	-8.829	1.287	1.78E-10	28-97	0.185	5.28E-09
Celiac disease*	Disease	PRS_LDPred	Weighted median	-0.220	0.180	0.222	28-97		
Celiac disease*	Disease	PRS_LDPred	Inverse variance weighted	-1.058	0.332	0.001	28-97		
Chronic sinusitis*	Disease	PRS_classic	MR Egger	-0.140	0.454	0.758	4-8	0.005	0.666
Chronic sinusitis*	Disease	PRS_classic	Weighted median	0.036	0.145	0.804	4-8		
Chronic sinusitis*	Disease	PRS_classic	Inverse variance weighted	0.052	0.098	0.599	4-8		
Chronic sinusitis*	Disease	PRS_LDPred	MR Egger	-0.215	0.456	0.638	36-99	0.007	0.514
Chronic sinusitis*	Disease	PRS_LDPred	Weighted median	0.037	0.142	0.795	36-99		
Chronic sinusitis*	Disease	PRS_LDPred	Inverse variance weighted	0.076	0.099	0.442	36-99		
Herpes simplex infection*	Disease	PRS_classic	MR Egger	0.591	1.261	0.640	3-4	-0.021	0.476
Herpes simplex infection*	Disease	PRS_classic	Weighted median	0.123	0.377	0.744	3-4		
Herpes simplex infection*	Disease	PRS_classic	Inverse variance weighted	-0.288	0.279	0.302	3-4		
Herpes simplex infection*	Disease	PRS_LDPred	MR Egger	0.813	1.262	0.520	9-41	-0.026	0.382
Herpes simplex infection*	Disease	PRS_LDPred	Weighted median	0.098	0.390	0.801	9-41		
Herpes simplex infection*	Disease	PRS_LDPred	Inverse variance weighted	-0.265	0.281	0.346	9-41		
Psoriasis*	Disease	PRS_classic	MR Egger	2.301	0.610	2.32E-04	4-8	-0.047	0.001
Psoriasis*	Disease	PRS_classic	Weighted median	0.076	0.128	0.556	4-8		
Psoriasis*	Disease	PRS_classic	Inverse variance weighted	0.330	0.138	0.017	4-8		
Psoriasis*	Disease	PRS_LDPred	MR Egger	2.323	0.614	2.23E-04	42-100	-0.048	9.57E-04
Psoriasis*	Disease	PRS_LDPred	Weighted median	0.063	0.133	0.634	42-100		
Psoriasis*	Disease	PRS_LDPred	Inverse variance weighted	0.305	0.140	0.029	42-100		
Stroke*	Disease	PRS_classic	MR Egger	0.035	0.325	0.914	4-10	-0.003	0.667
Stroke*	Disease	PRS_classic	Weighted median	-0.096	0.105	0.362	4-10		
Stroke*	Disease	PRS_classic	Inverse variance weighted	-0.101	0.071	0.151	4-10		
Stroke*	Disease	PRS_LDPred	MR Egger	0.074	0.326	0.821	58-100	-0.004	0.600
Stroke*	Disease	PRS_LDPred	Weighted median	-0.090	0.102	0.380	58-100		
Stroke*	Disease	PRS_LDPred	Inverse variance weighted	-0.093	0.071	0.192	58-100		
Tinnitus*	Disease	PRS_classic	MR Egger	-0.549	0.780	0.482	3-5	0.022	0.232
Tinnitus*	Disease	PRS_classic	Weighted median	0.359	0.238	0.132	3-5		
Tinnitus*	Disease	PRS_classic	Inverse variance weighted	0.365	0.171	0.033	3-5		
Tinnitus*	Disease	PRS_LDPred	MR Egger	-0.461	0.786	0.558	17-78	0.019	0.292
Tinnitus*	Disease	PRS_LDPred	Weighted median	0.350	0.240	0.145	17-78		
Tinnitus*	Disease	PRS_LDPred	Inverse variance weighted	0.349	0.173	0.043	17-78		
Varicella*	Disease	PRS_classic	MR Egger	0.839	2.857	0.769	3-3	-0.006	0.927
Varicella*	Disease	PRS_classic	Weighted median	0.586	0.871	0.501	3-3		
Varicella*	Disease	PRS_classic	Inverse variance weighted	0.584	0.608	0.337	3-3		
Varicella*	Disease	PRS_LDPred	MR Egger	0.810	2.893	0.780	5-11	-0.007	0.921
Varicella*	Disease	PRS_LDPred	Weighted median	0.470	0.888	0.597	5-11		
Varicella*	Disease	PRS_LDPred	Inverse variance weighted	0.530	0.619	0.392	5-11		
Cushing's syndrome*	Endocrine	PRS_classic	MR Egger	-2.199	2.199	0.319	3-3	0.049	0.330
Cushing's syndrome*	Endocrine	PRS_classic	Weighted median	-0.101	0.621	0.871	3-3		
Cushing's syndrome*	Endocrine	PRS_classic	Inverse variance weighted	-0.102	0.475	0.830	3-3		
Cushing's syndrome*	Endocrine	PRS_LDPred	MR Egger	-2.140	2.212	0.335	5-17	0.048	0.343
Cushing's syndrome*	Endocrine	PRS_LDPred	Weighted median	-0.026	0.681	0.970	5-17		
Cushing's syndrome*	Endocrine	PRS_LDPred	Inverse variance weighted	-0.085	0.480	0.860	5-17		
Oestradiol levels	Endocrine	PRS_classic	MR Egger	2.023	1.996	0.324	6-21	-0.038	0.375
Oestradiol levels	Endocrine	PRS_classic	Inverse variance weighted	0.221	0.236	0.350	6-21		
Oestradiol levels	Endocrine	PRS_LDPred	MR Egger	2.023	1.996	0.324	89-100	-0.038	0.375
Oestradiol levels	Endocrine	PRS_LDPred	Inverse variance weighted	0.221	0.236	0.350	89-100		
Employment status	Functional	PRS_classic	MR Egger	-0.511	0.237	0.033	7-24	0.004	0.446
Employment status	Functional	PRS_classic	Weighted median	-0.337	0.069	1.14E-06	7-24		
Employment status	Functional	PRS_classic	Inverse variance weighted	-0.334	0.052	9.65E-11	7-24		
Employment status	Functional	PRS_LDPred	MR Egger	-0.509	0.239	0.035	97-100	0.004	0.463
Employment status	Functional	PRS_LDPred	Weighted median	-0.343	0.070	1.02E-06	97-100		
Employment status	Functional	PRS_LDPred	Inverse variance weighted	-0.337	0.052	1.22E-10	97-100		
Health dissatisfaction	Functional	PRS_classic	MR Egger	0.738	1.309	0.581	9-49	-0.014	0.627
Health dissatisfaction	Functional	PRS_classic	Inverse variance weighted	0.093	0.150	0.537	9-49		
Health dissatisfaction	Functional	PRS_LDPred	MR Egger	0.738	1.309	0.581	100-100	-0.014	0.627
Health dissatisfaction	Functional	PRS_LDPred	Inverse variance weighted	0.093	0.150	0.537	100-100		
Highschool drop-out	Functional	PRS_classic	MR Egger	0.692	0.308	0.026	6-18	-0.006	0.373
Highschool drop-out	Functional	PRS_classic	Weighted median	0.389	0.078	6.93E-07	6-18		
Highschool drop-out	Functional	PRS_classic	Inverse variance weighted	0.423	0.067	3.08E-10	6-18		
Highschool drop-out	Functional	PRS_LDPred	MR Egger	0.686	0.313	0.030	89-100	-0.006	0.393
Highschool drop-out	Functional	PRS_LDPred	Weighted median	0.390	0.074	1.42E-07	89-100		
Highschool drop-out	Functional	PRS_LDPred	Inverse variance weighted	0.425	0.069	5.70E-10	89-100		
Hospitalizations	Functional	PRS_classic	MR Egger	1.515	0.702	0.044	15-80	-0.025	0.108
Hospitalizations	Functional	PRS_classic	Inverse variance weighted	0.337	0.091	2.25E-04	15-80		
Hospitalizations	Functional	PRS_LDPred	MR Egger	1.515	0.702	0.044	100-100	-0.025	0.108
Hospitalizations	Functional	PRS_LDPred	Inverse variance weighted	0.337	0.091	2.25E-04	100-100		
Length working week	Functional	PRS_classic	MR Egger	0.149	0.654	0.822	10-56	-0.003	0.844
Length working week	Functional	PRS_classic	Inverse variance weighted	0.021	0.122	0.862	10-56		
Length working week	Functional	PRS_LDPred	MR Egger	0.149	0.654	0.822	100-100	-0.003	0.844
Length working week	Functional	PRS_LDPred	Inverse variance weighted	0.021	0.122	0.862	100-100		
Mobility problems	Functional	PRS_classic	MR Egger	0.157	1.308	0.906	8-35	0.003	0.928
Mobility problems	Functional	PRS_classic	Inverse variance weighted	0.275	0.156	0.078	8-35		
Mobility problems	Functional	PRS_LDPred	MR Egger	0.157	1.308	0.906	99-100	0.003	0.928
Mobility problems	Functional	PRS_LDPred	Inverse variance weighted	0.275	0.156	0.078	99-100		
Pain/discomfort	Functional	PRS_classic	MR Egger	1.825	1.308	0.179	8-35	-0.030	0.292
Pain/discomfort	Functional	PRS_classic	Inverse variance weighted	0.418	0.156	0.008	8-35		
Pain/discomfort	Functional	PRS_LDPred	MR Egger	1.825	1.308	0.179	99-100	-0.030	0.292
Pain/discomfort	Functional	PRS_LDPred	Inverse variance weighted	0.418	0.156	0.008	99-100		
Problems doing usual activities	Functional	PRS_classic	MR Egger	-1.072	1.317	0.426	8-35	0.028	0.325
Problems doing usual activities	Functional	PRS_classic	Inverse variance weighted	0.250	0.157	0.112	8-35		
Problems doing usual activities	Functional	PRS_LDPred	MR Egger	-1.072	1.317	0.426	99-100	0.028	0.325
Problems doing usual activities	Functional	PRS_LDPred	Inverse variance weighted	0.250	0.157	0.112	99-100		
Self-care problems	Functional	PRS_classic	MR Egger	0.516	1.312	0.698	8-35	-0.006	0.822
Self-care problems	Functional	PRS_classic	Inverse variance weighted	0.219	0.157	0.162	8-35		
Self-care problems	Functional	PRS_LDPred	MR Egger	0.516	1.312	0.698	99-100	-0.006	0.822
Self-care problems	Functional	PRS_LDPred	Inverse variance weighted	0.219	0.157	0.162	99-100		
Subjective health rating	Functional	PRS_classic	MR Egger	-0.238	1.314	0.858	8-35	-0.002	0.943
Subjective health rating	Functional	PRS_classic	Inverse variance weighted	-0.332	0.157	0.034	8-35		
Subjective health rating	Functional	PRS_LDPred	MR Egger	-0.238	1.314	0.858	99-100	-0.002	0.943
Subjective health rating	Functional	PRS_LDPred	Inverse variance weighted	-0.332	0.157	0.034	99-100		
Unable to work	Functional	PRS_classic	MR Egger	1.086	0.325	0.001	5-11	-0.008	0.271
Unable to work	Functional	PRS_classic	Weighted median	0.674	0.104	7.79E-11	5-11		
Unable to work	Functional	PRS_classic	Inverse variance weighted	0.735	0.071	3.12E-25	5-11		
Unable to work	Functional	PRS_LDPred	MR Egger	1.084	0.330	0.001	64-100	-0.008	0.284
Unable to work	Functional	PRS_LDPred	Weighted median	0.652	0.102	1.62E-10	64-100		
Unable to work	Functional	PRS_LDPred	Inverse variance weighted	0.738	0.072	1.49E-24	64-100		
Accidents*	Mortality	PRS_classic	MR Egger	0.185	0.658	0.779	7-26	0.003	0.830
Accidents*	Mortality	PRS_classic	Weighted median	0.403	0.205	0.049	7-26		
Accidents*	Mortality	PRS_classic	Inverse variance weighted	0.323	0.142	0.023	7-26		
Accidents*	Mortality	PRS_LDPred	MR Egger	0.244	0.659	0.712	98-100	0.001	0.942
Accidents*	Mortality	PRS_LDPred	Weighted median	0.225	0.205	0.271	98-100		
Accidents*	Mortality	PRS_LDPred	Inverse variance weighted	0.291	0.143	0.043	98-100		
Alcohol-related death*	Mortality	PRS_classic	MR Egger	0.432	0.303	0.156	5-12	3.36E-04	0.962
Alcohol-related death*	Mortality	PRS_classic	Weighted median	0.383	0.093	3.46E-05	5-12		
Alcohol-related death*	Mortality	PRS_classic	Inverse variance weighted	0.446	0.066	1.10E-11	5-12		
Alcohol-related death*	Mortality	PRS_LDPred	MR Egger	0.376	0.297	0.207	69-100	0.002	0.811
Alcohol-related death*	Mortality	PRS_LDPred	Weighted median	0.380	0.094	4.88E-05	69-100		
Alcohol-related death*	Mortality	PRS_LDPred	Inverse variance weighted	0.446	0.065	5.76E-12	69-100		
Number of stillbirths	Reproduction	PRS_classic	MR Egger	3.194	2.447	0.207	6-26	-0.067	0.209
Number of stillbirths	Reproduction	PRS_classic	Inverse variance weighted	0.044	0.384	0.908	6-26		
Number of stillbirths	Reproduction	PRS_LDPred	MR Egger	3.194	2.447	0.207	96-100	-0.067	0.209
Number of stillbirths	Reproduction	PRS_LDPred	Inverse variance weighted	0.044	0.384	0.908	96-100		
Pregnancy terminations	Reproduction	PRS_classic	MR Egger	-9.598	10.038	0.340	4-8	0.203	0.341
Pregnancy terminations	Reproduction	PRS_classic	Inverse variance weighted	-0.016	0.554	0.977	4-8		
Pregnancy terminations	Reproduction	PRS_LDPred	MR Egger	-9.598	10.038	0.341	36-99	0.203	0.341
Pregnancy terminations	Reproduction	PRS_LDPred	Inverse variance weighted	-0.016	0.554	0.977	36-99		
Cannabis use disorder*	Risk behav	PRS_classic	MR Egger	1.421	1.703	0.405	3-4	-0.024	0.539
Cannabis use disorder*	Risk behav	PRS_classic	Weighted median	0.536	0.507	0.290	3-4		
Cannabis use disorder*	Risk behav	PRS_classic	Inverse variance weighted	0.398	0.367	0.278	3-4		
Cannabis use disorder*	Risk behav	PRS_LDPred	MR Egger	1.603	1.721	0.353	7-26	-0.028	0.477
Cannabis use disorder*	Risk behav	PRS_LDPred	Weighted median	0.527	0.532	0.322	7-26		
Cannabis use disorder*	Risk behav	PRS_LDPred	Inverse variance weighted	0.406	0.373	0.277	7-26		
Marital separation/ divorce	Social	PRS_classic	MR Egger	0.887	0.491	0.073	4-7	-0.014	0.208
Marital separation/ divorce	Social	PRS_classic	Weighted median	0.287	0.158	0.069	4-7		
Marital separation/ divorce	Social	PRS_classic	Inverse variance weighted	0.280	0.107	0.009	4-7		
Marital separation/ divorce	Social	PRS_LDPred	MR Egger	0.988	0.493	0.047	32-99	-0.017	0.140
Marital separation/ divorce	Social	PRS_LDPred	Weighted median	0.288	0.154	0.062	32-99		
Marital separation/ divorce	Social	PRS_LDPred	Inverse variance weighted	0.275	0.108	0.011	32-99		

Townsend deprivation index	Socioecon	PRS_classic	MR Egger	0.571	0.721	0.438	18-87	-0.007	0.641
Townsend deprivation index	Socioecon	PRS_classic	Inverse variance weighted	0.232	0.083	0.005	18-87		
Townsend deprivation index	Socioecon	PRS_LDPred	MR Egger	0.571	0.721	0.438	100-100	-0.007	0.641
Townsend deprivation index	Socioecon	PRS_LDPred	Inverse variance weighted	0.232	0.083	0.005	100-100		
Attempted suicide	Suicide	PRS_classic	MR Egger	1.362	1.622	0.402	3-4	-0.030	0.415
Attempted suicide	Suicide	PRS_classic	Weighted median	-0.147	0.502	0.770	3-4		
Attempted suicide	Suicide	PRS_classic	Inverse variance weighted	0.069	0.351	0.845	3-4		
Attempted suicide	Suicide	PRS_LDPred	MR Egger	1.316	1.628	0.420	8-35	-0.028	0.449
Attempted suicide	Suicide	PRS_LDPred	Weighted median	-0.124	0.502	0.805	8-35		
Attempted suicide	Suicide	PRS_LDPred	Inverse variance weighted	0.110	0.354	0.756	8-35		
Self-harm*	Suicide	PRS_classic	MR Egger	1.085	0.535	0.044	4-7	-0.004	0.721
Self-harm*	Suicide	PRS_classic	Weighted median	0.798	0.162	8.89E-07	4-7		
Self-harm*	Suicide	PRS_classic	Inverse variance weighted	0.898	0.116	9.70E-15	4-7		
Self-harm*	Suicide	PRS_LDPred	MR Egger	1.036	0.537	0.056	34-99	-0.003	0.794
Self-harm*	Suicide	PRS_LDPred	Weighted median	0.770	0.165	2.90E-06	34-99		
Self-harm*	Suicide	PRS_LDPred	Inverse variance weighted	0.899	0.117	1.49E-14	34-99		
Been in serious accident	Trauma	PRS_classic	MR Egger	0.185	0.658	0.779	4-7	0.003	0.830
Been in serious accident	Trauma	PRS_classic	Weighted median	0.403	0.201	0.044	4-7		
Been in serious accident	Trauma	PRS_classic	Inverse variance weighted	0.323	0.142	0.023	4-7		
Been in serious accident	Trauma	PRS_LDPred	MR Egger	0.244	0.659	0.712	28-97	0.001	0.942
Been in serious accident	Trauma	PRS_LDPred	Weighted median	0.225	0.206	0.274	28-97		
Been in serious accident	Trauma	PRS_LDPred	Inverse variance weighted	0.291	0.143	0.043	28-97		
Exposure to war	Trauma	PRS_classic	MR Egger	0.777	1.203	0.519	3-5	-0.006	0.823
Exposure to war	Trauma	PRS_classic	Weighted median	0.440	0.345	0.203	3-5		
Exposure to war	Trauma	PRS_classic	Inverse variance weighted	0.515	0.257	0.045	3-5		
Exposure to war	Trauma	PRS_LDPred	MR Egger	1.026	1.193	0.391	13-61	-0.013	0.641
Exposure to war	Trauma	PRS_LDPred	Weighted median	0.436	0.342	0.203	13-61		
Exposure to war	Trauma	PRS_LDPred	Inverse variance weighted	0.482	0.256	0.060	13-61		
Family relationship satisfaction	Trauma	PRS_classic	MR Egger	-1.039	1.563	0.514	7-33	0.020	0.546
Family relationship satisfaction	Trauma	PRS_classic	Inverse variance weighted	-0.085	0.181	0.640	7-33		
Family relationship satisfaction	Trauma	PRS_LDPred	MR Egger	-1.039	1.563	0.514	99-100	0.020	0.546
Family relationship satisfaction	Trauma	PRS_LDPred	Inverse variance weighted	-0.085	0.181	0.640	99-100		
Partner violence	Trauma	PRS_classic	MR Egger	2.019	2.007	0.327	7-34	-0.036	0.404
Partner violence	Trauma	PRS_classic	Inverse variance weighted	0.318	0.236	0.178	7-34		
Partner violence	Trauma	PRS_LDPred	MR Egger	2.019	2.007	0.327	99-100	-0.036	0.404
Partner violence	Trauma	PRS_LDPred	Inverse variance weighted	0.318	0.236	0.178	99-100		

Table S7. Full results of the observational association between MDD and the traits included in the one-sample MR analyses. Traits derived from hospital records are indicated with a *.

Trait	Category	b	se	p	R2	N	N_cases
Job involves night shifts	Circadian	-0.020	0.023	0.376	0.018	10121	NA
Age completed education	Cognition	-0.104	0.008	0.000	0.032	74507	NA
Fish consumer	Diet	-0.267	0.024	0.000	0.011	38238	16663
Fruit consumer	Diet	-0.295	0.034	0.000	0.055	38238	33047
Oily fish intake	Diet	-0.070	0.007	0.000	0.025	104509	NA
Calcium intake	Diet	0.044	0.012	0.000	0.006	35651	NA
Iron intake	Diet	-0.048	0.012	0.000	0.028	35651	NA
Magnesium intake	Diet	-0.017	0.012	0.166	0.022	35651	NA
Herpes simplex infection*	Disease	-1.027	0.124	0.000	0.012	105567	620
Varicella*	Disease	-0.918	0.265	0.001	0.033	105567	118
Stroke*	Disease	-0.720	0.033	0.000	0.077	105567	8441
Tinnitus*	Disease	-0.646	0.070	0.000	0.008	105567	1563
Chronic sinusitis*	Disease	-1.117	0.048	0.000	0.024	105567	4327
Celiac disease*	Disease	-1.282	0.059	0.000	0.034	105567	3167
Psoriasis*	Disease	-0.863	0.041	0.000	0.017	105567	5243
Cushing's syndrome*	Endocrine	-0.657	0.182	0.000	0.023	105567	203
Ever used contraceptive pill	Endocrine	-0.018	0.024	0.454	0.100	55620	43781
Age voice broke	Endocrine	-0.025	0.012	0.034	0.000	44355	NA
Oestradiol levels	Endocrine	-0.002	0.017	0.925	0.136	14342	NA
Highschool drop-out	Functional	0.303	0.017	0.000	0.088	105567	23570
Employment status	Functional	-0.682	0.017	0.000	0.334	104201	49725
Unable to work	Functional	1.110	0.022	0.000	0.088	104201	9889
Length working week	Functional	-0.089	0.010	0.000	0.169	49080	NA
Health dissatisfaction	Functional	0.476	0.011	0.000	0.051	41707	NA
Hospitalizations	Functional	0.331	0.008	0.000	0.028	87587	NA
Mobility problems	Functional	0.442	0.014	0.000	0.043	27669	NA
Self-care problems	Functional	0.408	0.014	0.000	0.029	27669	NA
Problems doing usual activities	Functional	0.501	0.014	0.000	0.048	27669	NA
Pain/discomfort	Functional	0.474	0.014	0.000	0.050	27669	NA
Subjective health rating	Functional	-0.600	0.014	0.000	0.065	27534	NA
Alcohol-related death*	Mortality	0.148	0.023	0.000	0.063	105567	11505
Accidents*	Mortality	-1.941	0.016	0.000	0.210	105567	63534
Pregnancy terminations	Reproduction	0.048	0.031	0.128	0.043	18847	8298
Birth weight first child	Reproduction	-0.016	0.010	0.111	0.000	44509	NA
Number of stillbirths	Reproduction	0.041	0.015	0.007	0.008	18976	NA
Cannabis use disorder*	Risk behav	0.402	0.112	0.000	0.078	105567	360
Marital separation/ divorce	Social	0.251	0.035	0.000	0.041	54857	4044
Townsend deprivation index	Socioecon	0.182	0.007	0.000	0.020	105441	NA
Self-harm*	Suicide	1.566	0.035	0.000	0.121	105567	3984
Attempted suicide	Suicide	0.486	0.092	0.000	0.025	2124	1355
Been in serious accident	Trauma	-0.085	0.043	0.050	0.043	26583	3824
Exposure to war	Trauma	-0.042	0.079	0.590	0.061	26598	1112
Family relationship satisfaction	Trauma	-0.193	0.015	0.000	0.007	25922	NA
Partner violence	Trauma	0.227	0.014	0.000	0.051	26524	NA
