# Supplementary material for: An encompassing Mendelian randomization study of the causes and consequences of major depressive disorder
Source: Nat Ment Health. 2025 Aug 22;3(9):1002–11. doi: 10.1038/s44220-025-00471-x (PMC12417207; doi:10.1038/s44220-025-00471-x)
Supplement: Supplementary file 2 — Reporting Summary [file 44220_2025_471_MOESM2_ESM.pdf]

nature portfolio

Corresponding author(s): Joëlle A. Pasman

Last updated by author(s): Jun 25, 2025

Reporting Summary

Nature Portfolio wishes to improve the reproducibility of the work that we publish. This form provides structure for consistency and transparency in reporting. For further information on Nature Portfolio policies, see our Editorial Policies and the Editorial Policy Checklist.

Statistics

For all statistical analyses, confirm that the following items are present in the figure legend, table legend, main text, or Methods section.

n/a	Confirmed	
☐	☒	The exact sample size (n) for each experimental group/condition, given as a discrete number and unit of measurement
☒	☐	A statement on whether measurements were taken from distinct samples or whether the same sample was measured repeatedly
☐	☒	The statistical test(s) used AND whether they are one- or two-sided<br>*Only common tests should be described solely by name; describe more complex techniques in the Methods section.*
☐	☒	A description of all covariates tested
☐	☒	A description of any assumptions or corrections, such as tests of normality and adjustment for multiple comparisons
☐	☒	A full description of the statistical parameters including central tendency (e.g. means) or other basic estimates (e.g. regression coefficient) AND variation (e.g. standard deviation) or associated estimates of uncertainty (e.g. confidence intervals)
☐	☒	For null hypothesis testing, the test statistic (e.g. F, t, r) with confidence intervals, effect sizes, degrees of freedom and P value noted<br>*Give P values as exact values whenever suitable.*
☒	☐	For Bayesian analysis, information on the choice of priors and Markov chain Monte Carlo settings
☒	☐	For hierarchical and complex designs, identification of the appropriate level for tests and full reporting of outcomes
☐	☒	Estimates of effect sizes (e.g. Cohen's d, Pearson's r), indicating how they were calculated

Our web collection on statistics for biologists contains articles on many of the points above.

Software and code

Policy information about availability of computer code

Data collection	This study relied on existing datasets, including publicly available GWAS summary statistics and individual-level data from UK-Biobank.
Data analysis	Analyses were conducted using R packages (notably, TwoSampleMR, gsmr, lhcmr, ivreg, OneSampleMR) and genetics software (PLINK, LDpred2). All code developed for this study consisted of standard implementations of these tools and have been made available on Github, https://github.com/KI-PGI/MDD-MR

For manuscripts utilizing custom algorithms or software that are central to the research but not yet described in published literature, software must be made available to editors and reviewers. We strongly encourage code deposition in a community repository (e.g. GitHub). See the Nature Portfolio guidelines for submitting code & software for further information.

Data

Policy information about availability of data

All manuscripts must include a data availability statement. This statement should provide the following information, where applicable:

- Accession codes, unique identifiers, or web links for publicly available datasets
- A description of any restrictions on data availability
- For clinical datasets or third party data, please ensure that the statement adheres to our policy

All GWAS summary statistics used are publicly available, with sources listed in Table S2. The UK-Biobank data source is accessible to all researchers upon application. All code as well as the instrument datasets used for the TSMR are shared on https://github.com/KI-PGI.

Research involving human participants, their data, or biological material

Policy information about studies with human participants or human data. See also policy information about sex, gender (identity/presentation), and sexual orientation and race, ethnicity and racism.

Reporting on sex and gender	Sex and gender were not a main focus of this study. Where appropriate, analyses were controlled for biological sex.
Reporting on race, ethnicity, or other socially relevant groupings	Due to limited availability of genetic data from other ancestry groups, this work was focused on individuals of European descent.
Population characteristics	We included 105,567 individuals from UK-Biobank for the One-Sample MR part of this study (year of birth M=1950, 53% females)
Recruitment	These participants were recruited through invitation letters across the United Kingdom. Participants provided informed consent. Travel expenses were compensated.
Ethics oversight	UK Biobank has approval from the North West Multi-centre Research Ethics Committee (MREC) as a Research Tissue Bank (RTB) approval.

Note that full information on the approval of the study protocol must also be provided in the manuscript.

Field-specific reporting

Please select the one below that is the best fit for your research. If you are not sure, read the appropriate sections before making your selection.

☐ Life sciences ☒ Behavioural & social sciences ☐ Ecological, evolutionary & environmental sciences

For a reference copy of the document with all sections, see nature.com/documents/nr-reporting-summary-flat.pdf

Behavioural & social sciences study design

All studies must disclose on these points even when the disclosure is negative.

Study description	Re-analysis of existing genetic and behavioural data
Research sample	Data sources for the GWASs have been described in the original publications. The UK-Biobank has been described extensively elsewhere. It contains prospective data and electronic health records on ~500,000 individuals aged 40-69 years.
Sampling strategy	UK-Biobank individuals were sampled through GP registrations and random sampling and recruited via invitation letters.
Data collection	Recruitment centers across the UK collect biological samples, interview, and questionnaire data.
Timing	Data used for this study were collected between 2006 and 2014.
Data exclusions	Participants who had withdrawn consent have been excluded from the dataset.
Non-participation	Participation bias in UK-Biobank has been evaluated and reported in detail elsewhere, e.g. pmid 37106081. Generalizability is limited due to some amount of participation bias, with UK-Biobank participants being slightly healthier and more affluent than the average UK citizen.
Randomization	N/A

Reporting for specific materials, systems and methods

We require information from authors about some types of materials, experimental systems and methods used in many studies. Here, indicate whether each material, system or method listed is relevant to your study. If you are not sure if a list item applies to your research, read the appropriate section before selecting a response.

Materials & experimental systems

n/a	Involved in the study
☒	☐ Antibodies
☒	☐ Eukaryotic cell lines
☒	☐ Palaeontology and archaeology
☒	☐ Animals and other organisms
☒	☐ Clinical data
☒	☐ Dual use research of concern
☒	☐ Plants

Methods

n/a	Involved in the study
☒	☐ ChIP-seq
☒	☐ Flow cytometry
☒	☐ MRI-based neuroimaging

Plants

Seed stocks

Report on the source of all seed stocks or other plant material used. If applicable, state the seed stock centre and catalogue number. If plant specimens were collected from the field, describe the collection location, date and sampling procedures.

Novel plant genotypes

Describe the methods by which all novel plant genotypes were produced. This includes those generated by transgenic approaches, gene editing, chemical/radiation-based mutagenesis and hybridization. For transgenic lines, describe the transformation method, the number of independent lines analyzed and the generation upon which experiments were performed. For gene-edited lines, describe the editor used, the endogenous sequence targeted for editing, the targeting guide RNA sequence (if applicable) and how the editor was applied.

Authentication

Describe any authentication procedures for each seed stock used or novel genotype generated. Describe any experiments used to assess the effect of a mutation and, where applicable, how potential secondary effects (e.g. second site T-DNA insertions, mosiacism, off-target gene editing) were examined.
